# Sustainable Protocol for the Synthesis of 2′,3′-Dideoxynucleoside and 2′,3′-Didehydro-2′,3′-dideoxynucleoside Derivatives

**DOI:** 10.3390/molecules27133993

**Published:** 2022-06-21

**Authors:** Virginia Martín-Nieves, Yogesh S. Sanghvi, Susana Fernández, Miguel Ferrero

**Affiliations:** 1 Departamento de Química Orgánica e Inorgánica, Universidad de Oviedo, 33006 Oviedo, Spain; martinvirginia@uniovi.es; 2 Rasayan Inc., Encinitas, CA 92024, USA; ysanghvi@rasayan.us

**Keywords:** 2′,3′-dideoxynucleosides, 2′,3′-didehydro-2′,3′-dideoxynucleosides, synthesis, zalcitabine (ddC), didanosine (ddI), stavudine (d4T)

## Abstract

An improved protocol for the transformation of ribonucleosides into 2′,3′-dideoxynucleoside and 2′,3′-didehydro-2′,3′-dideoxynucleoside derivatives, including the anti-HIV drugs stavudine (d4T), zalcitabine (ddC) and didanosine (ddI), was established. The process involves radical deoxygenation of xanthate using environmentally friendly and low-cost reagents. Bromoethane or 3-bromopropanenitrile was the alkylating agent of choice to prepare the ribonucleoside 2′,3′-bisxanthates. In the subsequent radical deoxygenation reaction, tris(trimethylsilyl)silane and 1,1′-azobis(cyclohexanecarbonitrile) were used to replace hazardous Bu_3_SnH and AIBN, respectively. In addition, TBAF was substituted for camphorsulfonic acid in the deprotection step of the 5′-*O*-silyl ether group, and an enzyme (adenosine deaminase) was used to transform 2′,3′-dideoxyadenosine into 2′,3′-dideoxyinosine (ddI) in excellent yield.

## 1. Introduction

**Figure 1:**
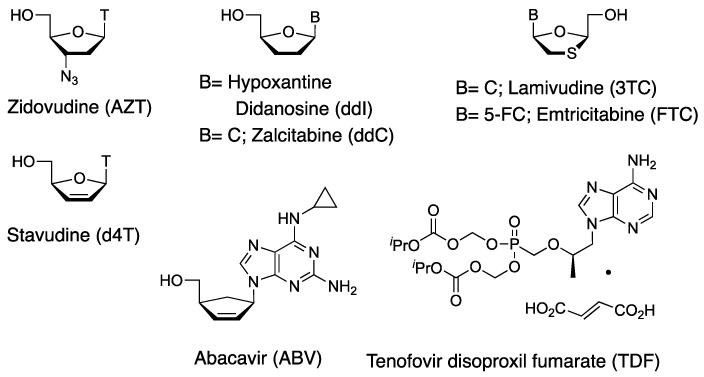
Several approved NRTIs against HIV.

Emerging viruses continue to be a global threat to human health. During the past 25 years, human immunodeficiency virus (HIV), the cause of AIDS, reached virtually every corner of the globe, with 680,000 dying of HIV-related illnesses worldwide in 2020 [1]. More than two-thirds of people infected with HIV live in Asia and Africa. Despite substantial progress in the development of anti-HIV drugs, only 20% of low- and middle-income countries in need of these drugs are receiving them. Among the different anti-HIV chemotherapeutic agents known, the Nucleoside Reverse Transcriptase Inhibitors (NRTI, Figure 1) represent an important class.

Since Mitsuya et al. [2] identified 3′-azido-2′,3′-dideoxythymidine (zidovudine, AZT) as a potent antiviral agent against HIV-1, other nucleoside derivatives showing activity against this virus, such as ddI, ddC, d4T, 3TC, FTC, ABV and TDF, have been successfully developed [3,4]. Most of these compounds are 2′,3′-dideoxynucleosides or 2′,3′-didehydro-2′,3′-dideoxynucleosides and are characterized by lacking hydroxyl groups at the 2′- and 3′-positions.

Various methodologies are reported in the literature for the synthesis of the title compounds. These protocols require formation of the glycosidic bonds [5,6,7,8,9,10,11], the Eastwood procedure [12,13], the Corey–Winter synthesis [14,15,16,17,18], the Barton–McCombie deoxygenation [16,19,20,21,22], the Garegg–Samuelsson reaction [23], photoinduced deoxygenations [24,25], reductive elimination [13,26,27,28,29,30,31,32,33,34,35], or metathesis reaction [36,37]. However, careful review of the literature indicated that the majority of these protocols are not amenable for large-scale production to meet the global demand of antiviral nucleosides. Particularly, some of the methods described involve difficult control of diastereoselectivity in glycosidic bond formation, reagents that are expensive or not environmentally friendly, or partial nucleoside decomposition with loss of the pyrimidine base.

Considering the ongoing challenge of HIV infections in underdeveloped countries, among NRTIs, ddI, ddC and d4T are the most affordable drugs for poor patient populations in Asia and Africa. Our objective is to develop improved protocols that are simple, inexpensive, safe and industrially benign for the large-scale syntheses of these three nucleoside derivatives and their analogs, with different heterocyclic bases. For that purpose, we develop a procedure that involves a Barton–McCombie deoxygenation and the use of commercial ribonucleosides as starting materials.

## 2. Results and Discussion

**Scheme 1:**
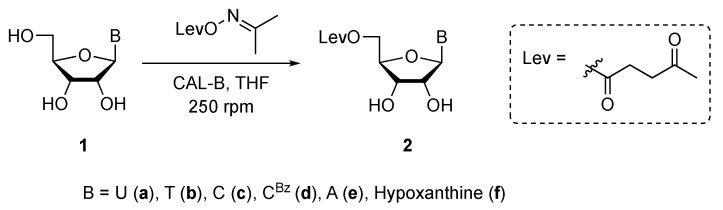
Regioselective enzymatic acylation of **1**.

The selective removal of the hydroxyl groups at the 2′- and 3′-positions of the ribonucleoside requires appropriate protection of the 5′-OH group. Due to prior experience in our group [38,39], we decided to carry out the regioselective enzymatic acylation of the primary hydroxyl with acetonoxime levulinate as an acylating agent and *Candida antarctica* lipase B (CAL-B) as the catalyst. The reactions were performed in THF at 250 rpm, varying the number of equivalents of the acyl donor, the temperature and the substrate concentration, depending on the starting nucleoside (Scheme 1).

Enzymatic acylation of β-d-uridine (**1a**) and β-d-5-methyluridine (**1b**) with 3 equiv of acetonoxime levulinate at 30 °C in the presence of CAL-B afforded the 5′-*O*-levulinyl esters **2a** and **2b** with excellent regioselectivity and high yields in short reaction times (entries 1 and 2, Table 1). However, the reaction with β-d-cytidine (**1c**) is slower, and complete conversion is not achieved, despite using long reaction times, 55 °C instead 30 °C, more dilute conditions, a large excess of acylating agent (9 vs. 3 equiv), and a higher ratio of **1c**:CAL-B, 1:2 (*w*/*w*). This resulted in the undesired acylation of the secondary hydroxyl group (entry 3, Table 1). The low reactivity was attributed to the poor solubility of the starting nucleoside in the reaction mixture. Next, the enzymatic acylation reaction of the base-protected cytidine was attempted. A complete conversion was observed when the same process was carried out with *N^4^*-benzoyl-β-d-cytidine (**1d**), giving rise to the acylated derivative **2d,** with total selectivity and 93% yield (entry 4, Table 1). A moderate selectivity and absence of complete conversion was also observed when the substrate was adenosine (**1e**), which was attributed to the low solubility of this compound in the reaction medium (entry 5, Table 1). In the case of inosine (**1f**), 90 h of reaction time was needed to achieve complete conversion, and although the formation of other acylation products occurred in a low ratio (entry 6, Table 1), compound **2f** was obtained in low yield after column chromatography purification.

**Scheme 2:**
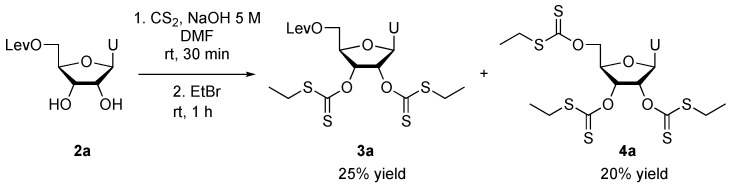
Transformation of 5′-*O*-Lev-uridine into bisxanthates.

Next, transformation of the 5′-*O*-levulinylribonucleoside **2a** into the corresponding bisxanthate was carried out by reaction with carbon disulfide followed by alkylation with bromoethane, a safer and cheaper reagent than other alkylating agents previously used, such as iodomethane or 3-bromopropanenitrile (Scheme 2) [16]. However, the desired bisxanthate **3a** was obtained in a low 25% yield because compound **4a**, resulting from the reaction at the primary hydroxyl, which was deprotected under the reaction conditions (NaOH 5 M), was formed as a by-product. Although different bases (inorganic: *^t^* BuOK, K_2_CO_3_; organic: DIPEA, DBU) were studied as alternatives, the appropriate conditions to carry out the reaction were not found, and the levulinyl group was not pursued as protecting group for the 5′-position.

**Scheme 3:**
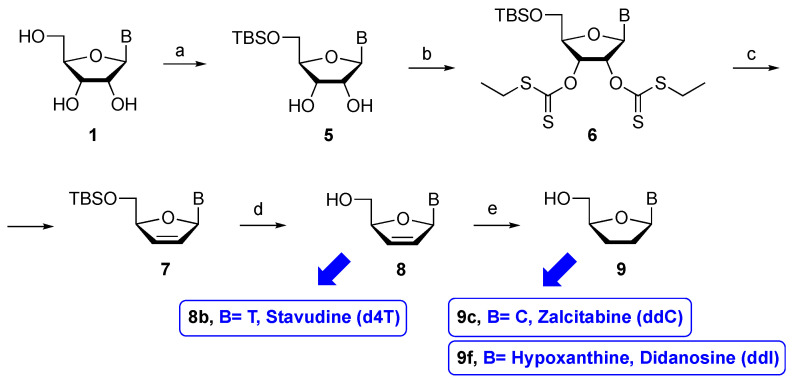
Synthesis of 2′,3′-didehydro-2′,3′-dideoxynucleosides and 2′,3′-dideoxynucleosides. Synthesis of d4T, ddC and ddI. *Reagents and conditions*: (**a**) TBSCl, imidazole, DMF, rt, 12 h; (**b**) (1) CS_2_, 3 M NaOH, DMF, 0 °C, 30 min; (2) EtBr, 0 °C → rt, 20 min; (**c**) Method A: Bu_3_SnH, AIBN, MeCN, reflux, 1 h; Method B: (Me_3_Si)_3_SiH, ACHN, MeCN, reflux, 1 h (**7a**,**b**,**d**,**e**) or 6 h (**7f**); (**d**) Method A: TBAF, THF, 0 °C → rt, 1 h; Method B: (–)-CSA, MeOH, 0 °C → rt, 1 h; (**e**) H_2_, 10% Pd-C, MeOH, rt, 2 h.

Therefore, we elected 5′-*O*-*tert*-butyldimethylsilyl (TBS) as the protecting group of choice due to low cost, high regioselectivity and stability during base treatment. Various ribonucleosides **1** were regioselectively protected at the primary hydroxyl as silyl ethers by treatment with TBSCl and imidazole in DMF for 12 h at room temperature (Scheme 3), furnishing the 5′-*O*-TBS protected nucleosides **5** in high to excellent yields (Table 2). TBS-protected nucleosides **5** were pure enough to carry forward into the next step without further purification by column chromatography. Next, the conversion of **5** to **6** was carefully optimized using the correct combination of the solvent, base, and reaction temperature. The ideal reaction condition calls for the reaction of **5** with CS_2_ in the presence of 3 M aqueous NaOH solution and DMF as solvent for 30 min at 0 °C, and subsequent in situ alkylation with bromoethane for 20 min, affording bisxanthates **6a**–**f** in high yields. It is important to note that compounds **6** were isolated with suitable purity by thorough washing with heptane, avoiding chromatographic purification. We expect the two-step simple chromatography-free protocol for the synthesis of bisxanthates **6a**–**f** will be conducive for scale-up.

Next, we tested the reduction of Bisxanthates **6** using conventional conditions to ensure the formation of desired nucleosides **7**. Using tributyltin hydride (Bu_3_SnH) and 2,2′-azobis(2-methylpropionitrile) (AIBN) in refluxing acetonitrile furnished **7a**,**b**,**d**,**e** in moderate yield (60%) and **7c** in low yield (35%) (Table 2). Interestingly, conversion of the hypoxanthine derivative **6f** resulted in a mixture of products difficult to separate and identify. Next, we sought to find a replacement for the traditional reducing agent Bu_3_SnH, which is toxic, expensive and difficult to remove from the reaction mixture. We elected to use tris(trimethylsilyl)silane [(Me_3_Si)_3_SiH] [40,41] as a greener, non-toxic reagent for reduction. We also replaced hazardous AIBN with a safer radical initiator 1,1′-azobis(cyclohexanecarbonitrile) (ACHN), which has a longer half-life than AIBN. Under optimized reaction conditions, reduction of bisxanthates **6** with green reagents afforded improved yields for uracil, thymine and adenine derivatives furnishing **7a**, **7b** and **7e** in 65%, 75% and 77% yield, respectively. In the case of cytosine, better conversion was observed with the *N*-protected derivative. It is important to note that reaction of the hypoxanthine derivative **6f** with (Me_3_Si)_3_SiH and ACHN allowed the synthesis of 2′,3′-didehydro-2′,3′-dideoxynucleoside **7f** in 80% yield, while its synthesis with Bu_3_SnH was not possible. Thus, the combination of [(Me_3_Si)_3_SiH] and ACHN represents a considerable improvement in the scalable green synthetic strategy proposed for the synthesis of these nucleoside analogs.

Compounds **7** were desilylated with tetrabutylammonium fluoride (TBAF) at room temperature to offer the 2′,3′-didehydro-2′,3′-dideoxynucleosides **8** in excellent yields. Nucleoside **8b** is the antiretroviral drug stavudine (d4T), establishing an efficient route of synthesis. The use of TBAF for deprotection of nucleosides during the final step results in trace contamination of the reagent. Therefore, we searched for an alternative TBS deprotection reagent that is easily removed. Camphorsulfonic acid [(–)-CSA] [42,43] emerged as a reagent of choice; it is an acid derived from camphor that has low sensitivity to air, is compatible with water, and is environmentally friendly. Treatment of **7** with (–)-CSA in MeOH leads to the 2′,3′-didehydro-2′,3′-dideoxynucleosides of uracil and thymine **8a** and **8b** with 92% and 95% yield, respectively. However, this protocol is not suitable for purine derivatives due to the cleavage of the glycosidic bond in the acidic reaction medium. Other TBS deprotection methods using povidone-iodine (PVP-1) [44] or phosphomolybdic acid [45] were not successful.

**Scheme 4:**
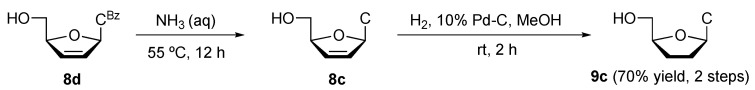
Synthesis of Zalcitabine (ddC) from **8d**.

Hydrogenation of 2′,3′-didehydro-2′,3′-dideoxynucleosides **8** using palladium on carbon in methanol at room temperature afford the corresponding 2′,3′-dideoxynucleosides **9a**,**b**,**e**,**f** in high yields. The reaction of the *N*^4^-benzoylcytidine derivative **8d** was carried out under similar conditions, but it resulted in the formation of a mixture of products. Therefore, we opted to reverse the sequence of the reactions, first carrying out the *N*-benzoyl deprotection by treating **8d** with an aqueous ammonia solution at 55 °C and then performing hydrogenation under the same conditions, isolating the drug zalcitabine (**9c**) with a 70% yield (Scheme 4).

**Scheme 5:**
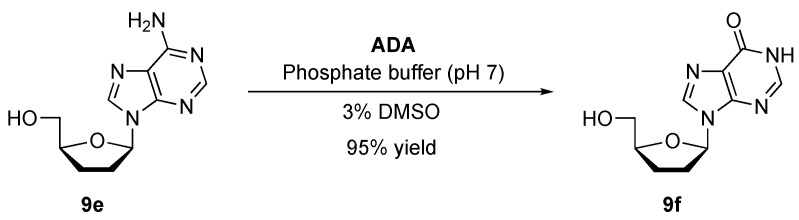
Synthesis of Didanosine (ddI) through enzymatic deamination of **9e**.

Additionally, the drug didanosine (**9f**) was obtained via enzymatic deamination of adenosine analogue **9e** (Scheme 5) [46]. Treatment of **9e** with adenosine deaminase (ADA) in a 0.10 M phosphate buffer (pH 7) and 3% DMSO provides the 2′,3′-dideoxynucleoside **9f** in an almost quantitative yield (95%) after 3 h of reaction.

The structure of the synthesized compounds was determined by NMR spectroscopy. The signals of the ^1^H and ^13^C-NMR spectra of the nucleoside derivatives are fully assigned on the basis of ^1^H and ^13^C chemical shifts, proton coupling constants, and two-dimensional ^1^H-^1^H (COSY) and ^1^H-^13^C spectra (HSQC and HMBC). As an illustrating example, the identification of zalcitabine (**9c**) was performed as follows. The protons H1′, H4′, H5′, H5 and H6 are assigned by ^1^H-NMR. Subsequent analysis of the ^1^H-^13^C HSQC experiment leads to identification of the corresponding carbons. Several multiplets at 1.6–2.5 ppm in the ^1^H-NMR spectrum are assigned, but not identified, to the hydrogens H2′ and H3′. In addition, the signals at 24.7 and 31.9 ppm in the ^13^C-NMR spectrum are assigned to C2′ and C3′. A correlation cross-peak in the ^1^H-^13^C HMBC experiment between the H5′ protons and the carbon at 24.7 ppm allows the assignment of C3′. This has been corroborated by a correlation cross-peak between H1′ and C3′. Further analysis of the ^1^H-^13^C HSQC experiment leads to unambiguously identification of H2 and H3. Finally, a correlation cross-peak between H1′ and the signal at 157.3 ppm in the ^1^H-^13^C HMBC experiment allows the assignment of C2, being the signal of the ^13^C-NMR spectrum at 165.9 ppm, which does not appear in the DEPT-135 experiment, identified as C4. The COSY experiment validates the assignment made. It is worth mentioning the three-bond correlation of H1′ with the two hydrogens H2′, but not with H3′, as well as the three-bond correlation of H4′ with the two hydrogens of H3′, but not with H2′.

## 3. Materials and Methods

### 3.1. General

All chemical reagents were purchased from Aldrich, Sigma, Merck, Acros or Alfa Aesar, and used without further purification. Thin-layer chromatography (TLC) was carried out on aluminum-backed Silica-Gel 60 F_254_ plates. The spots were visualized with UV light. Column chromatography was performed using Silica Gel (60 Å, 230 × 400 mesh).

*Candida antarctica* lipase type B (CAL-B, Novozyme 435, immobilized by adsorption in Lewatit, 9120 PLU/g) was purchased from Novozymes. Adenosine deaminase (ADA, 2–5 units/mg, intestinal bovine source, lyophilized) was purchased from Creative Enzymes.

NMR spectra were measured on Bruker DPX-300 (^1^H 300.13 MHz and ^13^C 75.5 MHz). High resolution mass spectra (HRMS) were recorded on a Bruker MicrOTOF-Q mass spectrometer under electron spray ionization (ESI). Melting points were recorded on a Gallemkamp apparatus with samples in open capillary tubes. Full analytical data for new compounds are available in the Supporting Information.

The structure of the synthesized compounds was determined by NMR spectroscopy. The signals of the ^1^H and ^13^C-NMR spectra are fully assigned on the basis of ^1^H and ^13^C chemical shifts, proton coupling constants, and two-dimensional ^1^H-^1^H (COSY) and ^1^H-^13^C spectra (HSQC and HMBC). Full NMR data are available in the Supporting Information. The level of purity is indicated by the inclusion of copies of ^1^H, ^13^C, DEPT and 2D NMR spectra.

### 3.2. General Procedure for Enzymatic Acylation of ***1*** Synthesis of ***2***

Anhydrous THF was added to an Erlenmeyer flask containing ribonucleoside **1** (0.2 mmol), acetonoxime levulinate and CAL-B (acylating agent equiv, enzyme ratio, concentration, temperature, and reaction time are indicated in Table 1) under nitrogen. The reaction was stirred at 250 rpm and followed by TLC (10% MeOH/CH_2_Cl_2_). Next, the enzyme was filtered and washed with CH_2_Cl_2_ and MeOH, and the solvents were removed under reduced pressure. The reaction crude was purified by column chromatography (gradient eluent: 2–5% MeOH/CH_2_Cl_2_), obtaining the corresponding acylated ribonucleosides **2a**–**f** (yields are indicated in Table 1).

5′-*O*-Levulinyl-β-d-uridine (**2a**). White solid, mp: 60–62 °C. *R_f_*: 0.32 (10% MeOH/CH_2_Cl_2_). HRMS (ESI^+^, *m/z*): Calcd. for C_14_H_19_N_2_O_8_ [M + H]^+^: 343.1136. Found: 343.1131.

5′-*O*-Levulinyl-β-d-5-methyluridine (**2b**). White solid, mp: 134–136 °C. *R_f_*: 0.33 (10% MeOH/CH_2_Cl_2_). HRMS (ESI^+^, *m/z*): Calcd. for C_15_H_21_N_2_O_8_ [M + H]^+^: 357.1292. Found: 357.1279.

5′-*O*-Levulinyl-β-d-cytidine (**2c**). White solid, mp: 53–55 °C. *R_f_*: 0.35 (20% MeOH/CH_2_Cl_2_). HRMS (ESI^+^, *m/z*): Calcd. for C_14_H_20_N_3_O_7_ [M + H]^+^: 342.1296. Found: 342.1295.

*N*^4^-Benzoyl-5′-*O*-levulinyl-β-d-cytidine (**2d**). White solid, mp: 193–195 °C. *R_f_*: 0.47 (10% MeOH/CH_2_Cl_2_). HRMS (ESI^+^, *m/z*): Calcd. for C_21_H_24_N_3_O_8_ [M + H]^+^: 446.1563. Found: 446.1564.

5′-*O*-Levulinyl-β-d-adenosine (**2e**). White solid, mp: 116 °C (decompose). *R_f_*: 0.26 (10% MeOH/CH_2_Cl_2_). HRMS (ESI^+^, *m/z*): Calcd. for C_15_H_20_N_5_O_6_ [M + H]^+^: 366.1408. Found: 366.1406.

5′-*O*-Levulinyl-β-d-inosine (**2f**). White solid, mp: 54–56 °C. *R_f_*: 0.44 (20% MeOH/CH_2_Cl_2_). HRMS (ESI^+^, *m/z*): Calcd. for C_15_H_19_N_4_O_7_ [M + H]^+^: 367.1248. Found: 367.1253.

### 3.3. Synthesis of ***5***

To a solution of ribonucleoside **1** (0.4 M for **1a**,**b** and 0.2 M for **1c**–**f**) in anhydrous DMF were added imidazole (2.4 equiv) and TBSCl (1.2 equiv). The mixture was stirred at rt for 12 h. Then, the residue was poured into EtOAc and washed with water. The organic phase was dried, filtered and evaporated under reduced pressure. Compounds **5** were obtained with sufficient purity for the next step and the following yields: 93% for **5a**, 85% for **5b**, 91% for **5c**, 80% for **5d**, 85% for **5e** and 80% for **5f**. If desired, a chromatographic column could be performed (gradient eluent: 5–10% MeOH/CH_2_Cl_2_).

5′-*O*-(*tert*-Butyldimethylsilyl)-β-d-uridine (**5a**). White solid, mp: 94–96 °C. *R_f_*: 0.41 (10% MeOH/CH_2_Cl_2_). HRMS (ESI^+^, *m/z*): Calcd. for C_15_H_27_N_2_O_6_Si [M + H]^+^: 359.16329. Found: 359.16332.

5′-*O*-(*tert*-Butyldimethylsilyl)-β-d-5-methyluridine (**5b**). White solid, mp: 197–198 °C. *R_f_*: 0.35 (10% MeOH/CH_2_Cl_2_). HRMS (ESI^+^, *m/z*): Calcd. for C_16_H_29_N_2_O_6_Si [M + H]^+^: 373.1789. Found: 373.1790.

5′-*O*-(*tert*-Butyldimethylsilyl)-β-d-cytidine (**5c**). Colorless foam. *R_f_*: 0.52 (10% MeOH/CH_2_Cl_2_). HRMS (ESI^+^, *m/z*): Calcd. for C_15_H_28_N_3_O_5_Si [M + H]^+^: 358.1798. Found: 358.1791.

*N*^4^-Benzoyl-5′-*O*-(*tert*-butyldimethylsilyl)-β-d-cytidine (**5d**). White solid, mp: 86–88 °C. *R_f_*: 0.47 (10% MeOH/CH_2_Cl_2_). HRMS (ESI^+^, *m/z*): Calcd. for C_22_H_31_N_3_O_6_Si [M + H]^+^: 462.2055. Found: 462.2048.

5′-*O*-(*tert*-Butyldimethylsilyl)-β-d-adenosine (**5e**). White solid, mp: 178–179 °C. *R_f_*: 0.33 (10% MeOH/CH_2_Cl_2_). HRMS (ESI^+^, *m/z*): Calcd. for C_16_H_28_N_5_O_4_Si [M + H]^+^: 382.1911. Found: 382.1902.

5′-*O*-(*tert*-Butyldimethylsilyl)-β-d-inosine (**5f**). White solid, mp: 229–230 °C. *R_f_*: 0.17 (10% MeOH/CH_2_Cl_2_). HRMS (ESI^+^, *m/z*): Calcd. for C_16_H_27_N_4_O_5_Si [M + H]^+^: 383.1745. Found: 383.1743.

### 3.4. Synthesis of ***6***

To a solution of 5′-*O*-silyl protected ribonucleosides **5** and CS_2_ (7 equiv) in DMF (0.4 M) at 0 °C, an aqueous 3 M NaOH solution (3 equiv) was added dropwise. After being stirred for 30 min at this temperature, bromoethane (15 equiv) was added dropwise, and stirring continued for 20 min at rt. Then, the residue was poured into EtOAc and washed with water. The organic phase was dried, filtered, and evaporated under reduced pressure. The resulting solid was thoroughly washed with heptane to afford compounds **6** with suitable purity, avoiding chromatographic purification. Yields: 82% for **6a**, 81% for **6b**, 75% for **6c**, 72% for **6d**, 90% for **6e** and 70% for **6f**.

5′-*O*-(*tert*-Butyldimethylsilyl)-2′,3′-bis-*O*-[(ethylthio)thiocarbonyl]-β-d-uridine (**6a**). White solid, mp: 102–104 °C. *R_f_*: 0.45 (40% EtOAc/hexane). HRMS (ESI^+^, *m/z*): Calcd. for C_21_H_35_N_2_O_6_S_4_Si [M + H]^+^: 567.1142. Found: 567.1133.

5′-*O*-(*tert*-Butyldimethylsilyl)-2′,3′-bis-*O*-[(ethylthio)thiocarbonyl]-β-d-5-methyluridine (**6b**). White solid, mp: 131–132 °C. *R_f_*: 0.50 (40% EtOAc/hexane). HRMS (ESI^+^, *m/z*): Calcd. for C_22_H_37_N_2_O_6_S_4_Si [M + H]^+^: 581.1298. Found: 581.1292.

5′-*O*-(*tert*-Butyldimethylsilyl)-2′,3′-bis-*O*-[(ethylthio)thiocarbonyl]-β-d-cytidine (**6c**). White solid, mp: 99–101 °C. *R_f_*: 0.35 (10% MeOH/CH_2_Cl_2_). HRMS (ESI^+^, *m/z*): Calcd. for C_21_H_36_N_3_O_5_S_4_Si [M + H]^+^: 566.1302. Found: 566.1295.

*N*^4^-Benzoyl-5′-*O*-(*tert*-butyldimethylsilyl)-2′,3′-bis-*O*-[(ethylthio)thiocarbonyl]-β-d-cytidine (**6d**). White solid, mp: 140–141 °C. *R_f_*: 0.25 (40% EtOAc/hexane). HRMS (ESI^+^, *m/z*): Calcd. for C_28_H_40_N_3_O_6_S_4_Si [M + H]^+^: 670.1564. Found: 670.1558.

5′-*O*-(*tert*-Butyldimethylsilyl)-2′,3′-bis-*O*-[(ethylthio)thiocarbonyl]-β-d-adenosine (**6e**). White solid, mp: 164–165 °C. *R_f_*: 0.18 (50% EtOAc/hexane). HRMS (ESI^+^, *m/z*): Calcd. for C_22_H_36_N_5_O_4_S_4_Si [M + H]^+^: 590.1414. Found: 590.1409.

5′-*O*-(*tert*-Butyldimethylsilyl)-2′,3′-bis-*O*-[(ethylthio)thiocarbonyl]-β-d-inosine (**6f**). White solid, mp: 201–203 °C. *R_f_*: 0.36 (10% MeOH/CH_2_Cl_2_). HRMS (ESI^+^, *m/z*): Calcd. for C_22_H_36_N_4_O_5_S_4_Si [M + H]^+^: 591.1254. Found: 591.1239.

### 3.5. Synthesis of ***7***

#### 3.5.1. Method A: Bu_3_SnH

To a solution of **6** in anhydrous MeCN (0.13 M) at reflux was added dropwise a solution of Bu_3_SnH (4 equiv) and AIBN (0.4 equiv) in anhydrous MeCN (0.5 M). After being stirred for 1 h at this temperature, the solvent was removed under vacuum, and the residue was purified by column chromatography (gradient eluents: 40–50% EtOAc/hexane for **7a**,**b**; 70% EtOAc/hexane-EtOAc for **7d**; 2–5% MeOH/CH_2_Cl_2_ for **7c**,**e**) to afford **7a**, **7b**, **7d** and **7e** in 60% yield and **7c** in 35% yield.

#### 3.5.2. Method B: (Me_3_Si)_3_SiH

To a solution of **6** in anhydrous MeCN (0.13 M) at reflux, was added dropwise a solution of (Me_3_Si)_3_SiH (4 equiv) and 1,1′-azobis(cyclohexanecarbonitrile) (0.4 equiv) in anhydrous MeCN (0.5 M). The mixture was stirred for 1 h (**6a**–**e**) or 6 h (**6f**) at this temperature. Next, the solvent was removed under vacuum, and the residue was purified by column chromatography (gradient eluents: 40–50% EtOAc/hexane for **7a**,**b**; 70% EtOAc/hexane-EtOAc for **7d**; 2–5% MeOH/CH_2_Cl_2_ for **7e**,**f**) to afford **7** (65% for **7a**, 75% for **7b**, 40% for **7d**, 77% for **7e** and 80% yield for **7f**).

5′-*O*-(*tert*-Butyldimethylsilyl)-2′,3′-didehydro-2′,3′-dideoxy-β-d-uridine (**7a**). White solid, mp: 166–168 °C. *R_f_*: 0.16 (40% EtOAc/hexane). HRMS (ESI^+^, *m/z*): Calcd. for C_15_H_25_N_2_O_4_Si [M + H]^+^: 325.1578. Found: 325.1573.

5′-*O*-(*tert*-Butyldimethylsilyl)-2′,3′-didehydro-3′-deoxy-β-d-5-thymidine (**7b**). White solid, mp: 169–171 °C. *R_f_*: 0.29 (40% EtOAc/hexane). HRMS (ESI^+^, *m/z*): Calcd. for C_16_H_27_N_2_O_4_Si [M + H]^+^: 339.1735. Found: 339.1729.

5′-*O*-(*tert*-Butyldimethylsilyl)-2′,3′-didehydro-2′,3′-dideoxy-β-d-cytidine (**7c**). White solid, mp: 176–178 °C. *R_f_*: 0.52 (10% MeOH/CH_2_Cl_2_). HRMS (ESI^+^, *m/z*): Calcd. for C_15_H_26_N_3_O_3_Si [M + H]^+^: 324.1738. Found: 324.1743.

*N*^4^-Benzoyl-5′-*O*-(*tert*-butyldimethylsilyl)-2′,3′-didehydro-2′,3′-dideoxy-β-d-cytidine (**7d**). White solid, mp: 137–138 °C. *R_f_*: 0.19 (40% EtOAc/hexane). HRMS (ESI^+^, *m/z*): Calcd. for C_22_H_30_N_3_O_4_Si [M + H]^+^: 428.2000. Found: 428.1993.

5′-*O*-(*tert*-Butyldimethylsilyl)-2′,3′-didehydro-2′,3′-dideoxy-β-d-adenosine (**7e**). White solid, mp: 118–120 °C. *R_f_*: 0.48 (10% MeOH/CH_2_Cl_2_). HRMS (ESI^+^, *m/z*): Calcd. for C_16_H_26_N_5_O_2_Si [M + H]^+^: 348.1850. Found: 348.1848.

5′-*O*-(*tert*-Butyldimethylsilyl)-2′,3′-didehydro-2′,3′-dideoxy-β-d-inosine (**7f**). White solid, mp: 178–180 °C. *R_f_*: 0.36 (10% MeOH/CH_2_Cl_2_). HRMS (ESI^+^, *m/z*): Calcd. for C_16_H_25_N_4_O_3_Si [M + H]^+^: 349.1690. Found: 349.1690.

### 3.6. Synthesis of ***8***

#### 3.6.1. Method A: TBAF

TBAF (2 equiv, 1.0 M in THF) was added dropwise to a stirred solution of **7** (1 equiv) in anhydrous THF (0.1 M) at 0 °C. After 5 min, the ice bath was removed, and the reaction mixture was stirred at rt for 1 h. Next, the solvent was removed under vacuum, and the residue was purified by column chromatography (5% MeOH/CH_2_Cl_2_ for **8a**,**b**,**d**; 15% MeOH/CH_2_Cl_2_ for **8e**,**f**) to afford **8a**,**e** in 95%, **8b**,**d** in 90%, and **8f** in 75% yields.

#### 3.6.2. Method B: (–)-CSA

(–)-CSA (1 equiv) was added to a solution of **7** in anhydrous MeOH (0.1 M) at 0 °C, and the reaction was stirred at rt for 1 h. Solid NaHCO_3_ was then added, and the mixture was stirred for a further 5 min. Next, the solvent was removed under vacuum, and the residue was purified by column chromatography (5% MeOH/CH_2_Cl_2_) to afford **8a** in 92% and **8b** in 95% yields.

2′,3′-Didehydro-2′,3′-dideoxy-β-d-uridine (**8a**). White solid, mp: 154–155 °C. *R_f_*: 0.40 (10% MeOH/CH_2_Cl_2_). HRMS (ESI^+^, *m/z*): Calcd. for C_9_H_10_N_2_NaO_4_ [M + Na]^+^: 233.0533. Found: 233.0537.

2′,3′-Didehydro-3′-deoxy-β-d-5-thymidine (**8b**). White solid, mp: 165–166 °C. *R_f_*: 0.42 (10% MeOH/CH_2_Cl_2_). HRMS (ESI^+^, *m/z*): Calcd. for C_10_H_13_N_2_O_4_ [M + H]^+^: 225.0870. Found: 225.0873.

*N*^4^-Benzoyl-2′,3′-didehydro-2′,3′-dideoxy-β-d-cytidine (**8d**). White solid, mp: 280 °C (decompose). *R_f_*: 0.66 (10% MeOH/CH_2_Cl_2_). HRMS (ESI^+^, *m/z*): Calcd. for C_16_H_16_N_3_O_4_ [M + H]^+^: 314.1135. Found: 314.1140.

2′,3′-Didehydro-2′,3′-dideoxy-β-d-adenosine (**8e**). White solid, mp: 185–186 °C. *R_f_*: 0.24 (10% MeOH/CH_2_Cl_2_). HRMS (ESI^+^, *m/z*): Calcd. for C_10_H_12_N_5_O_2_ [M + H]^+^: 234.0986. Found: 234.0984.

2′,3′-Didehydro-2′,3′-dideoxy-β-d-inosine (**8f**). White solid, mp: >300 °C. *R_f_*: 0.19 (10% MeOH/CH_2_Cl_2_). HRMS (ESI^+^, *m/z*): Calcd. for C_10_H_11_N_4_O_3_ [M + H]^+^: 235.0826. Found: 235.0826.

### 3.7. Synthesis of ***9***

A flask containing **8** and 10% Pd/C (0.1 equiv) was exposed to a positive pressure of hydrogen gas (balloon). Anhydrous MeOH (0.02M) was added, and the mixture was stirred vigorously for 2 h under a hydrogen atmosphere. The suspension was filtered on Celite^®®^ and washed with MeOH, and the solvent was removed under vacuum. The crude was purified by column chromatography (gradient eluent 2–10% MeOH/CH_2_Cl_2_) to afford **9a** in 82%, **9b** in 87%, **9e** in 88%, and **9f** in 80% yields.

2′,3′-Dideoxy-β-d-uridine (**9a**). White solid, mp: 116–117 °C. *R_f_*: 0.42 (10% MeOH/CH_2_Cl_2_). HRMS (ESI^+^, *m/z*): Calcd. for C_9_H_13_N_2_O_4_ [M + H]^+^: 213.0870. Found: 213.0875.

3′-Deoxy-β-d-5-thymidine (**9b**). White solid, mp: 155–156 °C. *R_f_*: 0.44 (10% MeOH/CH_2_Cl_2_). HRMS (ESI^+^, *m/z*): Calcd. for C_10_H_15_N_2_O_4_ [M + H]^+^: 227.1026. Found: 227.1032.

2′,3′-Dideoxy-β-d-adenosine (**9e**). White solid, mp: 186–188 °C. *R_f_*: 0.33 (10% MeOH/CH_2_Cl_2_). HRMS (ESI^+^, *m/z*): Calcd. for C_10_H_14_N_5_O_2_ [M + H]^+^: 236.1142. Found: 236.1148.

2′,3′-Dideoxy-β-d-inosine (**9f**). White solid, mp: 160–163 °C. *R_f_*: 0.28 (10% MeOH/CH_2_Cl_2_). HRMS (ESI^+^, *m/z*): Calcd. for C_10_H_13_N_4_O_3_ [M + H]^+^: 237.0982. Found: 237.0989.

#### 3.7.1. Synthesis of Zalcitabine (**9c**)

A suspension of **8d** (50 mg, 0.16 mmol) in an aqueous 32% NH_3_ solution (2.5 mL) was stirred at 55 °C for 12 h. The solvent was removed under vacuum. Then, a mixture of the resulting residue and 10% Pd/C (17 mg) was exposed to a positive pressure of hydrogen gas (balloon). Anhydrous MeOH (8 mL) was added, and the mixture was stirred vigorously for 2 h under a hydrogen atmosphere. The suspension was filtered on Celite^®®^ and washed with MeOH, and the solvent was removed under vacuum. The crude was purified by column chromatography (20% MeOH/CH_2_Cl_2_) previously packed with silica gel using a 10% Et_3_N solution in MeOH:CH_2_Cl_2_ (2:8, *v*:*v*). Compound **9c** was isolated in 70% yield.

2′,3′-Dideoxy-β-d-cytidine (**9c**). White solid, mp: 208–210 °C. *R_f_*: 0.27 (20% MeOH/CH_2_Cl_2_). HRMS (ESI^+^, *m/z*): Calcd. for C_9_H_14_N_3_O_3_ [M + H]^+^: 212.1030. Found: 212.1034.

#### 3.7.2. Synthesis of Didanosine (**9f**)

To a suspension of **9e** (40 mg, 0.17 mmol) in a phosphate buffer solution pH 7 (0.8 mL) and 3% of DMSO, to promote dissolution, 2 mg of adenosine deaminase dissolved in the same buffer (0.2 mL) was added. The reaction was stirred at 250 rpm and 30 °C for 3 h. The crude was purified by column chromatography (10% MeOH/CH_2_Cl_2_) to afford **9f** in 95% yield.

## 4. Conclusions

We report an economical and green synthesis of 2′,3′-dideoxynucleoside and 2′,3′-didehydro-2′,3′-dideoxynucleoside derivatives of uracil, thymine, cytosine, adenine and hypoxanthine through deoxygenation of the corresponding 2′,3′-*O*-bisxanthate ribonucleosides. This protocol involves the use of tris(trimethylsilyl)silane [(Me_3_Si)_3_SiH] instead of Bu_3_SnH, which is toxic, expensive and difficult to remove from the reaction mixture, as a radical-based reducing agent. We also replaced potentially explosive AIBN with 1,1′-azobis(cyclohexanecarbonitrile) (ACHN) as a safer alternative. In addition, for the deprotection of silyl ethers at the 5′-position of the nucleosides, we were able to substitute TBAF for camphorsulfonic acid as a more sustainable reagent, in pyrimidine derivatives. The use of (Me_3_Si)_3_SiH in the deoxygenation of bisxanthate hypoxanthine derivative allows easy access to 2′,3′-didehydro-2′,3′-dideoxyinosine, an antiviral agent. As an alternative synthesis, this nucleoside was also obtained in excellent yield via enzymatic deamination of 2′,3′-dideoxyadenosine with adenosine deaminase. It is important to emphasize that these protocols may have potential industrial application for the synthesis of three of the most demanding anti-HIV drugs: stavudine (d4T), zalcitabine (ddC) and didanosine (ddI).

## Figures and Tables

**Table 1:** Regioselective enzymatic acylation of ribonucleosides **1**.

Entry	Substrate	T (°C)	conc (M)	t (h)	1 (%) *^a^*	2 (%) *^a,b^*	Other Acylated Compounds (%) *^a^*
1	**1a** *^c^*	30	0.1	2	-	>97 (*80*)	-
2	**1b** *^c^*	30	0.1	2.5	-	>97 (*78*)	-
3	**1c** *^d^*	55	0.025	54	26	53 (*50*)	21
4	**1d** *^d^*	55	0.025	24	-	>97 (*93*)	-
5	**1e** *^d^*	55	0.025	48	10	70 (*42*)	20
6	**1f** *^d^*	55	0.025	90	-	87 (*40*)	13

*^a^* Based on ^1^H-NMR signal integration. *^b^* Percentage of isolated yields are given in parenthesis. *^c^* 3 equiv of acetonoxime levulinate and ratio **1**:CAL-B, 1:1 (*w*/*w*). *^d^* 9 equiv of acetonoxime levulinate and ratio **1**:CAL-B, 1:2 (*w*/*w*).

**Table 2:** Reaction yields of **5**, **6**, **7**, **8** and **9**.

			6→7		7→8		
B	1→5	5→6	Bu_3_SnH	(Me_3_Si)_3_SiH	TBAF	(–)-CSA	8→9
**a** = U	93	82	60	65	95	92	82
**b** = T	85	81	60	75	90	95	87
**c** = C	91	75	35	ND	-	-	70 *^a,b^*
**d** = C^Bz^	80	72	60	40	90	ND	-
**e** = A	85	90	60	77	95	ND	88
**f** = Hypoxanthine	80	70	ND	80	75	ND	80

*^a^* From **8d**. *^b^* See Scheme 4. ND, not the desired product. -, reaction not performed.

## Data Availability

Not applicable.

## Supplementary Materials

The following supporting information can be downloaded at: https://www.mdpi.com/article/10.3390/molecules27133993/s1, ^1^H and ^13^C-NMR data with their assignment for all compounds. Level of purity is indicated by the inclusion of copies of ^1^H, ^13^C, and DEPT NMR spectra; in addition, some 2D NMR experiments are shown, which were used to assign the peaks.
