# Supplementary material for: Sustainable Protocol for the Synthesis of 2′,3′-Dideoxynucleoside and 2′,3′-Didehydro-2′,3′-dideoxynucleoside Derivatives
Source: Molecules. 2022 Jun 21;27(13):3993. doi: 10.3390/molecules27133993 (PMC9268517; doi:10.3390/molecules27133993)

Sustainable Protocol for the Synthesis of 2',3'-Dideoxynucleoside and 2',3'-Didehydro-2',3'-dideoxynucleoside Derivatives

Virginia Martín-Nieves,[1] Yogesh S. Sanghvi,[2] Susana Fernández,[1,*] and Miguel Ferrero[1,*]

[1]*Departamento de Química Orgánica e Inorgánica, Universidad de Oviedo, Avenida Julián de Clavería 8, 33006-Oviedo (Asturias), Spain*
[2]*Rasayan Inc., 2802 Crystal Ridge Road, Encinitas, CA 92024-6615, USA*

S.F.: fernandezgsusana@uniovi.es; M.F.: mferrero@uniovi.es

SUPPORTING INFORMATION (page 1 of 162 pages)

Table of Contents

NMR DATA OF COMPOUNDS:

Compound **2a** – **2f** S3
Compound **5a** – **5f** S3-S4
Compound **6a** – **6f** S4-S5
Compound **7a** – **7f** S5-S6
Compound **8a** – **8b**, **8d – 8f** S6
Compound **9a** – **9c**, **9e – 9f** S6-S7

COPIES OF 1D AND 2D NMR SPECTRA OF COMPOUNDS:

Compound **2a** (^{1}H, ^{13}C, DEPT, COSY, HSQC NMR) S8-S12
Compound **2b** (^{1}H, ^{13}C, DEPT, COSY, HSQC NMR) S13-S17
Compound **2c** (^{1}H, ^{13}C, DEPT, COSY, HSQC NMR) S18-S22
Compound **2d** (^{1}H, ^{13}C, DEPT, COSY, HSQC, HMBC NMR) S23-S28
Compound **2e** (^{1}H, ^{13}C, DEPT, COSY, HSQC, HMBC NMR) S29-S34
Compound **2f** (^{1}H, ^{13}C, DEPT, COSY, HSQC, HMBC NMR) S35-S40

Compound **5a** (^{1}H, ^{13}C NMR) S41-S42
Compound **5b** (^{1}H, ^{13}C NMR) S43-S44
Compound **5c** (^{1}H, ^{13}C NMR) S45-S46
Compound **5d** (^{1}H, ^{13}C, DEPT, HSQC NMR) S47-S50
Compound **5e** (^{1}H, ^{13}C NMR) S51-S52
Compound **5f** (^{1}H, ^{13}C, DEPT, COSY, HSQC, HMBC NMR) S53-S58

Compound **6a** (^{1}H, ^{13}C, DEPT, COSY, HSQC NMR) S59-S63
Compound **6b** (^{1}H, ^{13}C, DEPT, COSY, HSQC NMR) S64-S68
Compound **6c** (^{1}H, ^{13}C, DEPT, COSY, HSQC NMR) S69-S73
Compound **6d** (^{1}H, ^{13}C, DEPT, COSY, HSQC NMR) S74-S78
Compound **6e** (^{1}H, ^{13}C, DEPT, COSY, HSQC, HMBC NMR) S79-S84
Compound **6f** (^{1}H, ^{13}C, DEPT, COSY, HSQC NMR) S85-S89

Compound **7a** (^{1}H, ^{13}C NMR) S90-S91
Compound **7b** (^{1}H, ^{13}C, HSQC NMR) S92-S94
Compound **7c** (^{1}H, ^{13}C, DEPT, COSY, HSQC, HMBC NMR) S95-S100
Compound **7d** (^{1}H, ^{13}C, DEPT, COSY, HSQC, HMBC NMR) S101-S106
Compound **7e** (^{1}H, ^{13}C, DEPT, COSY, HSQC NMR) S107-S111
Compound **7f** (^{1}H, ^{13}C, COSY, HSQC NMR) S112-S115

Compound **8a** (^{1}H, ^{13}C, DEPT, COSY, HSQC NMR) S116-S120
Compound **8b** (^{1}H, ^{13}C NMR) S121-S122
Compound **8d** (^{1}H, ^{13}C, DEPT, COSY, HSQC, HMBC NMR) S123-S128
Compound **8e** (^{1}H, ^{13}C NMR) S129-S130
Compound **8f** (^{1}H, ^{13}C, COSY, HSQC, HMBC NMR) S131-S135

Compound **9a** (^{1}H, ^{13}C, DEPT, COSY, HSQC NMR) S136-S140
Compound **9b** (^{1}H, ^{13}C, DEPT, COSY, HSQC NMR) S141-S145
Compound **9c** (^{1}H, ^{13}C, DEPT, COSY, HSQC, HMBC NMR) S146-S151
Compound **9e** (^{1}H, ^{13}C, DEPT, COSY, HSQC, HMBC NMR) S152-S157
Compound **9f** (^{1}H, ^{13}C, COSY, HSQC, HMBC NMR) S158-S162

5′-*O*-Levulinyl-β-D-uridine (**2a**). ^{1}H-NMR (300.13 MHz, $CDCl_3$): δ 2.18 (s, 3H, *Me*-Lev), 2.58 (t, 2H, CH_2-Lev, *J* = 6.2 Hz), 2.78 (t, 2H, CH_2-Lev, *J* = 6.0 Hz), 4.19 (m, 1H, $H_{3'}$), 4.27 (m, 2H, $H_{2'}$ + $H_{4'}$), 4.36 (m, 2H, $H_{5'}$), 5.78 (d, 1H, H_5, *J* = 8.1 Hz), 5.82 (d, 1H, $H_{1'}$, *J* = 3.6 Hz), 7.66 (d, 1H, H_6, *J* = 8.1 Hz) ppm. ^{13}C-NMR (75.5 MHz, $CDCl_3$): δ 28.0 (*C*H_2-Lev), 29.9 (*C*H_3-Lev), 38.0 (*C*H_2-Lev), 63.6 ($C_{5'}$), 70.3 ($C_{3'}$), 75.0 ($C_{2'}$), 82.3 ($C_{4'}$), 90.6 ($C_{1'}$), 102.7 (C_5), 140.5 (C_6), 151.4 (C_2), 164.2 (C_4), 172.8 (*C*O-Lev), 207.6 (*C*O-Lev) ppm.

5′-*O*-Levulinyl-β-D-5-methyluridine (**2b**). ^{1}H-NMR (300.13 MHz, $CDCl_3$): δ 1.88 (s, 3H, *Me*), 2.19 (s, 3H, *Me*-Lev), 2.59 (t, 2H, CH_2-Lev, *J* = 6.3), 2.80 (t, 2H, CH_2-Lev, *J* = 6.3), 4.19 (m, 1H, $H_{3'}$), 4.23-4.37 (several m, 3H, $H_{2'}$ + $H_{4'}$ + $H_{5'}$), 4.45 (dd, 1H, $H_{5'}$, *J* = 12.3, 3.7 Hz), 5.84 (d, 1H, $H_{1'}$, *J* = 3.7 Hz), 7.41 (s, 1H, H_6) ppm. ^{13}C-NMR (75.5 MHz, MeOH-*d*$_4$): δ 12.5 (*C*H_3), 28.9 (*C*H_2-Lev), 29.6 (*C*H_3-Lev), 38.6 (*C*H_2-Lev), 64.8 ($C_{5'}$), 71.3 ($C_{3'}$), 74.9 ($C_{2'}$), 83.1 ($C_{4'}$), 91.1 ($C_{1'}$), 111.8 (C_5), 138.0 (C_6), 152.5 (C_2), 166.3 (C_4), 174.2 (*C*O-Lev), 209.5 (*C*O-Lev) ppm.

5′-*O*-Levulinyl-β-D-cytidine (**2c**). ^{1}H-NMR (300.13 MHz, MeOH-*d*$_4$): δ 2.17 (s, 3H, *Me*-Lev), 2.59 (t, 2H, CH_2-Lev, *J* = 5.9 Hz), 2.84 (t, 2H, CH_2-Lev, *J* = 6.8 Hz), 4.08 (dd, 1H, $H_{3'}$, *J* = 6.3, 5.3 Hz), 4.16 (m, 2H, $H_{2'}$ + $H_{4'}$), 4.33 (dd, 1H, $H_{5'}$, *J* = 12.3, 4.1 Hz), 4.40 (dd, 1H, $H_{5'}$, *J* = 12.4, 2.9 Hz), 5.84 (d, 1H, H_5, *J* = 3.2 Hz), 5.95 (d, 1H, $H_{1'}$, *J* = 7.5 Hz), 7.81 (d, 1H, H_6, *J* = 7.5 Hz) ppm. ^{13}C-NMR (75.5 MHz, MeOH-*d*$_4$): δ 28.8 (*C*H_2-Lev), 29.6 (*C*H_3-Lev), 38.6 (*C*H_2-Lev), 64.5 ($C_{5'}$), 71.0 ($C_{3'}$), 75.9 ($C_{2'}$), 82.7 ($C_{4'}$), 92.6 ($C_{1'}$), 96.2 (C_5), 142.5 (C_6), 158.2 (C_2), 167.6 (C_4), 174.2 (*C*O-Lev), 209.5 (*C*O-Lev) ppm.

N^4-Benzoyl-5′-*O*-levulinyl-β-D-cytidine (**2d**). ^{1}H-NMR (300.13 MHz, DMSO-*d*$_6$): δ 2.12 (s, 3H, *Me*-Lev), 2.54 (t, 2H, CH_2-Lev, *J* = 6.3 Hz), 2.75 (t, 2H, CH_2-Lev, *J* = 6.3 Hz), 3.97 (m, 1H, $H_{4'}$), 4.09 (m, 2H, $H_{2'}$ + $H_{3'}$), 4.25 (dd, 1H, $H_{5'}$, *J* = 12.2, 5.1 Hz), 4.35 (dd, 1H, $H_{5'}$, *J* = 12.1, 2.3 Hz), 5.82 (d, 1H, $H_{1'}$, *J* = 2.7 Hz), 7.38 (d, 1H, H_5, *J* = 7.5 Hz), 7.51 (m, 2H, H_{arom}), 7.63 (m, 1H, H_{arom}), 8.01 (m, 2H, H_{arom}), 8.16 (d, 1H, H_6, *J* = 7.5 Hz) ppm. ^{13}C-NMR (75.5 MHz, DMSO-*d*$_6$): δ 28.0 (*C*H_2-Lev), 30.0 (*C*H_3-Lev), 37.9 (*C*H_2-Lev), 63.9 ($C_{5'}$), 69.7 ($C_{4'}$), 74.3 ($C_{2'}$), 81.3 ($C_{3'}$), 91.4 ($C_{1'}$), 96.9 (C_5), 128.9 (4CH_{arom}), 133.2, 133.6 (CH_{arom} + C_{arom}), 145.5 (C_6), 154.9 (C_2), 163.5 (C_4), 168.0 (*C*=O), 172.2 (*C*O-Lev), 207.4 (*C*O-Lev) ppm.

5′-*O*-Levulinyl-β-D-adenosine (**2e**). ^{1}H-NMR (300.13 MHz, MeOH-*d*$_4$): δ 2.14 (s, 3H, *Me*-Lev), 2.56 (t, 2H, CH_2-Lev, *J* = 6.4 Hz), 2.78 (m, 2H, CH_2-Lev, *J* = 6.4 Hz), 4.25 (q, 1H, $H_{4'}$, *J* = 4.7 Hz), 4.37 (m, 3H, $H_{3'}$ + $H_{5'}$), 4.74 (t, 2H, $H_{2'}$, *J* = 4.9 Hz), 6.03 (d, 1H, $H_{1'}$, *J* = 4.5 Hz), 8.21 (s, 1H, H_2), 8.31 (s, 1H, H_8) ppm. ^{13}C-NMR (75.5 MHz, MeOH-*d*$_4$): δ 28.8 (*C*H_2-Lev), 29.6 (*C*H_3-Lev), 38.6 (*C*H_2-Lev), 64.8 ($C_{5'}$), 71.8 ($C_{3'}$), 75.2 ($C_{2'}$), 83.5 ($C_{4'}$), 90.2 ($C_{1'}$), 120.5 (C_5, cross peak with H_8 in HMBC), 141.2 (C_8), 150.6 (C_4),153.9 (C_2), 157.3 (C_6), 174.2 (*C*O-Lev), 209.5 (*C*O-Lev) ppm.

5′-*O*-Levulinyl-β-D-inosine (**2f**). ^{1}H-NMR (300.13 MHz, MeOH-*d*$_4$): δ 2.15 (s, 3H, *Me*-Lev), 2.57 (t, 2H, CH_2-Lev, *J* = 6.4 Hz), 2.80 (t, 2H, CH_2-Lev, *J* = 6.4 Hz), 4.25 (q, 1H, $H_{4'}$, *J* = 4.3 Hz), 4.37 (m, 3H, $H_{3'}$ + $H_{5'}$), 4.70 (t, 2H, $H_{2'}$, *J* = 4.8 Hz), 6.03 (d, 1H, $H_{1'}$, *J* = 4.6 Hz), 8.07 (s, 1H, H_2), 8.27 (s, 1H, H_8) ppm. ^{13}C-NMR (75.5 MHz, MeOH-*d*$_4$): δ 28.8 (*C*H_2-Lev), 29.6 (*C*H_3-Lev), 38.6 (*C*H_2-Lev), 64.8 ($C_{5'}$), 71.7 ($C_{3'}$), 75.5 ($C_{2'}$), 83.7 ($C_{4'}$), 90.4 ($C_{1'}$), 125.9 (C_5), 140.7 (C_8), 146.8 (C_2), 149.9 (C_4, cross peak with $H_{1'}$ in HMBC), 158.9 (C_6), 174.2 (*C*O-Lev), 209.5 (*C*O-Lev) ppm.

5′-*O*-(*tert*-Butyldimethylsilyl)-β-d-uridine (**5a**). ^{1}H-NMR (300.13 MHz, $CDCl_3$): δ 0.10 (s, 6H, Si-*Me*), 0.91 (s, 9H, Si-^{t}*Bu*), 3.83 (dd, 1H, $H_{5'}$, *J* = 11.7, 1.5 Hz), 4.01 (dd, 1H, $H_{5'}$, *J* = 11.8, 1.8 Hz), 4.12 (m, 1H, $H_{4'}$), 4.23 (m, 2H, $H_{2'}$ + $H_{3'}$), 5.64 (d, 1H, H_5, *J* = 8.1 Hz), 5.90 (d, 1H, $H_{1'}$, *J* = 2.2 Hz), 8.05 (d, 1H, H_6, *J* = 8.1 Hz) ppm. ^{13}C-NMR (75.5 MHz, $CDCl_3$): δ -5.4 (Si-*C*H_3), 18.6 (SiCMe$_3$), 26.1 (^{t}Bu-*C*H_3), 62.0 ($C_{5'}$), 69.5 ($C_{2'}$), 75.8 ($C_{3'}$), 85.1 ($C_{4'}$), 90.3 ($C_{1'}$), 102.3 (C_5), 140.6 (C_6), 151.5 (C_2), 164.1 (C_4) ppm.

5′-*O*-(*tert*-Butyldimethylsilyl)-β-d-5-methyluridine (**5b**). ^{1}H-NMR (300.13 MHz, MeOH-*d*$_4$): δ 0.15 (s, 3H, Si-*Me*), 0.16 (s, 3H, Si-*Me*), 0.97 (s, 9H, Si-^{t}*Bu*), 1.88 (d, 3H, Me, *J* = 1.1 Hz), 3.85 (dd, 1H, $H_{5'}$, *J* = 11.6, 2.7 Hz), 3.94 (dd, 1H, $H_{5'}$, *J* = 11.6, 2.3 Hz), 4.02 (q, 1H, $H_{4'}$, *J* = 2.6 Hz), 4.13 (m, 2H, $H_{2'}$ + $H_{3'}$), 5.95 (d, 1H, $H_{1'}$, *J* = 5.0 Hz), 7.58 (d, 1H, H_6, *J* = 1.1 Hz) ppm. ^{13}C-NMR (75.5 MHz, MeOH-*d*$_4$): δ -5.2 (Si-*C*H_3), 12.6 (*C*H_3), 19.4 (SiCMe$_3$), 26.5 (^{t}Bu-CH$_3$), 64.3 ($C_{5'}$), 71.7 ($C_{2'}$), 75.7 ($C_{3'}$), 86.4 ($C_{4'}$), 89.6 ($C_{1'}$), 111.6 (C_5), 137.4 (C_6), 152.6 (C_2), 166.2 (C_4) ppm.

5′-*O*-(*tert*-Butyldimethylsilyl)-β-d-cytidine (**5c**). ^{1}H-NMR (300.13 MHz, MeOH-d_4): δ 0.15 (s, 6H, Si-*Me*), 0.96 (s, 9H, Si-^{t}Bu), 3.87 (dd, 1H, $H_{5'}$, J = 12.0, 2.1 Hz), 4.02-4.16 (several m, 4H, $H_{2'}$ + $H_{3'}$ + $H_{4'}$ + $H_{5'}$), 5.85 (d, 1H, H_5, J = 7.6 Hz), 5.88 (d, 1H, $H_{1'}$, J = 2.7 Hz), 8.11 (d, 1H, H_6, J = 7.5 Hz) ppm. ^{13}C-NMR (75.5 MHz, MeOH-d_4): δ -5.39 (Si-CH_3), -5.36 (Si-CH_3), 19.3 (SiCMe$_3$), 26.5 (^{t}Bu-CH_3), 63.0 ($C_{5'}$), 70.1 ($C_{2'}$), 76.8 ($C_{3'}$), 85.3 ($C_{4'}$), 91.8 ($C_{1'}$), 95.71 (C_5), 142.4 (C_6), 158.4 (C_2), 167.6 (C_4) ppm.

N^4-Benzoyl-5′-*O*-(*tert*-butyldimethylsilyl)-β-d-cytidine (**5d**). ^{1}H-NMR (300.13 MHz, MeOH-d_4): δ 0.17 (s, 3H, Si-*Me*), 0.19 (s, 3H, Si-*Me*), 0.99 (s, 9H, Si-^{t}Bu), 3.91 (dd, 1H, $H_{5'}$, J = 12.2, 2.1 Hz), 4.15 (several m, 4H, $H_{2'}$+ $H_{3'}$+ $H_{4'}$+ $H_{5'}$), 5.90 (d, 1H, $H_{1'}$, J = 1.5 Hz), 7.58 (m, 4H, H_5 + H_{arom}), 7.98 (m, 2H, H_{arom}), 8.64 (d, 1H, H_6, J = 7.5 Hz) ppm. ^{13}C-NMR (75.5 MHz, MeOH-d_4): δ -5.3 (Si-CH_3), 19.4 (SiCMe$_3$), 26.5 (^{t}Bu-CH_3), 62.6 ($C_{5'}$), 69.5 ($C_{2'}$), 76.8 ($C_{3'}$), 85.5 ($C_{4'}$), 92.9 ($C_{1'}$), 98.1 (C_5), 129.2 (CH_{arom}), 129.8 (CH_{arom}), 134.1 (CH_{arom}), 134.7 (C_{arom}), 146.3 (C_6), 157.9 (C_2), 164.8 (C_4), 169.2 (*C*=O) ppm.

5′-*O*-(*tert*-Butyldimethylsilyl)-β-d-adenosine (**5e**). ^{1}H-NMR (300.13 MHz, MeOH-d_4): δ 0.11 (s, 6H, Si-*Me*), 0.92 (s, 9H, Si-^{t}Bu), 3.87 (dd, 1H, $H_{5'}$, J = 11.6, 3.0 Hz), 4.01 (dd, 1H, $H_{5'}$, J = 11.6, 3.0 Hz), 4.13 (m, 1H, $H_{4'}$), 4.36 (t, 1H, $H_{3'}$, J = 5.0 Hz), 4.56 (t, 1H, $H_{2'}$, J = 4.6 Hz), 6.05 (d, 1H, $H_{1'}$, J = 4.2 Hz), 8.20 (s, 1H, H_2), 8.38 (s, 1H, H_8) ppm. ^{13}C-NMR (75.5 MHz, MeOH-d_4): δ -5.33 (Si-CH_3), -5.31 (Si-CH_3), 19.3 (SiCMe$_3$), 26.5 (^{t}Bu-CH_3), 63.8 ($C_{5'}$), 71.3 ($C_{3'}$), 76.3 ($C_{2'}$), 86.3 ($C_{4'}$), 90.1 ($C_{1'}$), 120.4 (C_5), 140.7 (C_8), 150.5 (C_4), 153.9 (C_2), 157.3 (C_6) ppm.

5′-*O*-(*tert*-Butyldimethylsilyl)-β-d-inosine (**5f**). ^{1}H-NMR (300.13 MHz, MeOH-d_4): δ 0.10 (s, 6H, Si-*Me*), 0.91 (s, 9H, Si-^{t}Bu), 3.86 (dd, 1H, $H_{5'}$, J = 11.6, 3.1 Hz), 3.99 (dd, 1H, $H_{5'}$, J = 11.6, 3.0 Hz), 4.14 (m, 1H, $H_{4'}$), 4.36 (t, 1H, $H_{3'}$, J = 4.8 Hz), 4.56 (t, 1H, $H_{2'}$, J = 4.8 Hz), 6.07 (d, 1H, $H_{1'}$, J = 4.5 Hz), 8.07 (s, 1H, H_2), 8.31 (s, 1H, H_8) ppm. ^{13}C-NMR (75.5 MHz, MeOH-d_4): δ -5.33 (Si-CH_3), -5.29 (Si-CH_3), 19.3 (SiCMe$_3$), 26.5 (^{t}Bu-CH_3), 63.9 ($C_{5'}$), 71.5 ($C_{3'}$), 76.6 ($C_{2'}$), 86.6 ($C_{4'}$), 90.0 ($C_{1'}$), 125.6 (C_5), 140.1 (C_8), 146.8 (C_2), 149.9 (C_4), 158.9 (C_6) ppm.

5′-*O*-(*tert*-Butyldimethylsilyl)-2′,3′-bis-*O*-[(ethylthio)thiocarbonyl]-β-d-uridine (**6a**). ^{1}H-NMR (300.13 MHz, $CDCl_3$): δ 0.15 (s, 3H, Si-*Me*), 0.17 (s, 3H, Si-*Me*), 0.95 (s, 9H, Si-^{t}Bu), 1.35 (t, 3H, SCH_2-*Me*, J = 7.4 Hz), 1.37 (t, 3H, SCH_2-*Me*, J = 7.4 Hz), 3.13 (m, 4H, S-CH_2), 3.92 (dd, 1H, $H_{5'}$, J = 11.4, 1.7 Hz), 4.03 (dd, 1H, $H_{5'}$, J = 11.4, 1.5 Hz), 4.44 (m, 1H, $H_{4'}$), 5.75 (d, 1H, H_5, J = 8.2 Hz), 6.07 (dd, 1H, $H_{2'}$, J = 7.4, 5.7 Hz), 6.22 (dd, 1H, $H_{3'}$, J = 5.6, 1.1 Hz), 6.56 (d, 1H, $H_{1'}$, J = 7.4 Hz), 7.87 (d, 1H, H_6, J = 8.2 Hz), 9.09 (br s, 1H, NH) ppm. ^{13}C-NMR (75.5 MHz, $CDCl_3$): δ -5.5 (Si-CH_3), -5.2 (Si-CH_3), 13.3 (SCH_2-CH_3), 13.5 (SCH_2-CH_3), 18.5 (SiCMe$_3$), 26.1 (^{t}Bu-CH_3), 30.8 (S-CH_2), 63.5 ($C_{5'}$), 78.9 ($C_{3'}$), 79.6 ($C_{2'}$), 84.6 ($C_{4'}$), 85.4 ($C_{1'}$), 103.6 (C_5), 139.7 (C_6), 150.4 (C_2), 163.0 (C_4), 213.7 (*C*=S), 214.1 (*C*=S) ppm.

5′-*O*-(*tert*-Butyldimethylsilyl)-2′,3′-bis-*O*-[(ethylthio)thiocarbonyl]-β-d-5-methyluridine (**6b**). ^{1}H-NMR (300.13 MHz, $CDCl_3$): δ 0.19 (s, 6H, Si-*Me*), 0.98 (s, 9H, Si-^{t}Bu), 1.35 (t, 3H, SCH_2-*Me*, J = 7.4 Hz), 1.37 (t, 3H, SCH_2-*Me*, J = 7.4 Hz), 1.94 (d, 3H, Me, J = 1.1 Hz), 3.14 (m, 4H, S-CH_2), 3.93 (dd, 1H, $H_{5'}$, J = 11.4, 1.7 Hz), 4.07 (dd, 1H, $H_{5'}$, J = 11.4, 1.7 Hz), 4.42 (m, 1H, $H_{4'}$), 6.03 (dd, 1H, $H_{2'}$, J = 7.8, 5.6 Hz), 6.22 (dd, 1H, $H_{3'}$, J = 5.6, 1.0 Hz), 6.56 (d, 1H, $H_{1'}$, J = 7.9 Hz), 7.54 (d, 1H, H_6, J = 1.2 Hz), 8.16 (br s, 1H, NH) ppm. ^{13}C-NMR (75.5 MHz, $CDCl_3$): δ -5.3 (Si-CH_3), -4.9 (Si-CH_3), 12.6 (CH_3), 13.4 (SCH_2-CH_3), 13.5 (SCH_2-CH_3), 18.6 (SiCMe$_3$), 26.2 (^{t}Bu-CH_3), 30.79 (S-CH_2), 30.83 (S-CH_2), 63.5 ($C_{5'}$), 78.7 ($C_{3'}$), 79.1 ($C_{2'}$), 84.4 ($C_{4'}$), 84.7 ($C_{1'}$), 112.2 (C_5), 134.9 (C_6), 150.5 (C_2), 163.5 (C_4), 213.7 (*C*=S), 214.2 (*C*=S) ppm.

5′-*O*-(*tert*-Butyldimethylsilyl)-2′,3′-bis-*O*-[(ethylthio)thiocarbonyl]-β-d-cytidine (**6c**). ^{1}H-NMR (300.13 MHz, $CDCl_3$): δ 0.14 (s, 3H, Si-*Me*), 0.15 (s, 3H, Si-*Me*), 0.94 (s, 9H, Si-^{t}Bu), 1.33 (t, 3H, SCH_2-*Me*, J = 6.1 Hz), 1.35 (t, 3H, SCH_2-*Me*, J = 6.1 Hz), 3.13 (m, 4H, S-CH_2), 3.92 (dd, 1H, $H_{5'}$, J = 11.3, 1.8 Hz), 4.01 (dd, 1H, $H_{5'}$, J = 11.3, 1.7 Hz), 4.41 (m, 1H, $H_{4'}$), 5.76 (d, 1H, H_5, J = 7.4 Hz), 6.04 (dd, 1H, $H_{2'}$, J = 7.0, 5.6 Hz), 6.24 (dd, 1H, $H_{3'}$, J = 5.5, 2.0 Hz), 6.67 (d, 1H, $H_{1'}$, J = 7.0 Hz), 7.84 (d, 1H, H_6, J = 7.4 Hz) ppm. ^{13}C-NMR (75.5 MHz, $CDCl_3$): δ -5.4 (Si-CH_3), -5.2 (Si-CH_3), 13.4 (SCH_2-CH_3), 13.6 (SCH_2-CH_3), 18.5 (SiCMe$_3$), 26.2 (^{t}Bu-CH_3), 30.66 (S-CH_2), 30.71 (S-CH_2), 63.4 ($C_{5'}$), 78.8 ($C_{3'}$), 80.4 ($C_{2'}$), 83.9 ($C_{4'}$), 86.1 ($C_{1'}$), 95.6 (C_5), 141.3 (C_6), 155.8 (C_2), 165.7 (C_4), 213.7 (*C*=S), 214.1 (*C*=S) ppm.

N^4-Benzoyl-5′-*O*-(*tert*-butyldimethylsilyl)-2′,3′-bis-*O*-[(ethylthio)thiocarbonyl]-β-d-cytidine (**6d**). ^{1}H-NMR (300.13 MHz, $CDCl_3$): δ 0.18 (s, 6H, Si-*Me*), 0.97 (s, 9H, Si-^{t}Bu), 1.34 (t, 3H, SCH_2-*Me*, J = 6.1 Hz), 1.37 (t, 3H,

SCH_2-*Me*, *J* = 6.1 Hz), 3.14 (m, 4H, S-C*H*$_2$), 3.98 (dd, 1H, $H_{5'}$, *J* = 11.5, 1.8 Hz), 4.05 (dd, 1H, $H_{5'}$, *J* = 11.5, 1.6 Hz), 4.49 (m, 1H, $H_{4'}$), 6.15 (dd, 1H, $H_{2'}$, *J* = 6.8, 5.6 Hz), 6.27 (dd, 1H, $H_{3'}$, *J* = 5.5, 1.8 Hz), 6.74 (d, 1H, $H_{1'}$, *J* = 6.9 Hz), 7.50 (m, 3H, H_5 + H_{arom}), 7.61 (m, 1H, H_{arom}), 7.91 (m, 2H, H_{arom}), 8.30 (d, 1H, H_6, *J* = 7.6 Hz), 8.69 (br s, 1H, NH) ppm. ^{13}C-NMR (75.5 MHz, $CDCl_3$): δ -5.4 (Si-CH_3), -5.2 (Si-CH_3), 13.3 (SCH_2-CH_3), 13.5 (SCH_2-CH_3), 18.5 ($SiCMe_3$), 26.1 (^{t}Bu-CH_3), 30.65 (S-CH_2), 30.74 (S-CH_2), 63.2 ($C_{5'}$), 78.8 ($C_{3'}$), 80.7 ($C_{2'}$), 84.6 ($C_{4'}$), 86.5 ($C_{1'}$), 97.8 (C_5), 127.8 (CH_{arom}), 129.1 (CH_{arom}), 133.2 (C_{arom}), 133.3 (CH_{arom}), 144.4 (C_6), 154.6 (C_2), 162.5 (C_4), 167.1 (*C*=O), 213.6 (*C*=S), 214.0 (*C*=S) ppm.

5′-*O*-(*tert*-Butyldimethylsilyl)-2′,3′-bis-*O*-[(ethylthio)thiocarbonyl]-β-d-adenosine (**6e**). ^{1}H-NMR (300.13 MHz, $CDCl_3$): δ 0.16 (s, 6H, Si-*Me*), 0.95 (s, 9H, Si-^{t}*Bu*), 1.29 (t, 3H, SCH_2-*Me*, *J* = 7.4 Hz), 1.37 (t, 3H, SCH_2-*Me*, *J* = 7.4 Hz), 3.07 (q, 2H, S-C*H*$_2$, *J* = 7.4 Hz), 3.17 (q, 2H, S-C*H*$_2$, *J* = 7.3 Hz), 3.95 (dd, 1H, $H_{5'}$, *J* = 11.4, 2.1 Hz), 4.05 (dd, 1H, $H_{5'}$, *J* = 11.3, 2.1 Hz), 4.52 (m, 1H, $H_{4'}$), 6.14 (br s, 2H, NH_2), 6.41 (dd, 1H, $H_{3'}$, *J* = 5.4, 1.9 Hz), 6.48 (dd, 1H, $H_{2'}$, *J* = 6.8, 5.5 Hz), 6.60 (d, 1H, $H_{1'}$, *J* = 6.9 Hz), 8.22 (s, 1H, H_8), 8.35 (s, 1H, H_2) ppm. ^{13}C-NMR (75.5 MHz, $CDCl_3$): δ -5.3 (Si-CH_3), -5.1 (Si-CH_3), 13.3 (SCH_2-CH_3), 18.6 ($SiCMe_3$), 26.2 (^{t}Bu-CH_3), 30.8 (S-CH_2), 63.4 ($C_{5'}$), 78.8 ($C_{3'}$), 80.7 ($C_{2'}$), 84.7 ($C_{1'}$ + $C_{4'}$), 119.6 (C_5), 138.5 (C_8), 150.3 (C_4), 153.4 (C_2), 155.7 (C_6), 213.6 (*C*=S), 214.0 (*C*=S) ppm.

5′-*O*-(*tert*-Butyldimethylsilyl)-2′,3′-bis-*O*-[(ethylthio)thiocarbonyl]-β-d-inosine (**6f**). ^{1}H-NMR (300.13 MHz, $CDCl_3$): δ 0.17 (s, 6H, Si-*Me*), 0.96 (s, 9H, Si-^{t}*Bu*), 1.31 (t, 3H, SCH_2-*Me*, *J* = 7.4 Hz), 1.39 (t, 3H, SCH_2-*Me*, *J* = 7.4 Hz), 3.09 (q, 2H, S-C*H*$_2$, *J* = 7.4 Hz), 3.18 (q, 2H, S-C*H*$_2$, *J* = 7.4 Hz), 3.97 (dd, 1H, $H_{5'}$, *J* = 11.4, 2.0 Hz), 4.06 (dd, 1H, $H_{5'}$, *J* = 11.4, 2.0 Hz), 4.55 (m, 1H, $H_{4'}$), 6.40 (dd, 1H, $H_{3'}$, *J* = 5.3, 2.0 Hz), 6.46 (m, 1H, $H_{2'}$), 6.53 (d, 1H, $H_{1'}$, *J* = 6.7 Hz), 8.20 (s, 1H, H_2), 8.24 (s, 1H, H_8), 13.10 (br s, 1H, NH) ppm. ^{13}C-NMR (75.5 MHz, $CDCl_3$): δ -5.3 (Si-CH_3), -5.1 (Si-CH_3), 13.3 (SCH_2-CH_3), 13.4 (SCH_2-CH_3), 18.6 ($SiCMe_3$), 26.2 (^{t}Bu-CH_3), 30.86 (S-CH_2), 30.93 (S-CH_2), 63.3 ($C_{5'}$), 78.7 ($C_{3'}$), 80.9 ($C_{2'}$), 84.9 ($C_{4'}$), 85.2 ($C_{1'}$), 124.9 (C_5), 138.3 (C_8), 145.6 (C_2), 149.3 (C_4), 159.3 (C_6), 213.4 (*C*=S), 214.0 (*C*=S) ppm.

5′-*O*-(*tert*-Butyldimethylsilyl)-2′,3′-didehydro-2′,3′-dideoxy-β-d-uridine (**7a**). ^{1}H-NMR (300.13 MHz, DMSO-*d*$_6$): δ 0.03 (s, 6H, Si-*Me*), 0.85 (s, 9H, Si-^{t}*Bu*), 3.75 (dd, 1H, $H_{5'}$, *J* = 11.7, 3.7 Hz), 3.81 (dd, 1H, $H_{5'}$, *J* = 11.7, 3.0 Hz), 4.82 (m, 1H, $H_{4'}$), 5.52 (d, 1H, H_5, *J* = 8.0 Hz), 5.95 (dt, 1H, $H_{2'}$, *J* = 6.0, 1.8 Hz), 6.39 (dt, 1H, $H_{3'}$, *J* = 6.0, 1.6 Hz), 6.80 (m, 1H, $H_{1'}$), 7.63 (d, 1H, H_6, *J* = 8.0 Hz), 11.35 (br s, 1H, NH) ppm. ^{13}C-NMR (75.5 MHz, DMSO-*d*$_6$): δ -5.43 (Si-CH_3), -5.38 (Si-CH_3), 18.2 ($SiCMe_3$), 25.8 (^{t}Bu-CH_3), 64.3 ($C_{5'}$), 86.9 ($C_{4'}$), 89.1 ($C_{1'}$), 101.6 (C_5), 126.2 ($C_{2'}$), 134.5 ($C_{3'}$), 140.8 (C_6), 150.7 (C_2), 163.1 (C_4) ppm.

5′-*O*-(*tert*-Butyldimethylsilyl)-2′,3′-didehydro-3′-deoxy-β-d-5-thymidine (**7b**). ^{1}H-NMR (300.13 MHz, DMSO-*d*$_6$): δ 0.03 (s, 6H, Si-*Me*), 0.85 (s, 9H, Si-^{t}*Bu*), 1.75 (d, 3H, Me, *J* = 0.9 Hz), 3.72 (dd, 1H, $H_{5'}$, *J* = 11.5, 4.6 Hz), 3.80 (dd, 1H, $H_{5'}$, *J* = 11.6, 3.2 Hz), 4.78 (m, 1H, $H_{4'}$), 5.96 (dt, 1H, $H_{2'}$, *J* = 6.0, 2.0 Hz), 6.38 (dt, 1H, $H_{3'}$, *J* = 6.0, 1.6 Hz), 6.78 (m, 1H, $H_{1'}$), 7.28 (d, 1H, H_6, *J* = 1.2 Hz), 11.36 (br s, 1H, NH) ppm. ^{13}C-NMR (75.5 MHz, DMSO-*d*$_6$): δ -5.3 (Si-CH_3), -5.2 (Si-CH_3), 12.2 (CH_3), 18.3 ($SiCMe_3$), 25.9 (^{t}Bu-CH_3), 64.7 ($C_{5'}$), 86.7 ($C_{4'}$), 89.2 ($C_{1'}$), 109.4 (C_5), 126.2 ($C_{2'}$), 134.4 ($C_{3'}$), 135.9 (C_6), 150.8 (C_2), 163.8 (C_4) ppm.

5′-*O*-(*tert*-Butyldimethylsilyl)-2′,3′-didehydro-2′,3′-dideoxy-β-d-cytidine (**7c**). ^{1}H-NMR (300.13 MHz, $CDCl_3$): δ 0.066 (s, 3H, Si-*Me*), 0.070 (s, 3H, Si-*Me*), 0.90 (s, 9H, Si-^{t}*Bu*), 3.83 (dd, 1H, $H_{5'}$, *J* = 11.6, 2.8 Hz), 3.92 (dd, 1H, $H_{5'}$, *J* = 11.5, 3.0 Hz), 4.90 (m, 1H, $H_{4'}$), 5.62 (d, 1H, H_5, *J* = 7.4 Hz), 5.91 (m, 1H, $H_{2'}$), 6.14 (dt, 1H, $H_{3'}$, *J* = 6.0, 1.6 Hz), 7.07 (m, 1H, $H_{1'}$), 7.94 (d, 1H, H_6, *J* = 7.4 Hz) ppm. ^{13}C-NMR (75.5 MHz, $CDCl_3$): δ -5.3 (Si-CH_3), -5.2 (Si-CH_3), 18.6 ($SiCMe_3$), 26.1 (^{t}Bu-CH_3), 64.5 ($C_{5'}$), 87.3 ($C_{4'}$), 91.0 ($C_{1'}$), 94.0 (C_5), 128.2 ($C_{2'}$), 132.7 ($C_{3'}$), 142.8 (C_6), 156.3 (C_2), 165.6 (C_4) ppm.

N^4-Benzoyl-5′-*O*-(*tert*-butyldimethylsilyl)-2′,3′-didehydro-2′,3′-dideoxy-β-d-cytidine (**7d**). ^{1}H-NMR (300.13 MHz, $CDCl_3$): δ 0.08 (s, 3H, Si-*Me*), 0.10 (s, 3H, Si-*Me*), 0.90 (s, 9H, Si-^{t}*Bu*), 3.86 (dd, 1H, $H_{5'}$, *J* = 11.7, 2.6 Hz), 3.99 (dd, 1H, $H_{5'}$, *J* = 11.7, 2.7 Hz), 4.99 (m, 1H, $H_{4'}$), 6.02 (d, 1H, $H_{2'}$, *J* = 5.7 Hz), 6.18 (dt, 1H, $H_{3'}$, *J* = 6.0, 1.4 Hz), 7.03 (m, 1H, $H_{1'}$), 7.49 (m, 3H, H_5 + H_{arom}), 7.60 (m, 1H, H_{arom}), 7.93 (d, 2H, H_{arom}, *J* = 7.3 Hz), 8.40 (d, 1H, H_6, *J* = 7.5 Hz), 8.71 (br s, 1H, NH) ppm. ^{13}C-NMR (75.5 MHz, $CDCl_3$): δ -5.4 (Si-CH_3), -5.2 (Si-CH_3), 18.6

(SiCMe$_3$), 26.0 (^{t}Bu-CH$_3$), 64.2 (C$_{5'}$), 88.0 (C$_{4'}$), 91.8 (C$_{1'}$), 96.9 (C$_5$), 127.67 (C$_{2'}$), 127.71 (CH$_{arom}$), 129.1 (CH$_{arom}$), 133.0 (C$_{3'}$), 133.2 (CH$_{arom}$), 133.4 (C$_{arom}$), 145.7 (C$_6$), 155.1 (C$_2$), 162.3 (C$_4$), 167.3 (*C*=O) ppm.

5′-*O*-(*tert*-Butyldimethylsilyl)-2′,3′-didehydro-2′,3′-dideoxy-β-d-adenosine (**7e**). ^{1}H-NMR (300.13 MHz, CDCl$_3$): δ 0.03 (s, 3H, Si-*Me*), 0.04 (s, 3H, Si-*Me*), 0.87 (s, 9H, Si-*^{t}Bu*), 3.81 (d, 2H, H$_{5'}$, *J* = 3.8 Hz), 4.97 (m, 1H, H$_{4'}$), 5.95 (br s, 2H, NH$_2$), 6.03 (dt, 1H, H$_{2'}$, *J* = 6.0, 1.9 Hz), 6.38 (dt, 1H, H$_{3'}$, *J* = 6.0, 1.6 Hz), 7.09 (m, 1H, H$_{1'}$), 8.09 (s, 1H, H$_8$), 8.34 (s, 1H, H$_2$) ppm. ^{13}C-NMR (75.5 MHz, CDCl$_3$): δ -5.3 (Si-*C*H$_3$), -5.2 (Si-*C*H$_3$), 18.7 (SiCMe$_3$), 26.1 (^{t}Bu-CH$_3$), 64.9 (C$_{5'}$), 87.9 (C$_{4'}$), 88.4 (C$_{1'}$), 119.2 (C$_5$), 125.7 (C$_{2'}$), 134.7 (C$_{3'}$), 139.4 (C$_8$), 149.7 (C$_4$), 152.9 (C$_2$), 155.7 (C$_6$) ppm.

5′-*O*-(*tert*-Butyldimethylsilyl)-2′,3′-didehydro-2′,3′-dideoxy-β-d-inosine (**7f**). ^{1}H-NMR (300.13 MHz, CDCl$_3$): δ 0.03 (s, 3H, Si-*Me*), 0.04 (s, 3H, Si-*Me*), 0.87 (s, 9H, Si-*^{t}Bu*), 3.82 (m, 2H, H$_{5'}$), 4.99 (m, 1H, H$_{4'}$), 6.05 (dt, 1H, H$_{2'}$, *J* = 5.9, 1.8 Hz), 6.42 (dt, 1H, H$_{3'}$, *J* = 5.8, 1.7 Hz), 7.04 (m, 1H, H$_{1'}$), 8.09 (s, 1H, H$_8$), 8.23 (s, 1H, H$_2$), 13.23 (br s, 1H, NH) ppm. ^{13}C-NMR (75.5 MHz, CDCl$_3$): δ -5.3 (Si-CH$_3$), -5.2 (Si-CH$_3$), 18.7 (SiCMe$_3$), 26.1 (^{t}Bu-*C*H$_3$), 64.9 (C$_{5'}$), 88.1 (C$_{4'}$), 88.6 (C$_{1'}$), 124.7 (C$_5$), 125.3 (C$_{2'}$), 135.0 (C$_{3'}$), 139.1 (C$_8$), 145.3 (C$_2$), 149.0 (C$_4$), 159.5 (C$_6$) ppm.

2′,3′-Didehydro-2′,3′-dideoxy-β-d-uridine (**8a**). ^{1}H-NMR (300.13 MHz, MeOH-*d*$_4$): δ 3.73 (m, 2H, H$_{5'}$), 4.87 (m, 1H, H$_{4'}$), 5.65 (d, 1H, H$_5$, *J* = 8.1 Hz), 5.92 (m, 1H, H$_{2'}$), 6.41 (dt, 1H, H$_{3'}$, *J* = 6.0, 1.7 Hz), 6.94 (dt, 1H, H$_{1'}$, *J* = 3.1, 1.5 Hz), 7.89 (d, 1H, H$_6$, *J* = 8.1 Hz) ppm. ^{13}C-NMR (75.5 MHz, MeOH-*d*$_4$): δ 63.8 (C$_{5'}$), 89.1 (C$_{4'}$), 91.3 (C$_{1'}$), 102.5 (C$_5$), 127.0 (C$_{2'}$), 136.1 (C$_{3'}$), 143.1 (C$_6$), 152.7 (C$_2$), 166.3 (C$_4$) ppm.

2′,3′-Didehydro-3′-deoxy-β-d-5-thymidine (**8b**). ^{1}H-NMR (300.13 MHz, MeOH-*d*$_4$): δ 1.84 (d, 3H, Me, *J* = 1.1 Hz), 3.76 (m, 2H, H$_{5'}$), 4.86 (debajo de H$_2$O, 1H, H$_{4'}$), 5.90 (dt, 1H, H$_{2'}$, *J* = 6.0, 2.0 Hz), 6.40 (dt, 1H, H$_{3'}$, *J* = 6.0, 1.7 Hz), 6.95 (dt, 1H, H$_{1'}$, *J* = 3.4, 1.7 Hz), 7.75 (d, 1H, H$_6$, *J* = 1.2 Hz) ppm. ^{13}C-NMR (75.5 MHz, MeOH-*d*$_4$): δ 12.4 (*C*H$_3$), 63.8 (C$_{5'}$), 89.0 (C$_{4'}$), 91.0 (C$_{1'}$), 111.2 (C$_5$), 127.3 (C$_{2'}$), 135.9 (C$_{3'}$), 138.9 (C$_6$), 152.9 (C$_2$), 166.6 (C$_4$) ppm.

N^4-Benzoyl-2′,3′-didehydro-2′,3′-dideoxy-β-d-cytidine (**8d**). ^{1}H-NMR (300.13 MHz, MeOH-*d*$_4$): δ 3.78 (m, 2H, H$_{5'}$), 4.99 (m, 1H, H$_{4'}$), 6.05 (m, 1H, H$_{2'}$), 6.41 (dt, 1H, H$_{3'}$, *J* = 6.0, 1.6 Hz), 7.00 (dt, 1H, H$_{1'}$, *J* = 2.9, 1.4 Hz), 7.54 (m, 3H, H$_5$ + H$_{arom}$), 7.64 (m, 1H, H$_{arom}$), 7.97 (m, 2H, H$_{arom}$), 8.46 (d, 1H, H$_6$, *J* = 7.5 Hz) ppm. ^{13}C-NMR (75.5 MHz, MeOH-*d*$_4$): δ 63.7 (C$_{5'}$), 89.9 (C$_{4'}$), 93.4 (C$_{1'}$), 98.5 (C$_5$), 127.6 (C$_{2'}$), 129.2 (CH$_{arom}$), 129.8 (CH$_{arom}$), 134.1 (CH$_{arom}$), 134.7 (C$_{arom}$), 135.5 (C$_{3'}$), 147.3 (C$_6$), 158.3 (C$_2$), 165.0 (C$_4$), 169.1 (*C*=O) ppm.

2′,3′-Didehydro-2′,3′-dideoxy-β-d-adenosine (**8e**). ^{1}H-NMR (300.13 MHz, MeOH-*d*$_4$): δ 3.75 (m, 2H, H$_{5'}$), 5.01 (m, 1H, H$_{4'}$), 6.13 (m, 1H, H$_{2'}$), 6.48 (dt, 1H, H$_{3'}$, *J* = 6.0, 1.7 Hz), 7.04 (dt, 1H, H$_{1'}$, *J* = 3.2, 1.7 Hz), 8.20 (s, 1H, H$_2$), 8.28 (s, 1H, H$_8$) ppm. ^{13}C-NMR (75.5 MHz, MeOH-*d*$_4$): δ 64.2 (C$_{5'}$), 89.8 (C$_{1'}$), 90.3 (C$_{4'}$), 120.2 (C$_5$), 126.8 (C$_{2'}$), 135.7 (C$_{3'}$), 141.3 (C$_8$), 150.3 (C$_4$), 153.8 (C$_2$), 157.4 (C$_6$) ppm.

2′,3′-Didehydro-2′,3′-dideoxy-β-d-inosine (**8f**). ^{1}H-NMR (300.13 MHz, DMSO-*d*$_6$): δ 3.56 (m, 2H, H$_{5'}$), 4.89 (m, 1H, H$_{4'}$), 4.98 (t, OH$_{5'}$, *J* = 5.4 Hz), 6.13 (m, 1H, H$_{2'}$), 6.48 (dd, 1H, H$_{3'}$, *J* = 6.0, 1.5 Hz), 6.90 (m, 1H, H$_{1'}$), 8.08 (s, 1H, H$_2$), 8.11 (s, 1H, H$_8$), 12.39 (br s, 1H, NH) ppm. ^{13}C-NMR (75.5 MHz, DMSO-*d*$_6$): δ 62.7 (C$_{5'}$), 88.1 (C$_{1'}$), 88.3 (C$_{4'}$), 124.1 (C$_5$), 125.3 (C$_{2'}$), 134.8 (C$_{3'}$), 138.6 (C$_8$), 146.0 (C$_2$), 148.1 (C$_4$), 156.7 (C$_6$) ppm.

2′,3′-Dideoxy-β-d-uridine (**9a**). ^{1}H-NMR (300.13 MHz, MeOH-*d*$_4$): δ 1.96 (m, 2H, H$_{3'}$), 2.08 (m, 1H, H$_{2'}$), 2.40 (m, 1H, H$_{2'}$), 3.68 (dd, 1H, H$_{5'}$, *J* = 12.2, 4.0 Hz), 3.86 (dd, 1H, H$_{5'}$, *J* = 12.2, 3.1 Hz), 4.14 (m, 1H, H$_{4'}$), 5.66 (d, 1H, H$_5$, *J* = 8.1 Hz), 6.04 (dd, 1H, H$_{1'}$, *J* = 6.8, 3.4 Hz), 8.08 (d, 1H, H$_6$, *J* = 8.1 Hz) ppm. ^{13}C-NMR (75.5 MHz, MeOH-*d*$_4$): δ 25.9 (C$_{3'}$), 33.5 (C$_{2'}$), 63.7 (C$_{5'}$), 83.5 (C$_{4'}$), 87.7 (C$_{1'}$), 101.9 (C$_5$), 142.6 (C$_6$), 152.2 (C$_2$), 166.4 (C$_4$) ppm.

3′-Deoxy-β-d-5-thymidine (**9b**). ^{1}H-NMR (300.13 MHz, MeOH-*d*$_4$): δ 1.88 (d, 3H, Me, *J* = 1.1 Hz), 1.98 (m, 2H, H$_{3'}$), 2.07 (m, 1H, H$_{2'}$), 2.38 (m, 1H, H$_{2'}$), 3.68 (dd, 1H, H$_{5'}$, *J* = 12.2, 3.8 Hz), 3.88 (dd, 1H, H$_{5'}$, *J* = 12.2, 2.9

Hz), 4.13 (m, 1H, $H_{4'}$), 6.05 (dd, 1H, $H_{1'}$, J = 6.8, 3.5 Hz), 7.92 (d, 1H, H_6, J = 1.1 Hz) ppm. ^{13}C-NMR (75.5 MHz, MeOH-d_4): δ 12.4 (CH_3), 25.9 ($C_{3'}$), 33.3 ($C_{2'}$), 63.7 ($C_{5'}$), 83.2 ($C_{4'}$), 87.3 ($C_{1'}$), 110.8 (C_5), 138.4 (C_6), 152.4 (C_2), 166.6 (C_4) ppm.

2′,3′-Dideoxy-β-d-cytidine (**9c**). ^{1}H-NMR (300.13 MHz, D_2O): δ 1.78 (m, 1H, $H_{3'}$), 2.05 (m, 2H, $H_{2'}$ + $H_{3'}$), 2.45 (m, 1H, $H_{2'}$), 3.72 (dd, 1H, $H_{5'}$, J = 12.4, 5.4 Hz), 3.87 (dd, 1H, $H_{5'}$, J = 12.4, 3.1 Hz), 4.23 (m, 1H, $H_{4'}$), 6.00 (d, 1H, H_5, J = 7.5 Hz), 6.05 (dd, 1H, $H_{1'}$, J = 7.0, 2.7 Hz), 7.89 (d, 1H, H_6, J = 7.5 Hz) ppm. ^{13}C-NMR (75.5 MHz, D_2O): δ 24.7 ($C_{3'}$), 31.9 ($C_{2'}$), 62.6 ($C_{5'}$), 82.2 ($C_{4'}$), 87.0 ($C_{1'}$), 95.5 (C_5), 141.6 (C_6), 157.3 (C_2), 165.9 (C_4) ppm.

2′,3′-Dideoxy-β-d-adenosine (**9e**). ^{1}H-NMR (300.13 MHz, MeOH-d_4): δ 2.15 (m, 2H, $H_{3'}$), 2.51 (m, 2H, $H_{2'}$), 3.67 (dd, 1H, $H_{5'}$, J = 12.2, 3.9 Hz), 3.87 (dd, 1H, $H_{5'}$, J = 12.2, 3.0 Hz), 4.27 (m, 1H, $H_{4'}$), 6.28 (dd, 1H, $H_{1'}$, J = 5.8, 4.8 Hz), 8.18 (s, 1H, H_2), 8.41 (s, 1H, H_8) ppm. ^{13}C-NMR (75.5 MHz, MeOH-d_4): δ 26.5 ($C_{3'}$), 33.7 ($C_{2'}$), 64.5 ($C_{5'}$), 83.6 ($C_{4'}$), 87.4 ($C_{1'}$), 120.5 (C_5), 141.0 (C_2), 149.8 (C_4), 153.5 (C_8), 157.3 (C_6) ppm.

2′,3′-Dideoxy-β-d-inosine (**9f**). ^{1}H-NMR (300.13 MHz, MeOH-d_4): δ 2.12 (m, 2H, $H_{3'}$), 2.52 (m, 2H, $H_{2'}$), 3.67 (dd, 1H, $H_{5'}$, J = 12.1, 4.2 Hz), 3.84 (dd, 1H, $H_{5'}$, J = 12.1, 3.2 Hz), 4.26 (m, 1H, $H_{4'}$), 6.31 (dd, 1H, $H_{1'}$, J = 6.5, 3.5 Hz), 8.04 (s, 1H, H_2), 8.39 (s, 1H, H_8) ppm. ^{13}C-NMR (75.5 MHz, MeOH-d_4): δ 26.4 ($C_{3'}$), 33.9 ($C_{2'}$), 64.3 ($C_{5'}$), 83.9 ($C_{4'}$), 87.3 ($C_{1'}$), 125.7 (C_5), 140.4 (C_8), 146.6 (C_2), 149.3 (C_4), 159.0 (C_6) ppm.

5’-*O*-Levulinyl-β-D-uridine (2a)

^{1}H-NMR (300.13 MHz, $CDCl_3$)

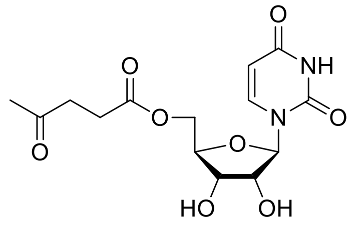

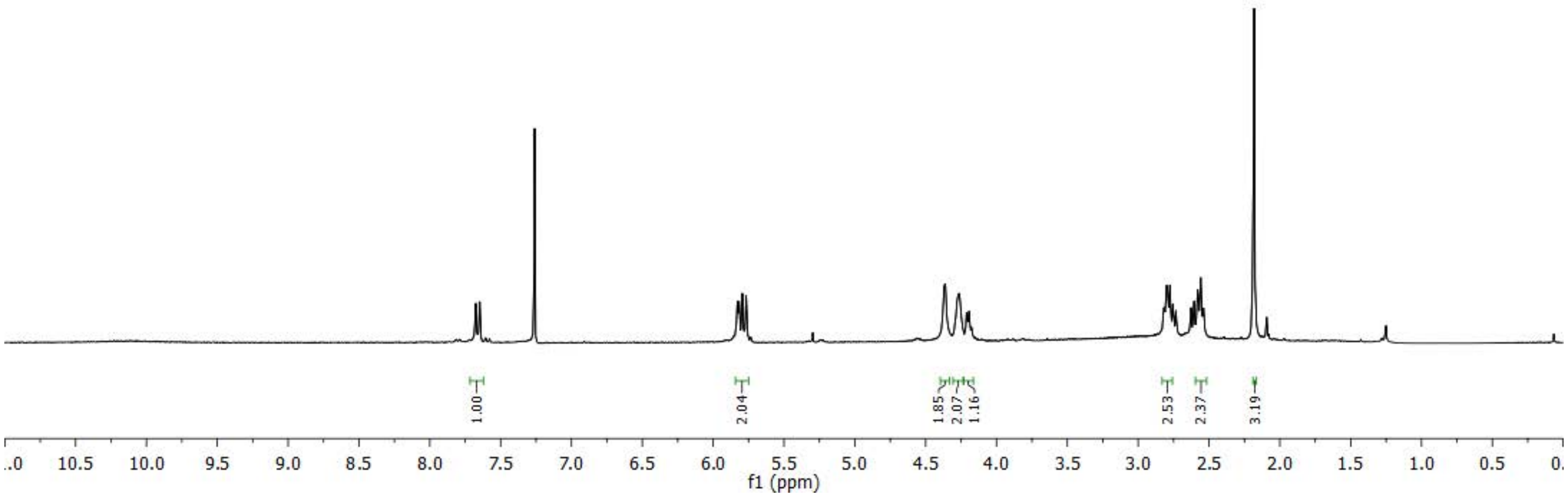

5’-*O*-Levulinyl-β-D-uridine (2a)

^{13}C-NMR (75.5 MHz, $CDCl_3$)

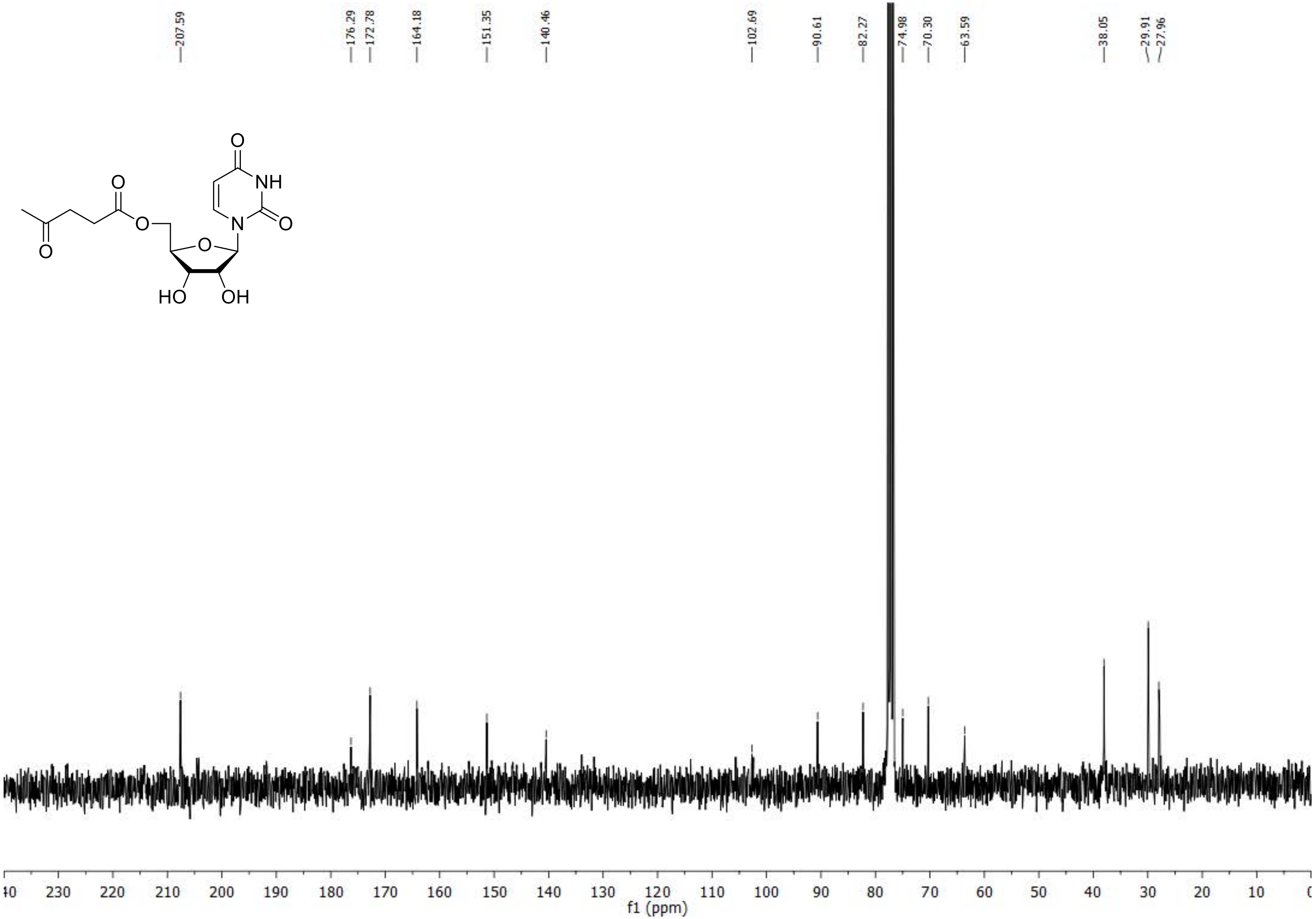

5'-*O*-Levulinyl-β-D-uridine (2a)

DEPT NMR (75.5 MHz, $CDCl_3$)

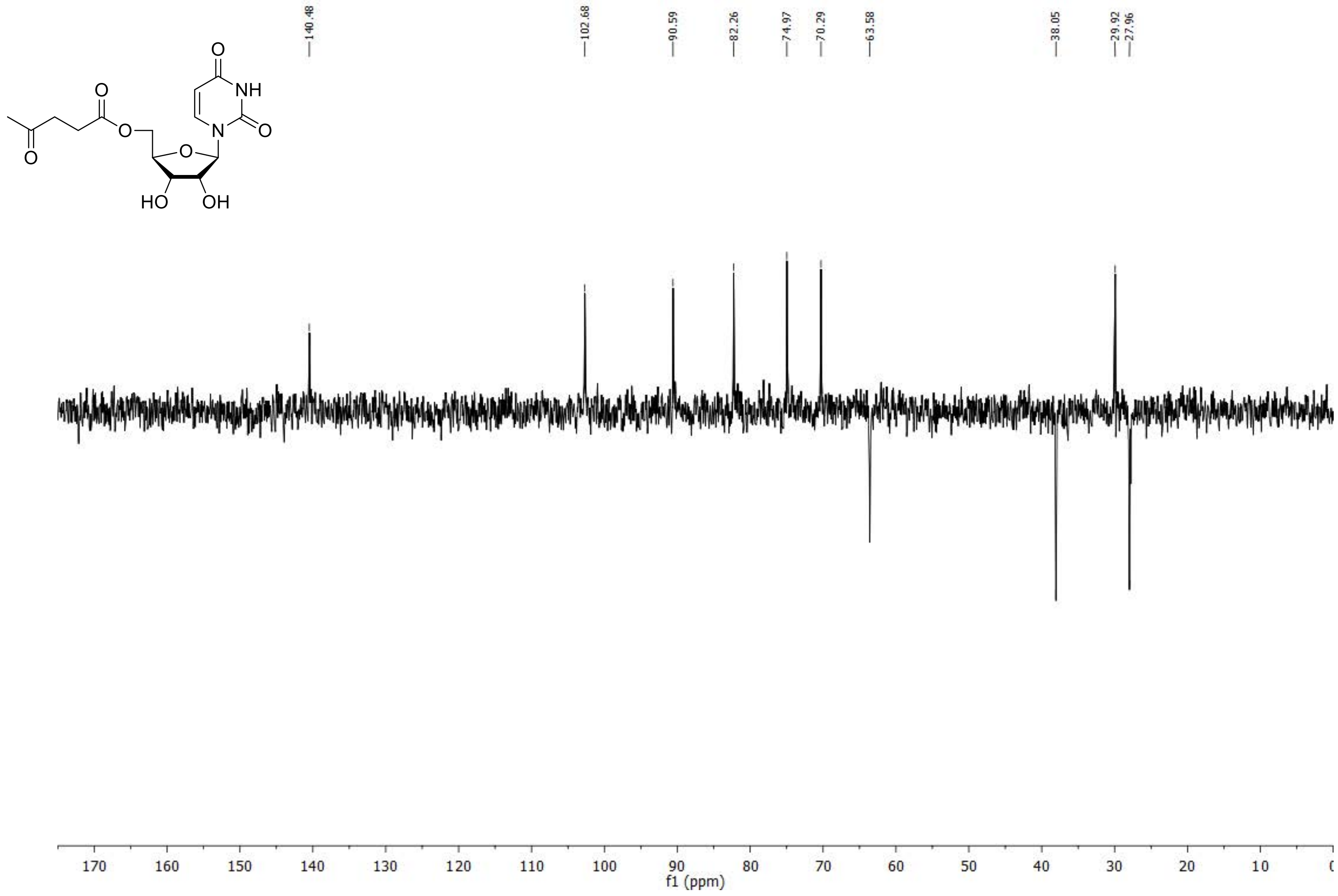

5’-*O*-Levulinyl-β-D-uridine (2a)

COSY NMR ($CDCl_3$)

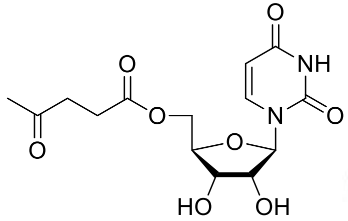

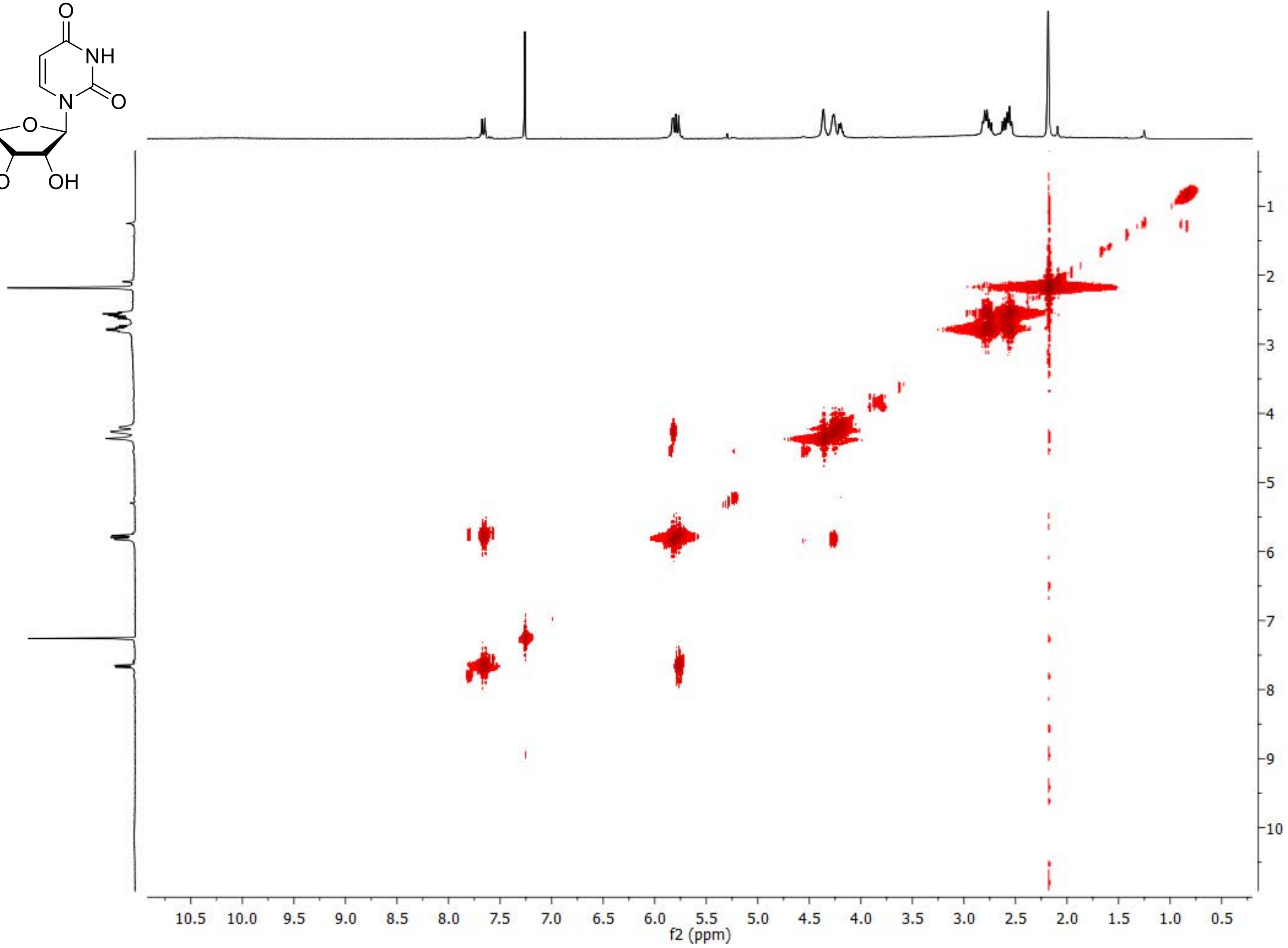

5’-*O*-Levulinyl-β-D-uridine (2a)

HSQC NMR ($CDCl_3$)

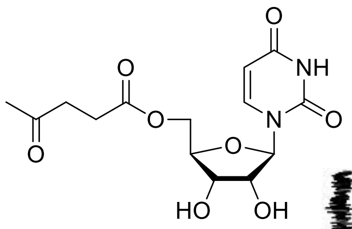

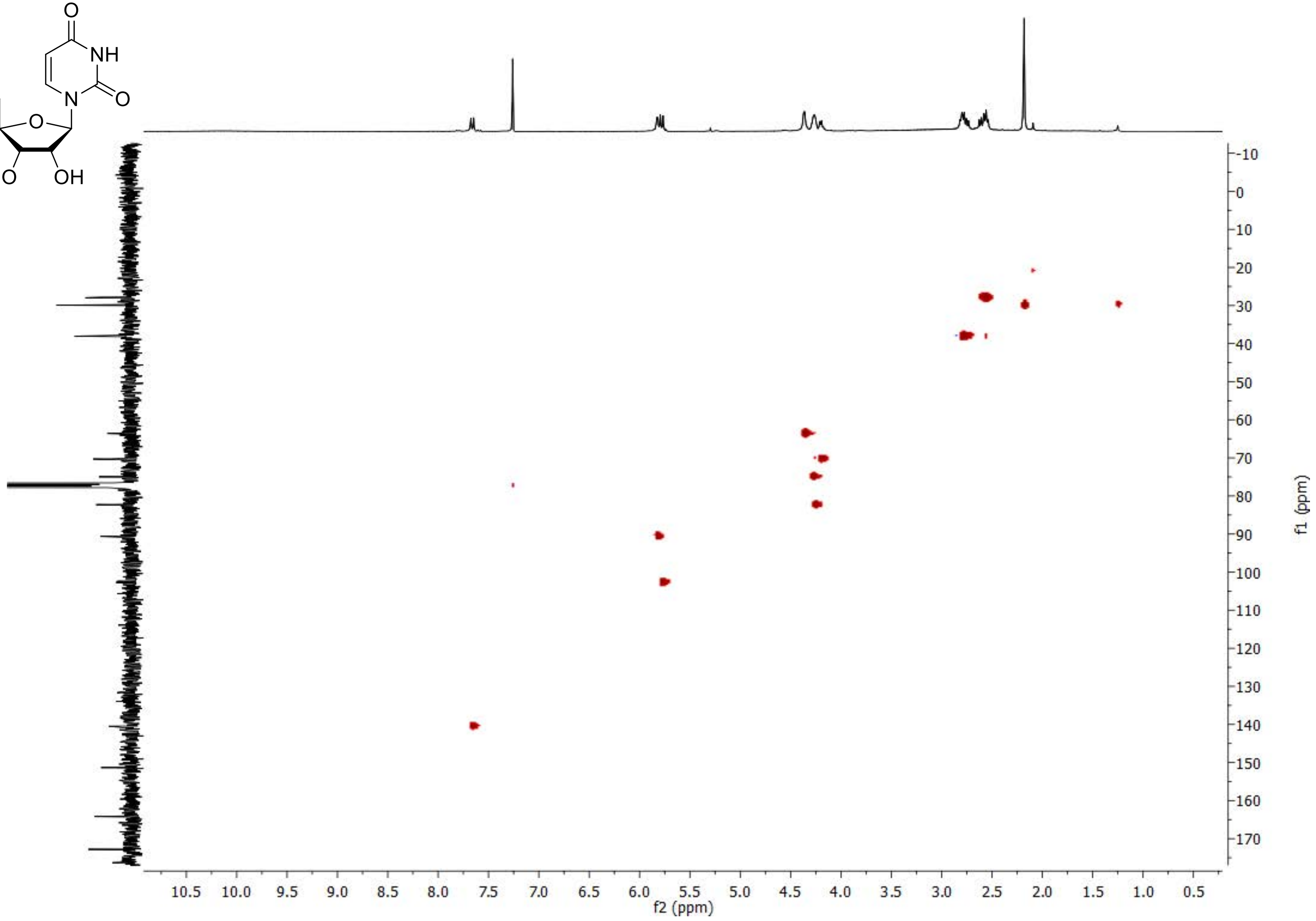

5'-*O*-Levulinyl-β-D-5-methyluridine (2b)

^{1}H-NMR (300.13 MHz, $CDCl_3$)

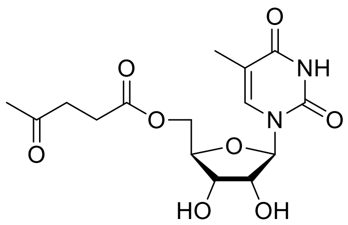

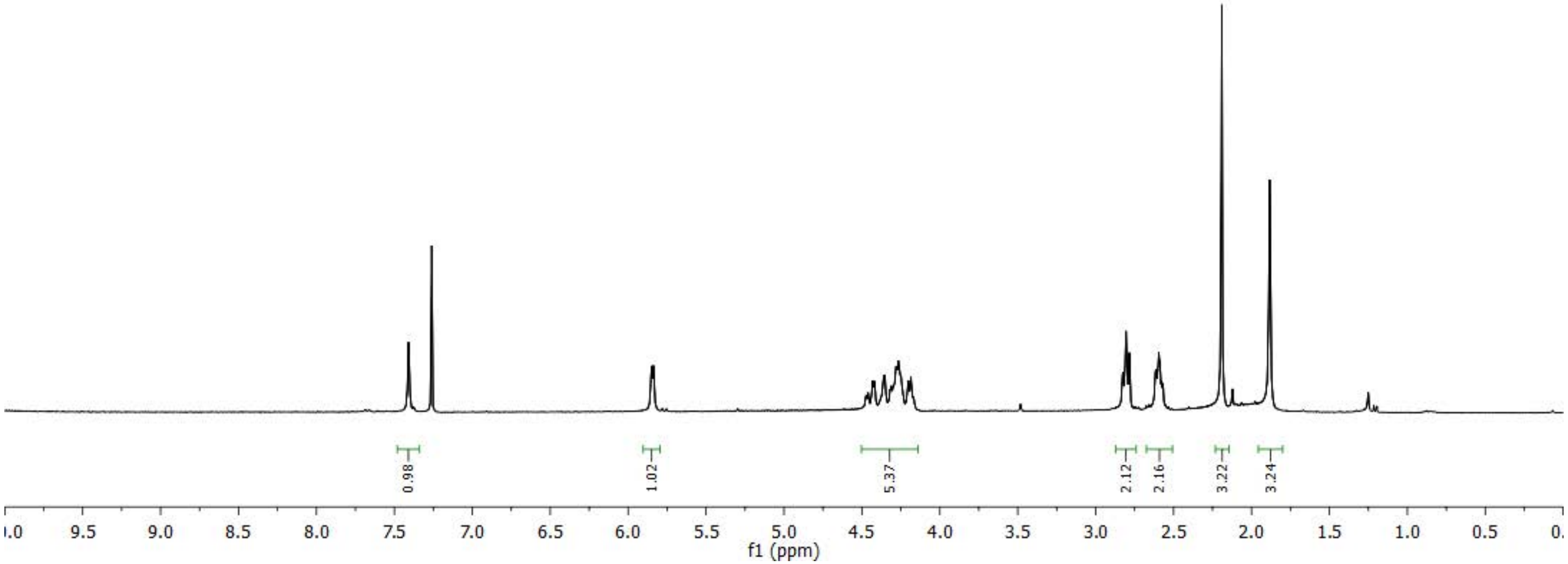

5’-*O*-Levulinyl-β-D-5-methyluridine (2b)

^{13}C-NMR (75.5 MHz, MeOH-d_4)

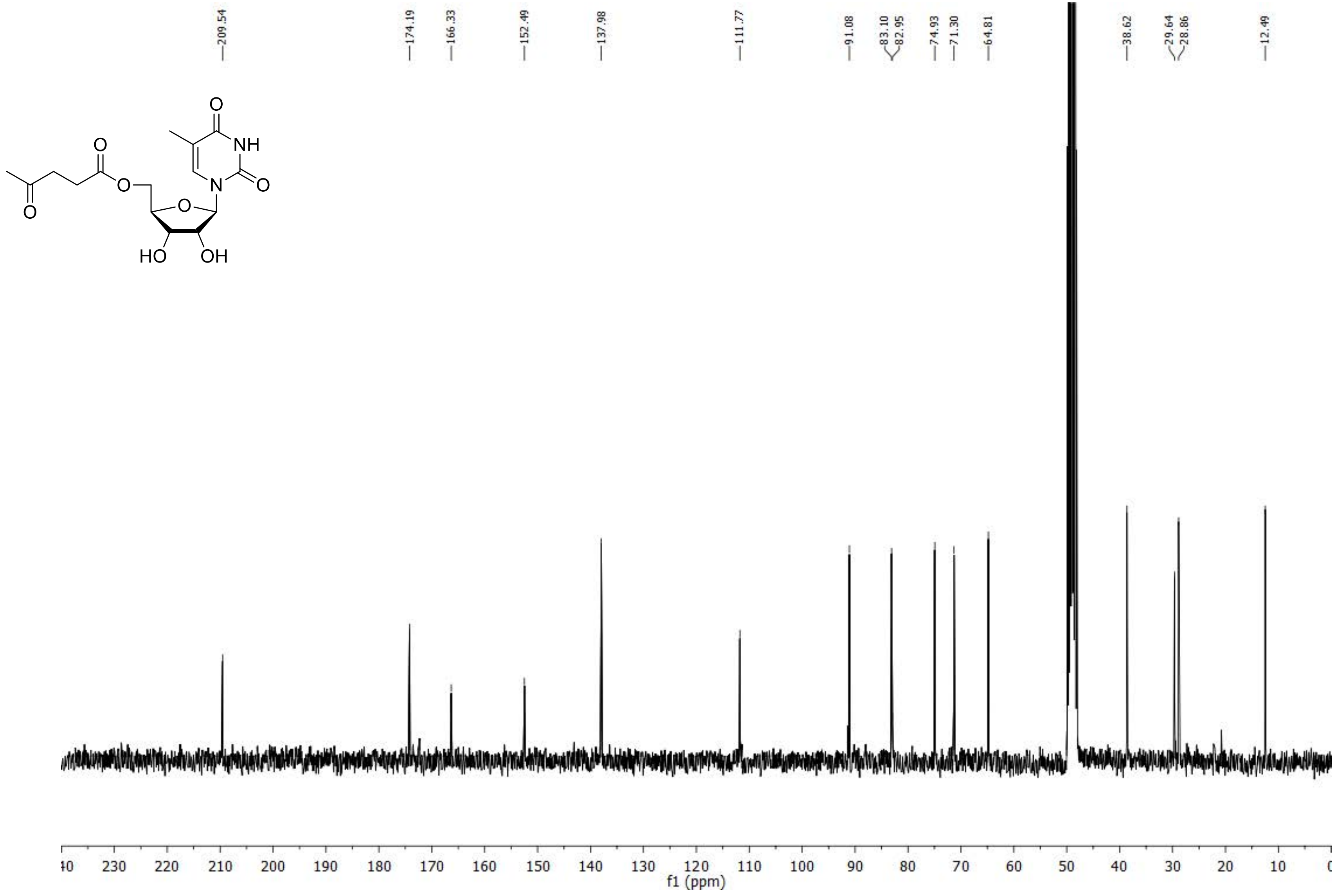

5’-*O*-Levulinyl-β-D-5-methyluridine (2b)

DEPT NMR (75.5 MHz, $CDCl_3$)

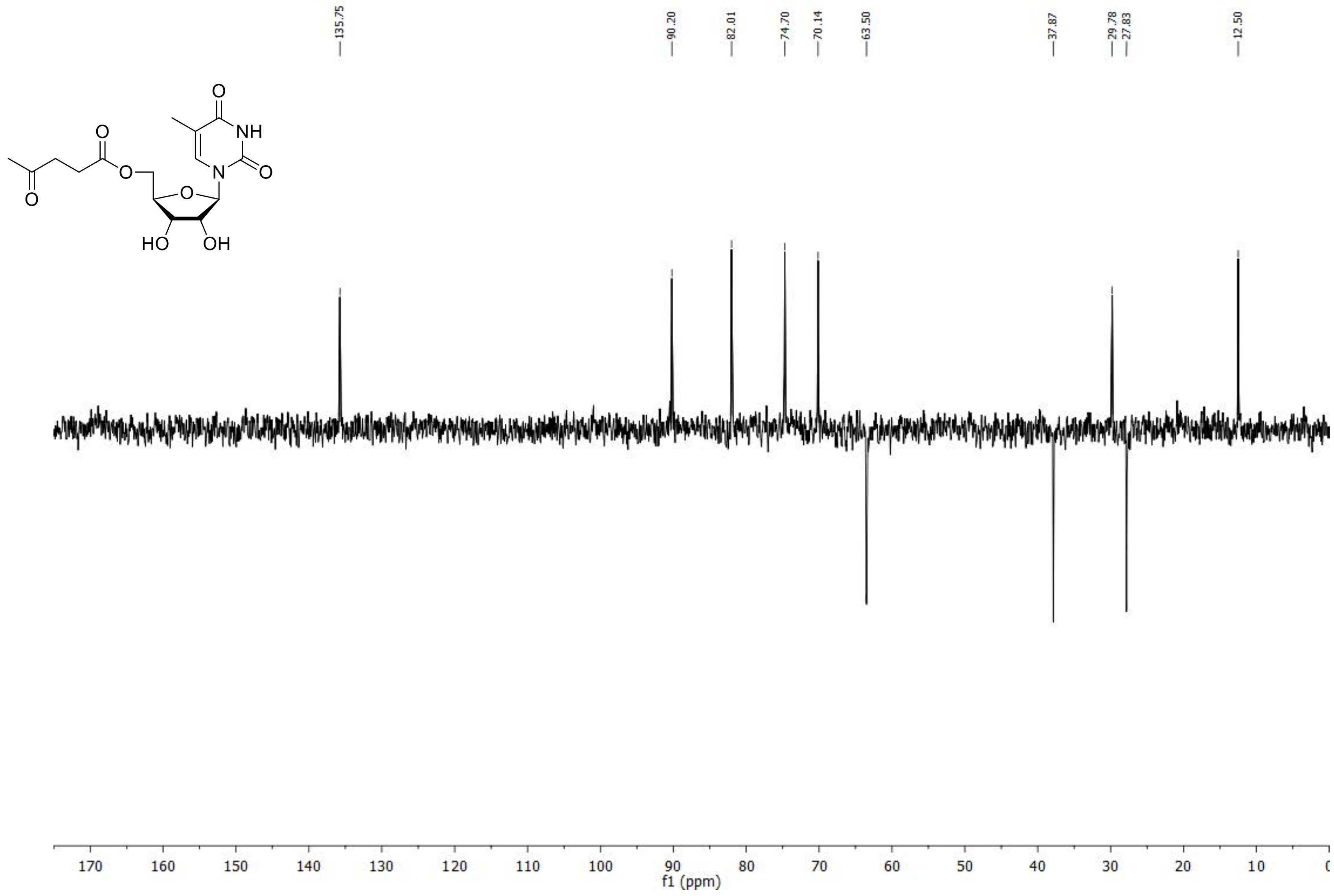

5’-*O*-Levulinyl-β-D-5-methyluridine (2b)

COSY NMR ($CDCl_3$)

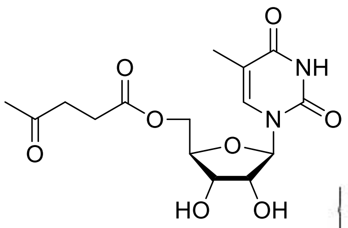

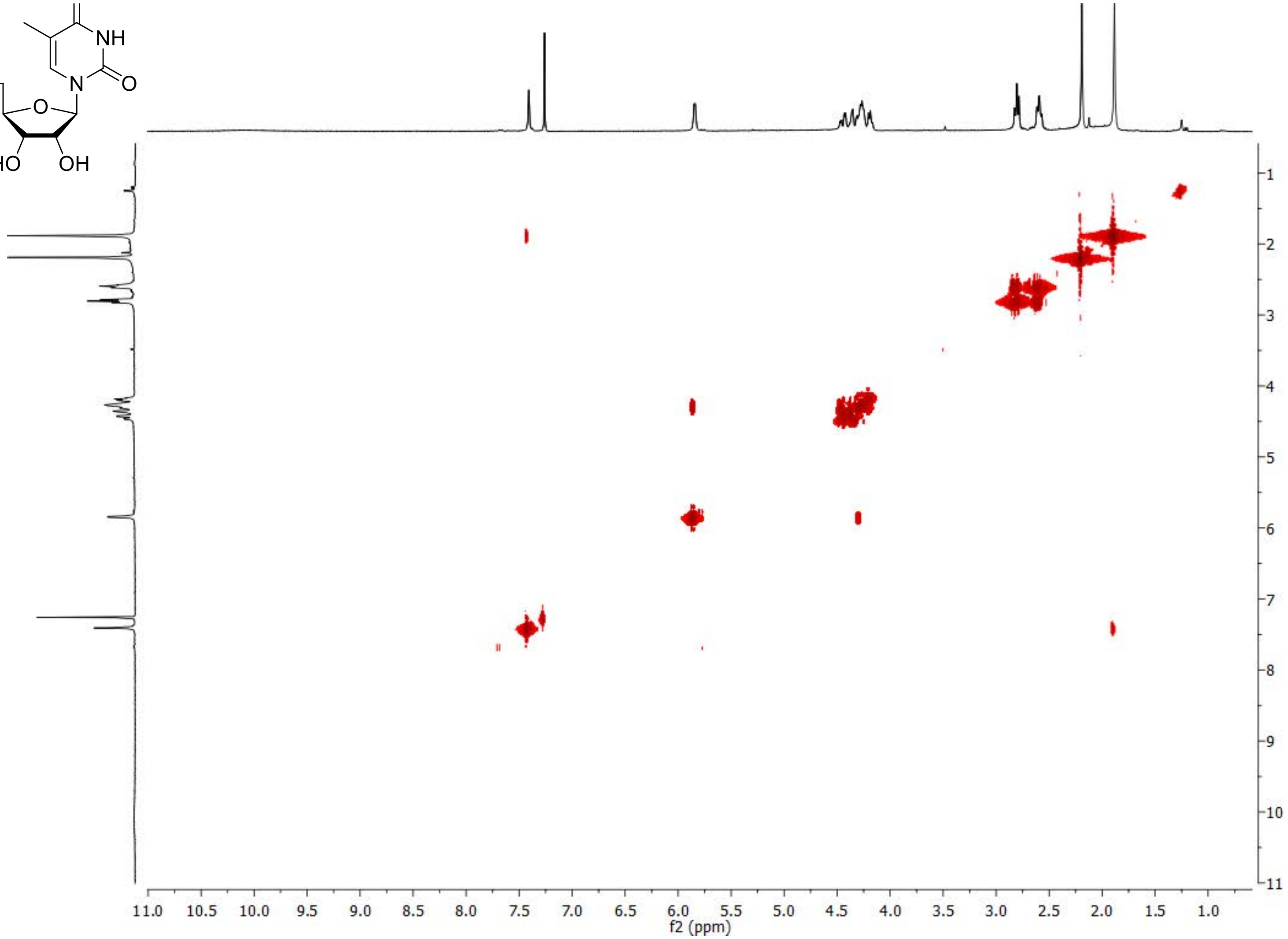

HSQC NMR ($CDCl_3$)

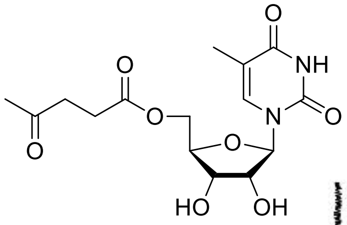

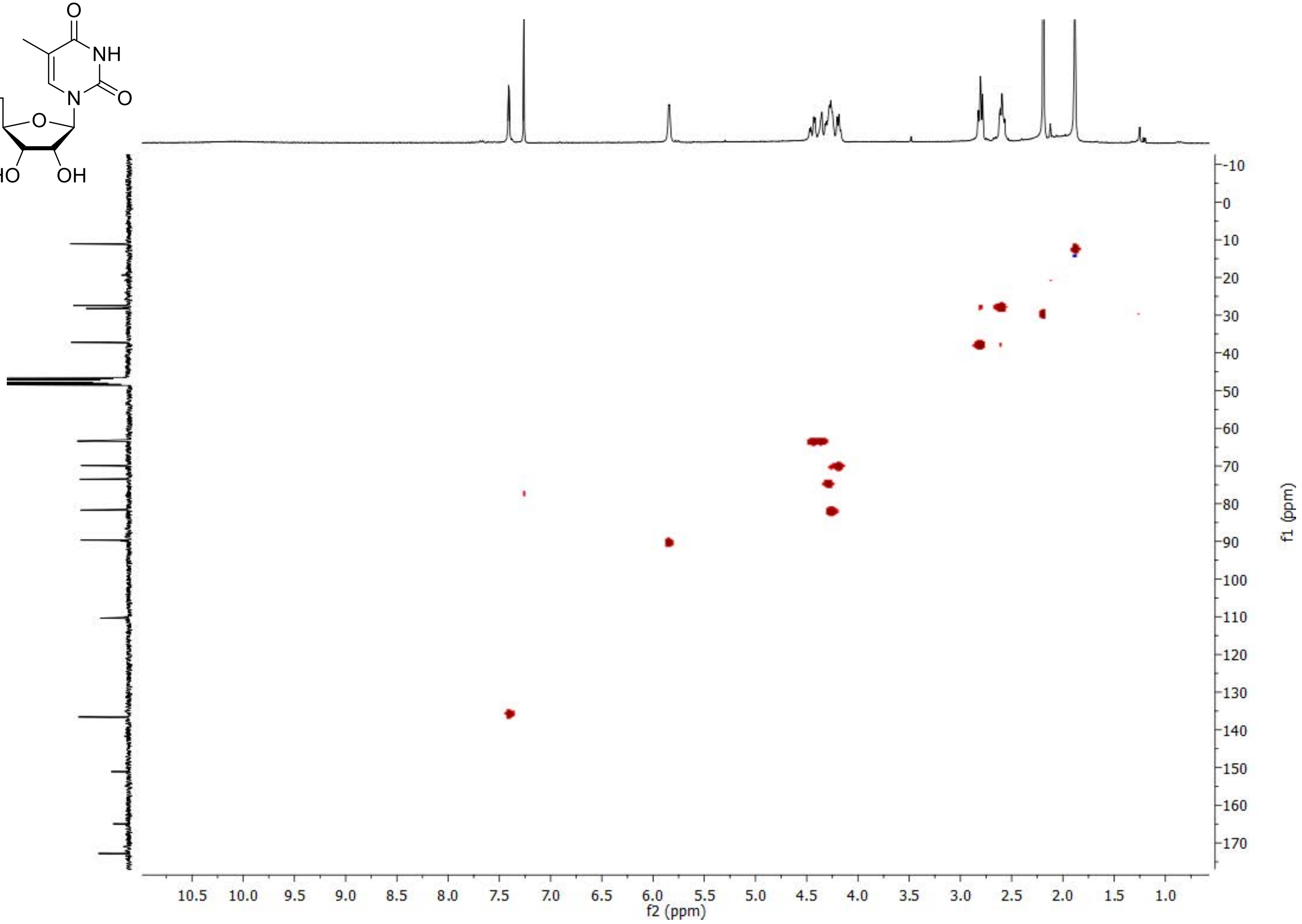

5'-*O*-Levulinyl-β-D-cytidine (2c)

^{1}H-NMR (300.13 MHz, MeOD-d_4)

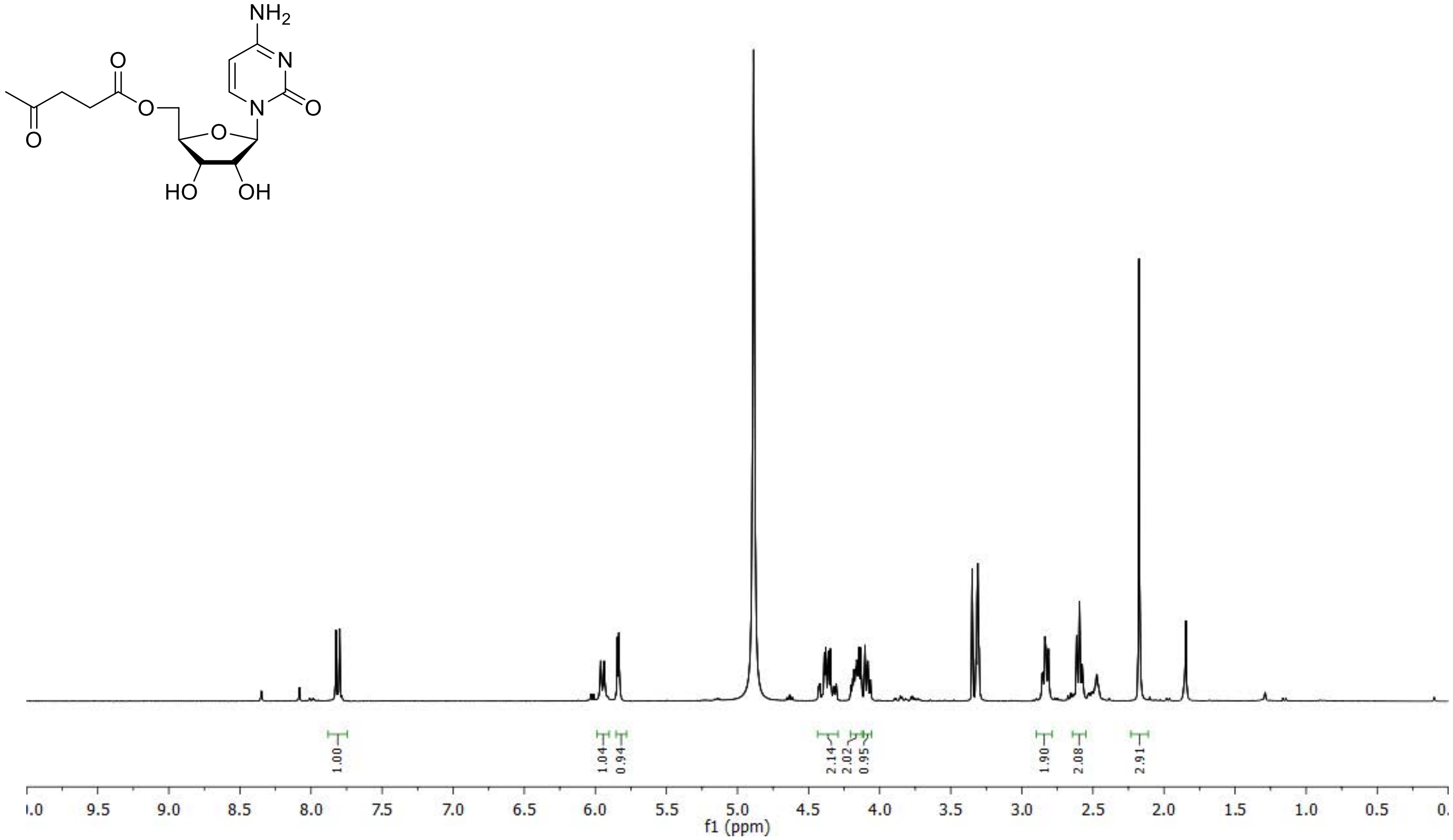

5’-*O*-Levulinyl-β-D-cytidine (2c)

^{13}C-NMR (75.5 MHz, MeOD-d_4)

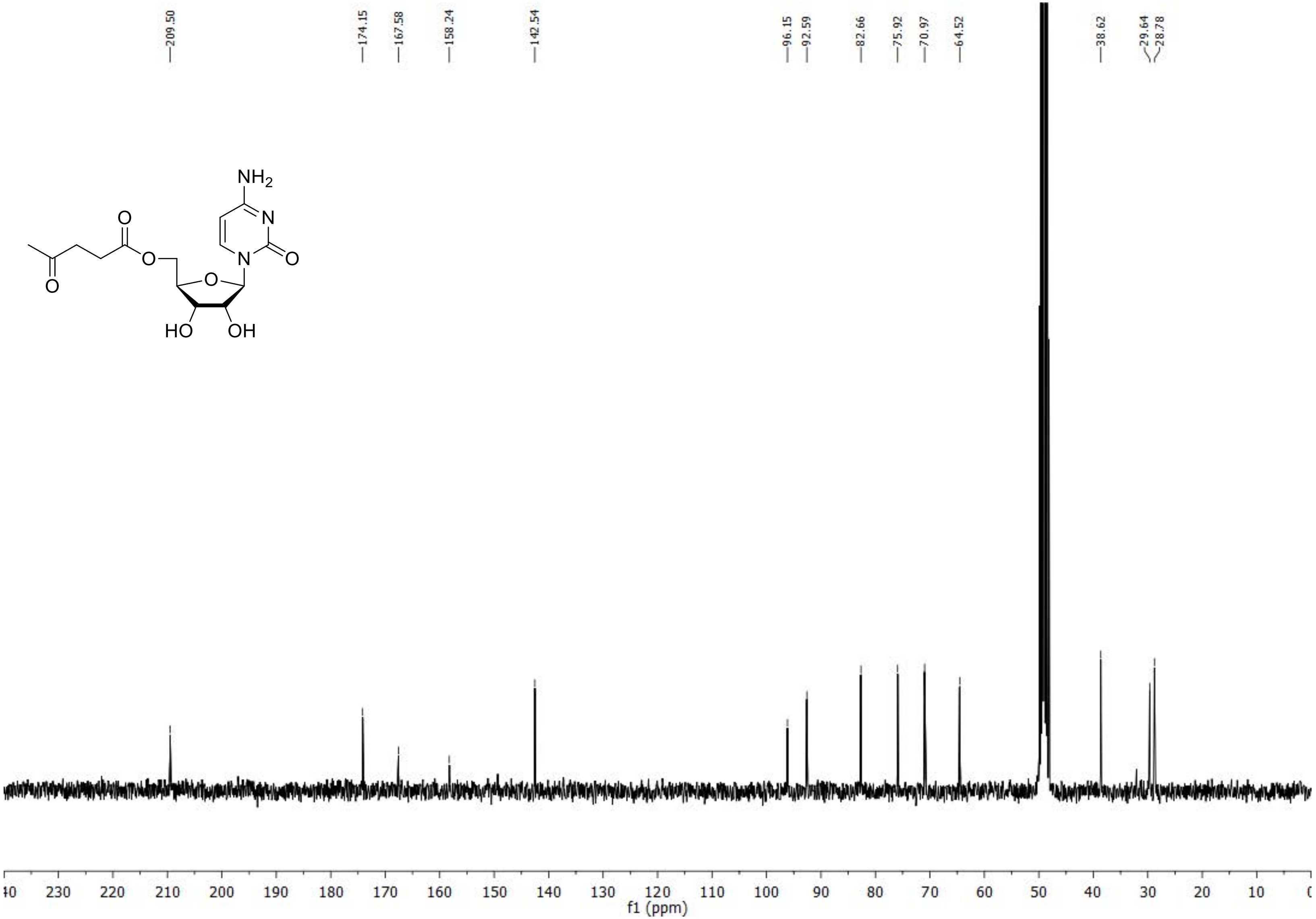

5’-*O*-Levulinyl-β-D-cytidine (2c)

DEPT NMR (75.5 MHz, MeOD-d_4)

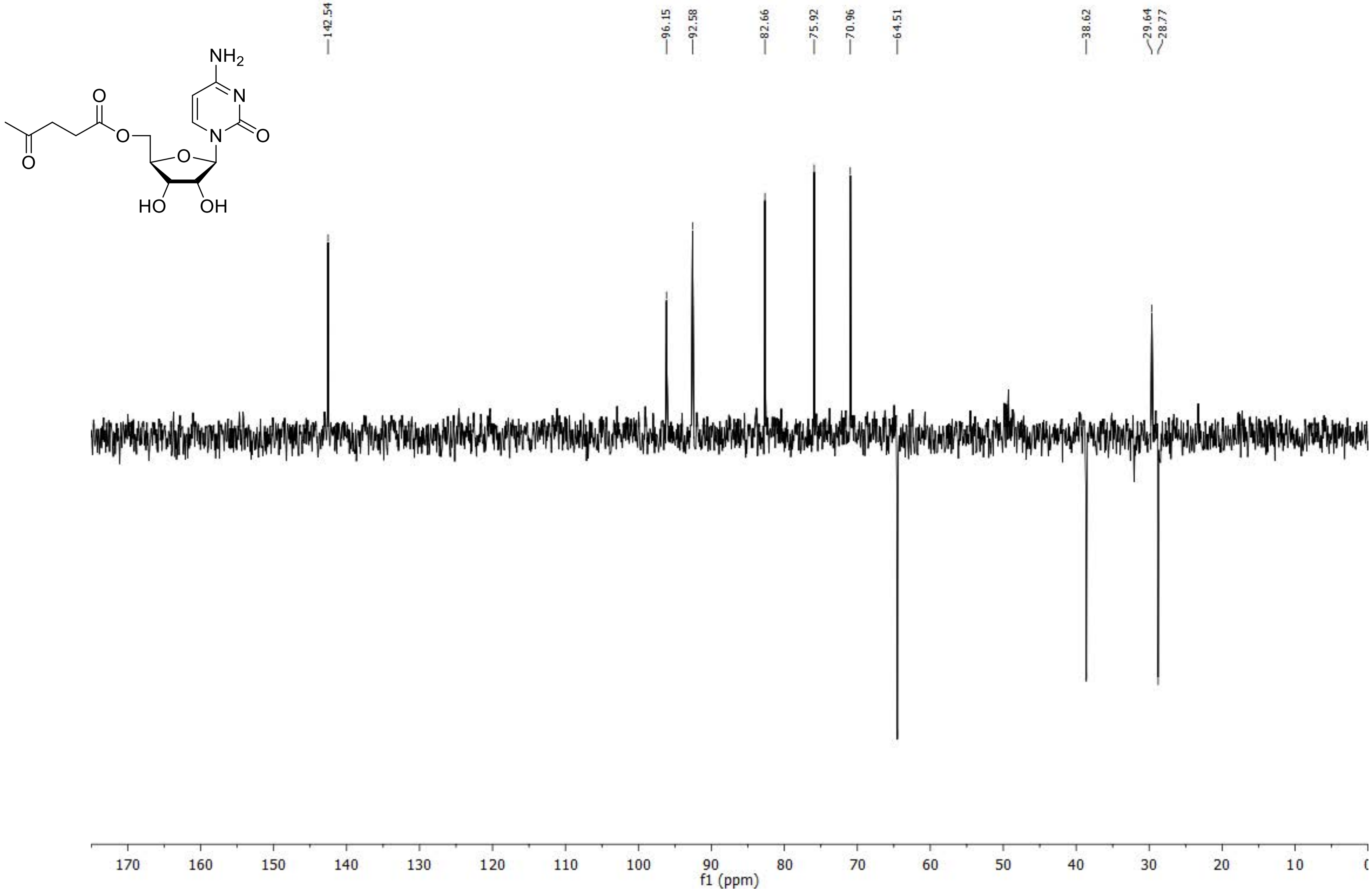

5’-*O*-Levulinyl-β-D-cytidine (2c)

COSY NMR (MeOD-d_4)

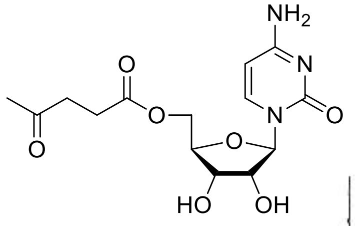

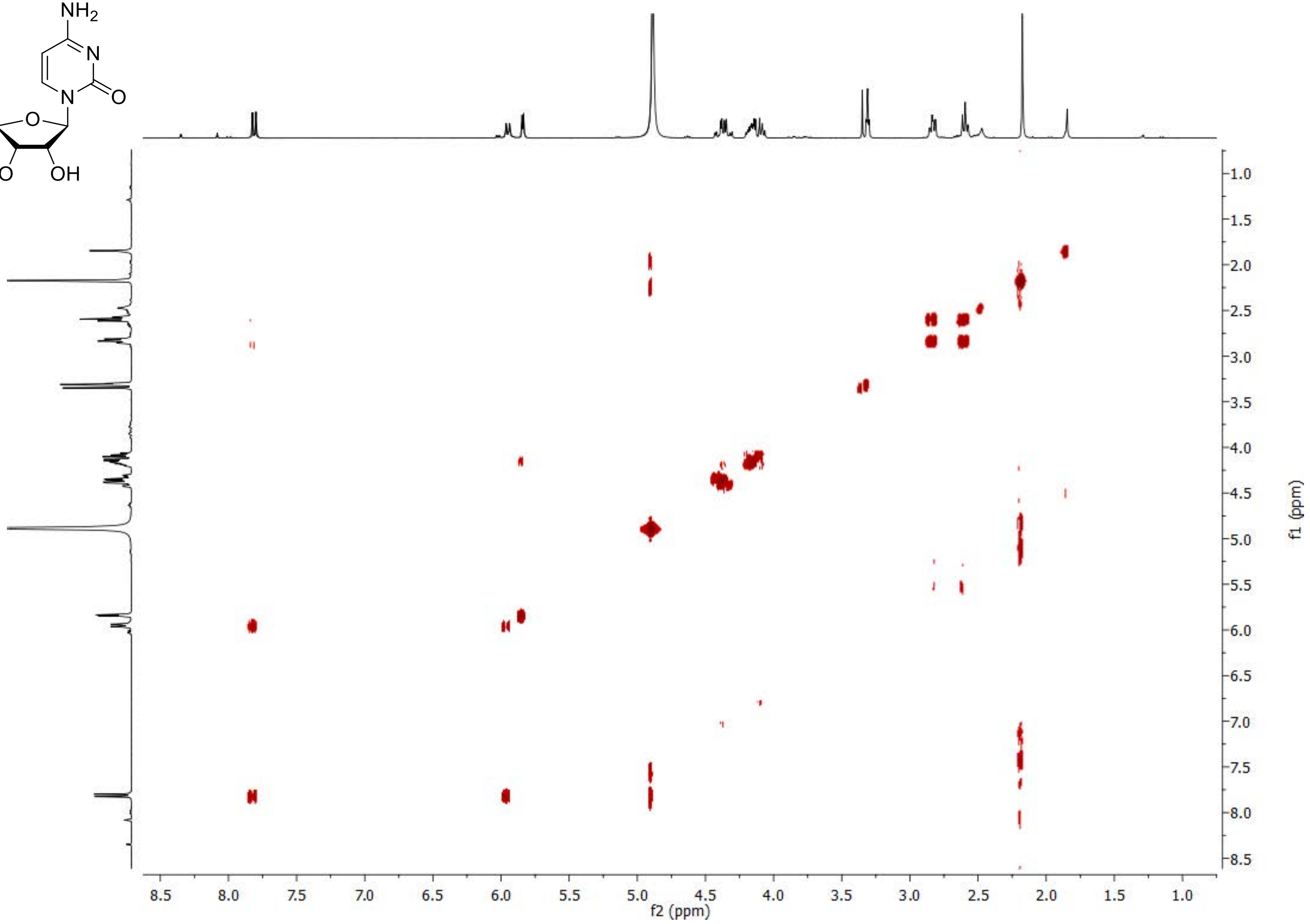

5'-*O*-Levulinyl-β-D-cytidine (2c)

HSQC NMR (MeOD-d_4)

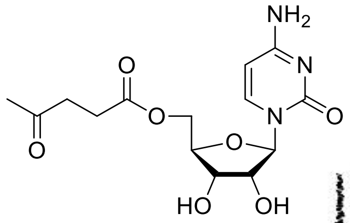

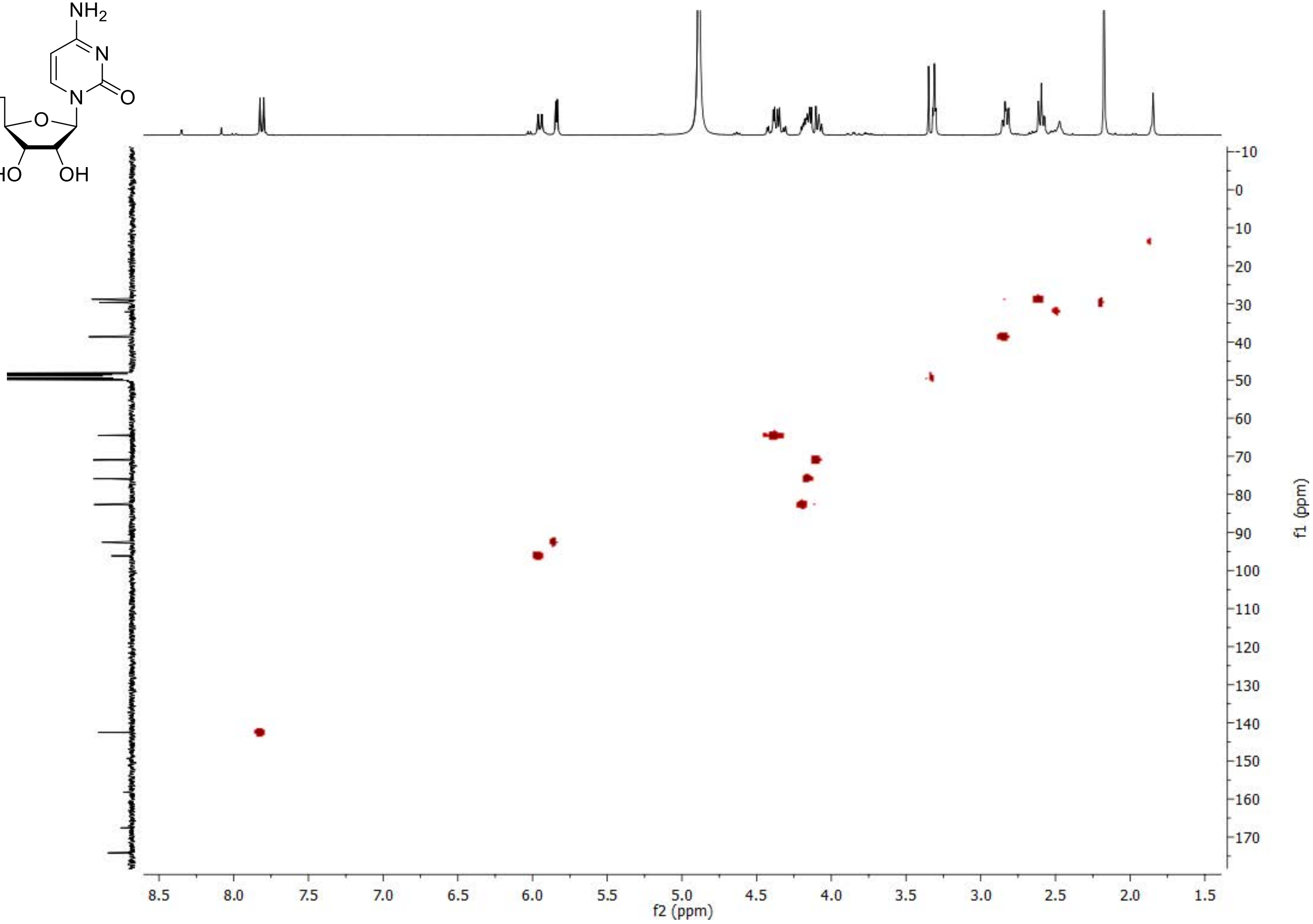

N-Benzoyl-5'-*O*-Levulinyl-β-D-cytidine (2d)

^{1}H-NMR (300.13 MHz, DMSO-d_6)

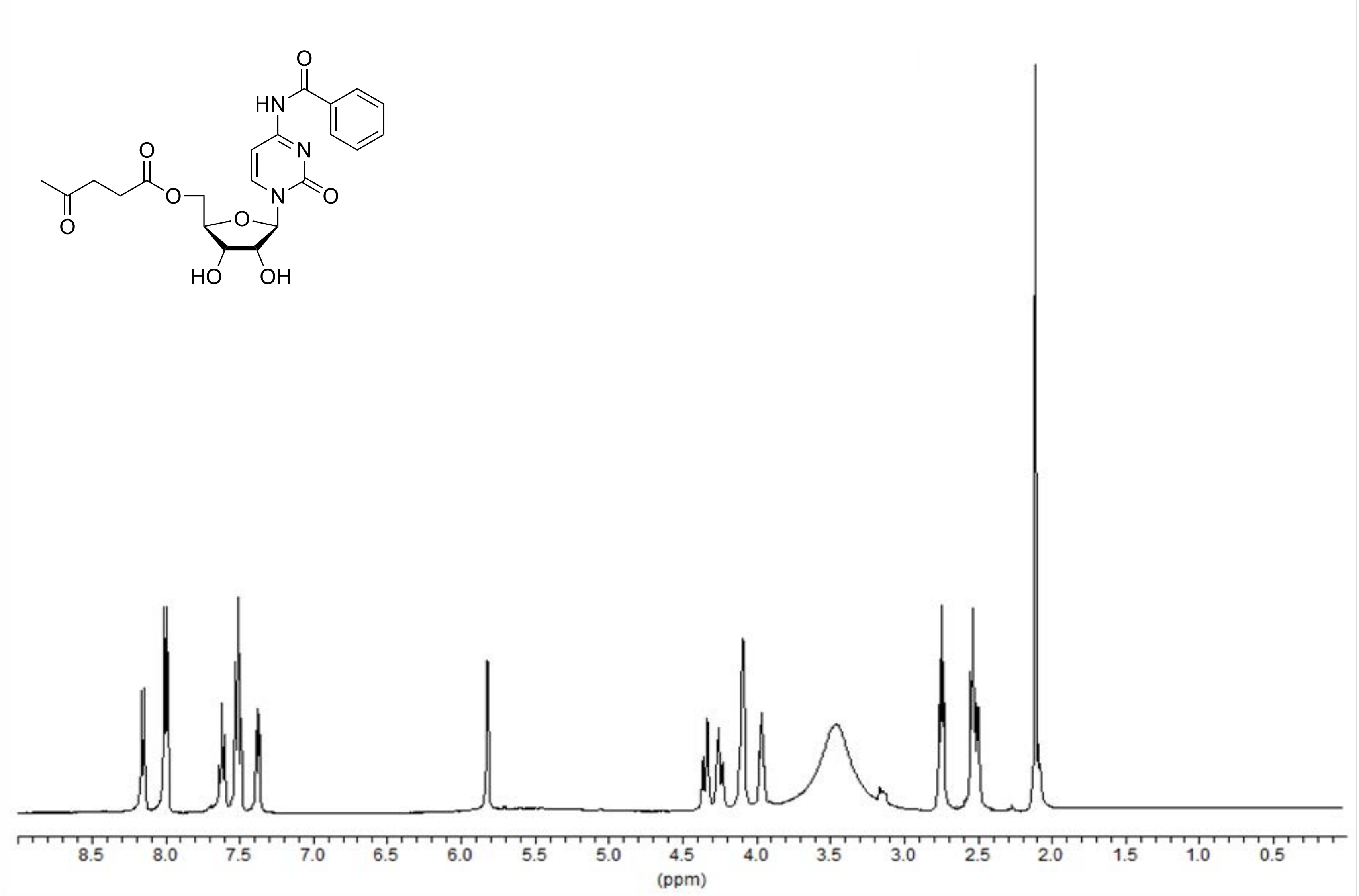

^{13}C-NMR (75.5 MHz, DMSO-d_6)

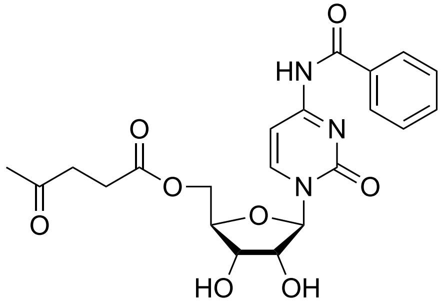

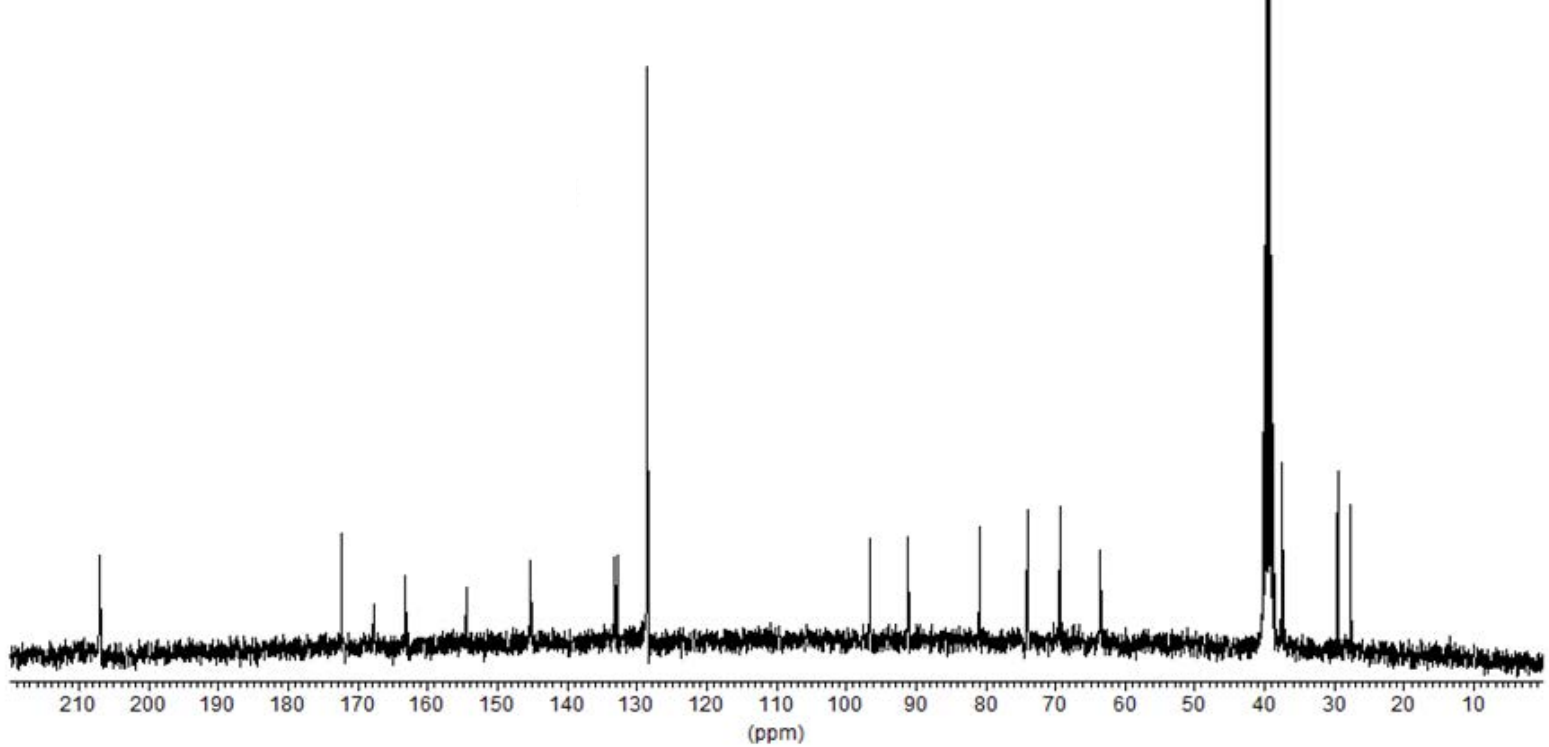

N-Benzoyl-5'-*O*-Levulinyl-β-D-cytidine (2d)

DEPT NMR (75.5 MHz, DMSO-d_6)

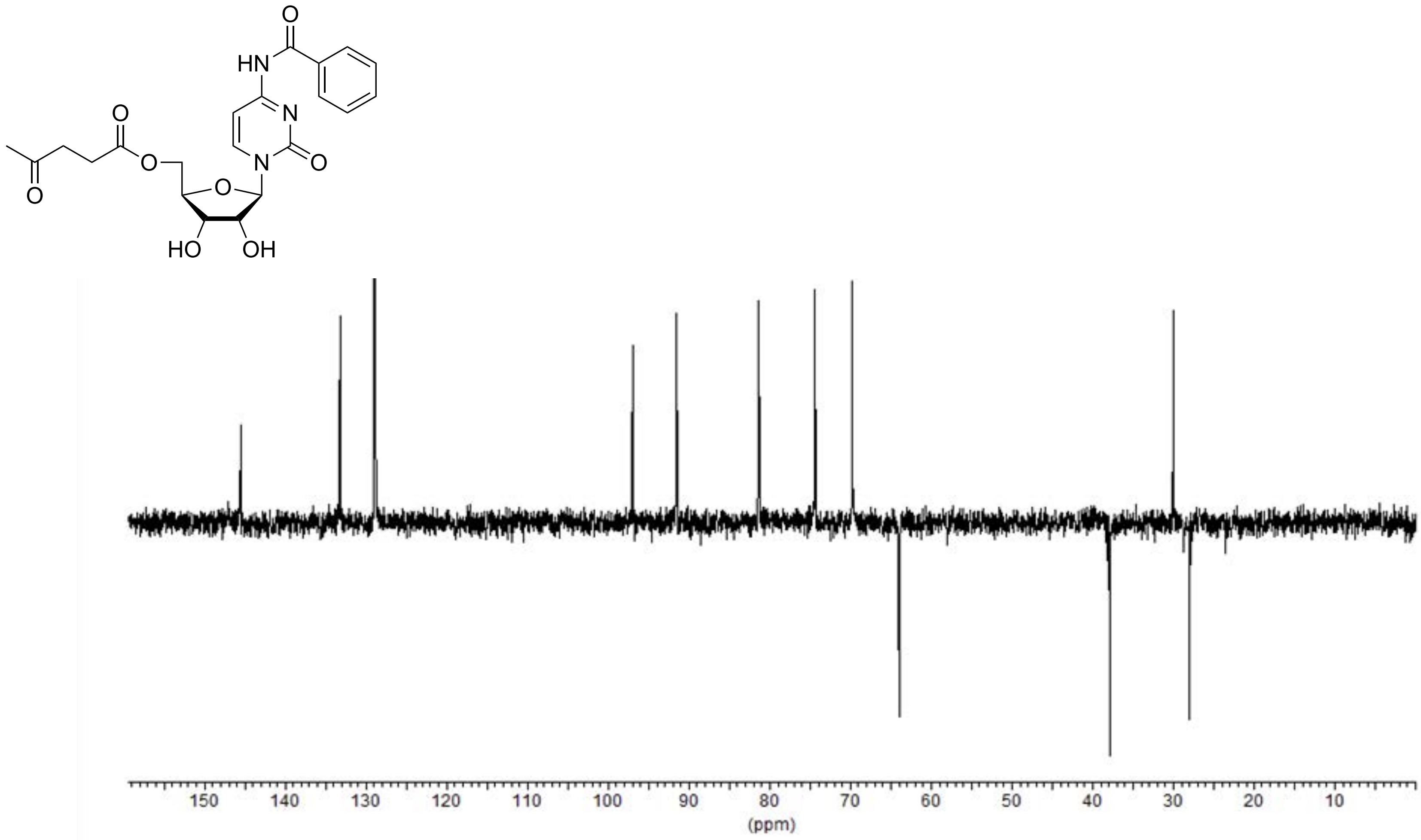

COSY NMR (300.13 MHz, DMSO-d_6)

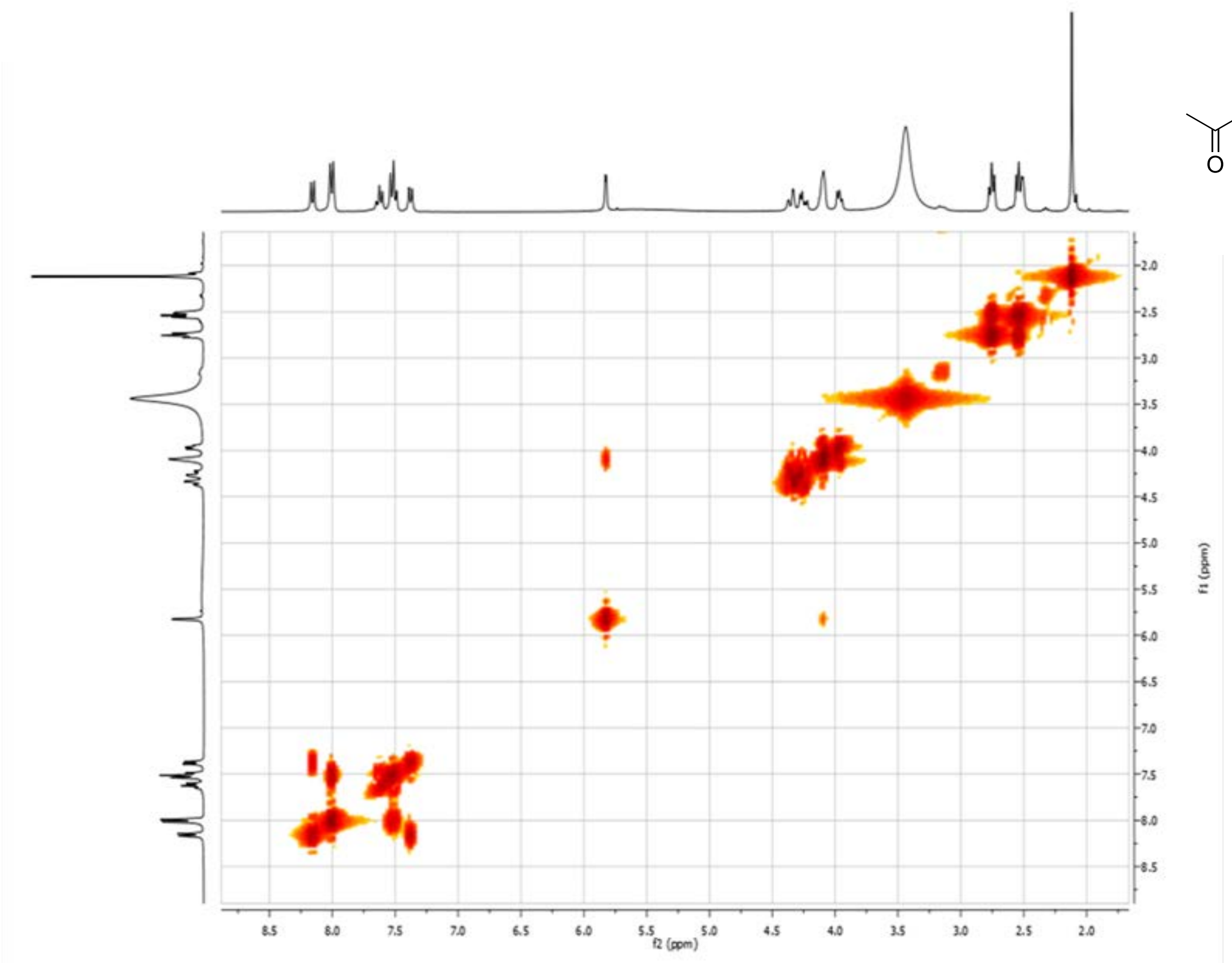

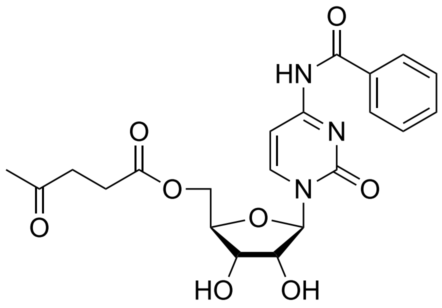

N-Benzoyl-5’-O-Levulinyl-β-D-cytidine (2d)

HSQC NMR (300.13 MHz, DMSO-d_6)

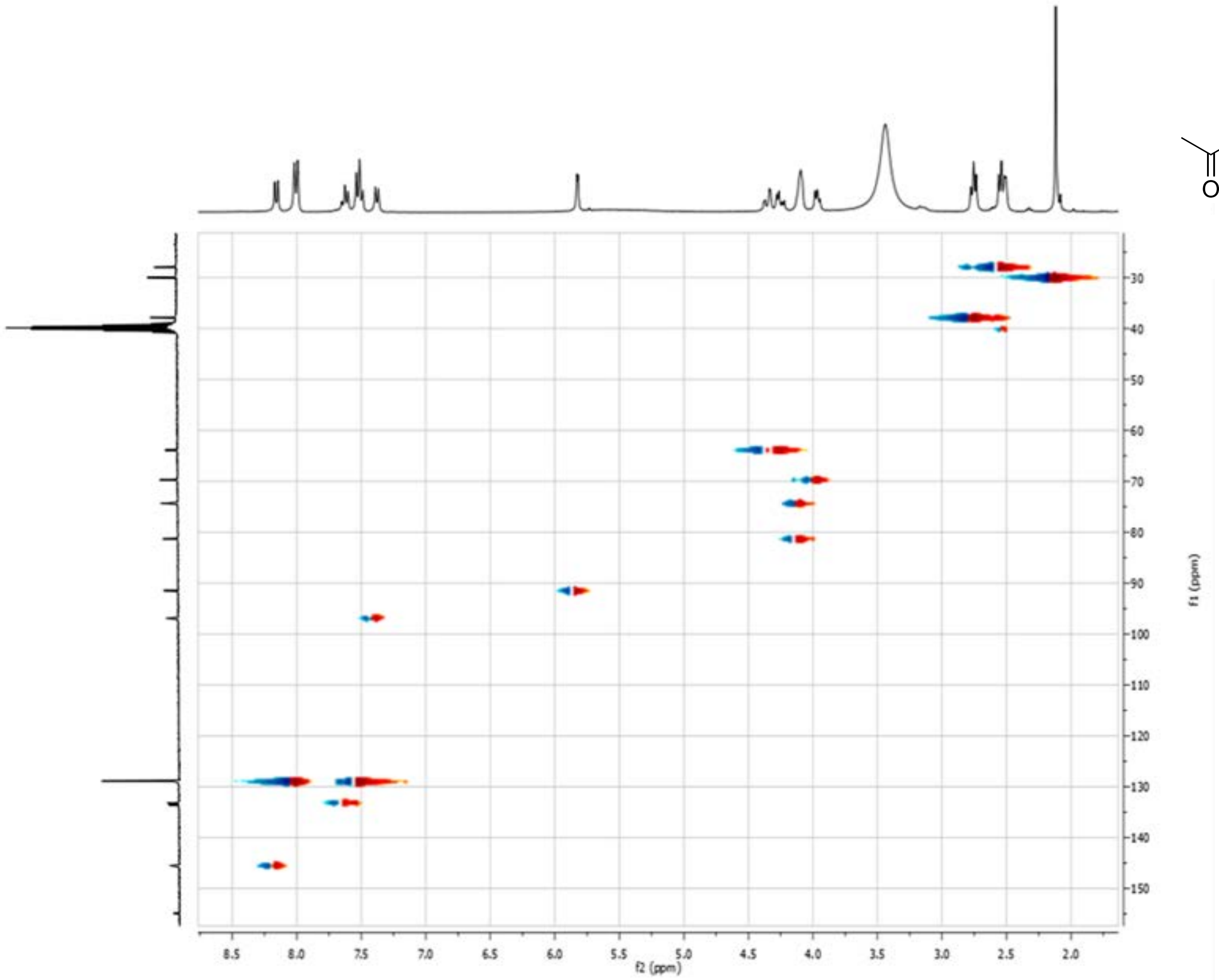

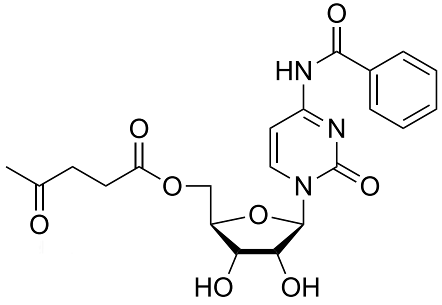

N-Benzoyl-5’-*O*-Levulinyl-β-D-cytidine (2d)

HMBC NMR (300.13 MHz, DMSO-d_6)

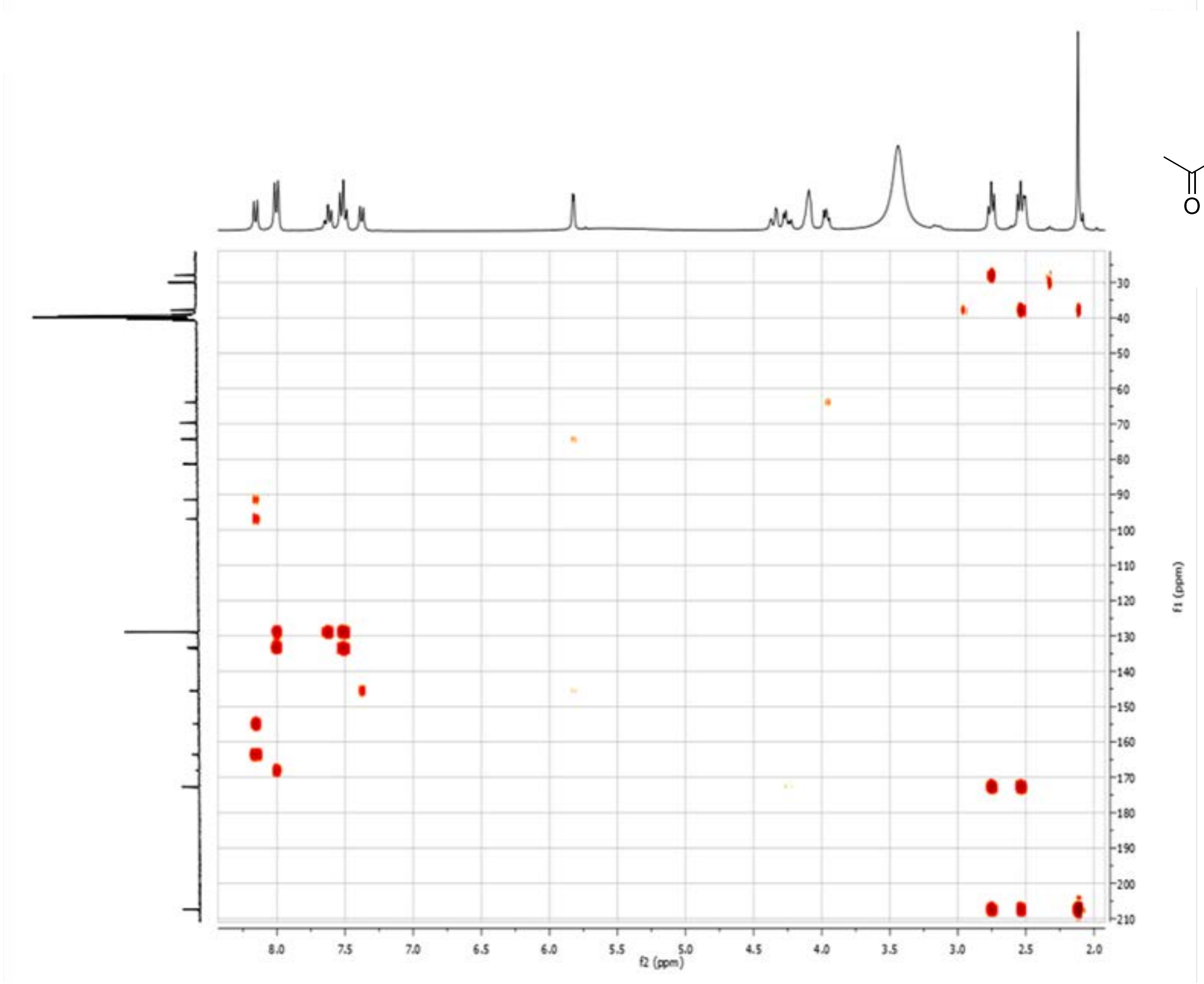

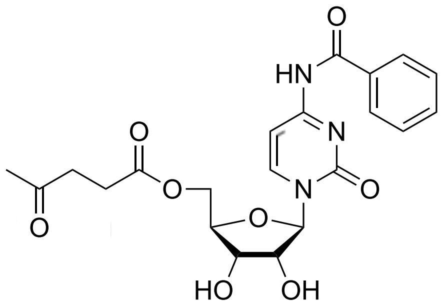

5'-*O*-Levulinyl-β-D-adenosine (2e)

^{1}H-NMR (300.13 MHz, MeOD-d_4)

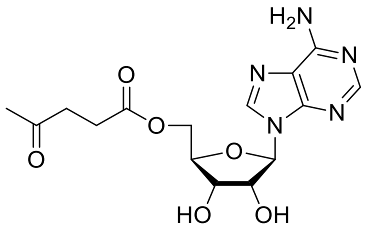

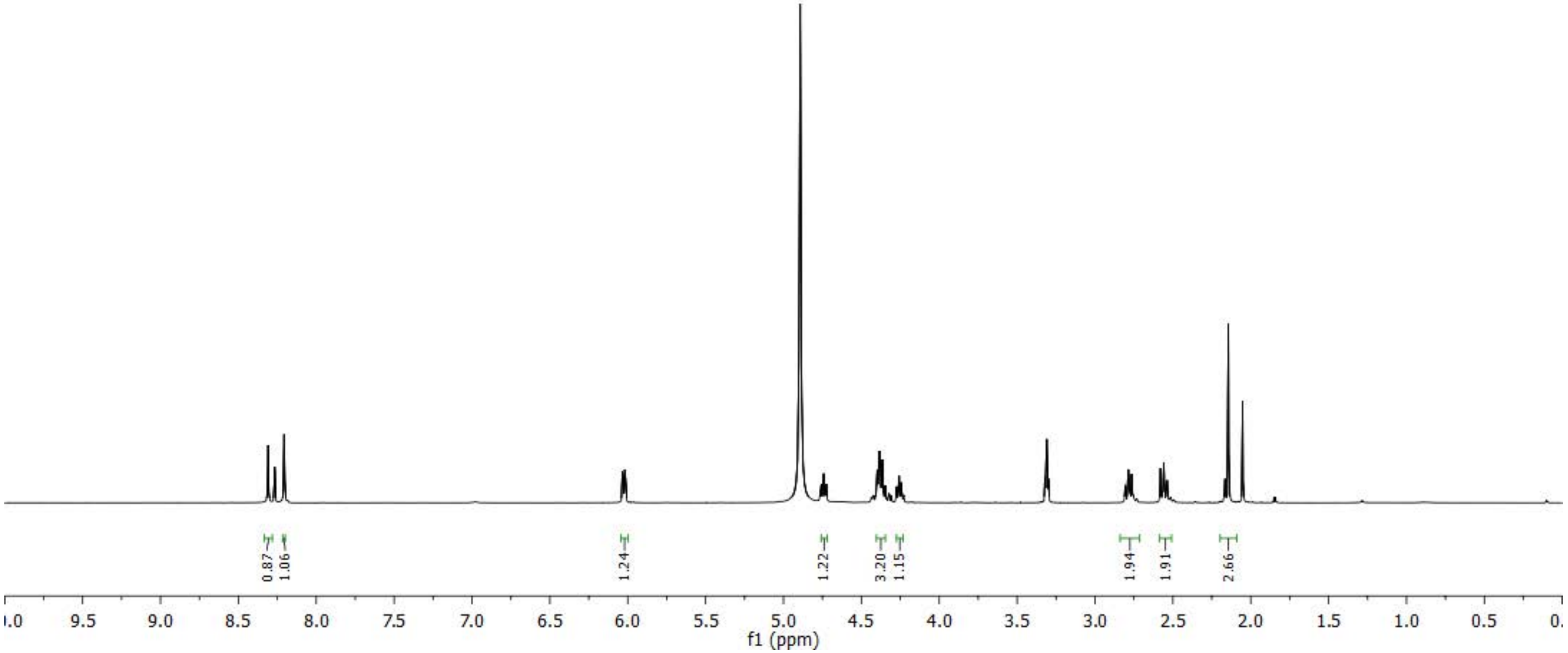

5’-*O*-Levulinyl-β-D-adenosine (2e)

^{13}C-NMR (75.5 MHz, MeOD-d_4)

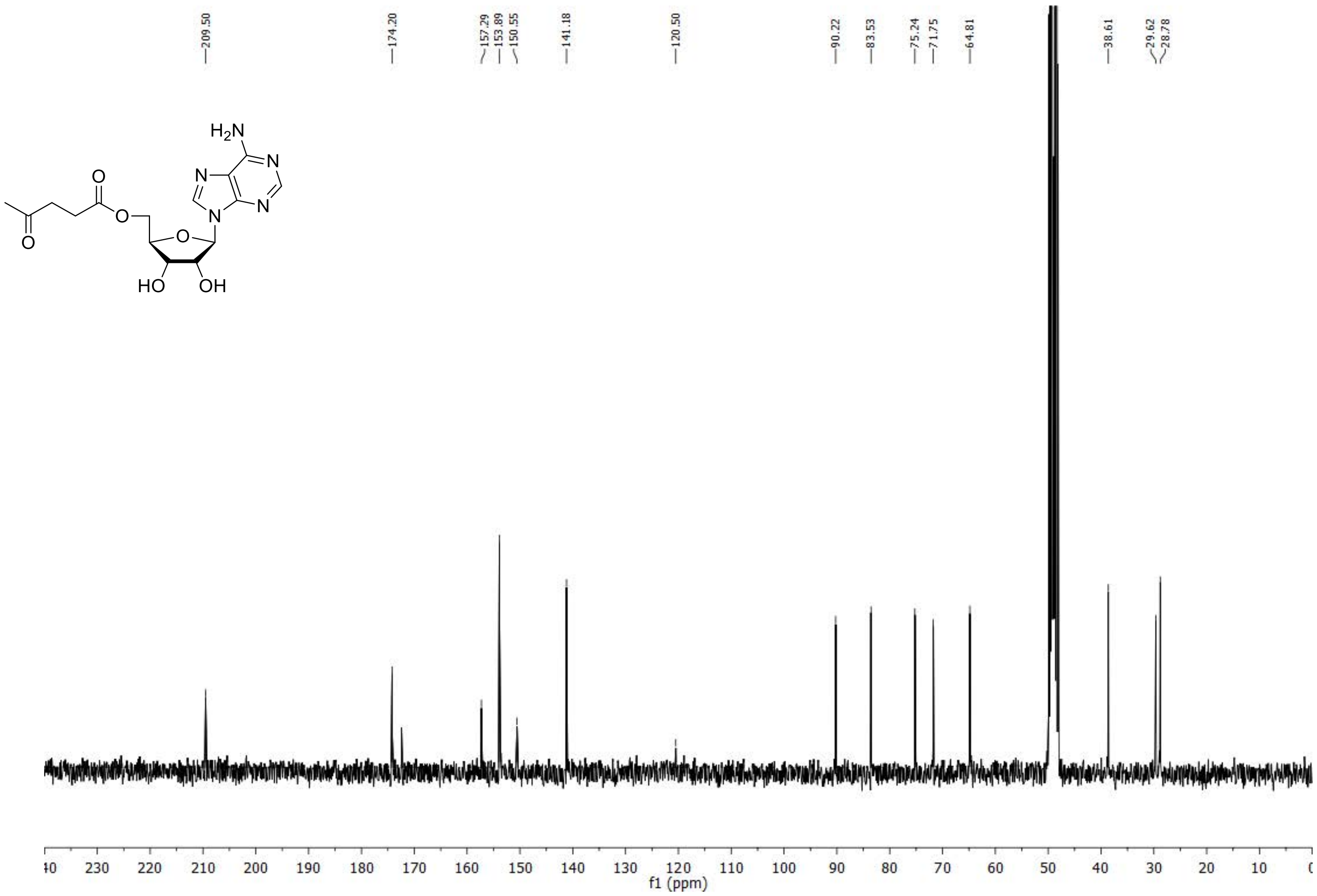

5’-*O*-Levulinyl-β-D-adenosine (2e)

DEPT NMR (75.5 MHz, MeOD-d_4)

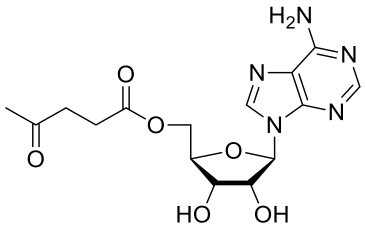

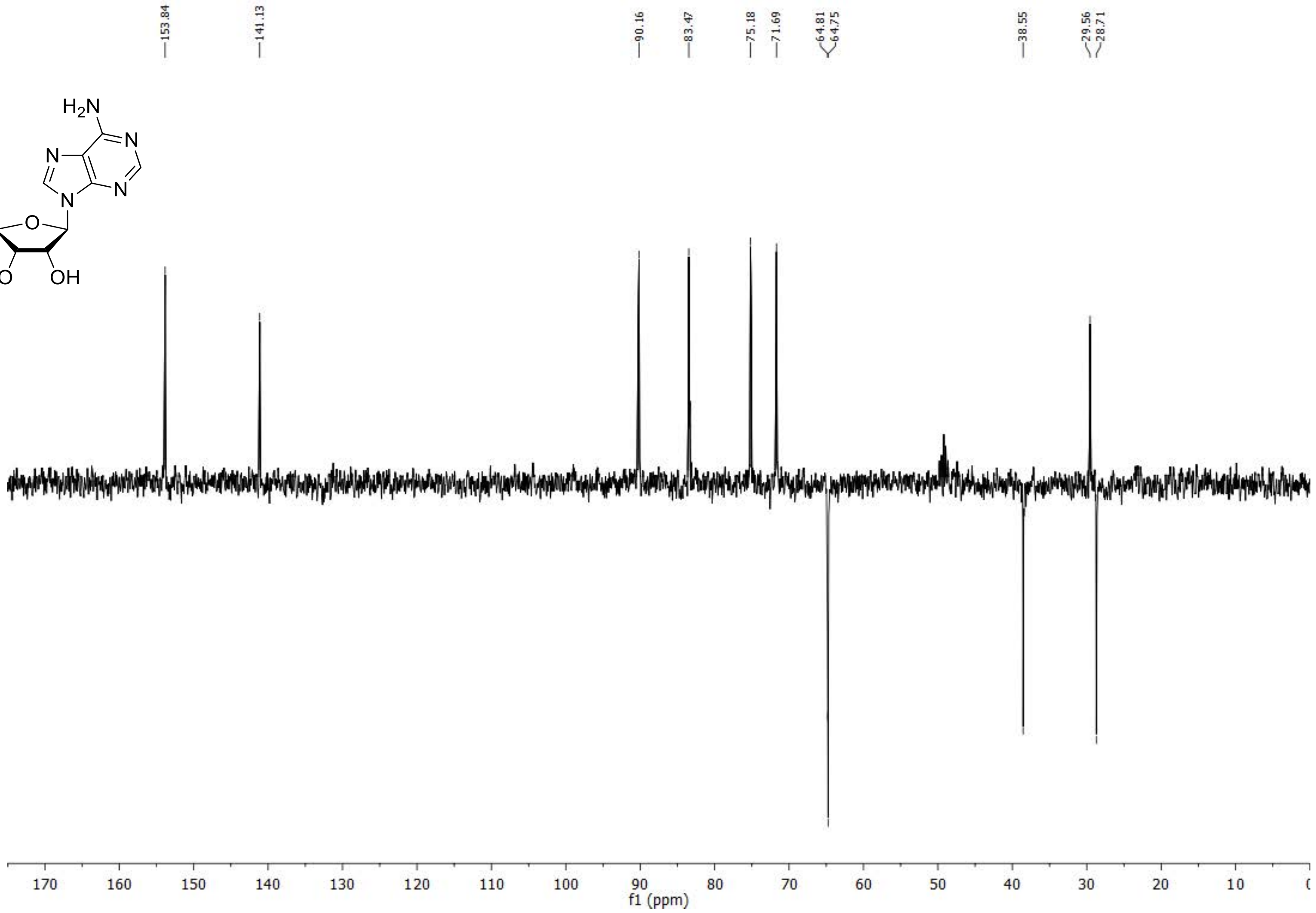

5’-*O*-Levulinyl-β-D-adenosine (2e)

COSY NMR (MeOD-d_4)

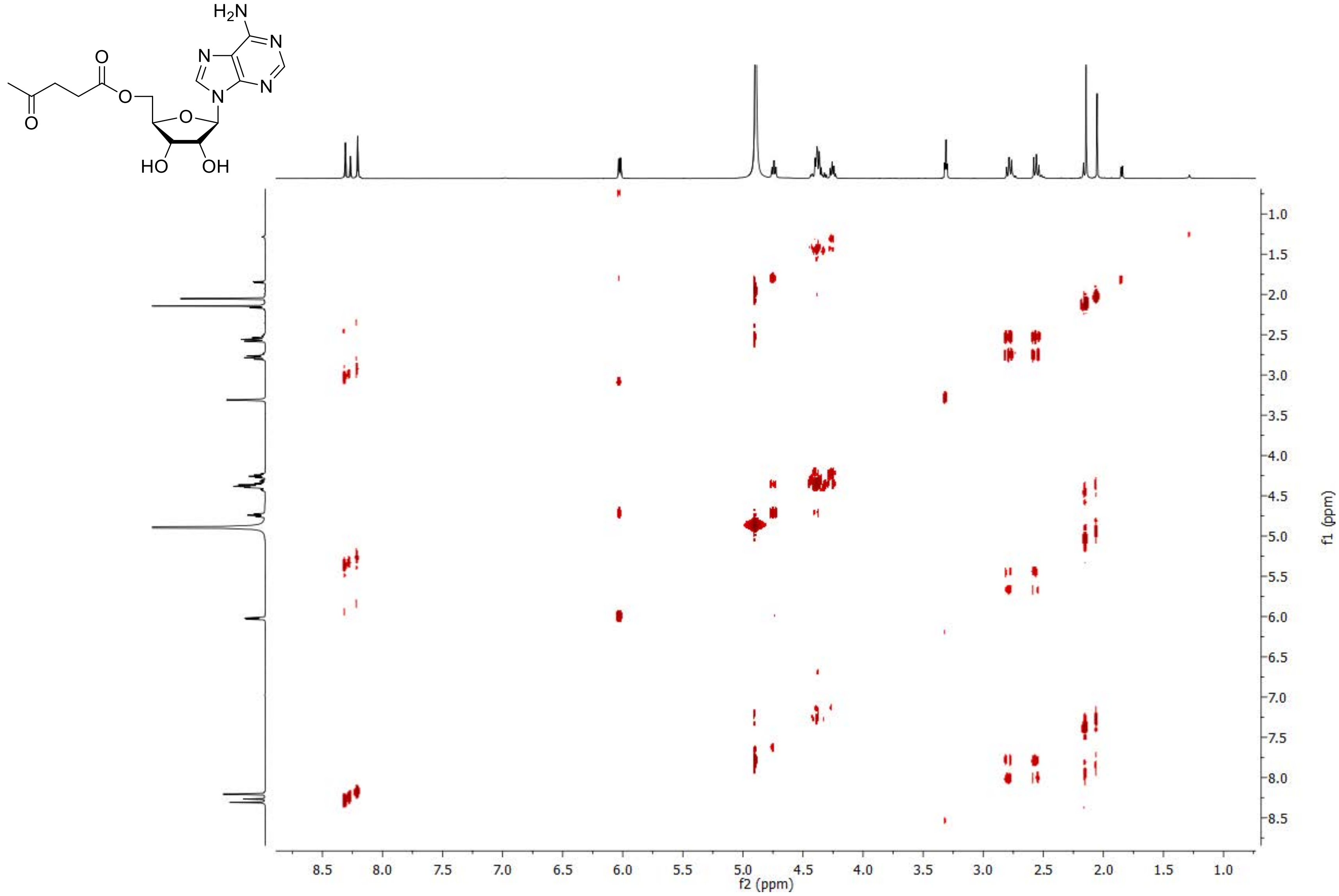

5’-*O*-Levulinyl-β-D-adenosine (2e)

HSQC NMR (MeOD-d_4)

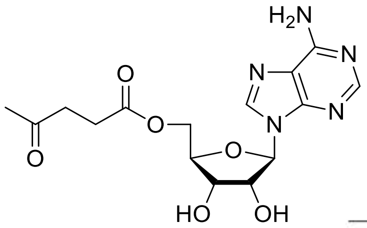

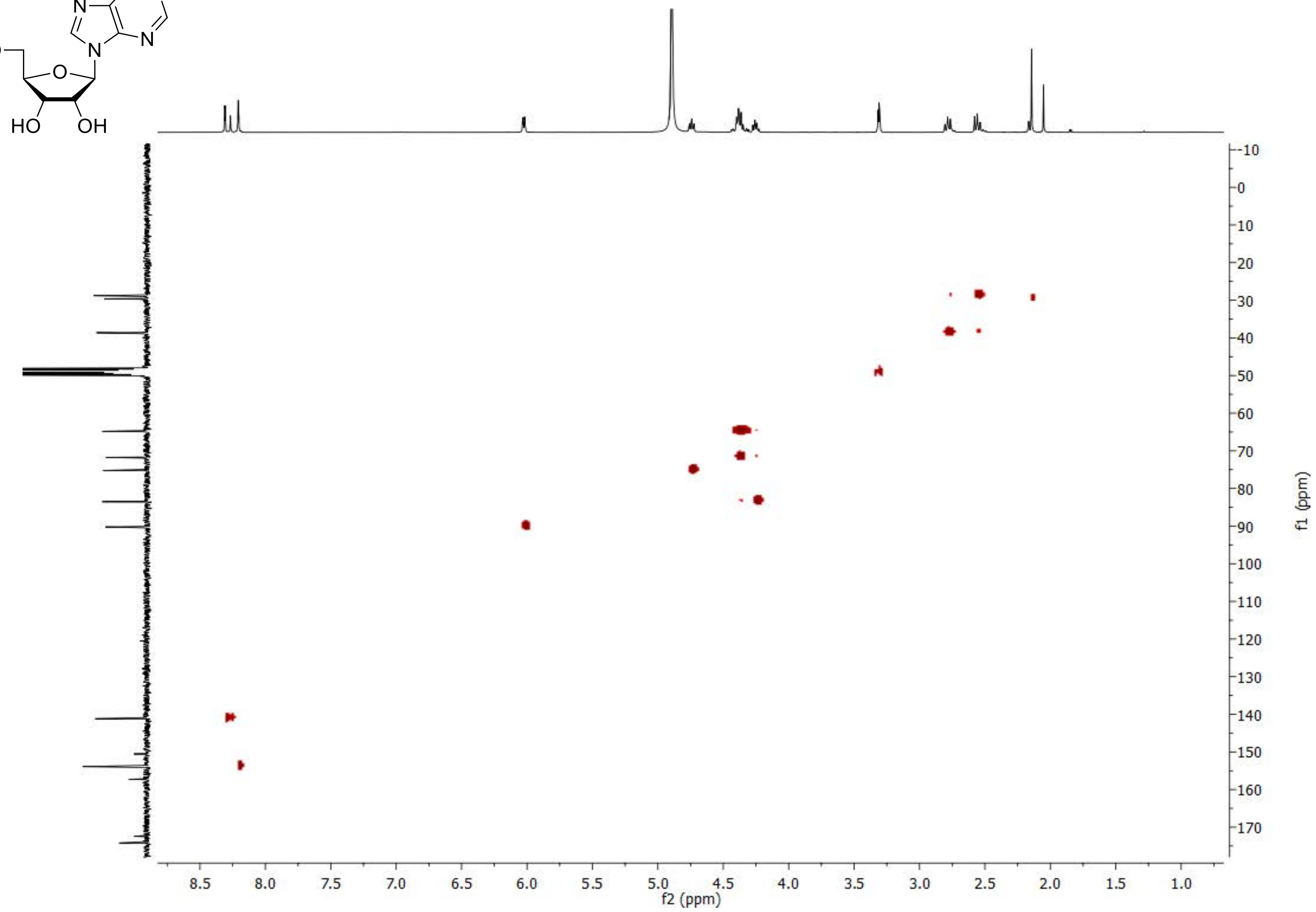

5’-*O*-Levulinyl-β-D-adenosine (2e)

HMBC NMR (MeOD-d_4)

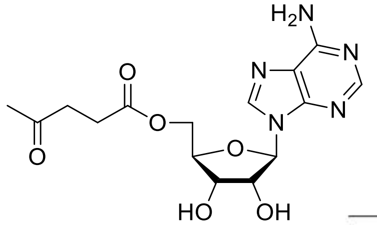

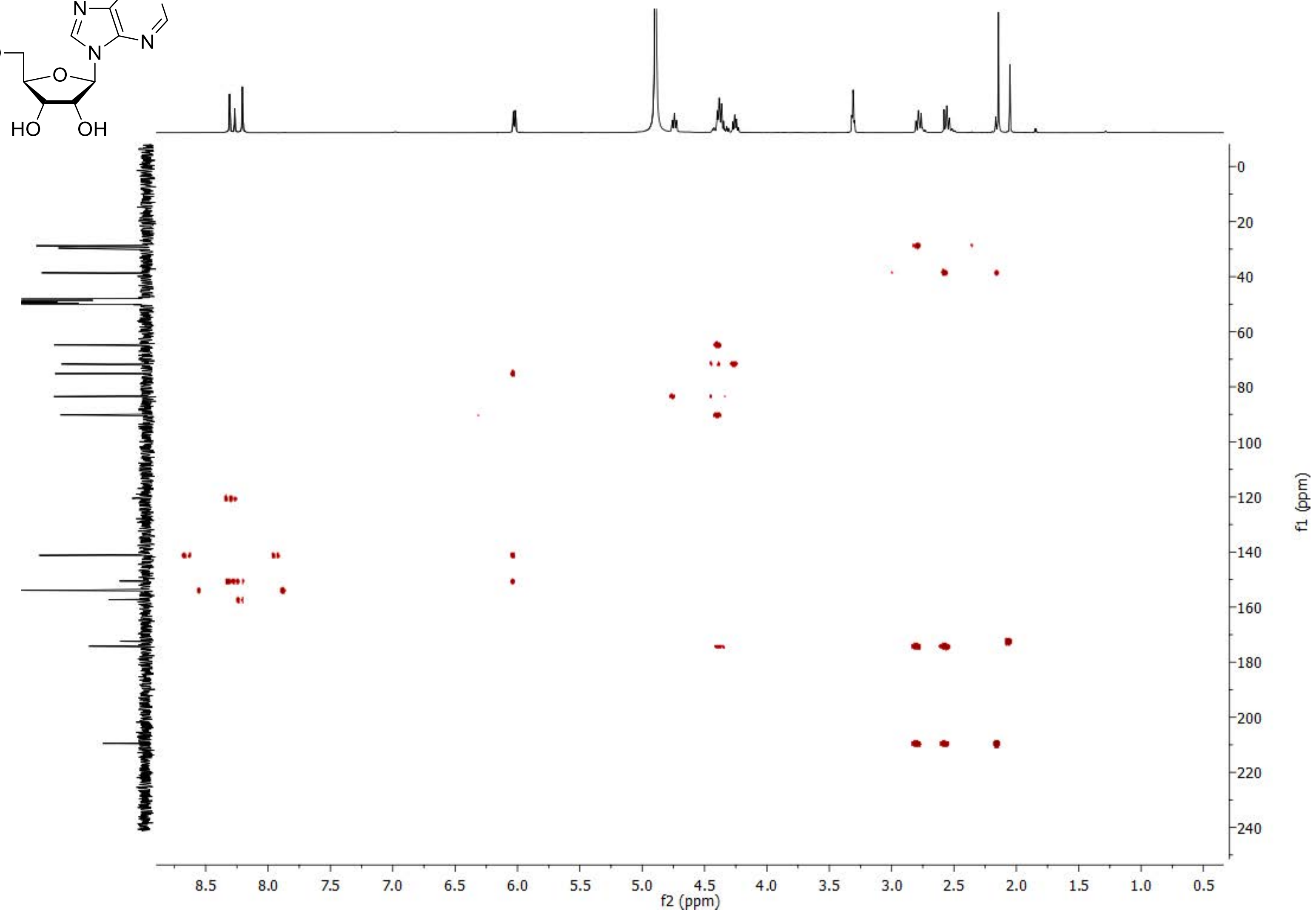

5’-*O*-Levulinyl-β-D-inosine (2f)

^{1}H-NMR (300.13 MHz, MeOD-d_4)

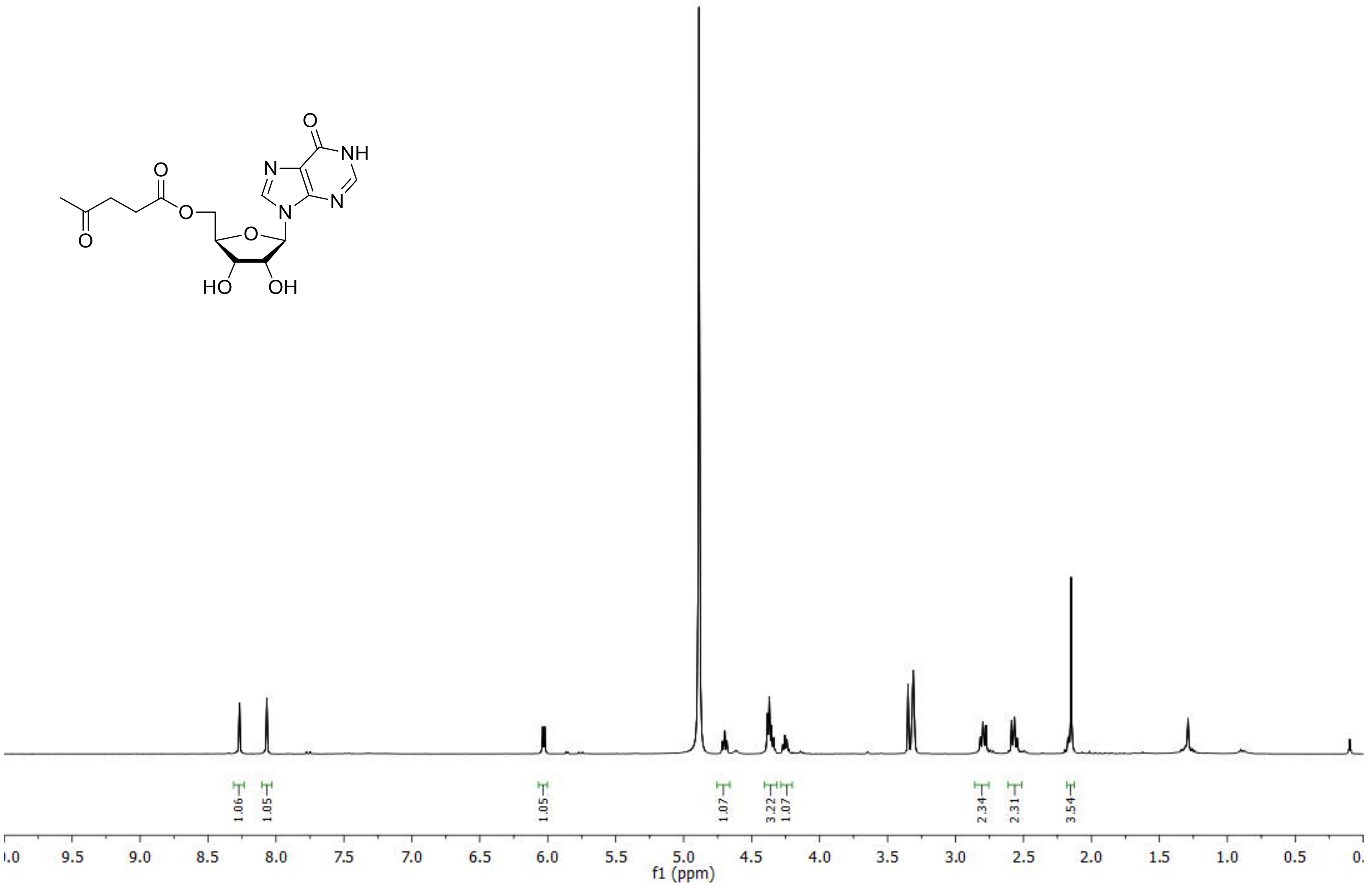

^{13}C-NMR (75.5 MHz, MeOD-d_4)

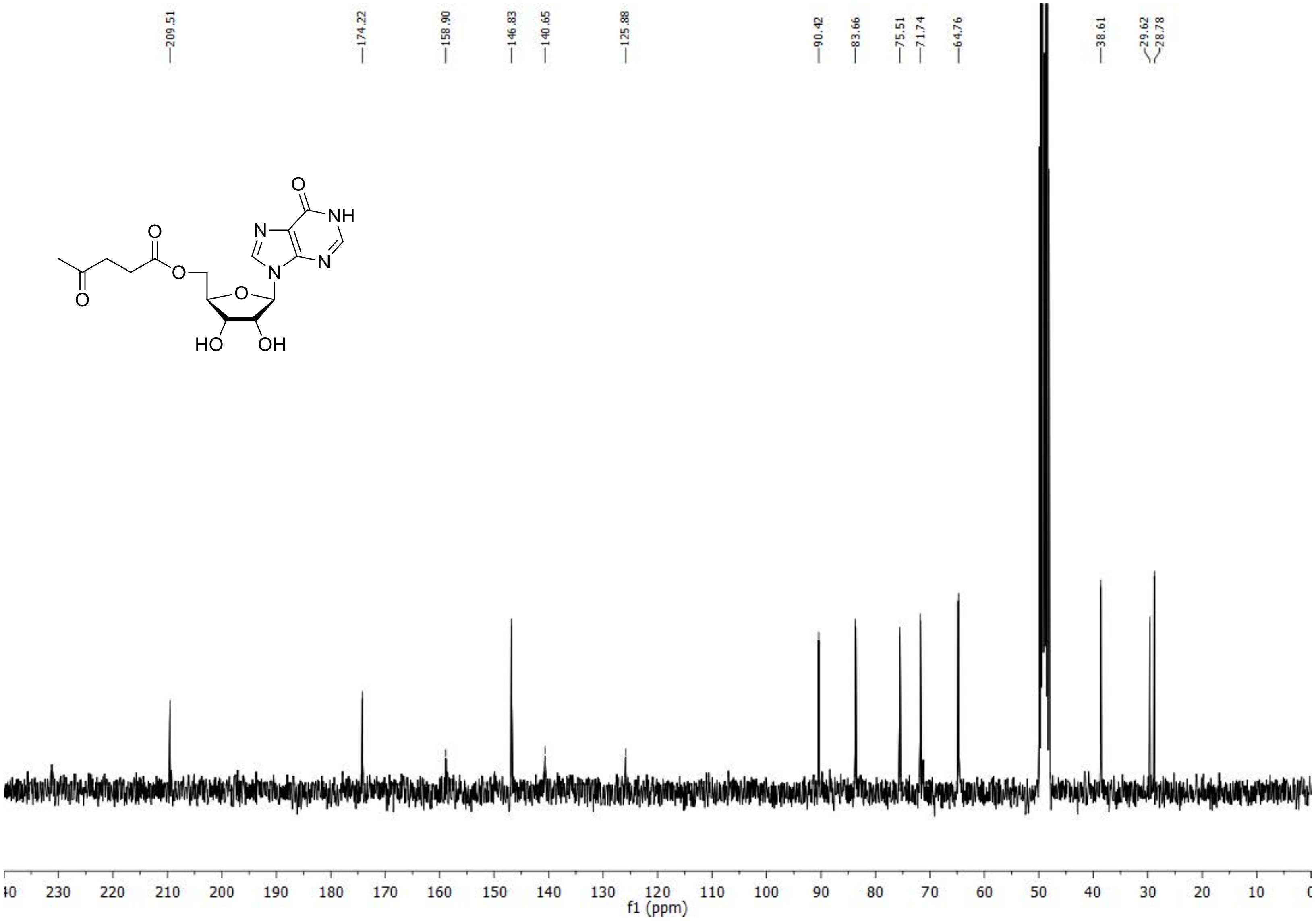

5'-*O*-Levulinyl-β-D-inosine (2f)

DEPT NMR (75.5 MHz, MeOD-d_4)

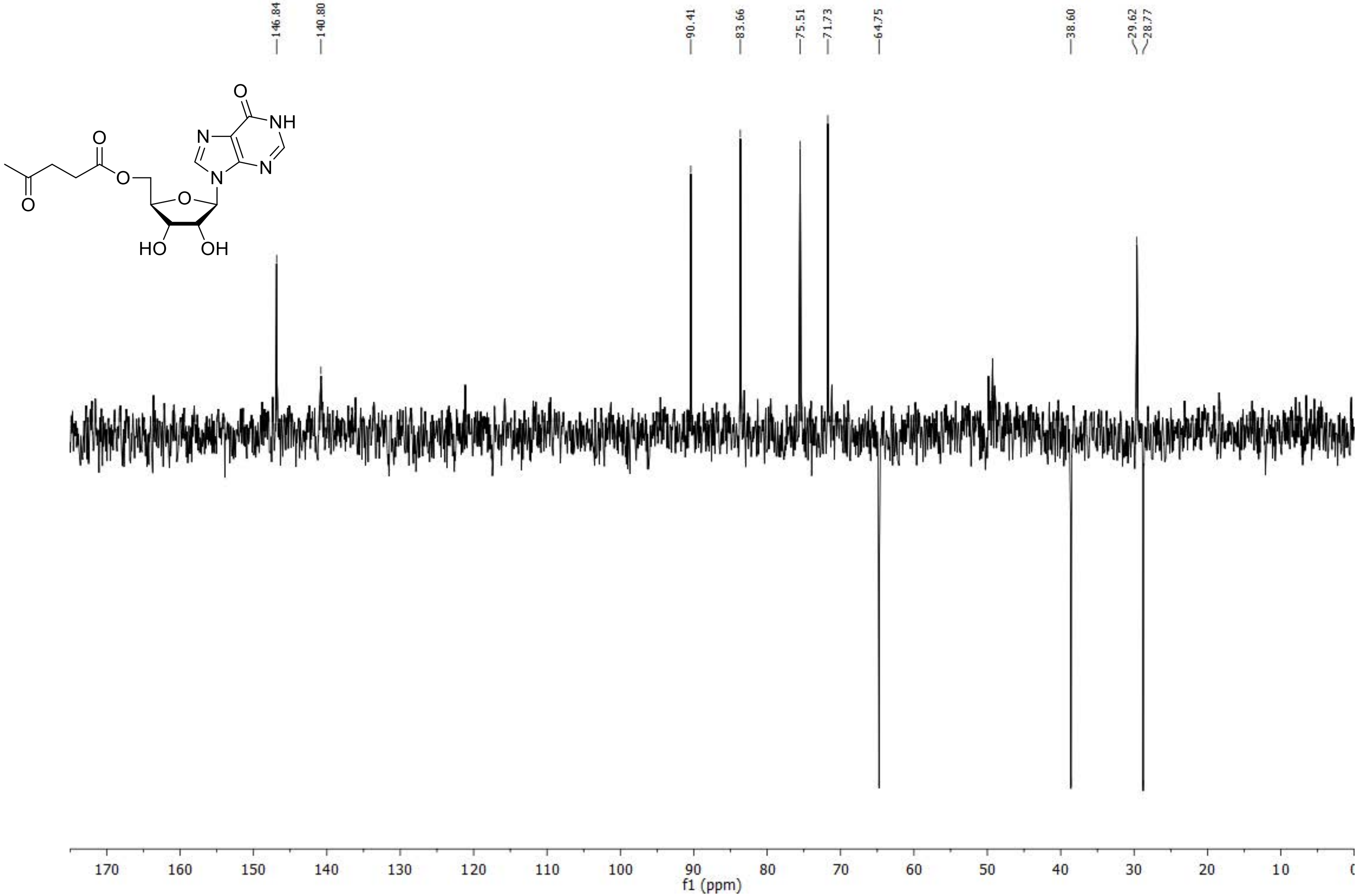

5’-*O*-Levulinyl-β-D-inosine (2f)

COSY NMR (MeOD-d_4)

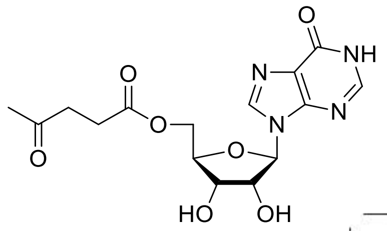

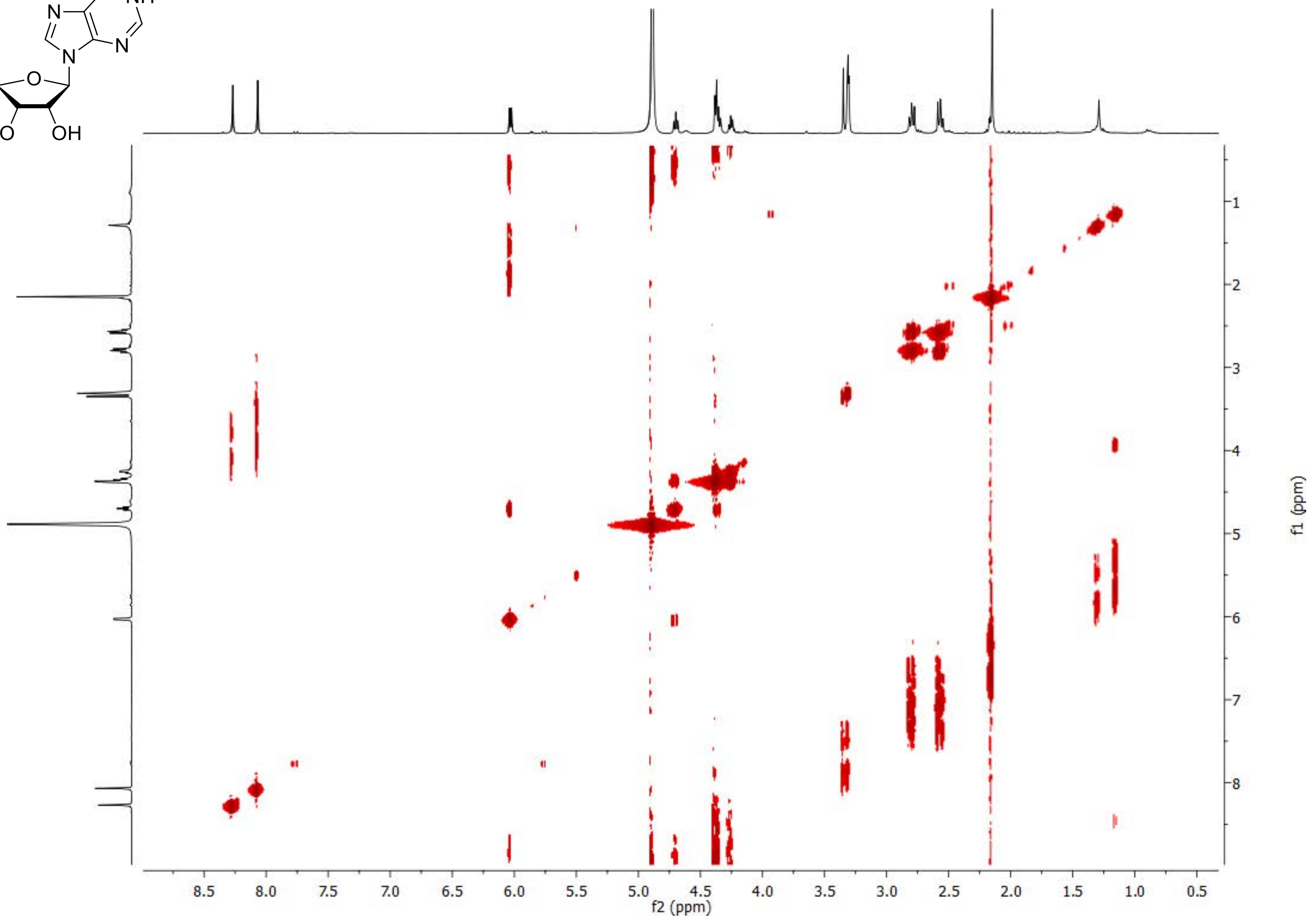

5'-*O*-Levulinyl-β-D-inosine (2f)

HSQC NMR (MeOD-d_4)

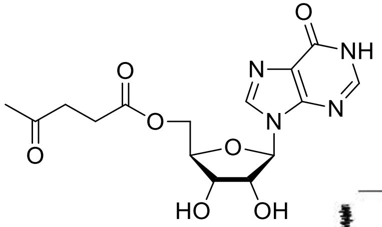

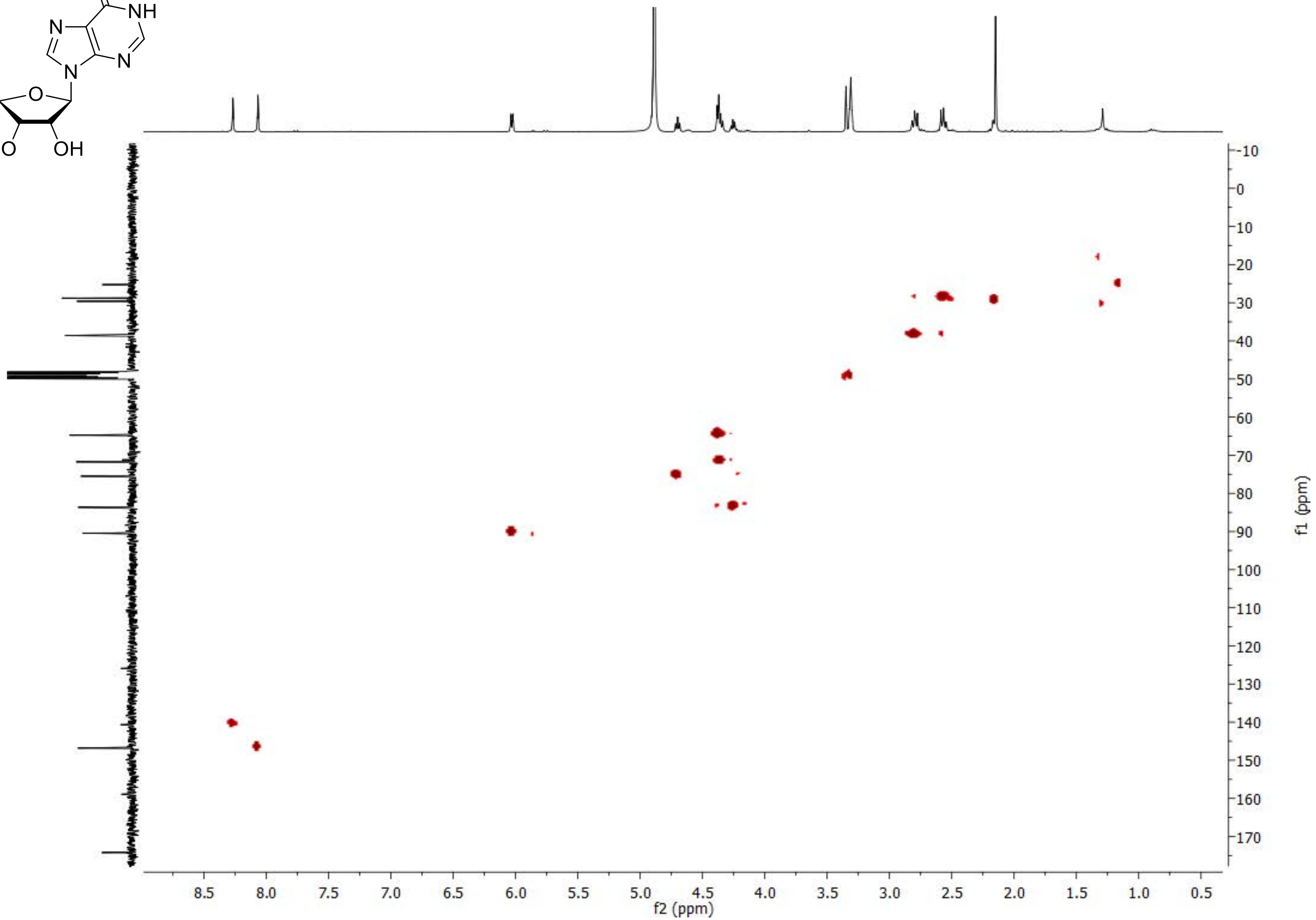

5’-*O*-Levulinyl-β-D-inosine (2f)

HMBC NMR (MeOD-d_4)

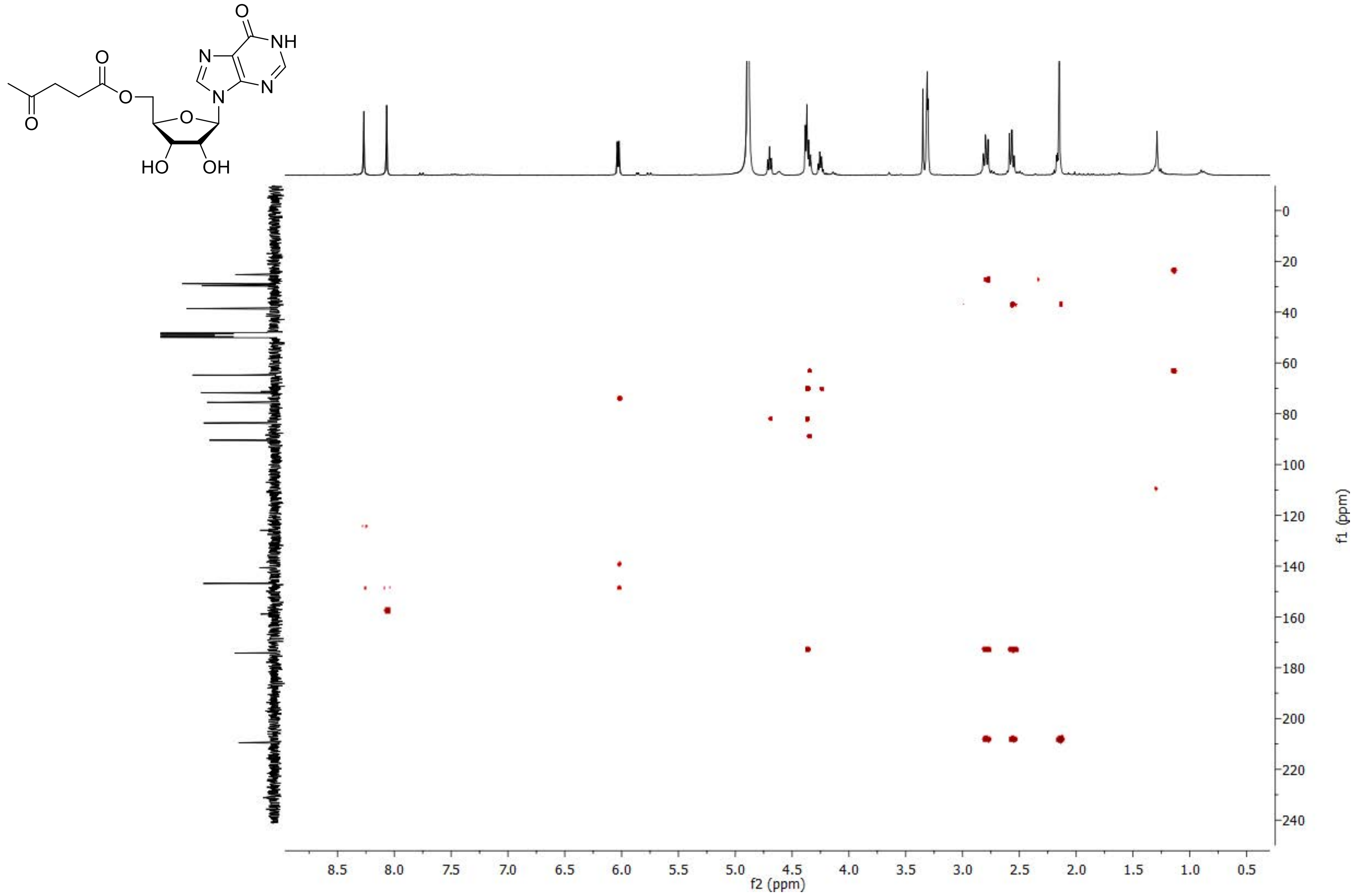

5’-*O*-(*tert*-Butyldimethylsilyl)-β-D-uridine (5a)

^{1}H-NMR (300.13 MHz, $CDCl_3$)

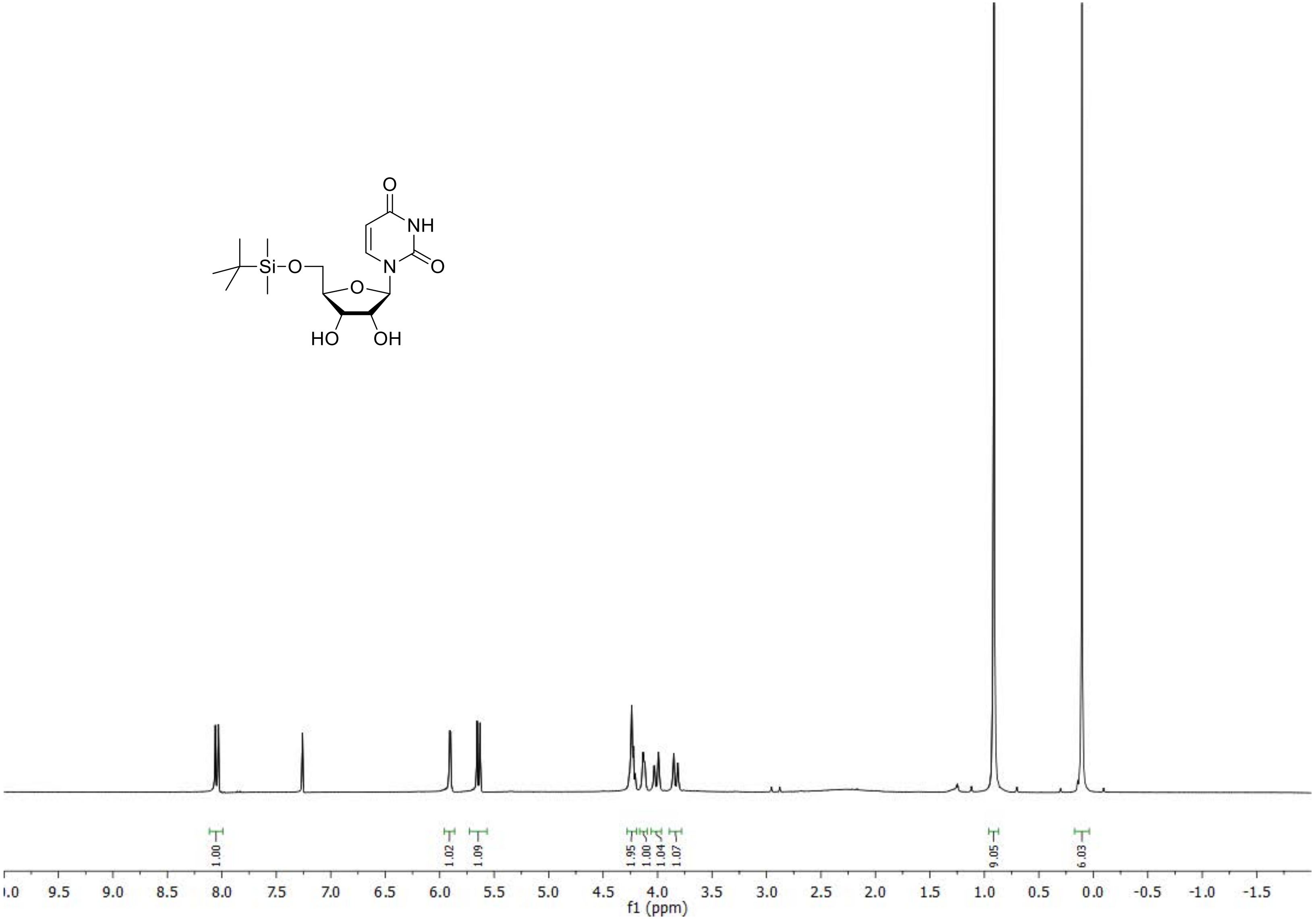

5'-*O*-(*tert*-Butyldimethylsilyl)-β-D-uridine (5a)

^{13}C-NMR (300.13 MHz, $CDCl_3$)

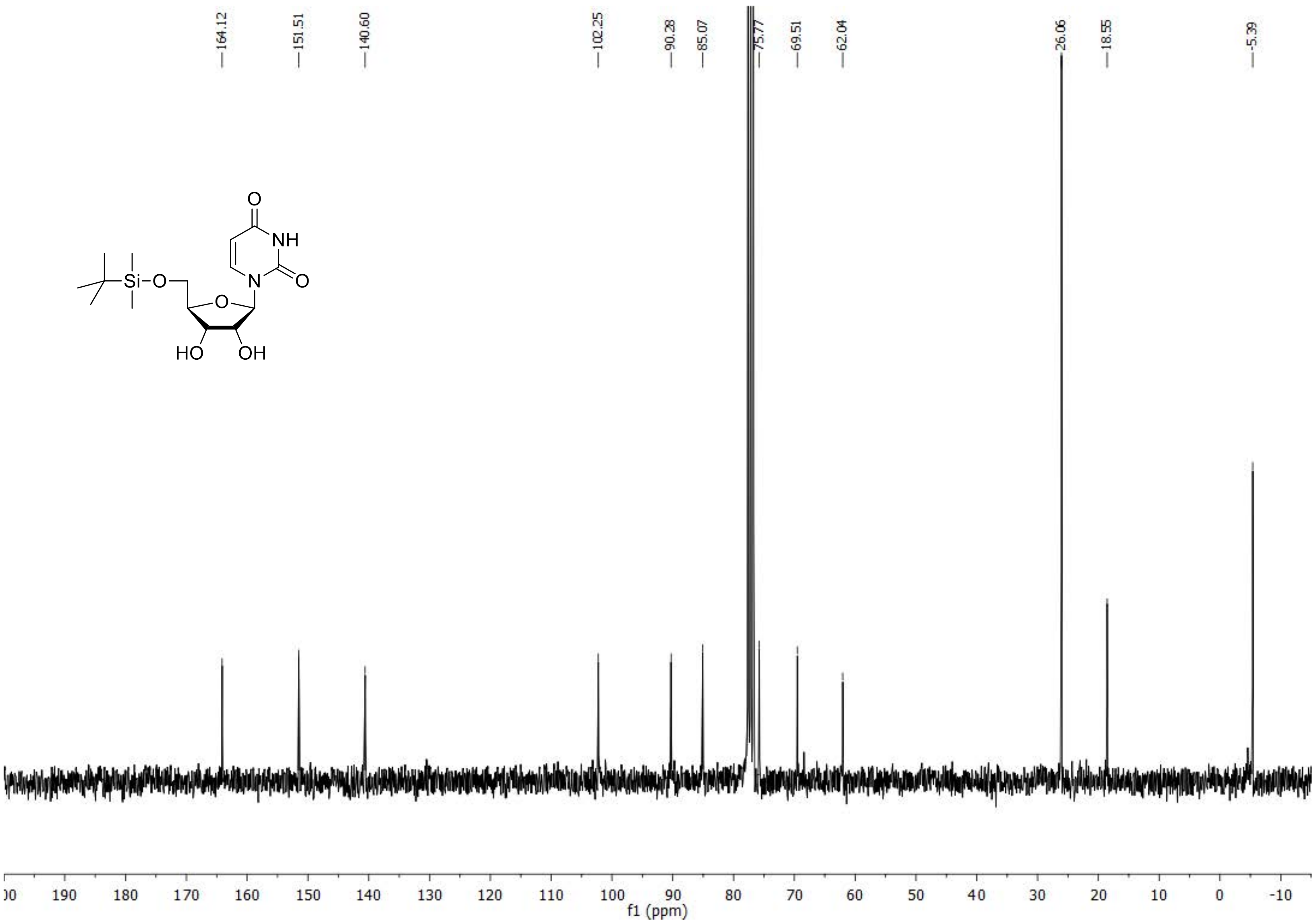

5'-*O*-(*tert*-Butyldimethylsilyl)-β-D-5-methyluridine (5b)

^{1}H-NMR (300.13 MHz, $CDCl_3$)

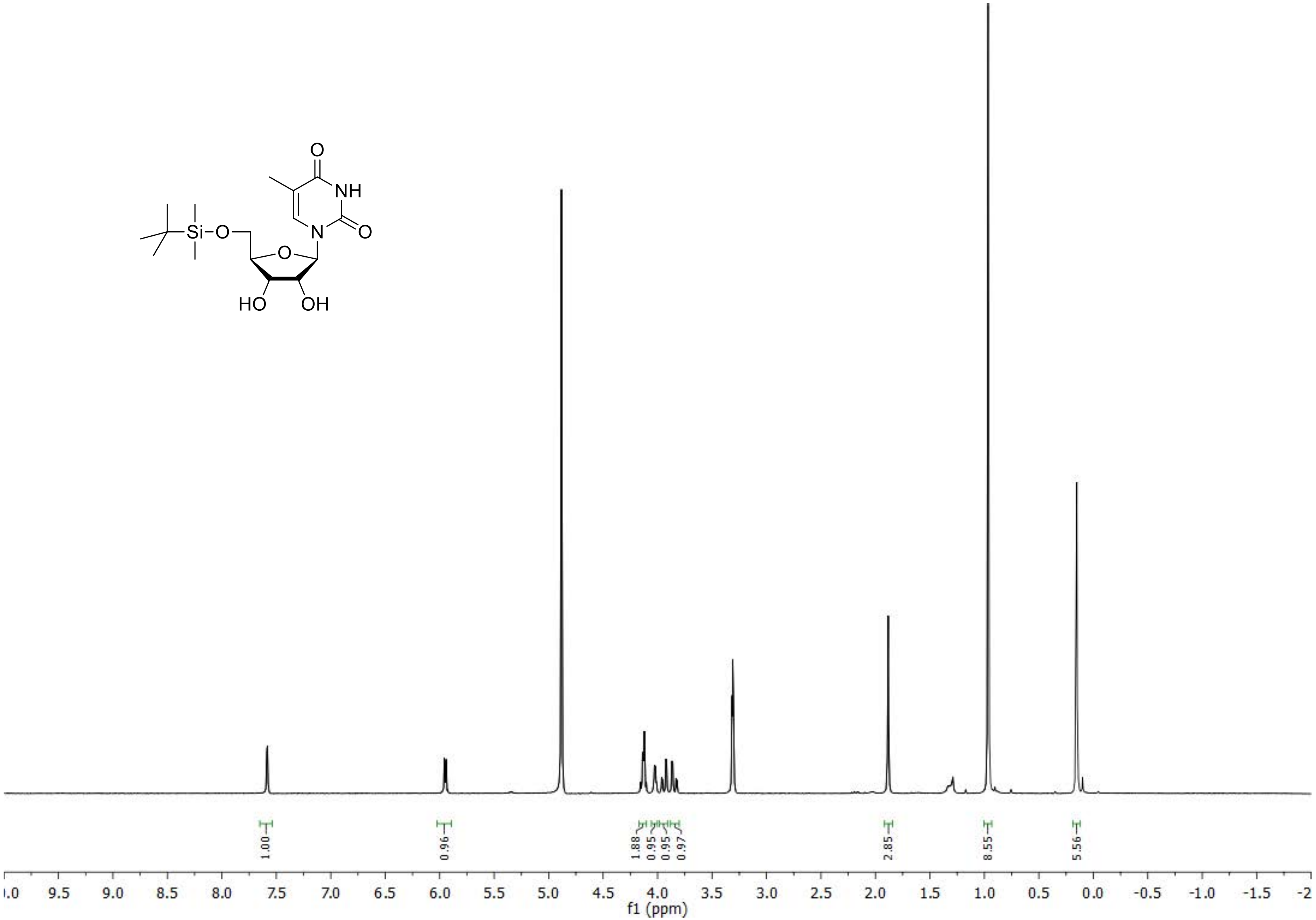

5'-*O*-(*tert*-Butyldimethylsilyl)-β-D-5-methyluridine (5b)

^{13}C-NMR (75.5 MHz, $CDCl_3$)

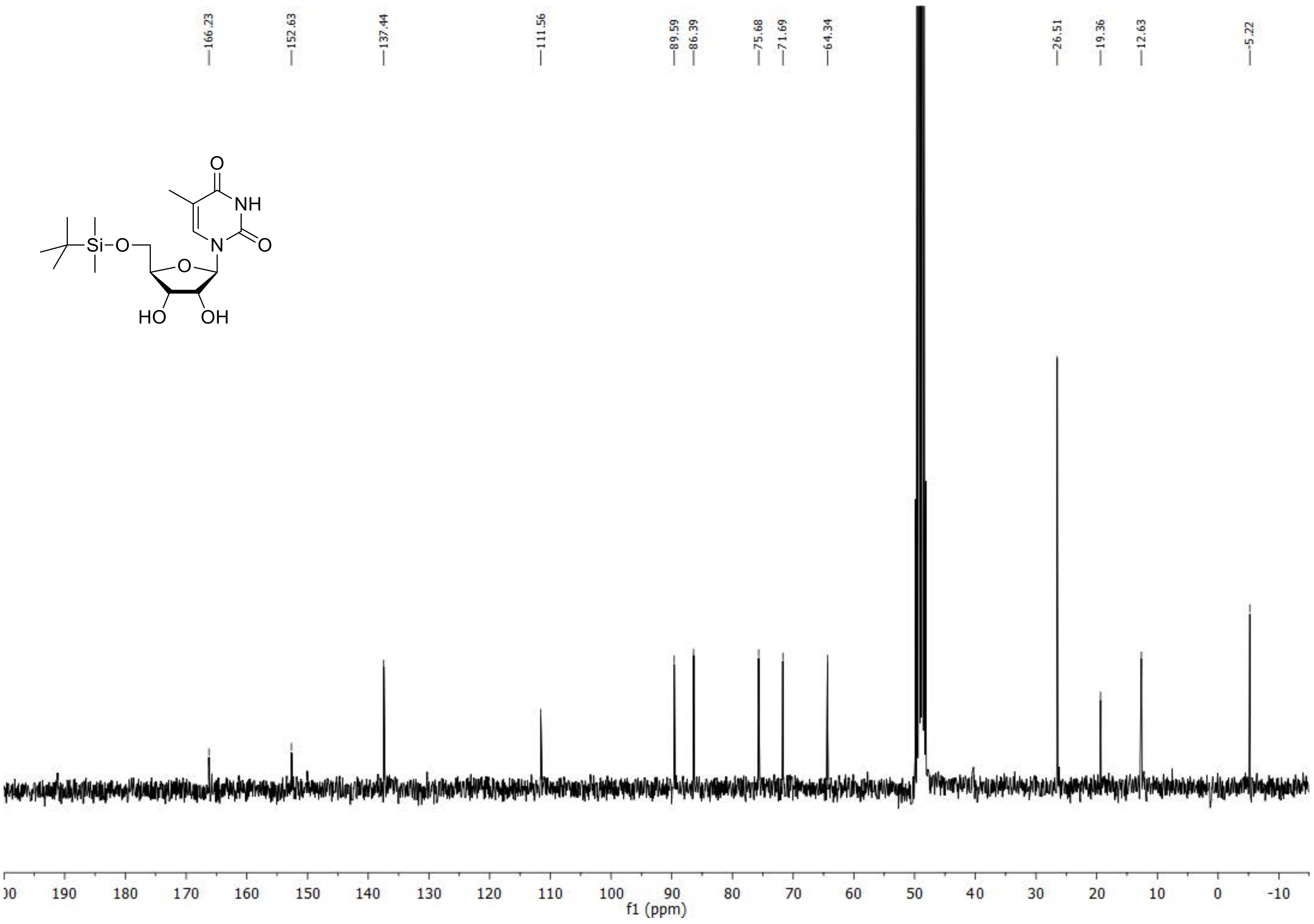

5’-*O*-(*tert*-Butyldimethylsilyl)-β-D-cytidine (5c)

^{1}H-NMR (300.13 MHz, MeOH-d_4)

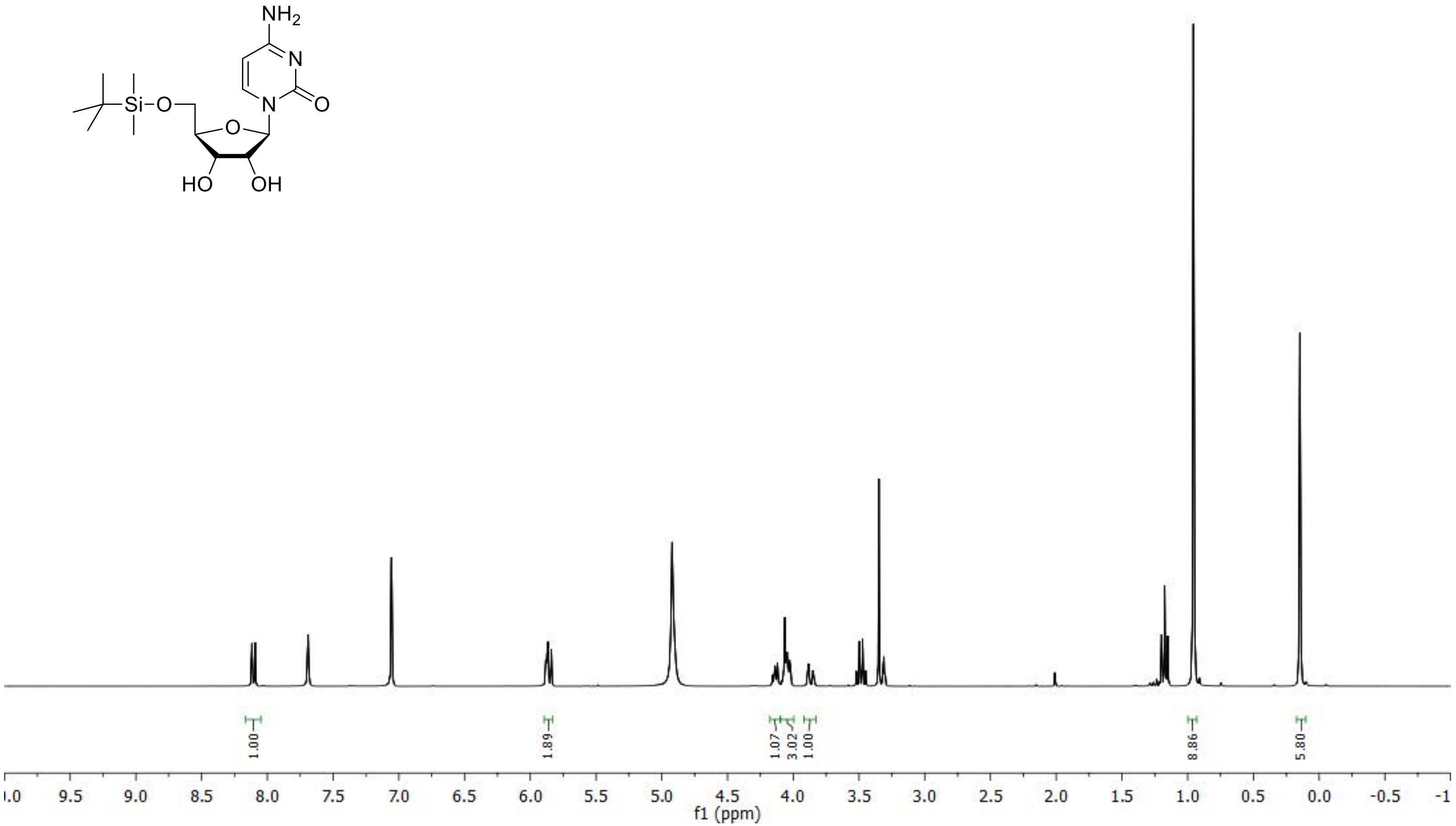

5’-*O*-(*tert*-Butyldimethylsilyl)-β-D-cytidine (5c)

^{13}C-NMR (75.5 MHz, MeOH-d_4)

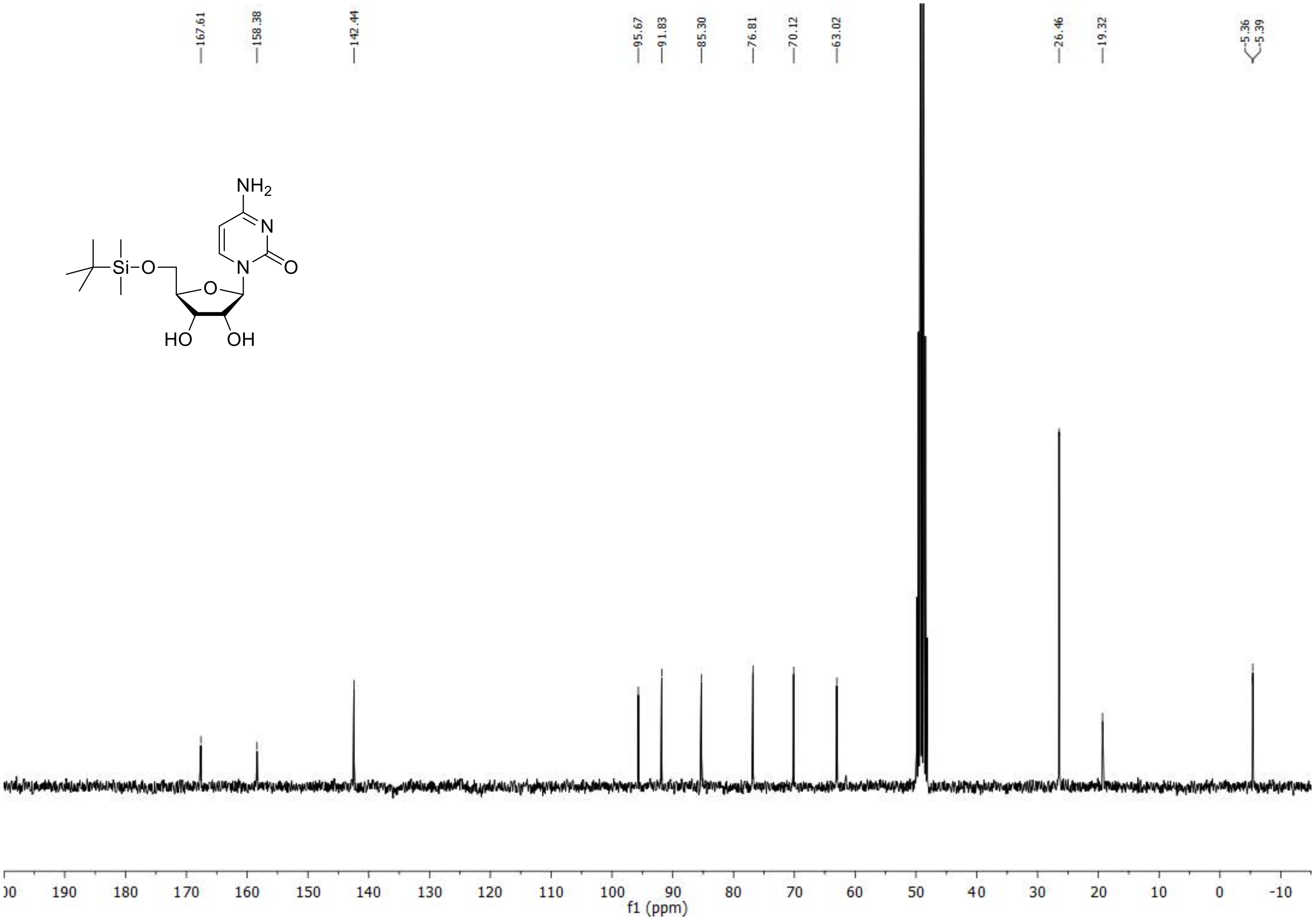

N^4-Benzoyl-5'-*O*-(*tert*-butyldimethylsilyl)-β-D-cytidine (5d)

^{1}H-NMR (300.13 MHz, MeOH-d_4)

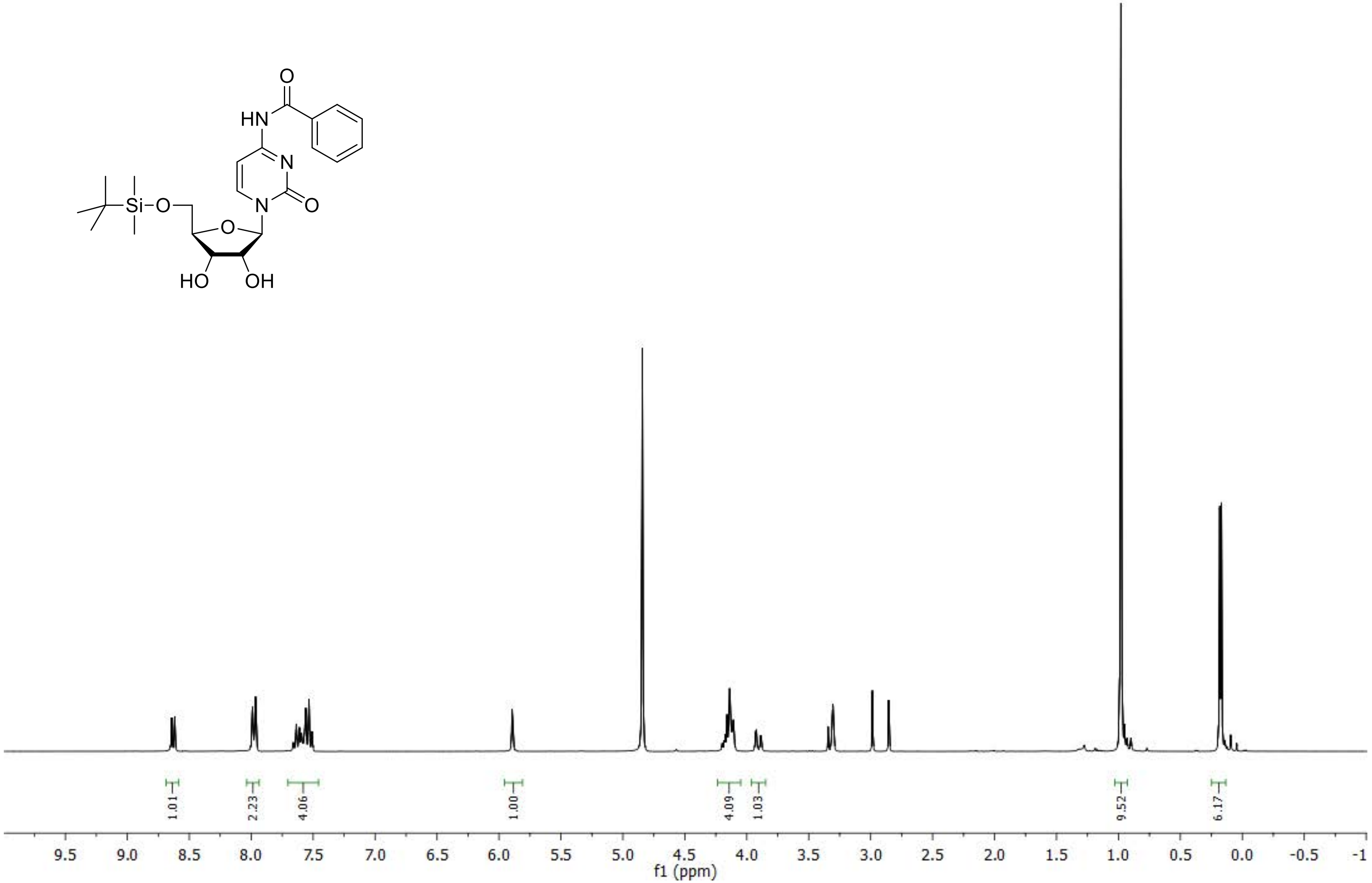

N[4]-Benzoyl-5'-*O*-(*tert*-butyldimethylsilyl)-β-D-cytidine (5d)

^{13}C-NMR (75.5 MHz, MeOH-d_4)

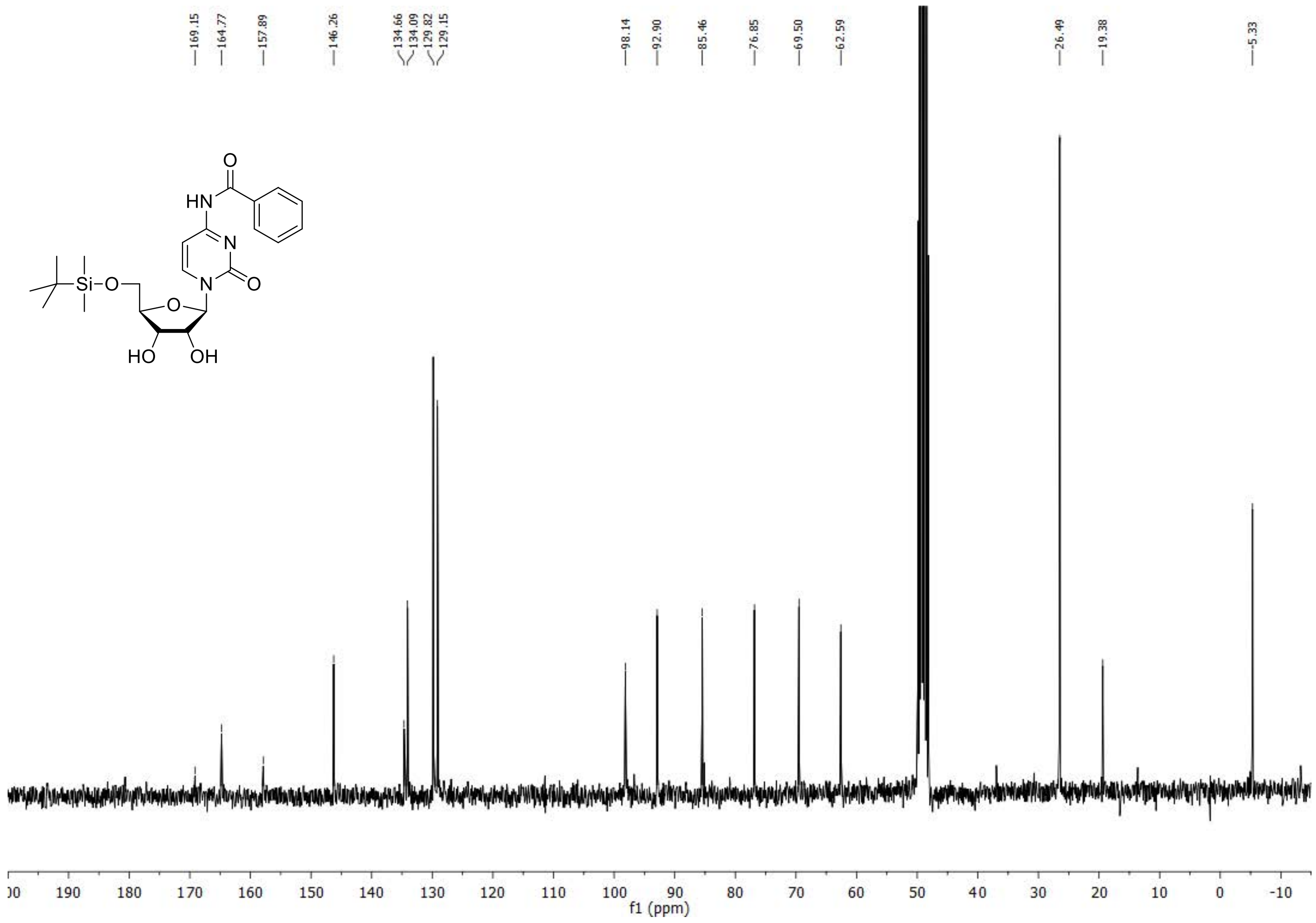

N^4-Benzoyl-5'-*O*-(*tert*-butyldimethylsilyl)-β-D-cytidine (5d)

DEPT NMR (75.5 MHz, MeOH-d_4)

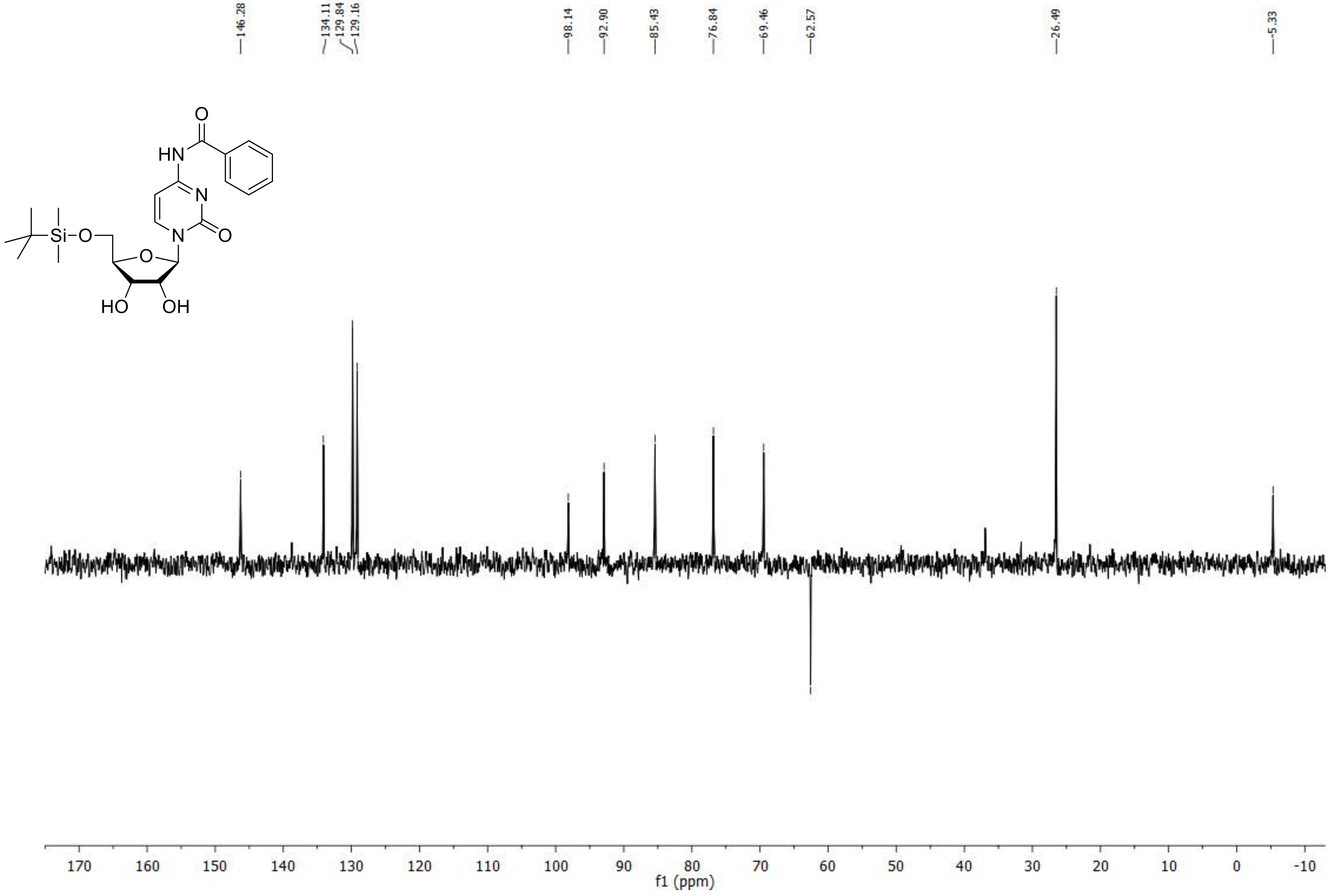

N^4-Benzoyl-5'-*O*-(*tert*-butyldimethylsilyl)-β-D-cytidine (5d)

HSQC NMR (MeOH-d_4)

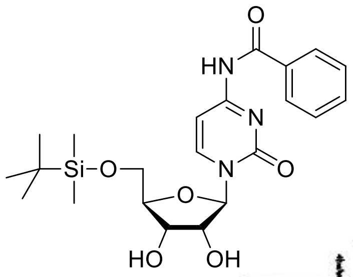

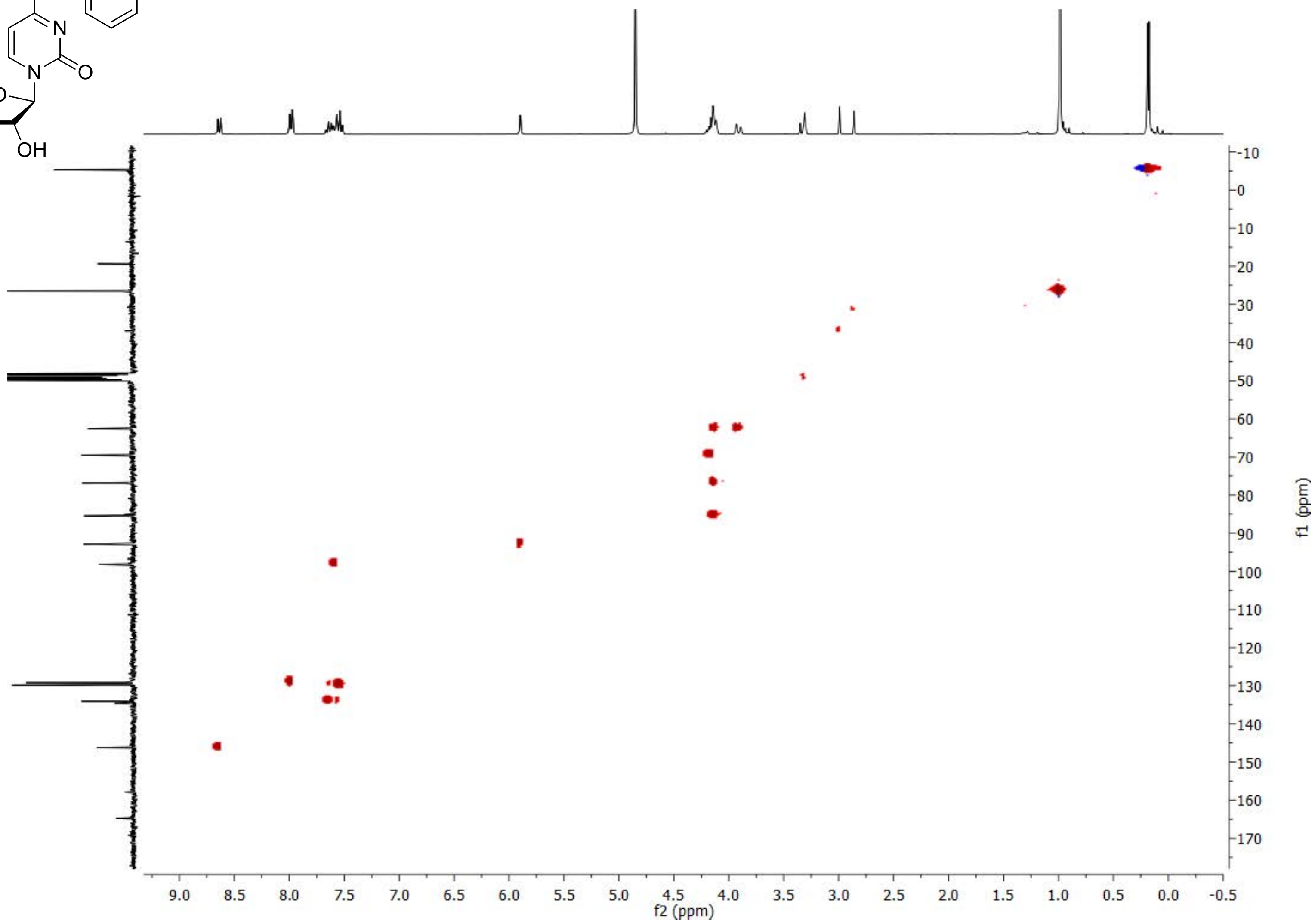

5’-*O*-(*tert*-Butyldimethylsilyl)-β-D-adenosine (5e)

^{1}H-NMR (300.13 MHz, MeOH-d_4)

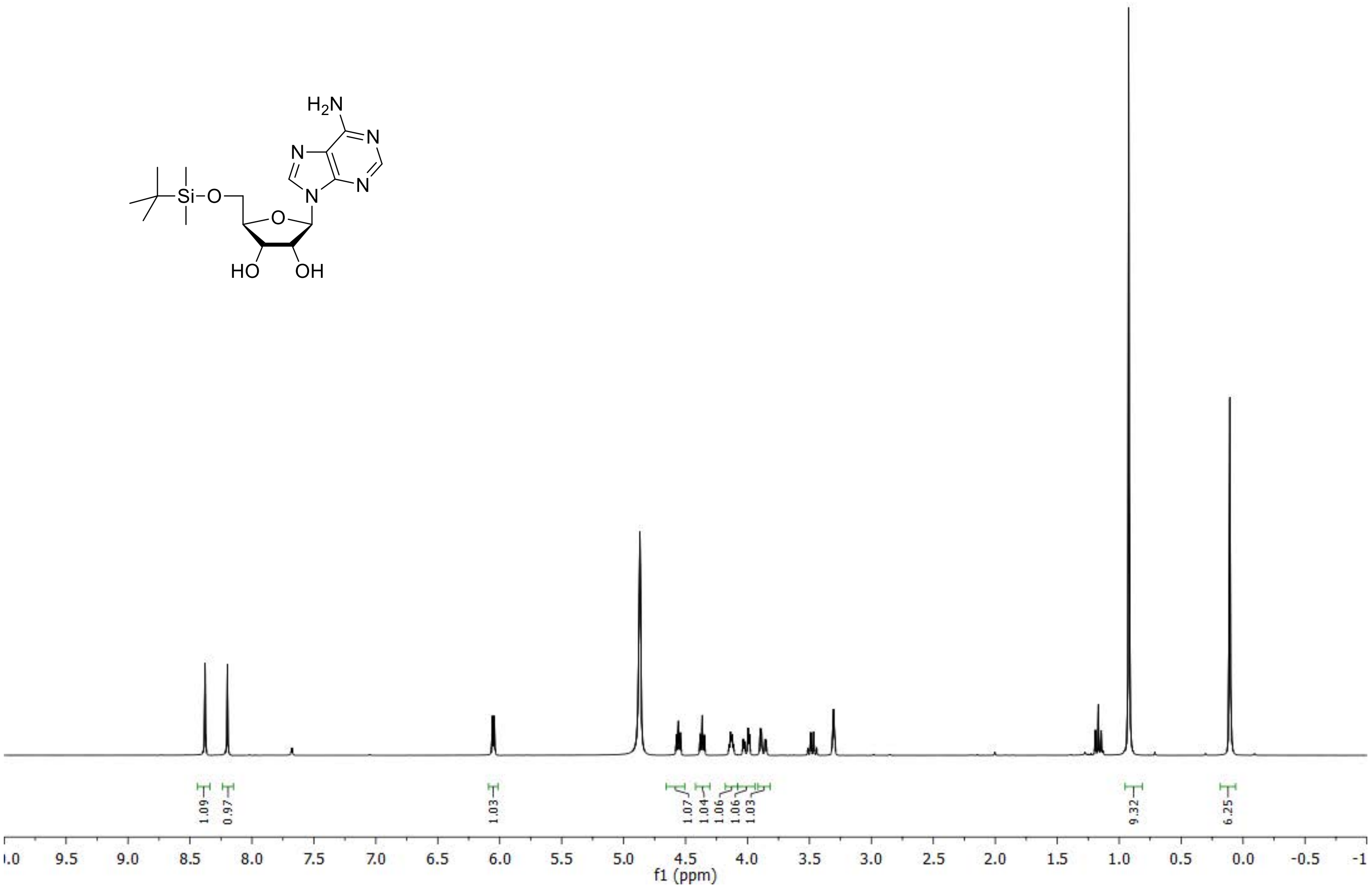

5’-*O*-(*tert*-Butyldimethylsilyl)-β-D-adenosine (5e)

^{13}C-NMR (75.5 MHz, MeOH-d_4)

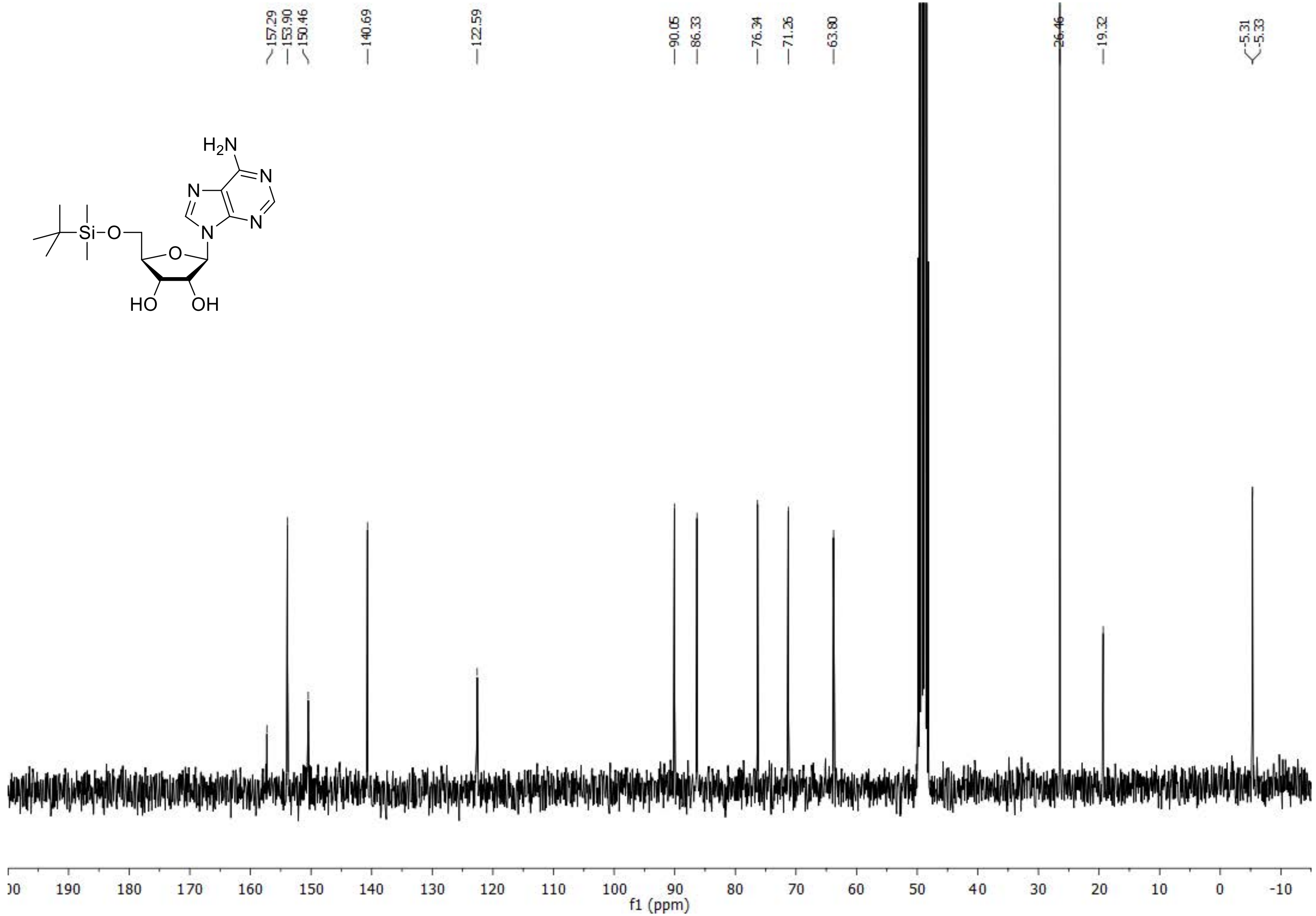

5'-*O*-(*tert*-Butyldimethylsilyl)-β-D-inosine (5f)

^{1}H-NMR (300.13 MHz, MeOH-d_4)

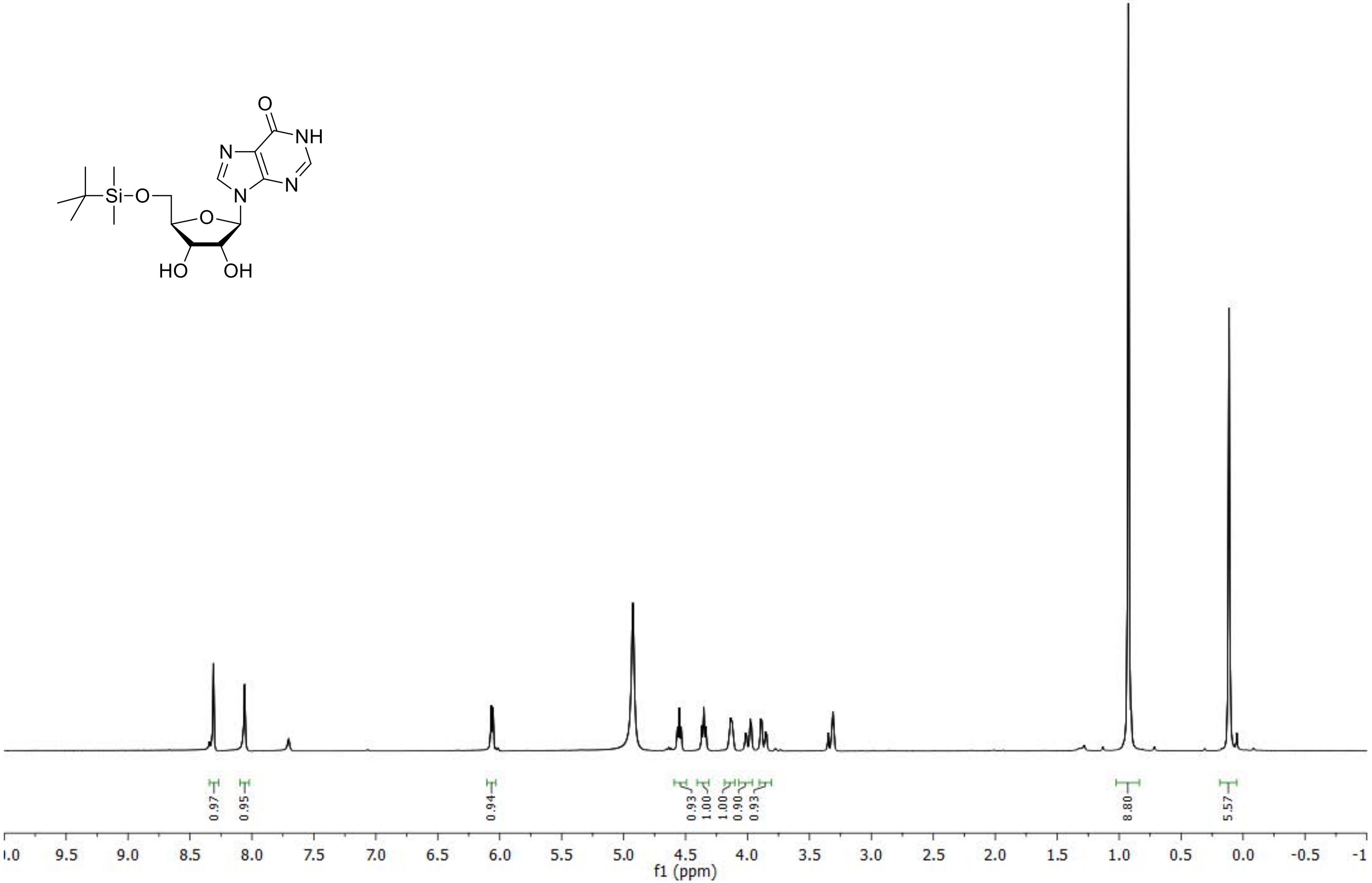

5’-*O*-(*tert*-Butyldimethylsilyl)-β-D-inosine (5f)

^{13}C-NMR (75.5 MHz, MeOH-d_4)

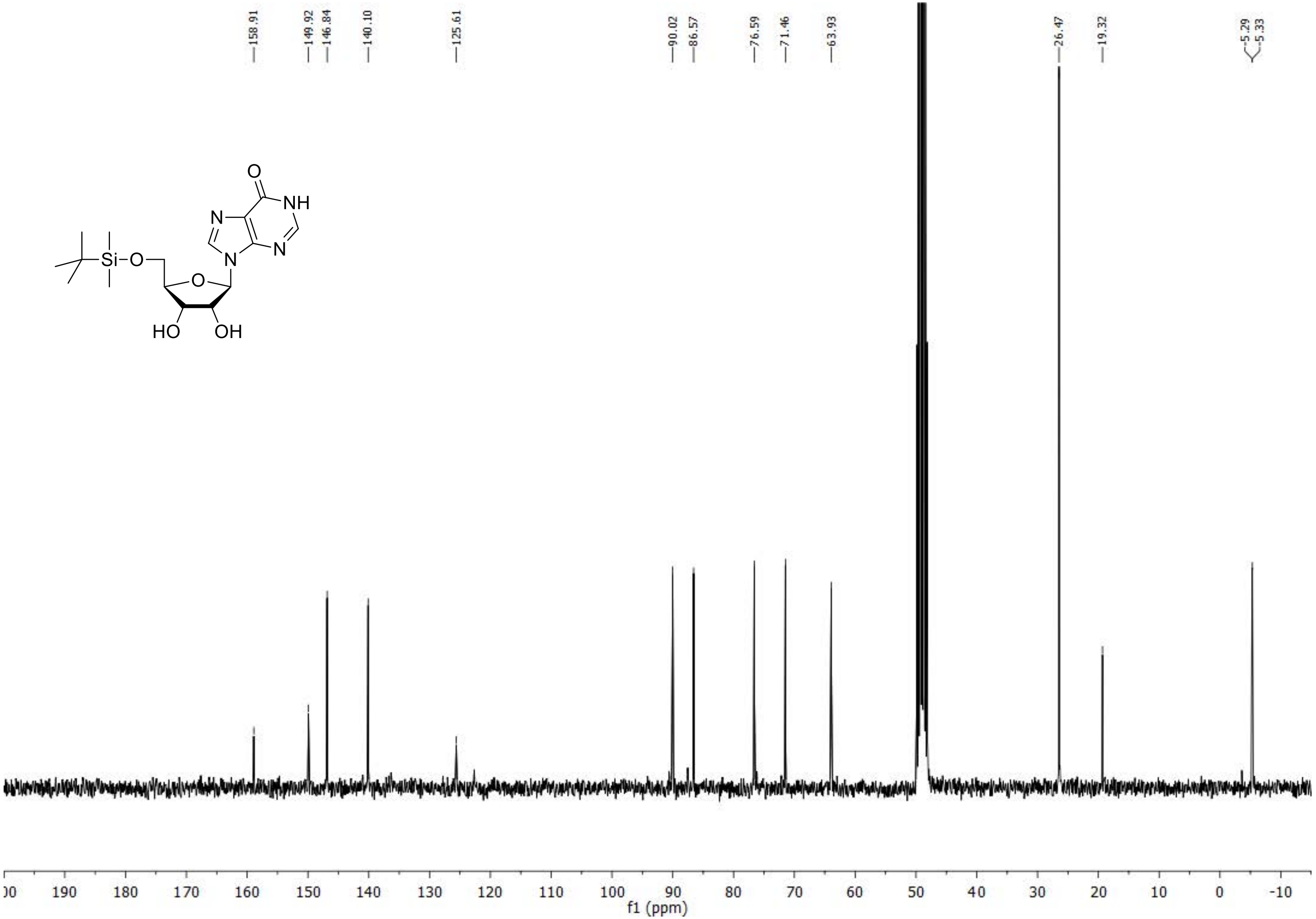

5’-*O*-(*tert*-Butyldimethylsilyl)-β-D-inosine (5f)

DEPT NMR (75.5 MHz, MeOH-d_4)

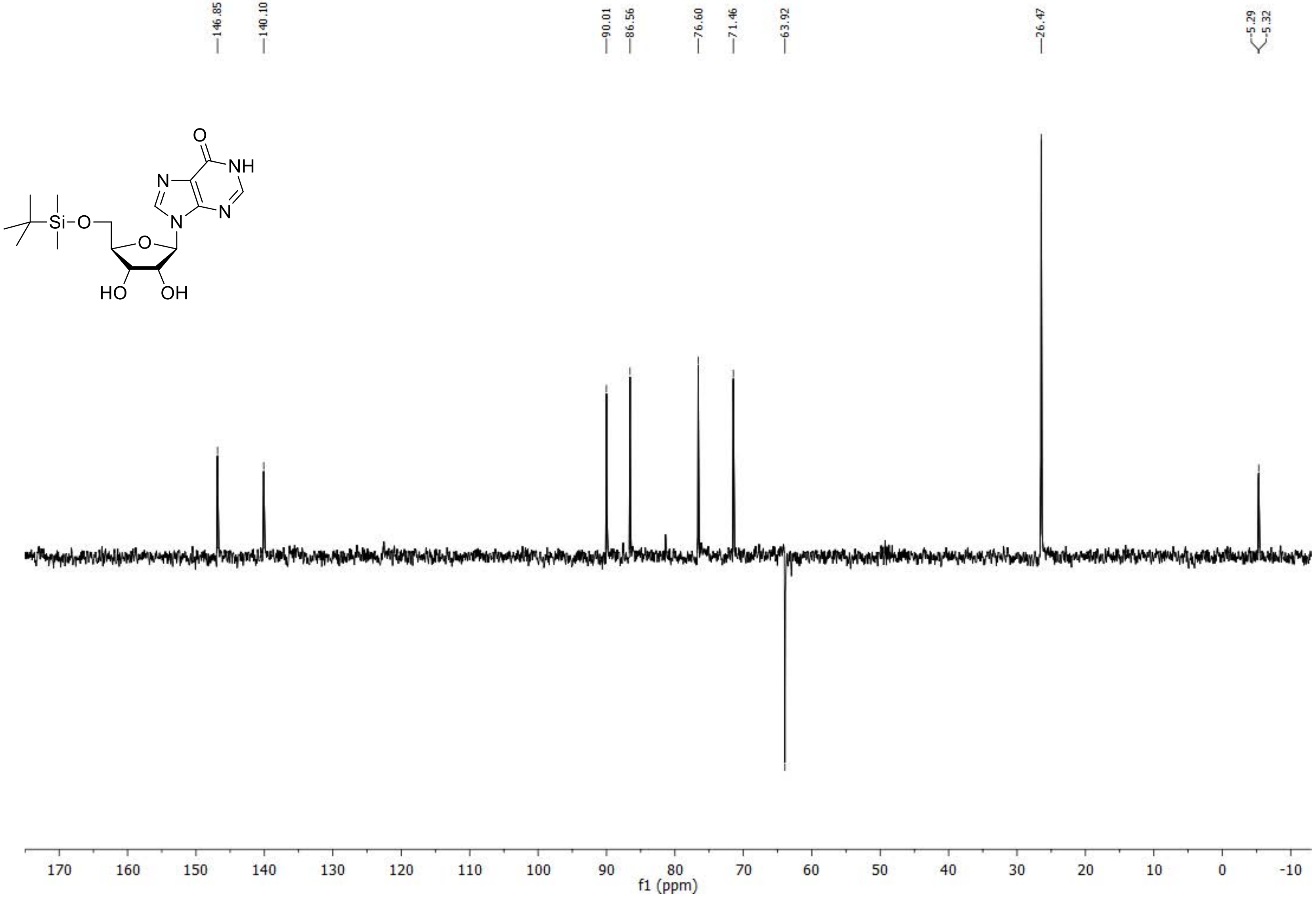

5’-*O*-(*tert*-Butyldimethylsilyl)-β-D-inosine (5f)

COSY NMR (MeOH-d_4)

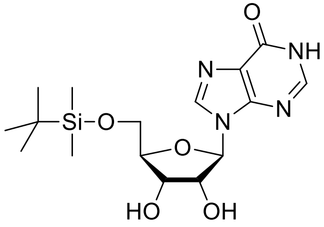

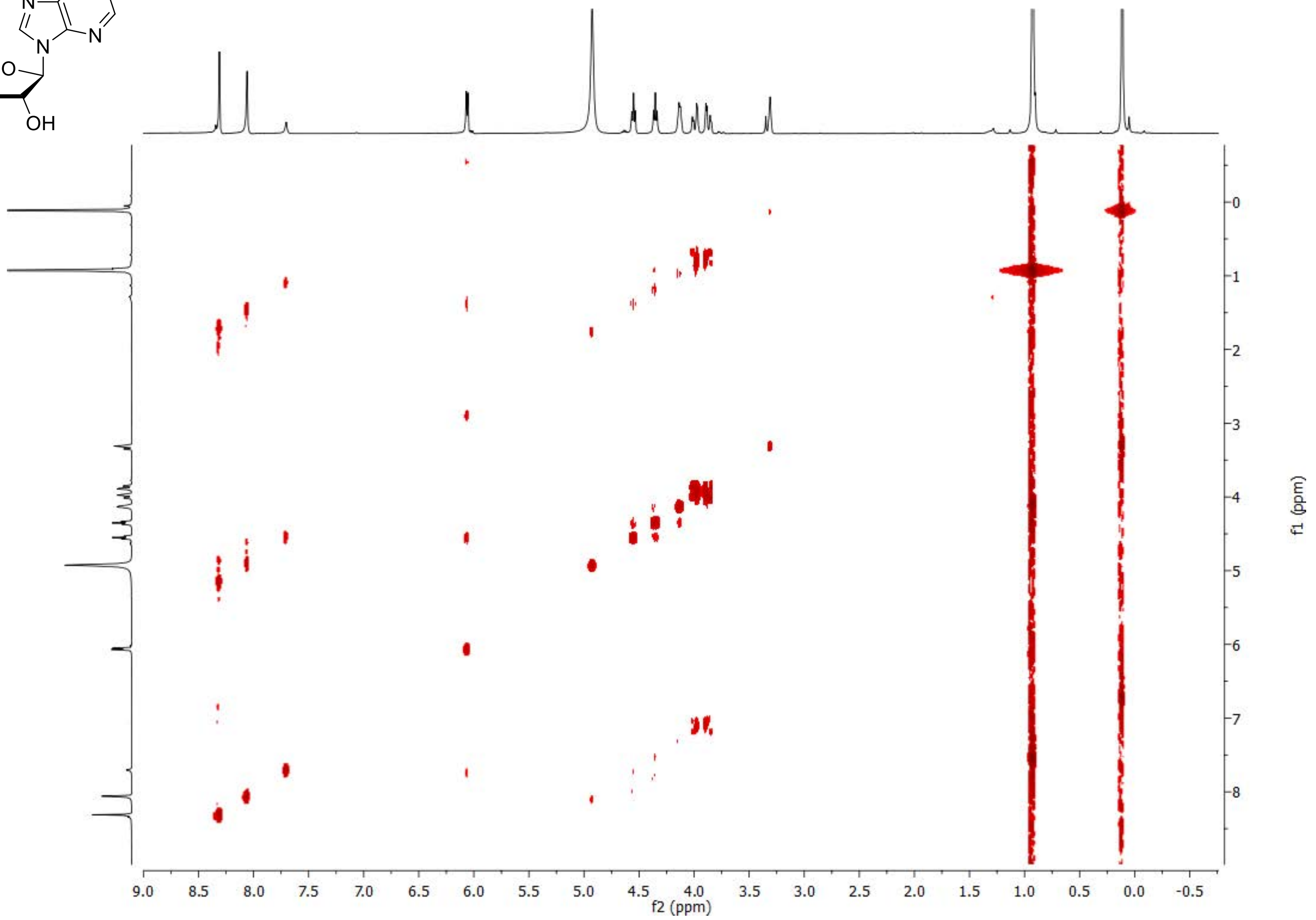

5’-*O*-(*tert*-Butyldimethylsilyl)-β-D-inosine (5f)

HSQC NMR (MeOH-d_4)

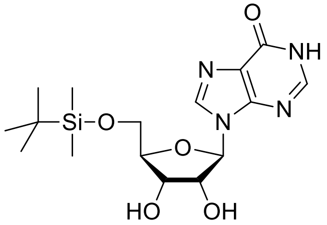

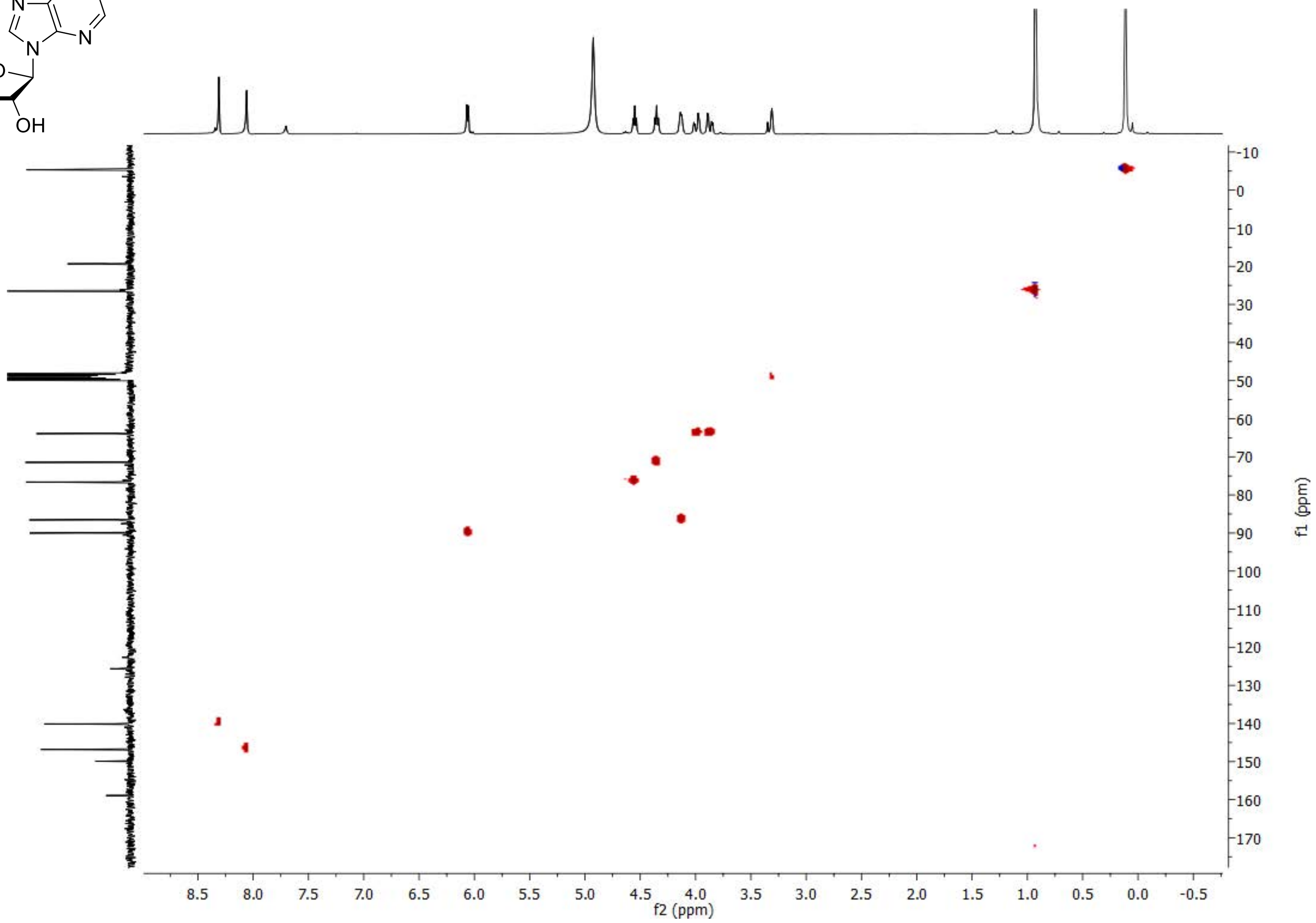

5’-*O*-(*tert*-Butyldimethylsilyl)-β-D-inosine (5f)

HMBC NMR (MeOH-d_4)

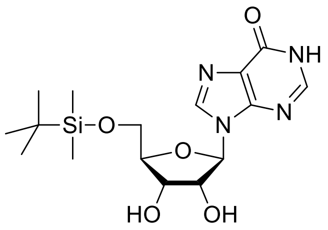

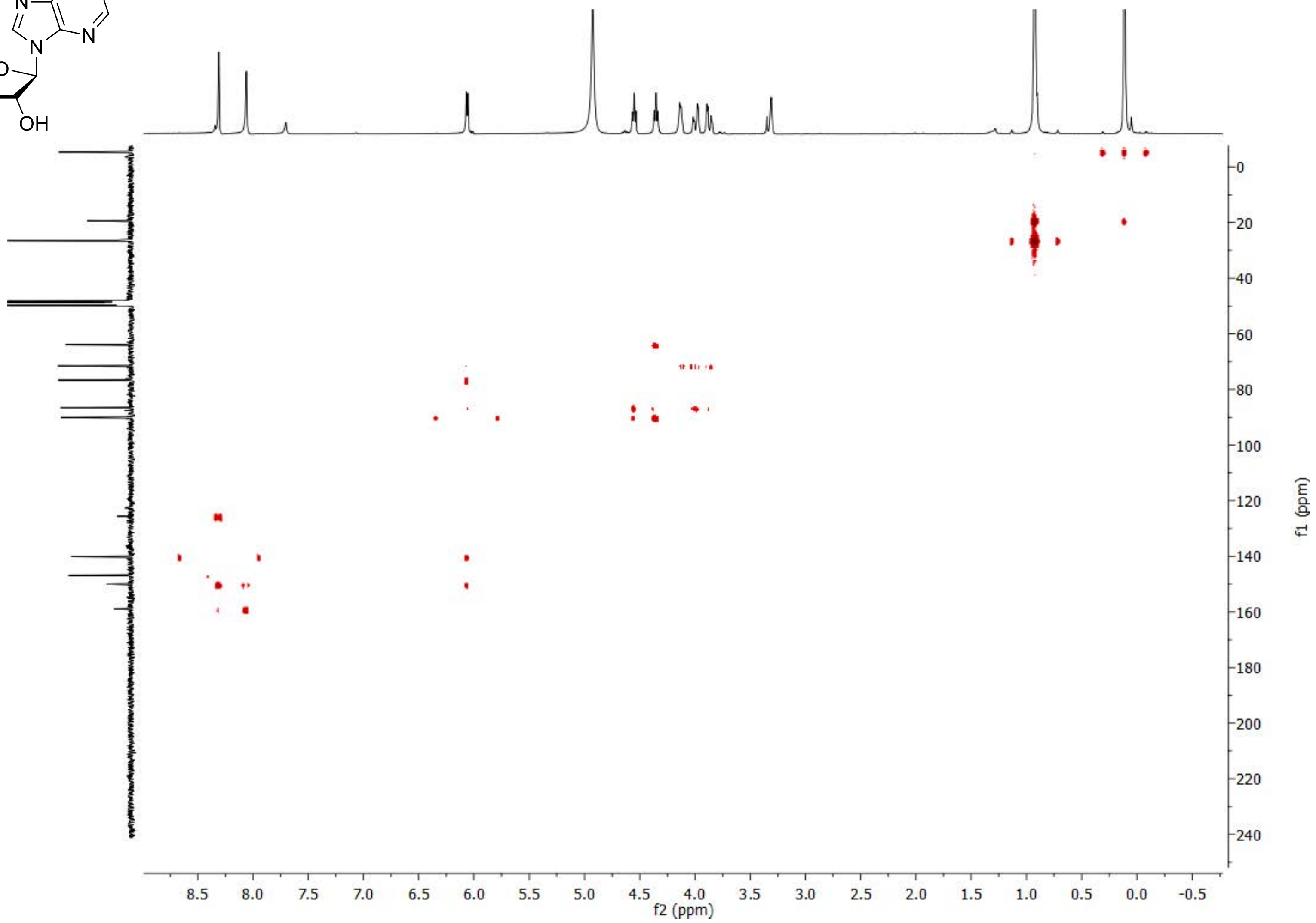

5’-*O*-(*tert*-Butyldimethylsilyl)-2’,3’-bis-*O*-[(ethylthio)thiocarbonyl]-β-D-uridine (6a)

^{1}H-NMR (300.13 MHz, $CDCl_3$)

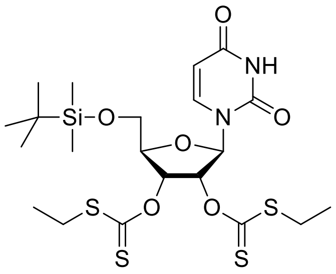

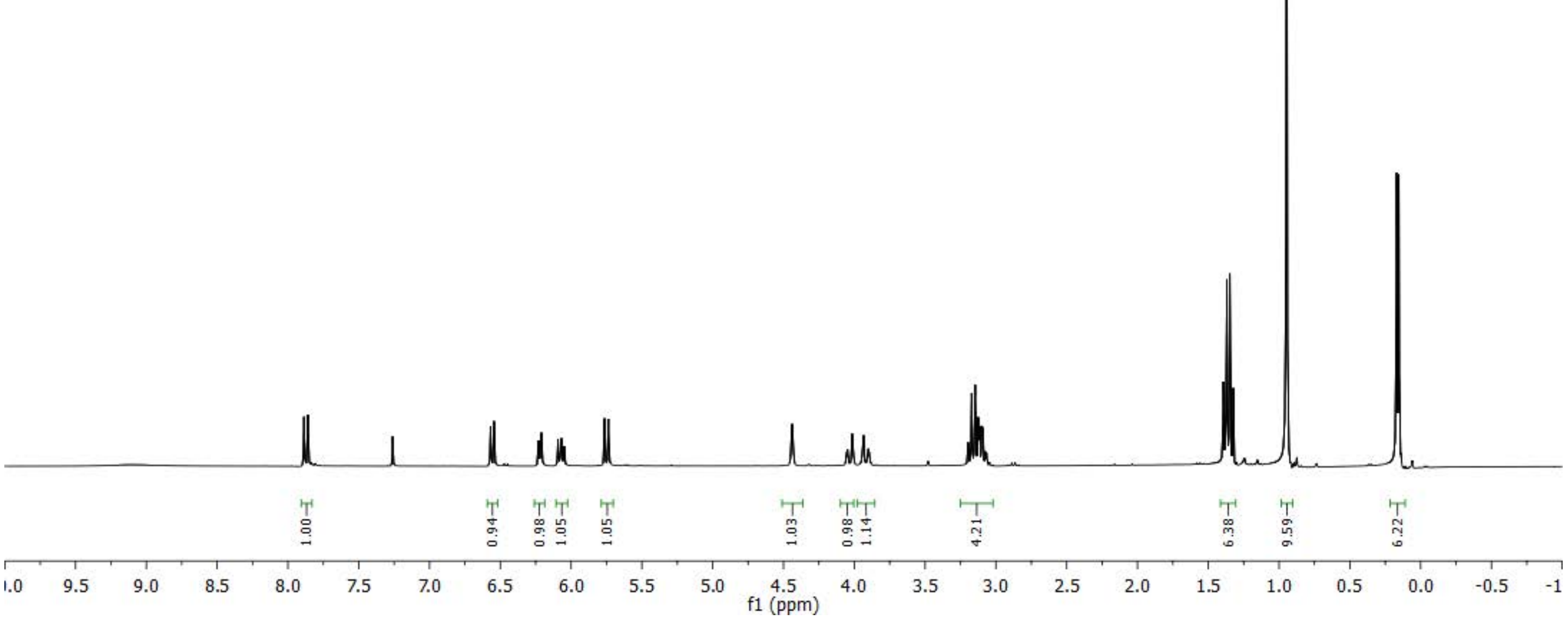

5'-*O*-(*tert*-Butyldimethylsilyl)-2',3'-bis-*O*-[(ethylthio)thiocarbonyl]-β-D-uridine (6a)

^{13}C-NMR (75.5 MHz, $CDCl_3$)

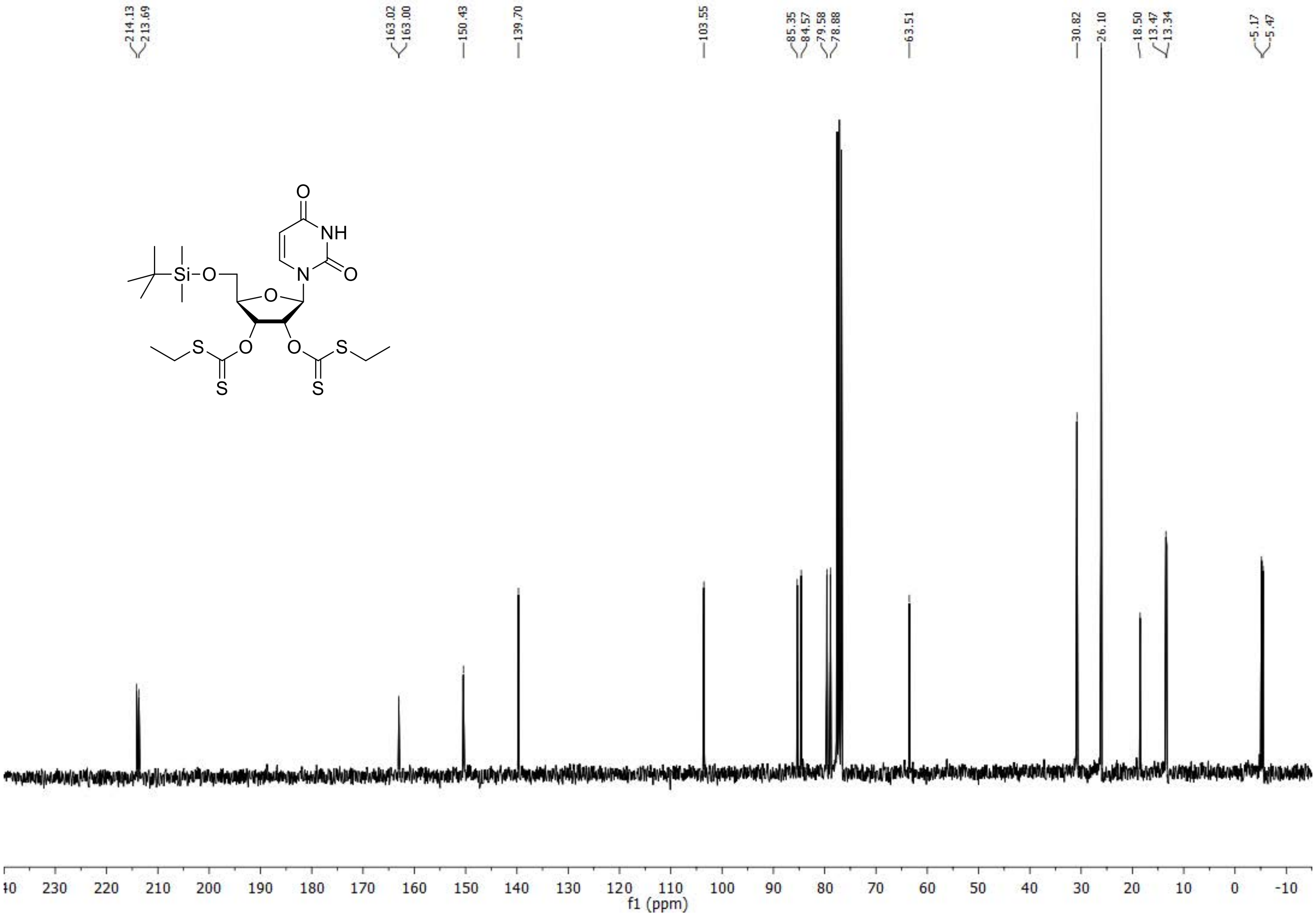

5'-*O*-(*tert*-Butyldimethylsilyl)-2',3'-bis-*O*-[(ethylthio)thiocarbonyl]-β-D-uridine (6a)

^{13}C-NMR (75.5 MHz, $CDCl_3$)

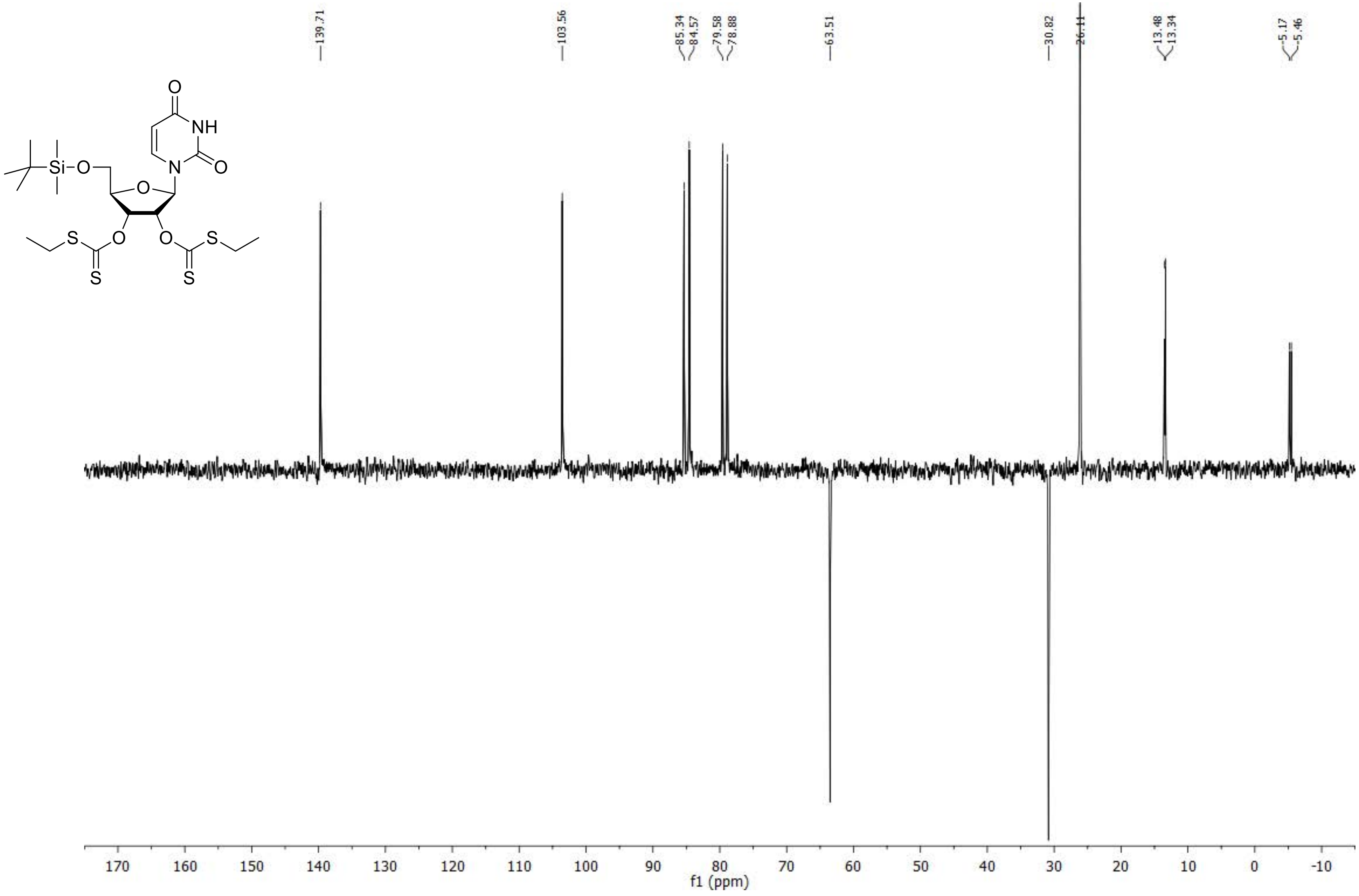

5’-*O*-(*tert*-Butyldimethylsilyl)-2’,3’-bis-*O*-[(ethylthio)thiocarbonyl]-β-D-uridine (6a)

COSY NMR ($CDCl_3$)

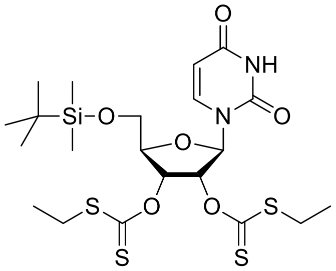

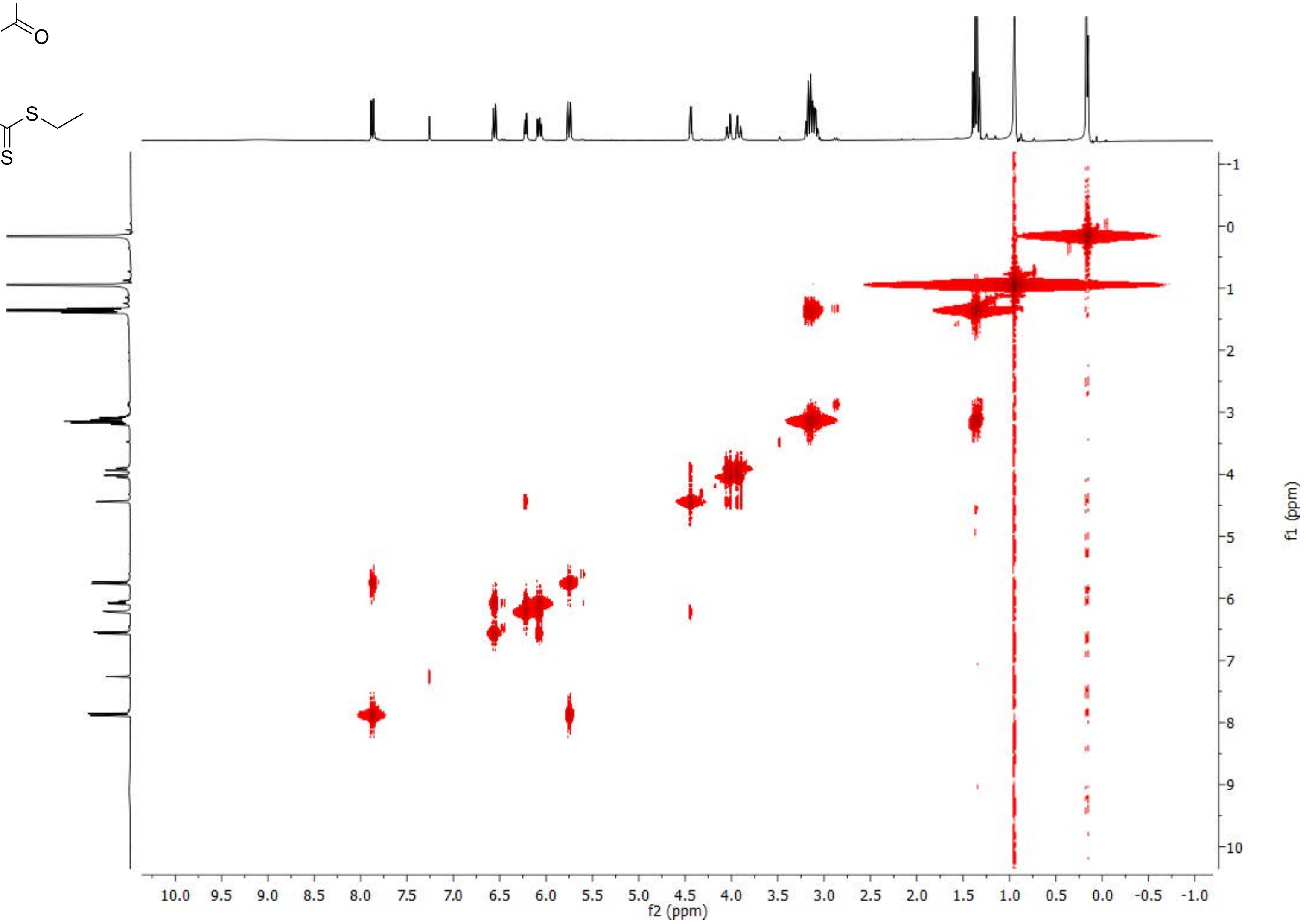

5'-*O*-(*tert*-Butyldimethylsilyl)-2',3'-bis-*O*-[(ethylthio)thiocarbonyl]-β-D-uridine (6a)

HSQC NMR ($CDCl_3$)

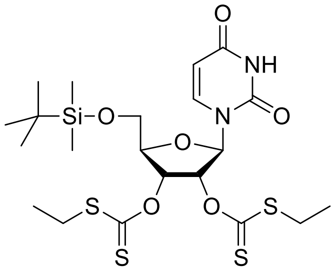

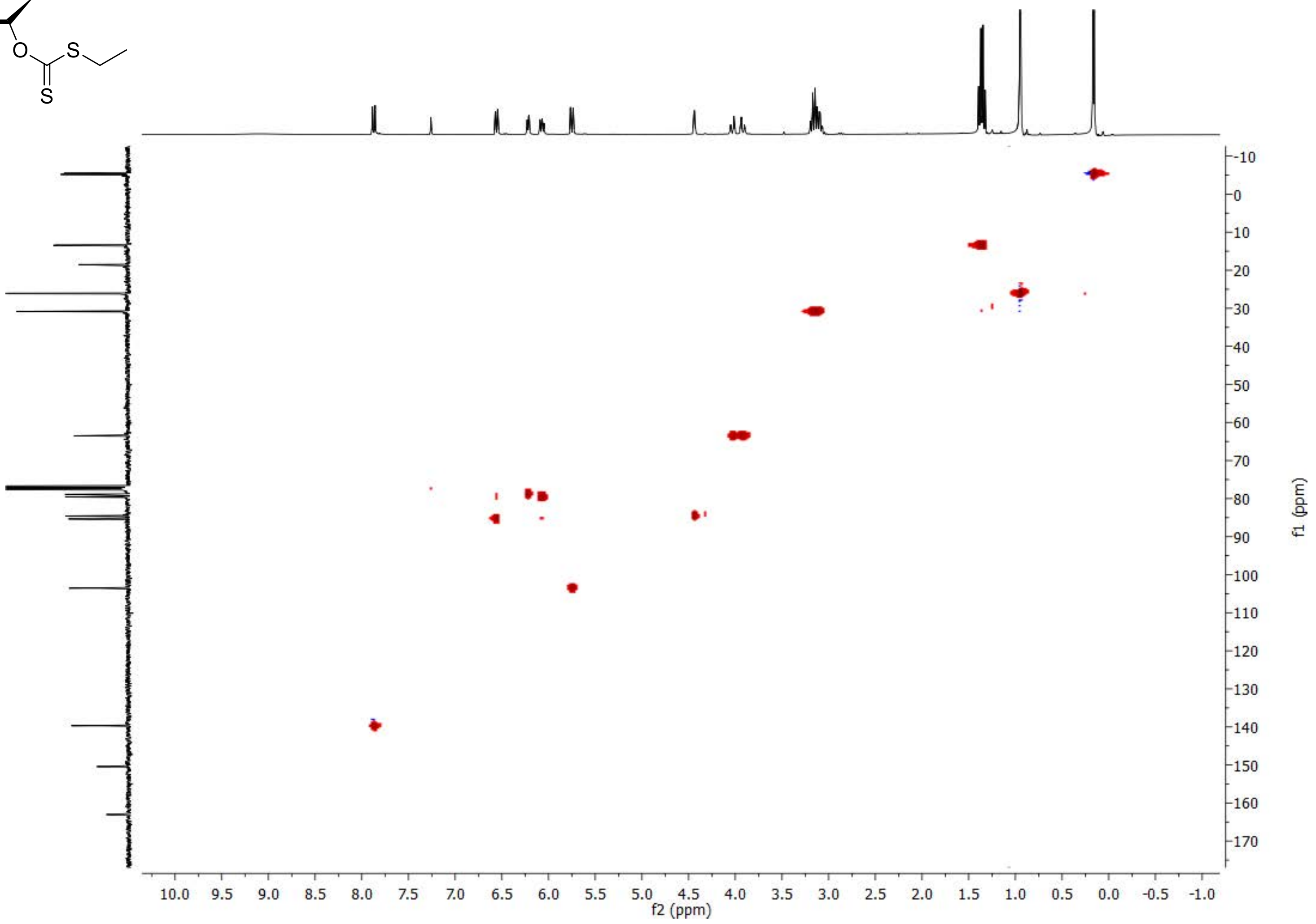

5’-*O*-(*tert*-Butyldimethylsilyl)-2’,3’-bis-*O*-[(ethylthio)thiocarbonyl]-β-D-5-methyluridine (6b)

^{1}H-NMR (300.13 MHz, $CDCl_3$)

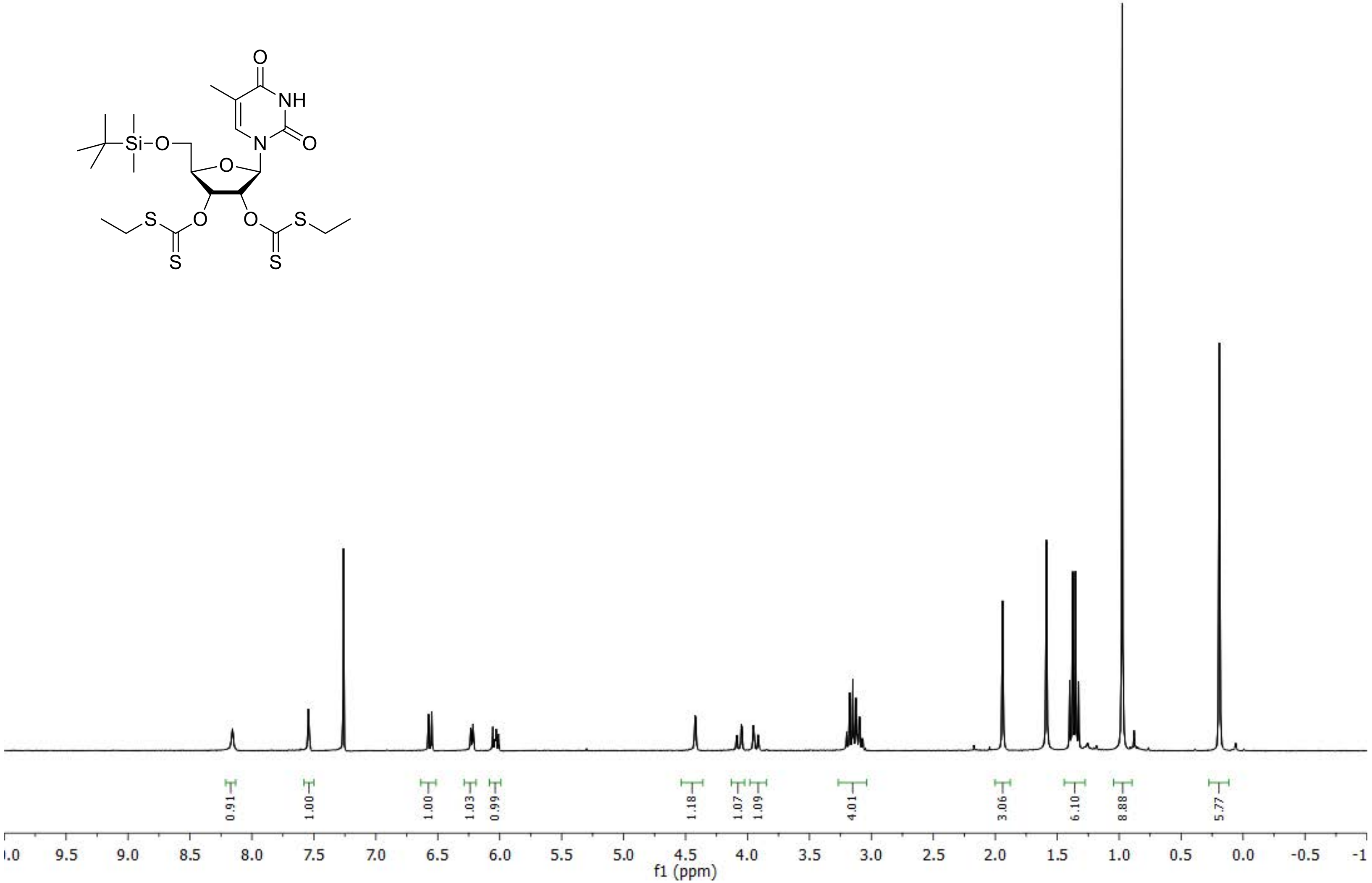

5’-*O*-(*tert*-Butyldimethylsilyl)-2’,3’-bis-*O*-[(ethylthio)thiocarbonyl]-β-D-5-methyluridine (6b)

^{13}C-NMR (75.5 MHz, $CDCl_3$)

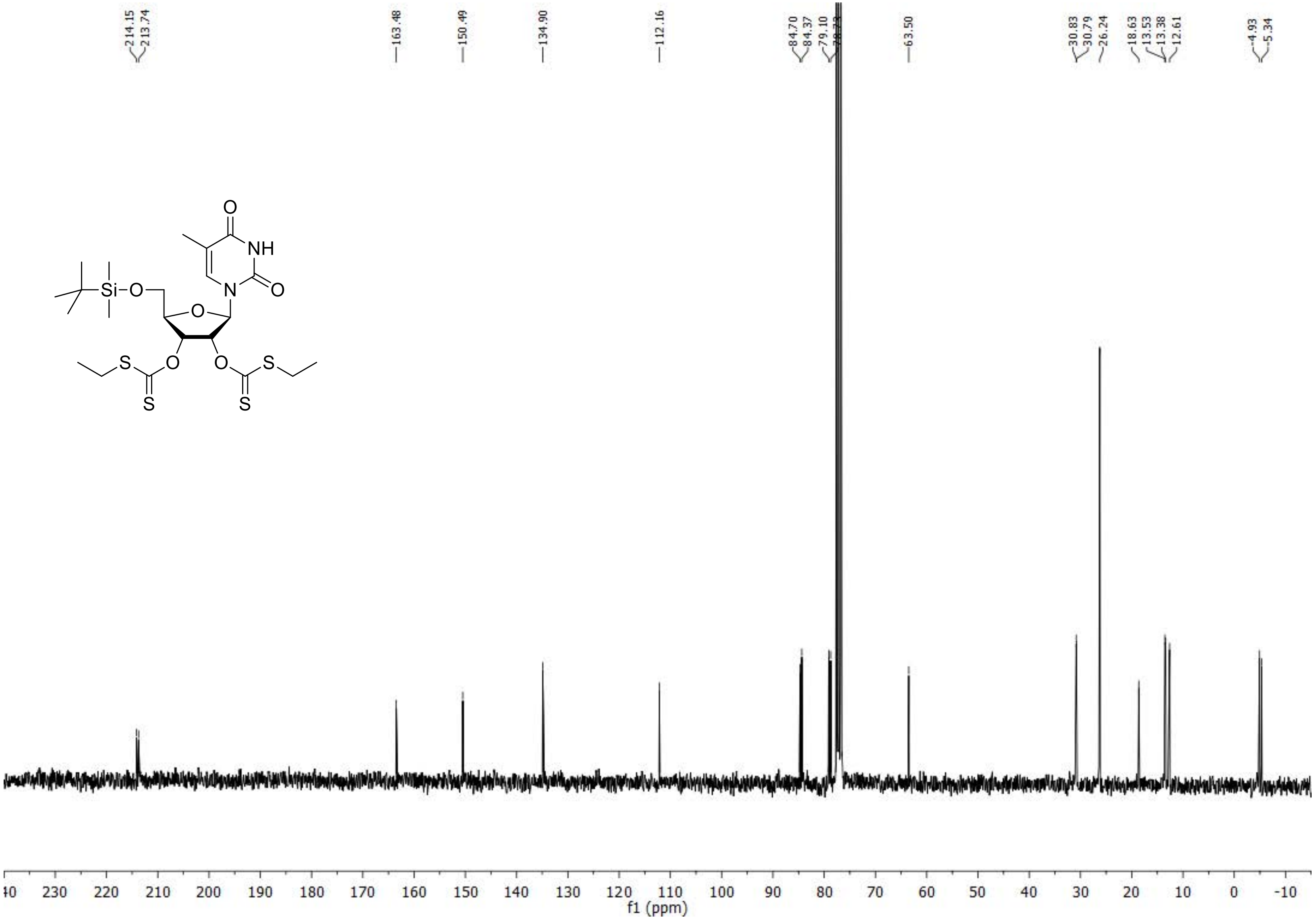

5’-*O*-(*tert*-Butyldimethylsilyl)-2’,3’-bis-*O*-[(ethylthio)thiocarbonyl]-β-D-5-methyluridine (6b)

DEPT NMR (75.5 MHz, $CDCl_3$)

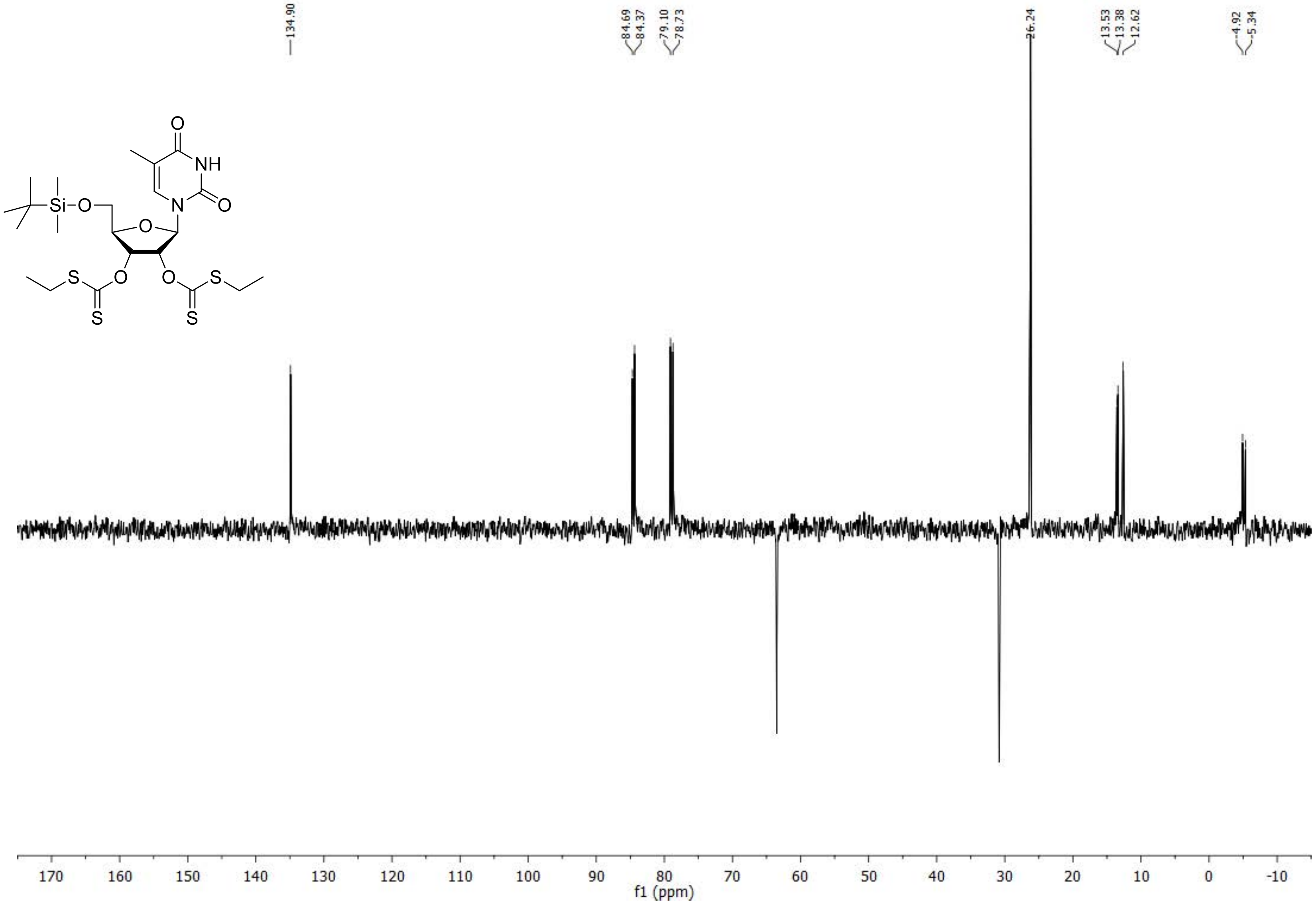

5’-*O*-(*tert*-Butyldimethylsilyl)-2’,3’-bis-*O*-[(ethylthio)thiocarbonyl]-β-D-5-methyluridine (6b)

COSY NMR ($CDCl_3$)

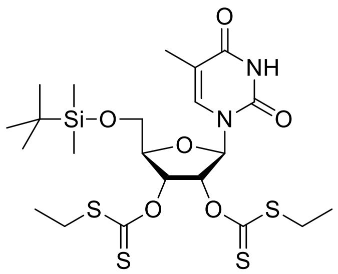

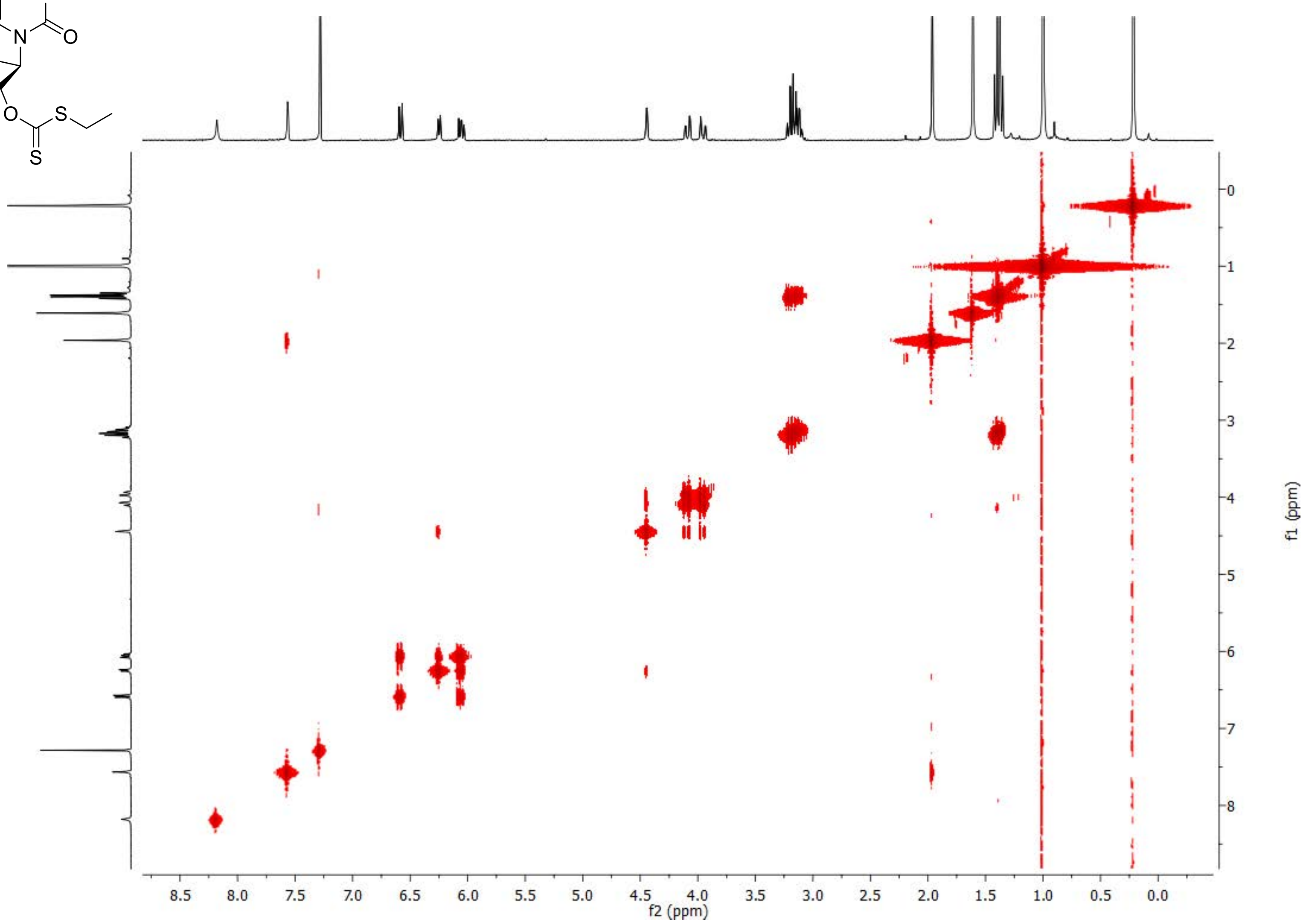

5’-*O*-(*tert*-Butyldimethylsilyl)-2’,3’-bis-*O*-[(ethylthio)thiocarbonyl]-β-D-5-methyluridine (6b)

HSQC NMR ($CDCl_3$)

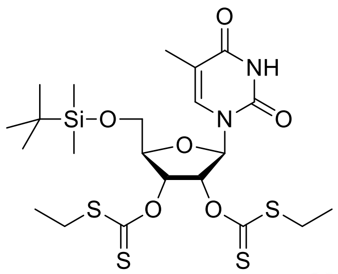

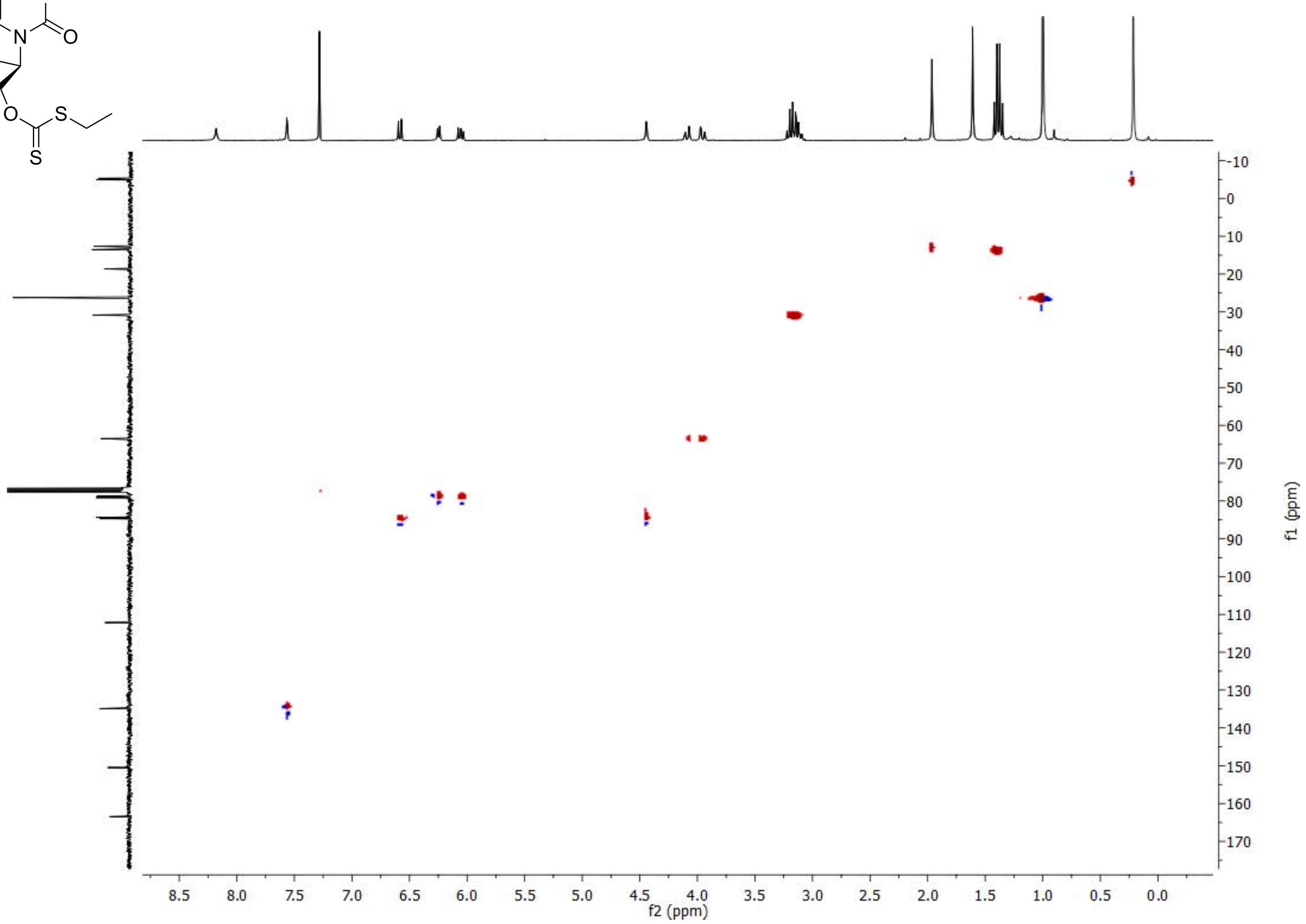

5’-*O*-(*tert*-Butyldimethylsilyl)-2’,3’-bis-*O*-[(ethylthio)thiocarbonyl]-β-D-cytidine (6c)

^{1}H-NMR (300.13 MHz, $CDCl_3$)

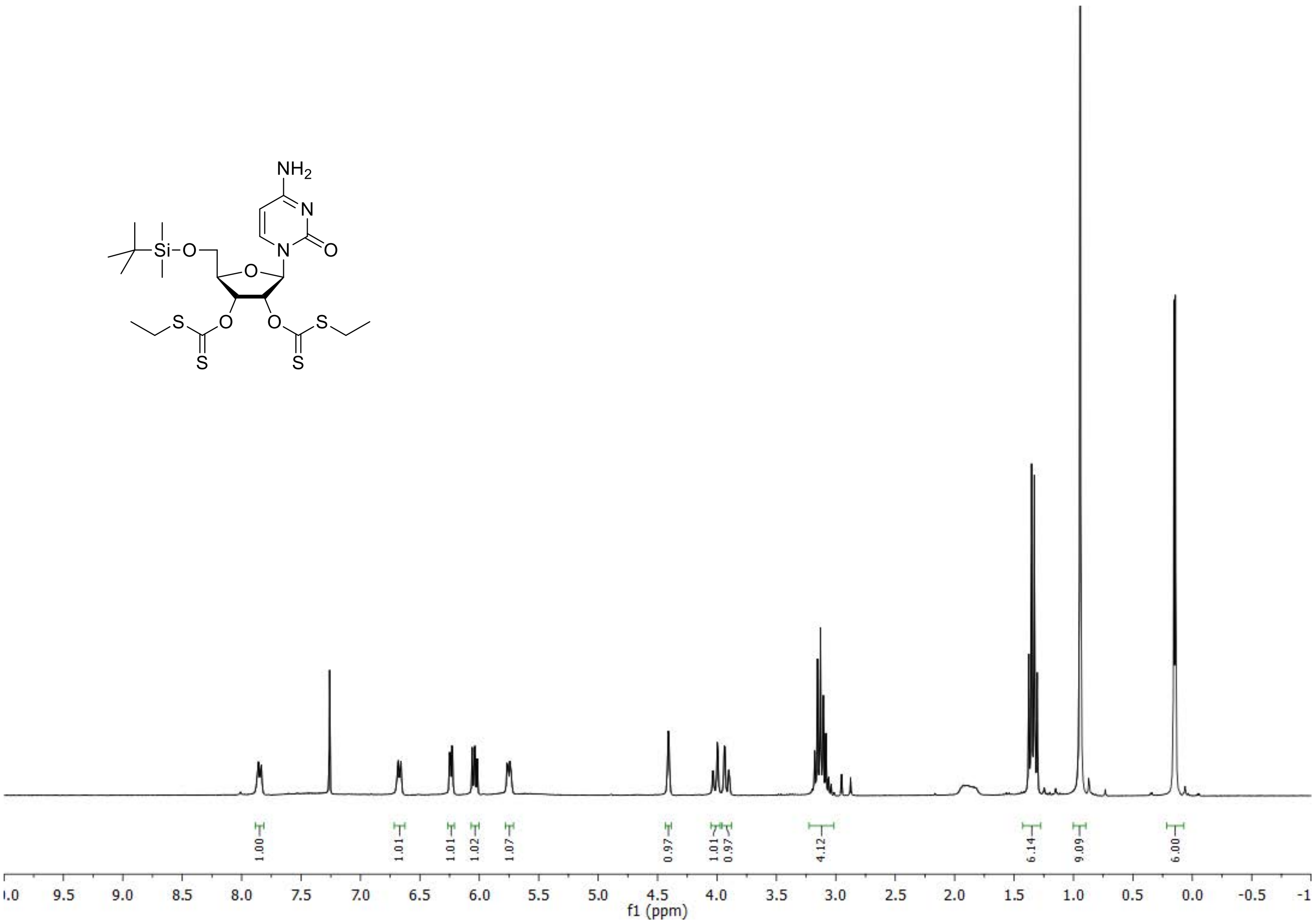

5’-*O*-(*tert*-Butyldimethylsilyl)-2’,3’-bis-*O*-[(ethylthio)thiocarbonyl]-β-D-cytidine (6c)

^{13}C-NMR (75.5 MHz, $CDCl_3$)

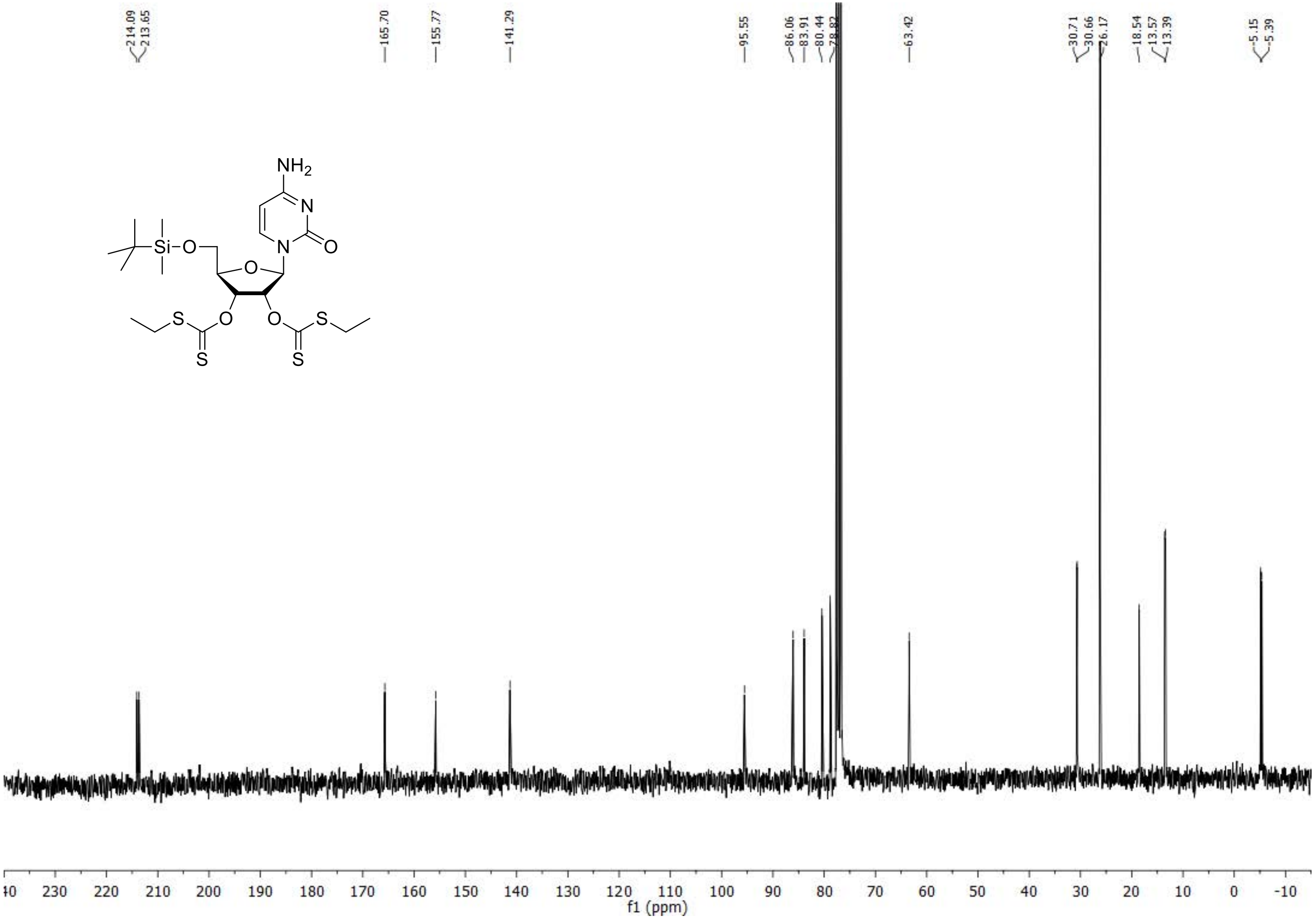

5’-*O*-(*tert*-Butyldimethylsilyl)-2’,3’-bis-*O*-[(ethylthio)thiocarbonyl]-β-D-cytidine (6c)

DEPT NMR (75.5 MHz, $CDCl_3$)

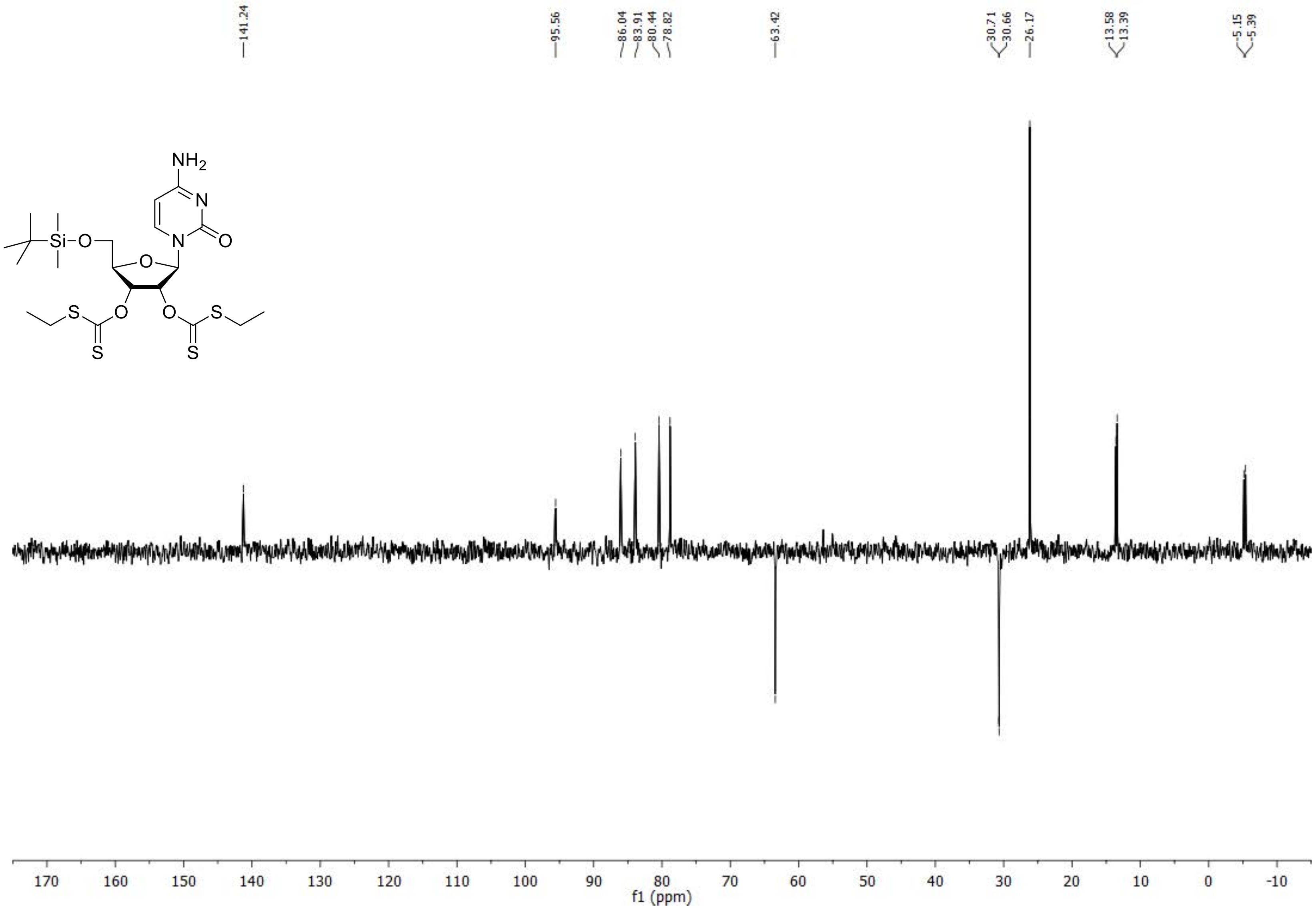

5’-*O*-(*tert*-Butyldimethylsilyl)-2’,3’-bis-*O*-[(ethylthio)thiocarbonyl]-β-D-cytidine (6c)

COSY NMR ($CDCl_3$)

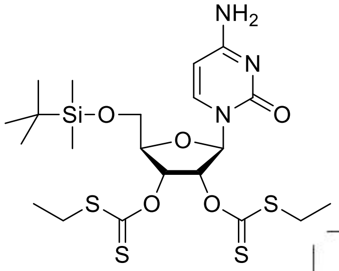

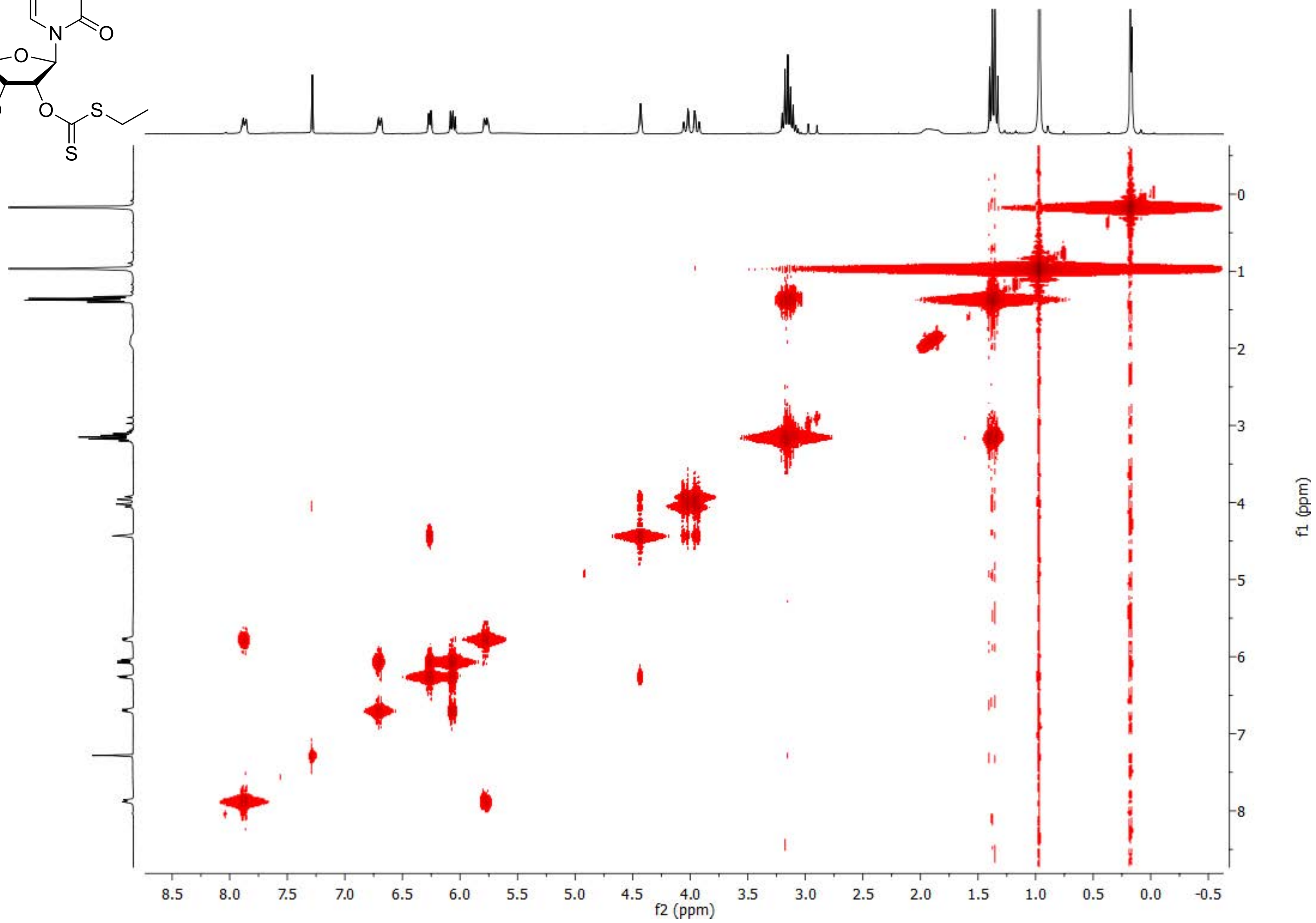

5’-*O*-(*tert*-Butyldimethylsilyl)-2’,3’-bis-*O*-[(ethylthio)thiocarbonyl]-β-D-cytidine (6c)

HSQC NMR ($CDCl_3$)

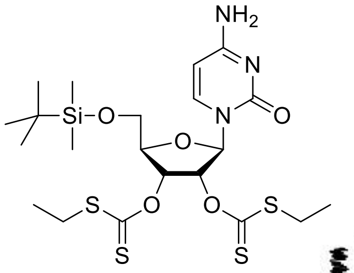

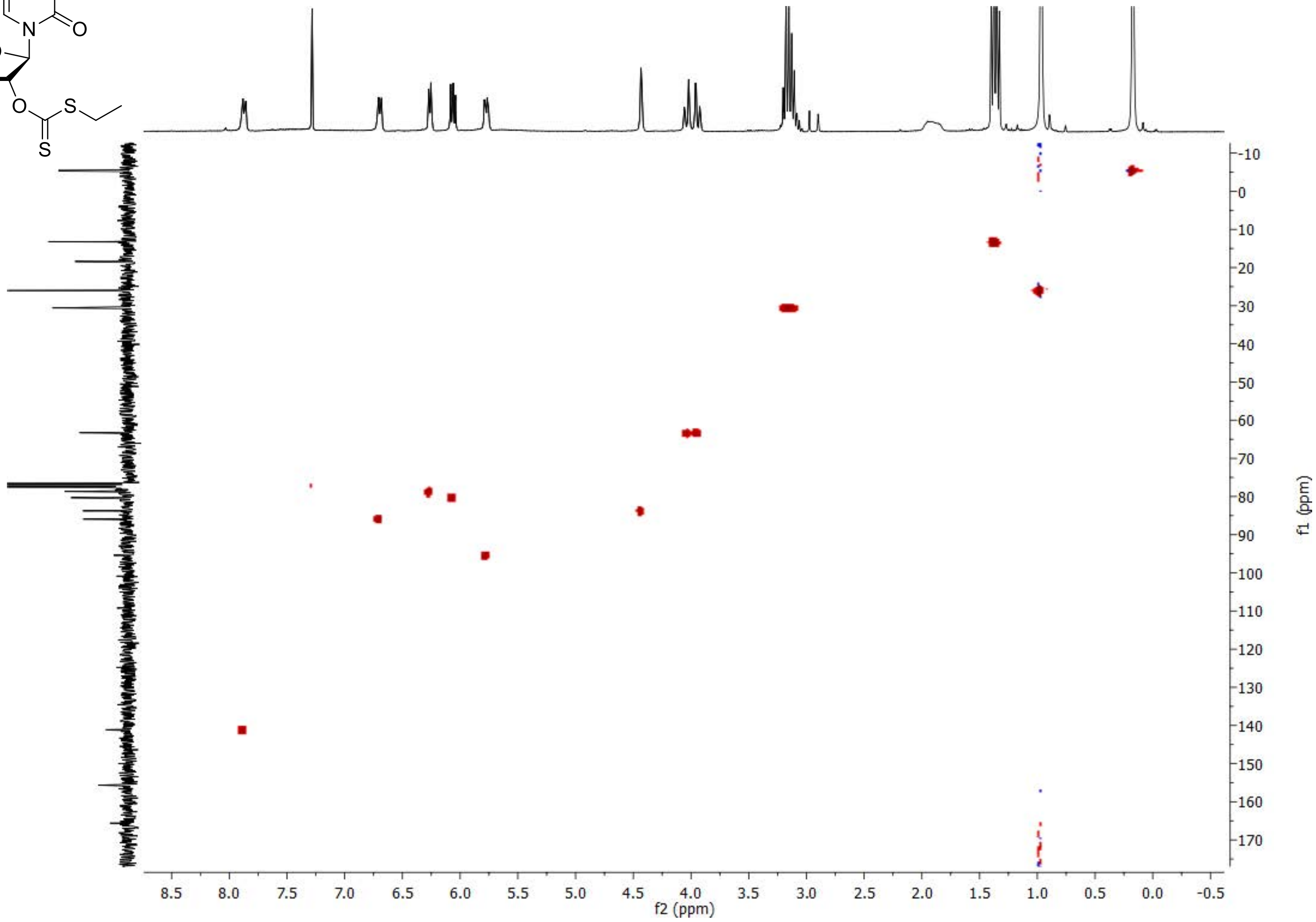

N^4-Benzoyl-5'-*O*-(*tert*-butyldimethylsilyl)-2',3'-bis-*O*-[(ethylthio)thiocarbonyl]-β-D-cytidine (6d)

^{1}H-NMR (300.13 MHz, $CDCl_3$)

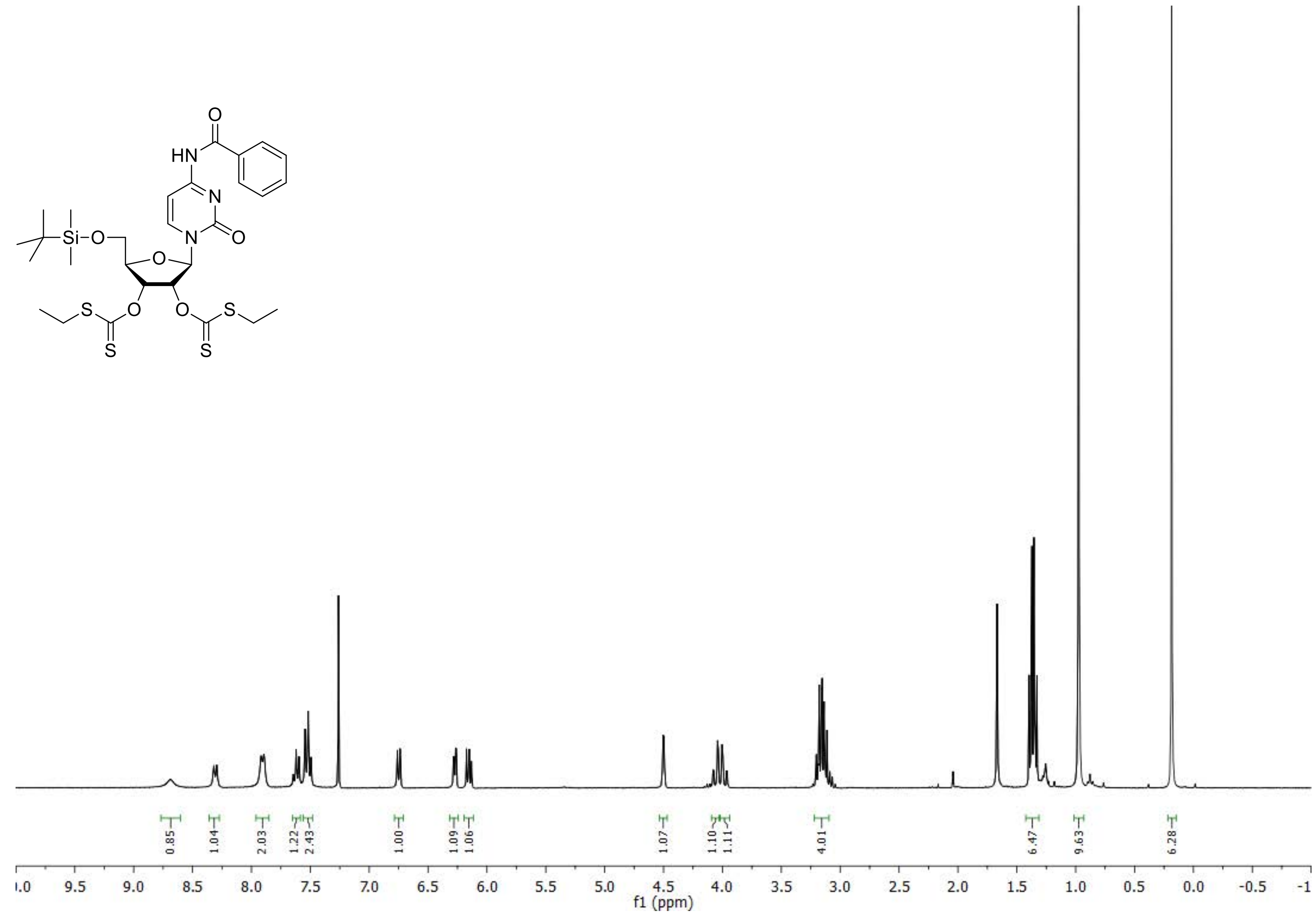

N^4-Benzoyl-5'-*O*-(*tert*-butyldimethylsilyl)-2',3'-bis-*O*-[(ethylthio)thiocarbonyl]-β-D-cytidine (6d)

^{13}C-NMR (75.5 MHz, $CDCl_3$)

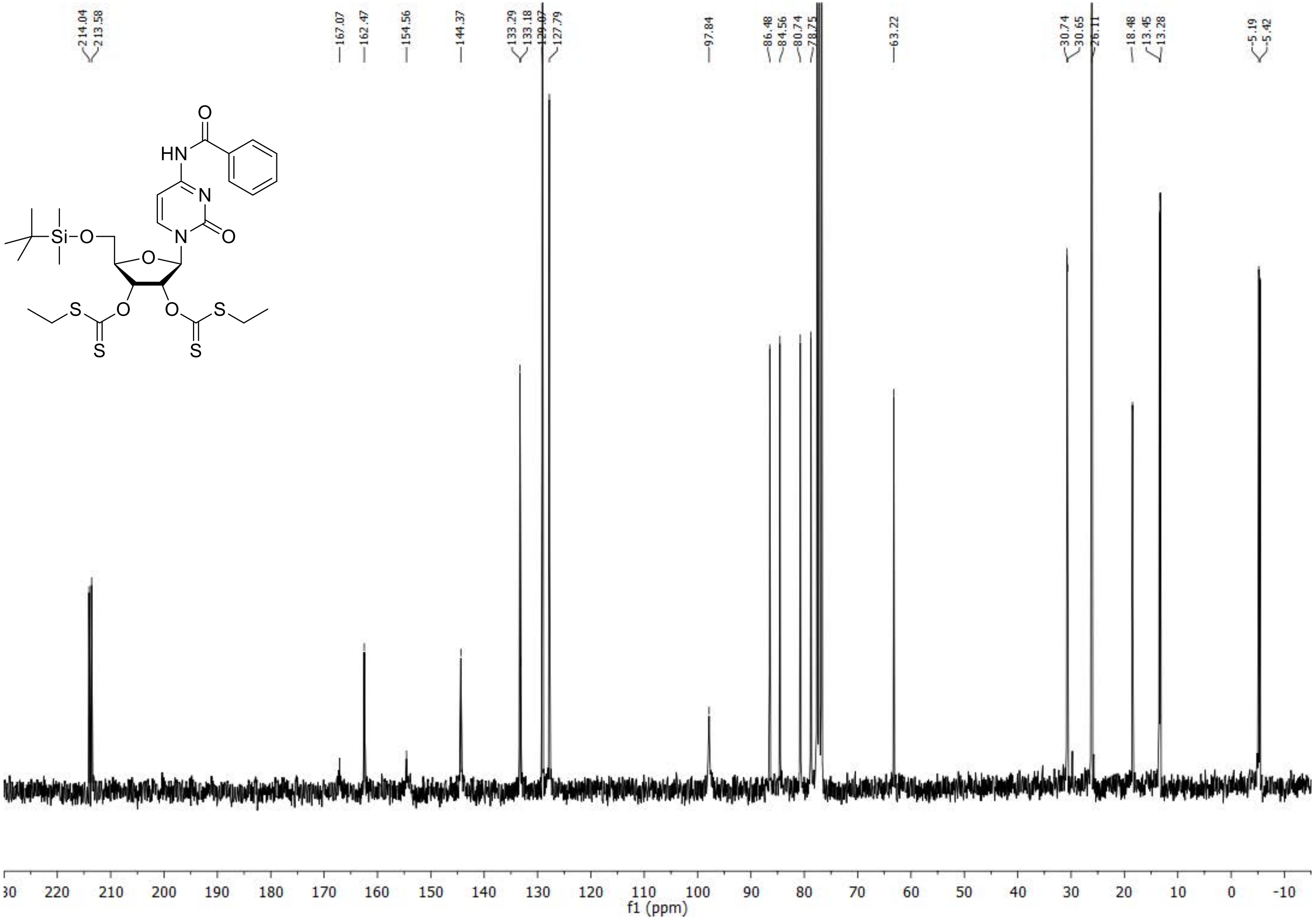

N^4-Benzoyl-5'-*O*-(*tert*-butyldimethylsilyl)-2',3'-bis-*O*-[(ethylthio)thiocarbonyl]-β-D-cytidine (6d)

DEPT NMR (75.5 MHz, $CDCl_3$)

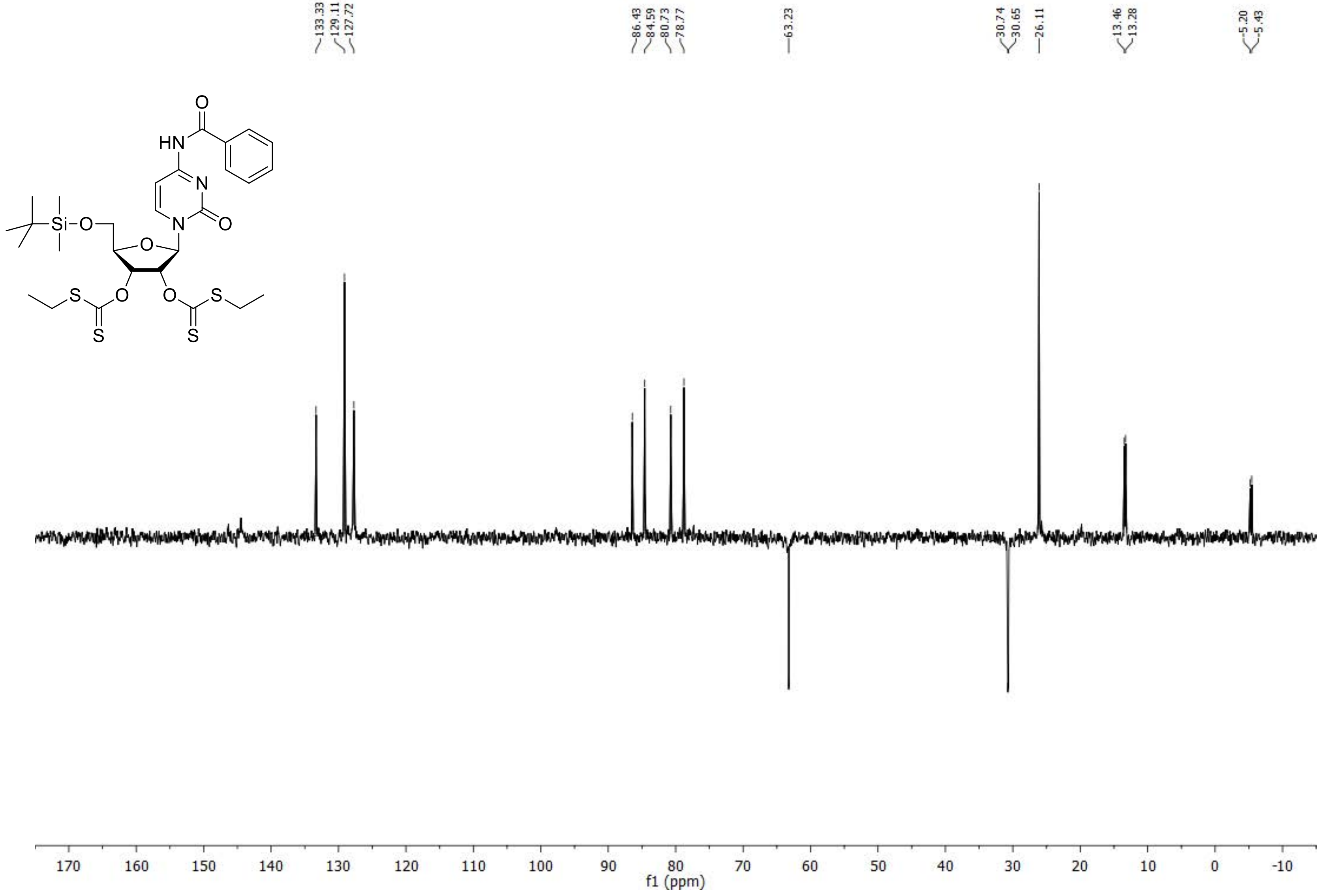

N^4-Benzoyl-5'-*O*-(*tert*-butyldimethylsilyl)-2',3'-bis-*O*-[(ethylthio)thiocarbonyl]-β-D-cytidine (6d)

COSY NMR (75.5 MHz, $CDCl_3$)

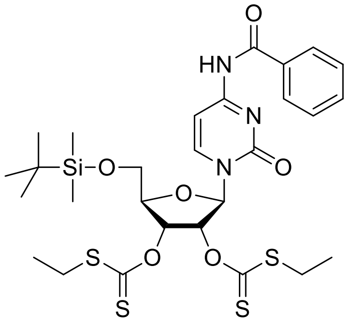

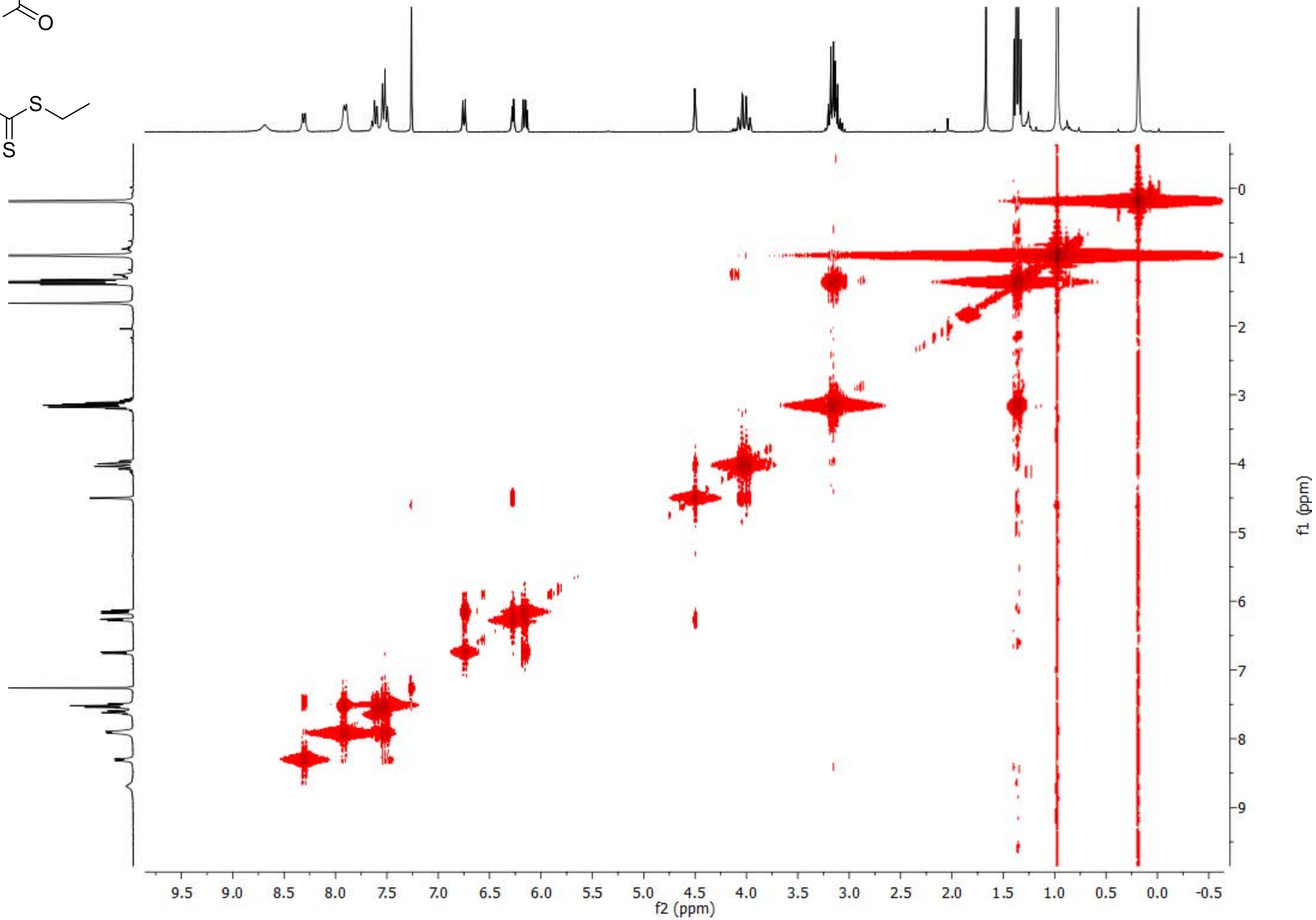

N^4-Benzoyl-5'-*O*-(*tert*-butyldimethylsilyl)-2',3'-bis-*O*-[(ethylthio)thiocarbonyl]-β-D-cytidine (6d)

HSQC NMR (75.5 MHz, $CDCl_3$)

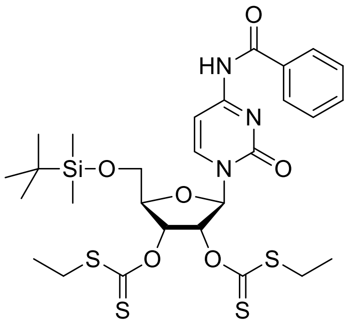

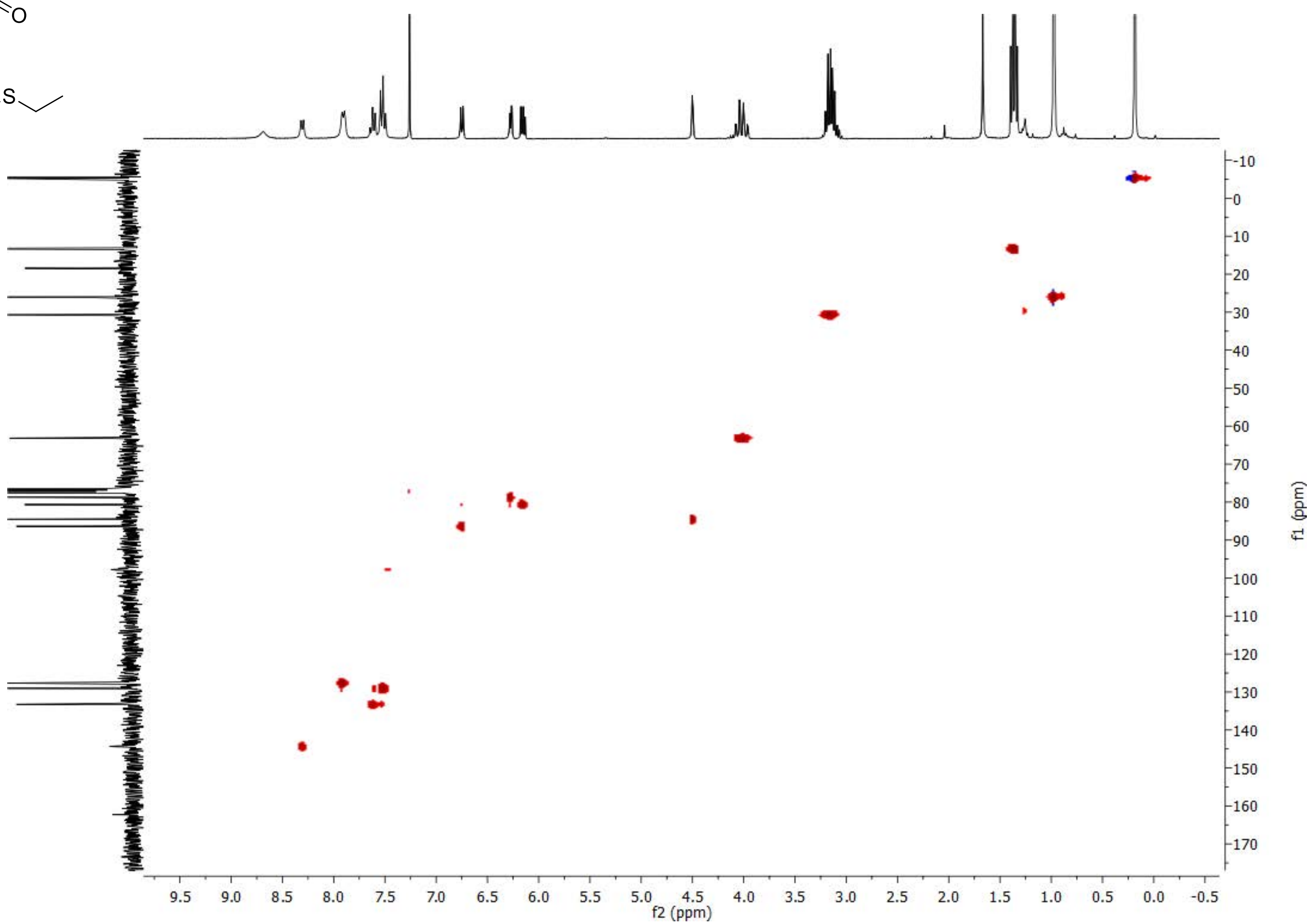

5’-*O*-(*tert*-Butyldimethylsilyl)-2’,3’-bis-*O*-[(ethylthio)thiocarbonyl]-β-D-adenosine (6e)

^{1}H-NMR (300.13 MHz, $CDCl_3$)

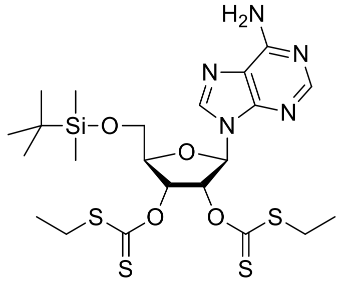

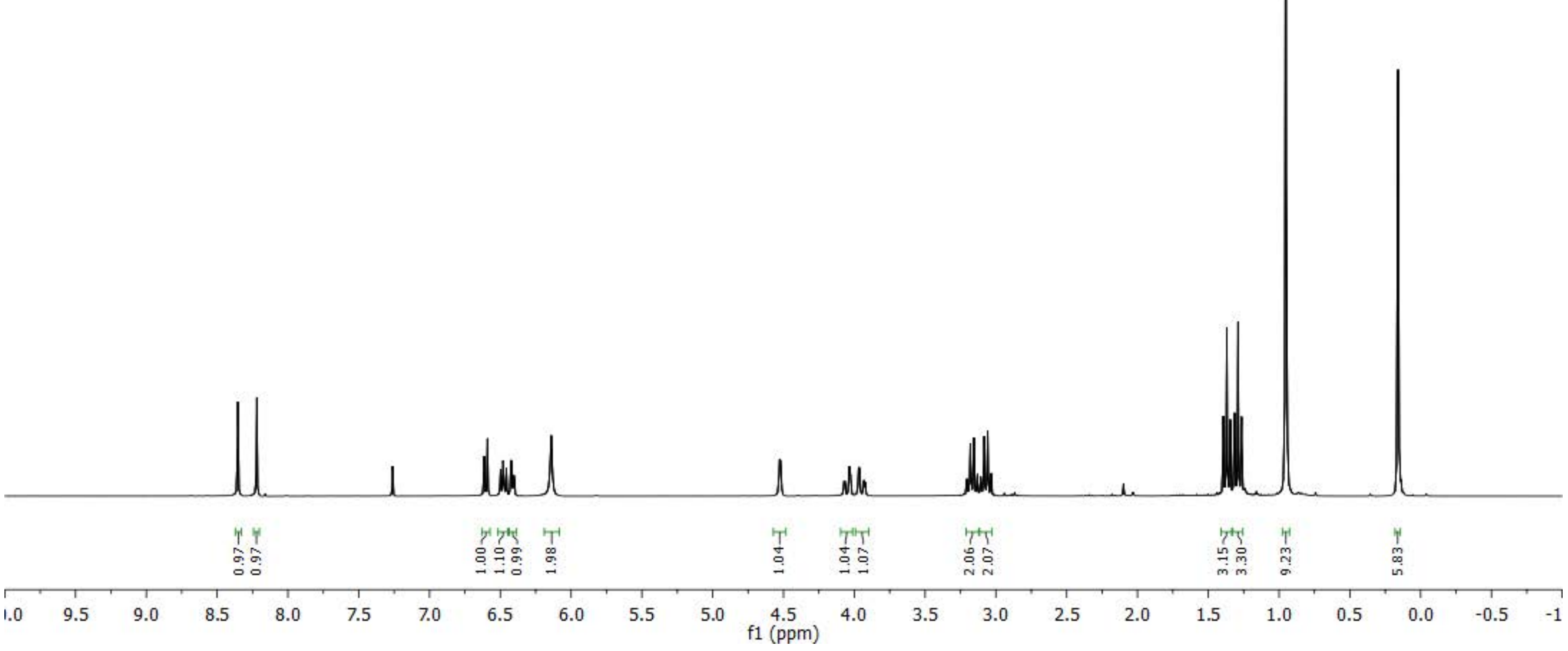

5’-*O*-(*tert*-Butyldimethylsilyl)-2’,3’-bis-*O*-[(ethylthio)thiocarbonyl]-β-D-adenosine (6e)

^{13}C-NMR (75.5 MHz, CDCl$_3$)

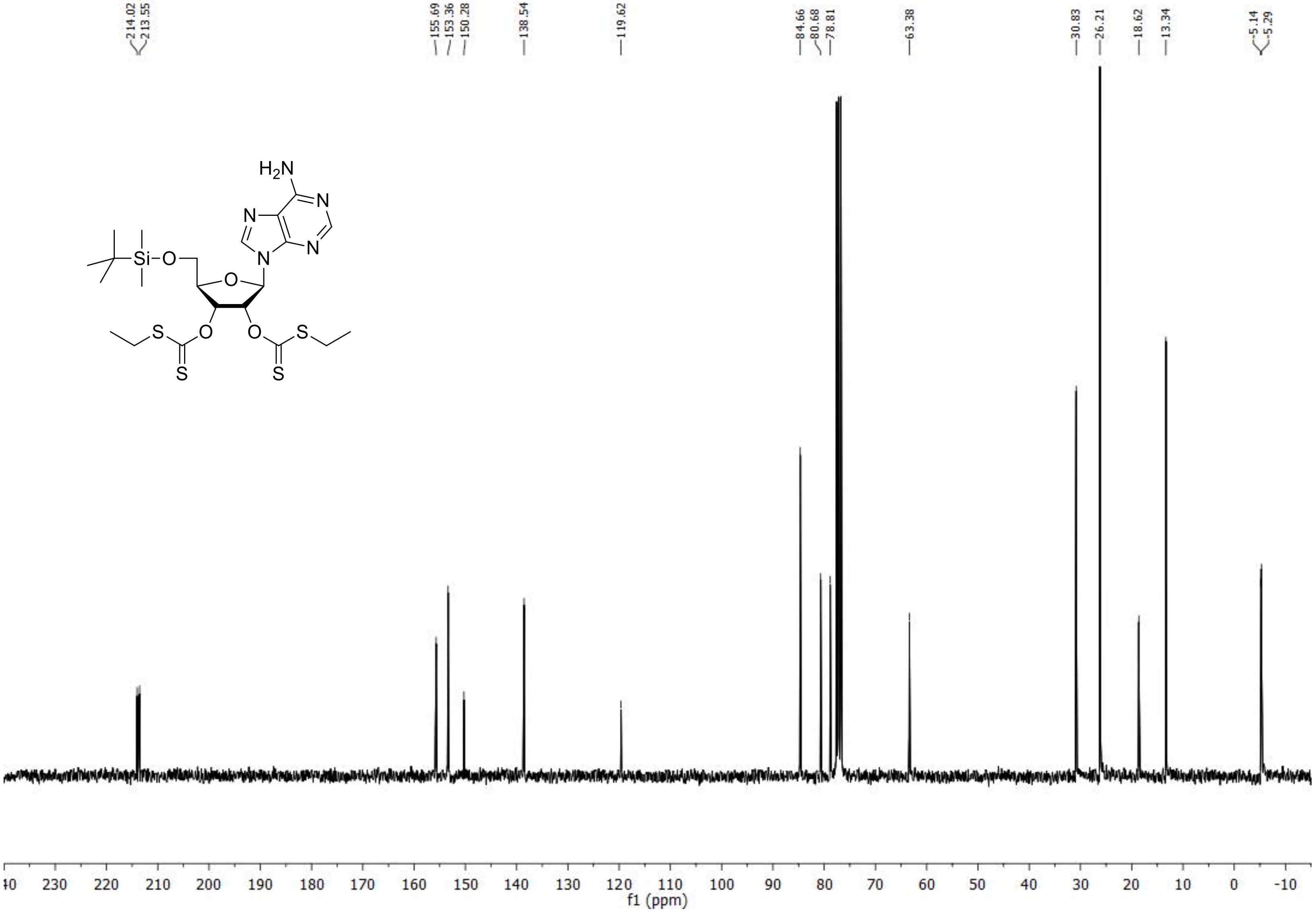

5’-*O*-(*tert*-Butyldimethylsilyl)-2’,3’-bis-*O*-[(ethylthio)thiocarbonyl]-β-D-adenosine (6e)

DEPT NMR (75.5 MHz, $CDCl_3$)

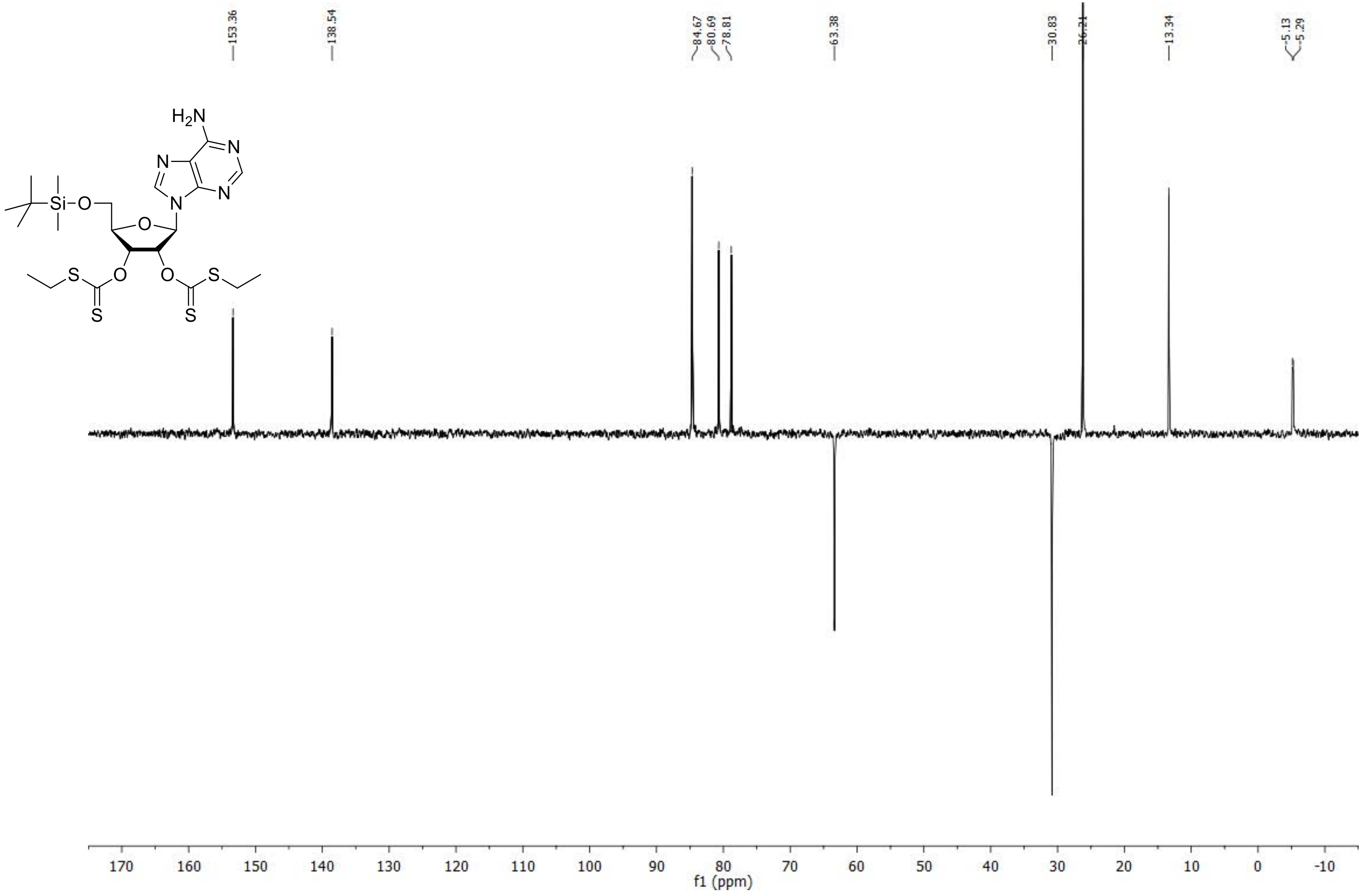

5’-*O*-(*tert*-Butyldimethylsilyl)-2’,3’-bis-*O*-[(ethylthio)thiocarbonyl]-β-D-adenosine (6e)

COSY NMR ($CDCl_3$)

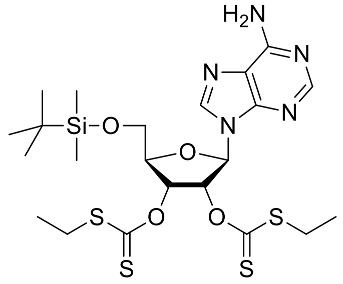

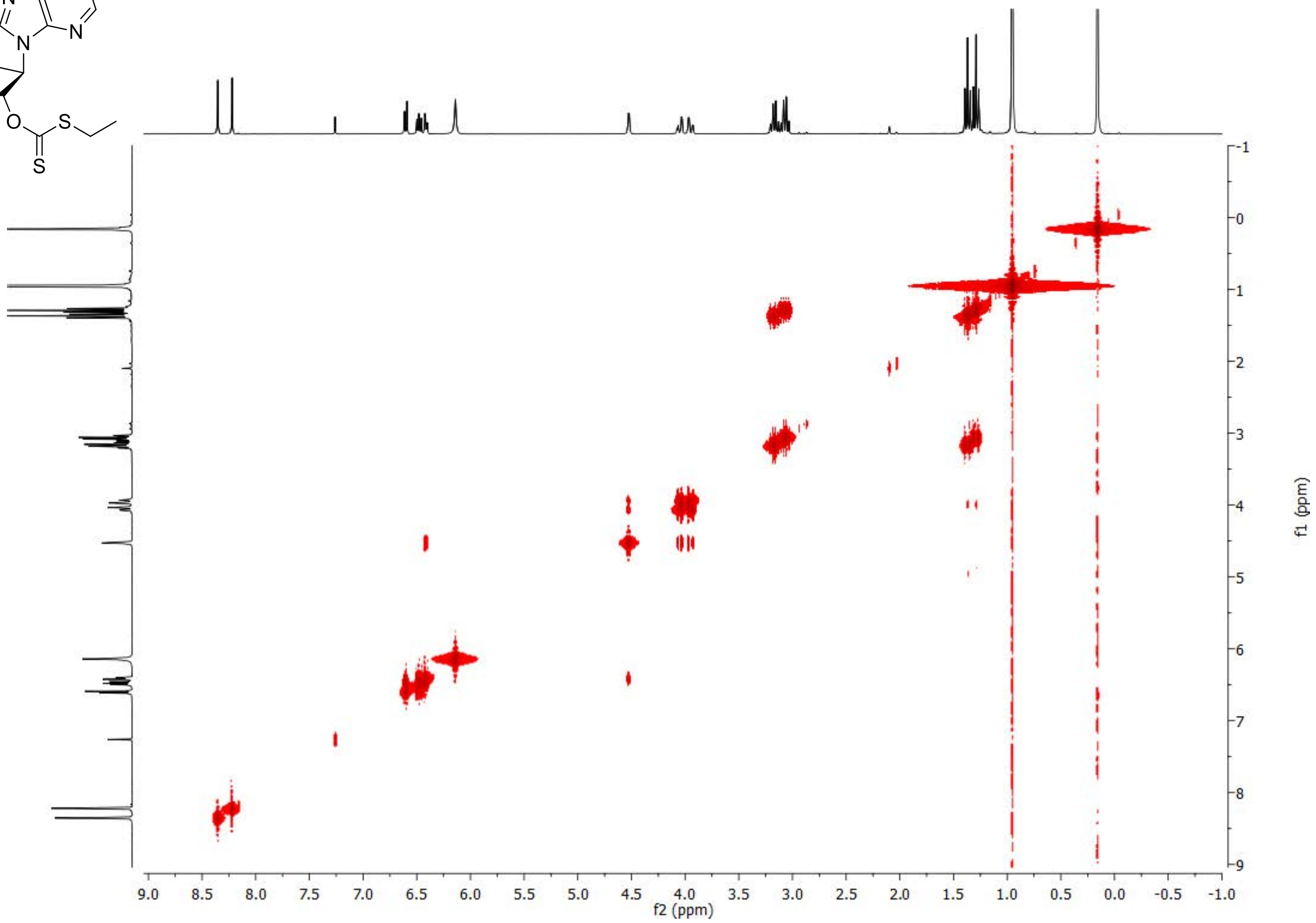

5'-*O*-(*tert*-Butyldimethylsilyl)-2',3'-bis-*O*-[(ethylthio)thiocarbonyl]-β-D-adenosine (6e)

HSQC NMR ($CDCl_3$)

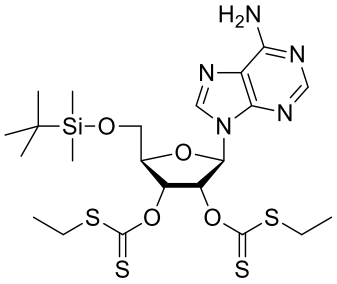

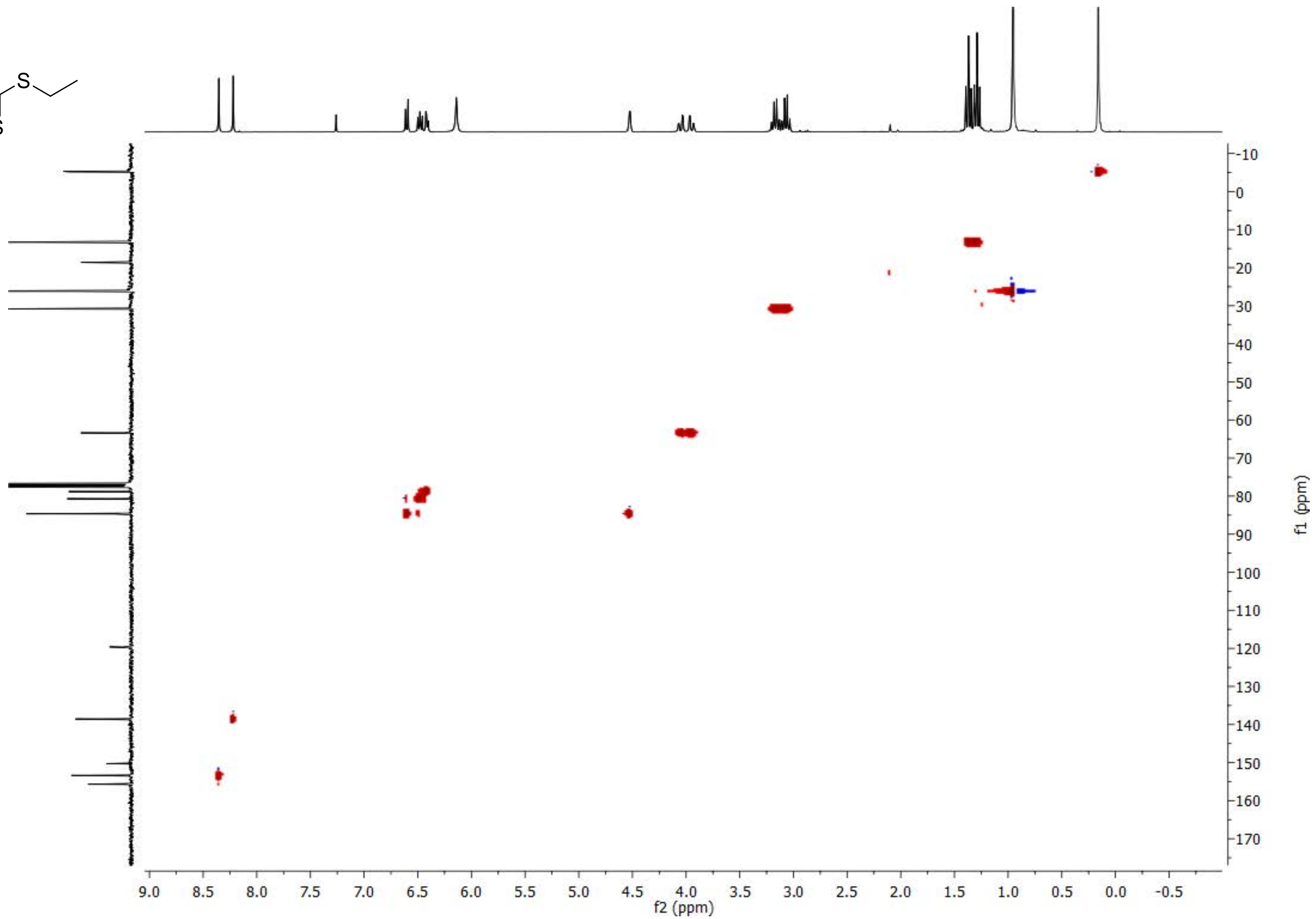

5'-*O*-(*tert*-Butyldimethylsilyl)-2',3'-bis-*O*-[(ethylthio)thiocarbonyl]-β-D-adenosine (6e)

HMBC NMR ($CDCl_3$)

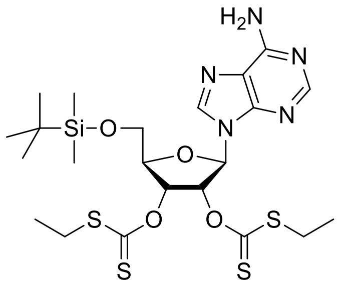

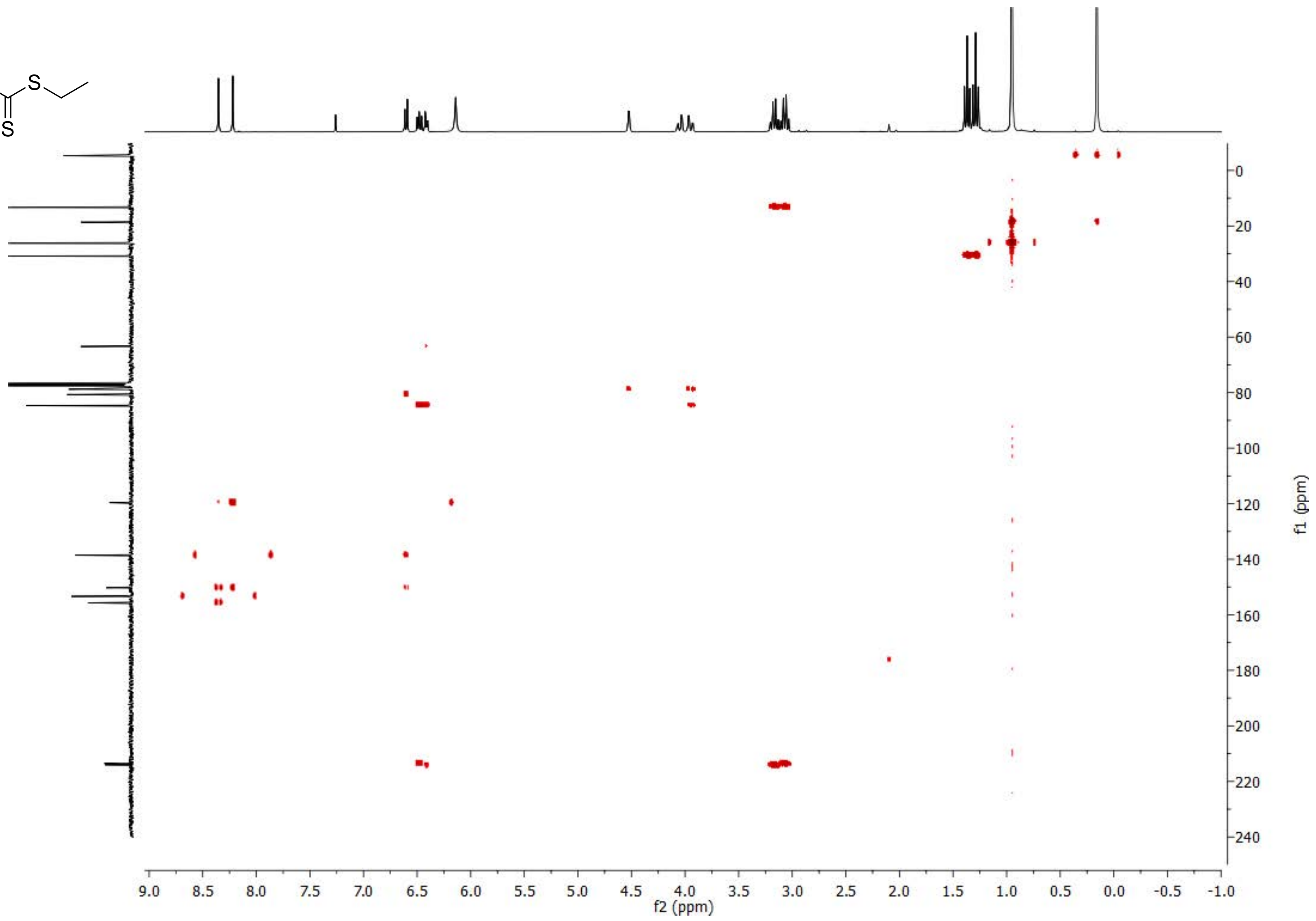

5’-*O*-(*tert*-Butyldimethylsilyl)-2’,3’-bis-*O*-[(ethylthio)thiocarbonyl]-β-D-inosine (6f)

^{1}H-NMR (300.13 MHz, $CDCl_3$)

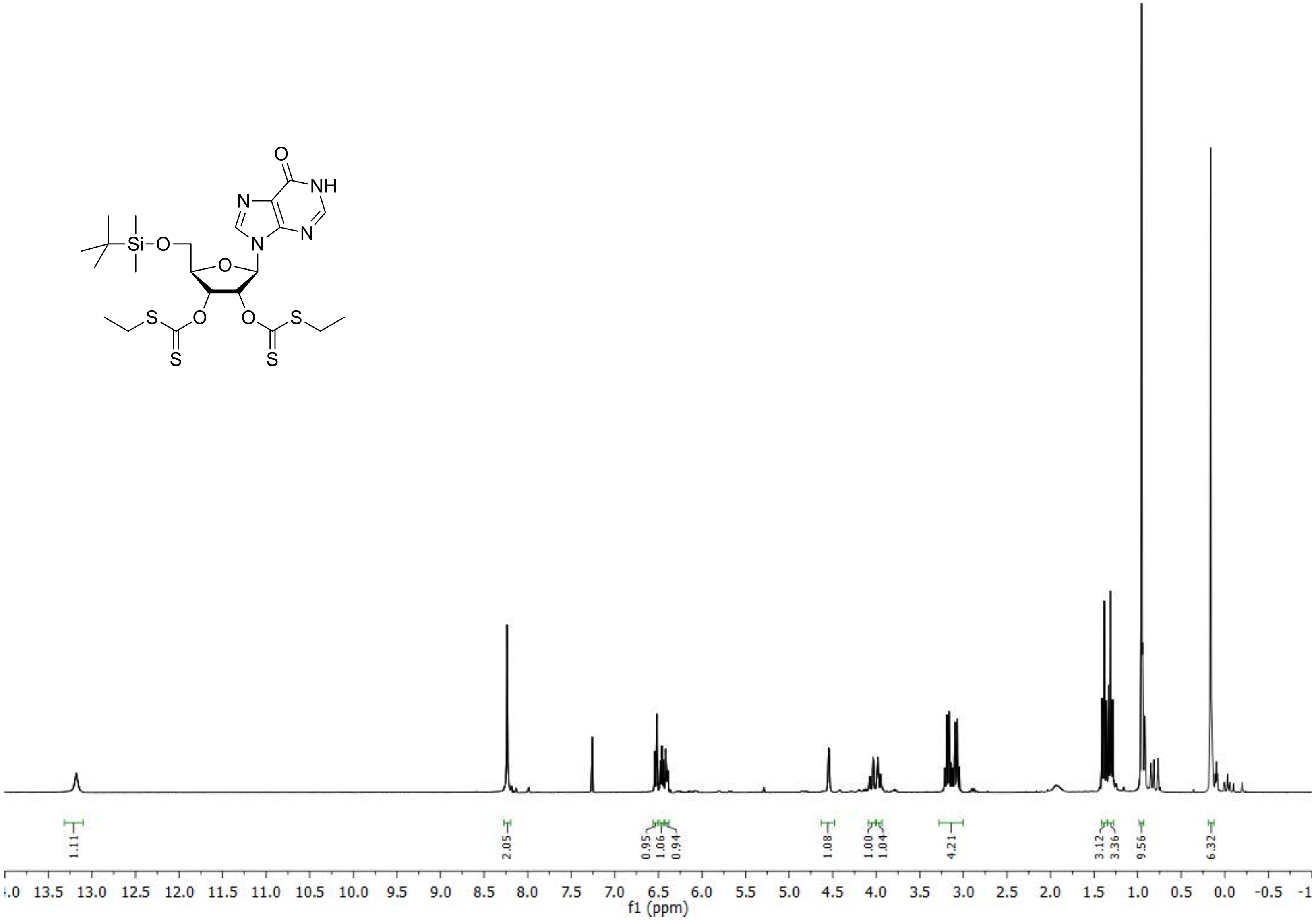

5’-*O*-(*tert*-Butyldimethylsilyl)-2’,3’-bis-*O*-[(ethylthio)thiocarbonyl]-β-D-inosine (6f)

^{13}C-NMR (75.5 MHz, $CDCl_3$)

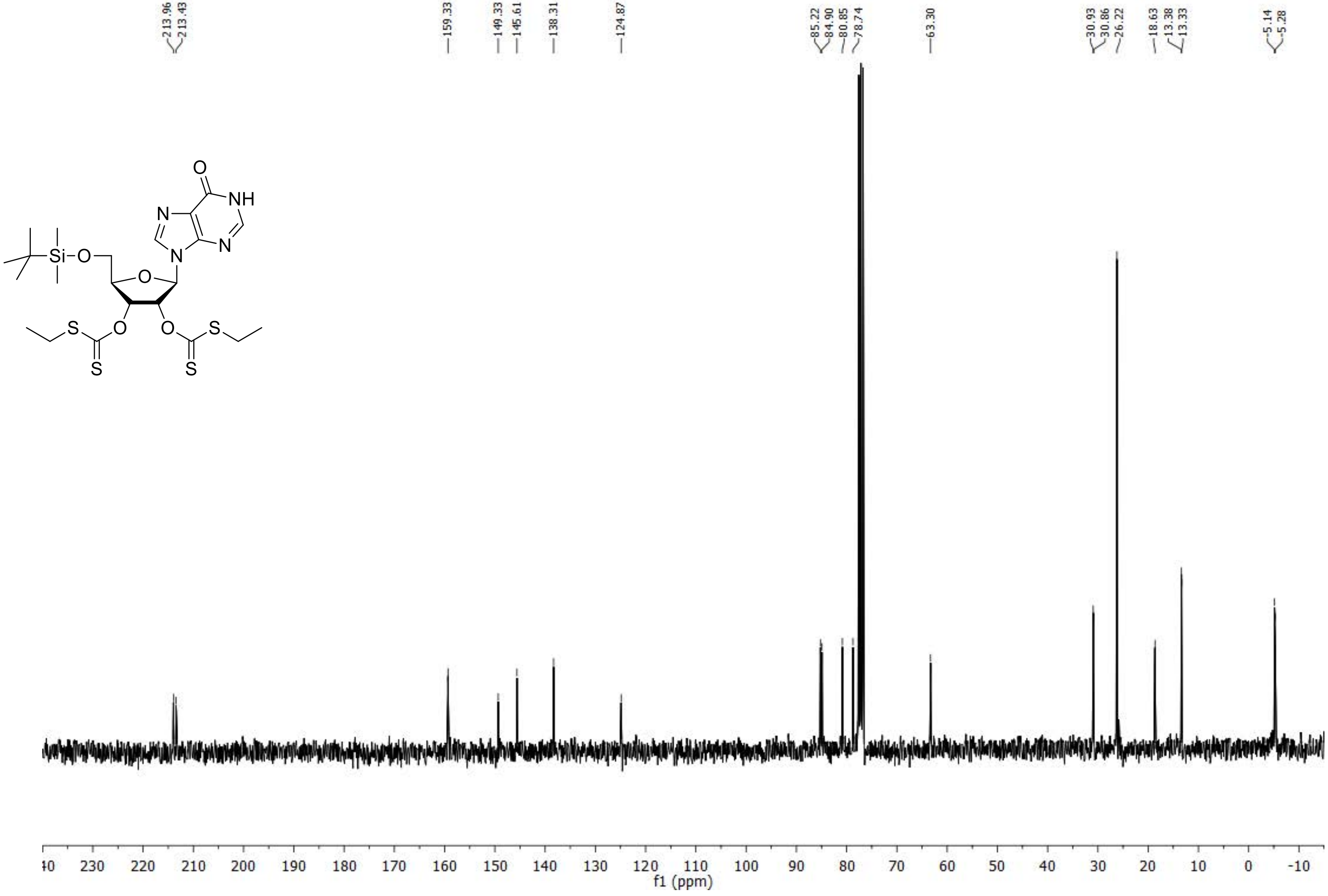

5’-*O*-(*tert*-Butyldimethylsilyl)-2’,3’-bis-*O*-[(ethylthio)thiocarbonyl]-β-D-inosine (6f)

DEPT NMR (75.5 MHz, $CDCl_3$)

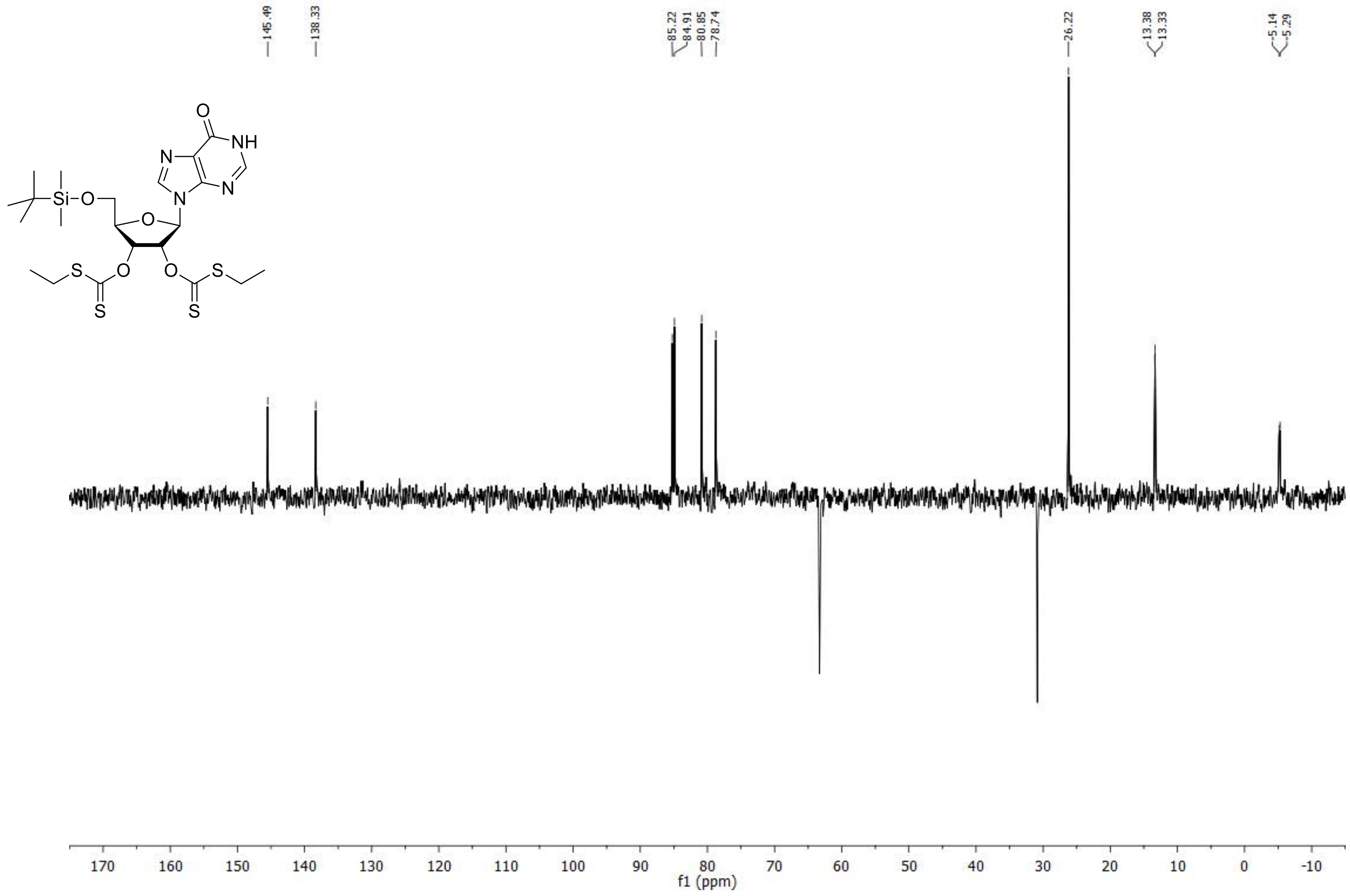

5'-*O*-(*tert*-Butyldimethylsilyl)-2',3'-bis-*O*-[(ethylthio)thiocarbonyl]-β-D-inosine (6f)

COSY NMR ($CDCl_3$)

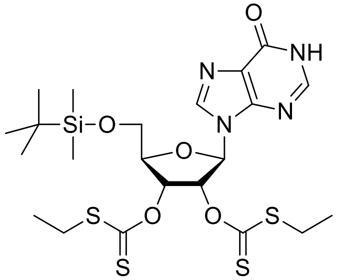

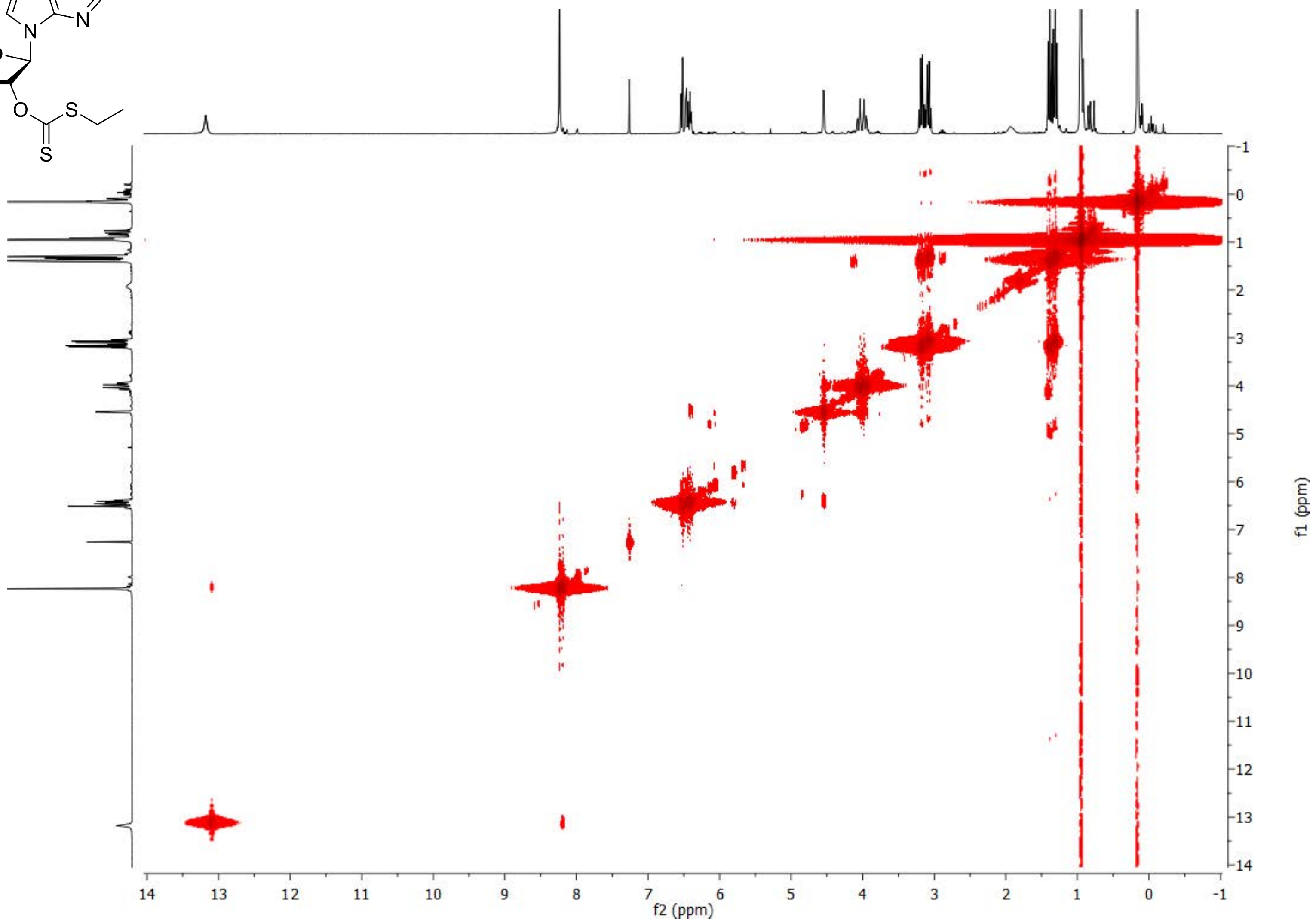

5'-*O*-(*tert*-Butyldimethylsilyl)-2',3'-bis-*O*-[(ethylthio)thiocarbonyl]-β-D-inosine (6f)

HSQC NMR ($CDCl_3$)

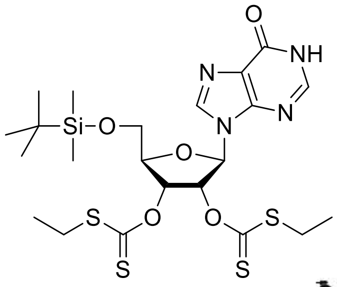

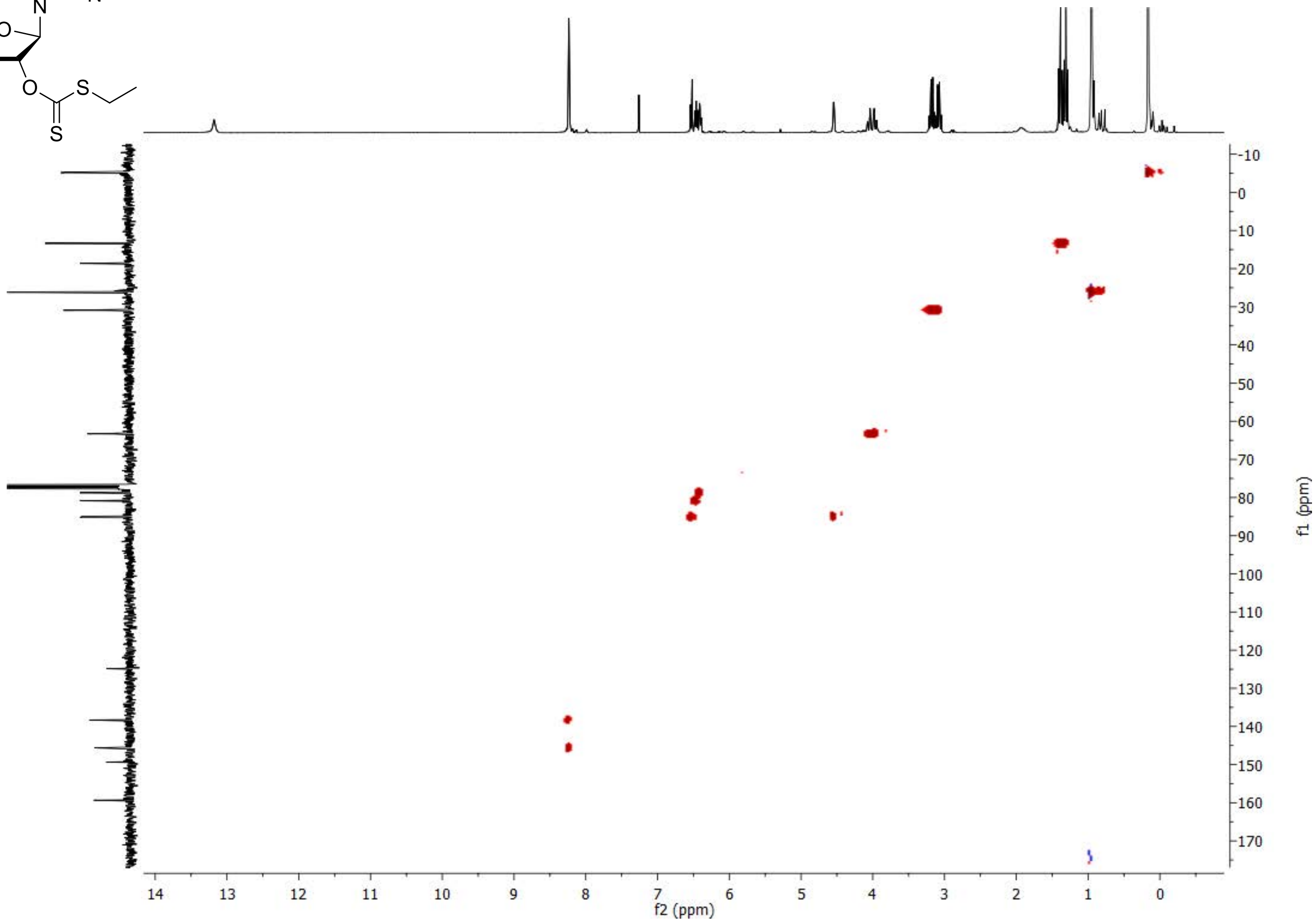

5’-*O*-(*tert*-Butyldimethylsilyl)-2’,3’-didehydro-2’,3’-dideoxy-β-D-uridine (7a)

^{1}H-NMR (300.13 MHz, DMSO-d_6)

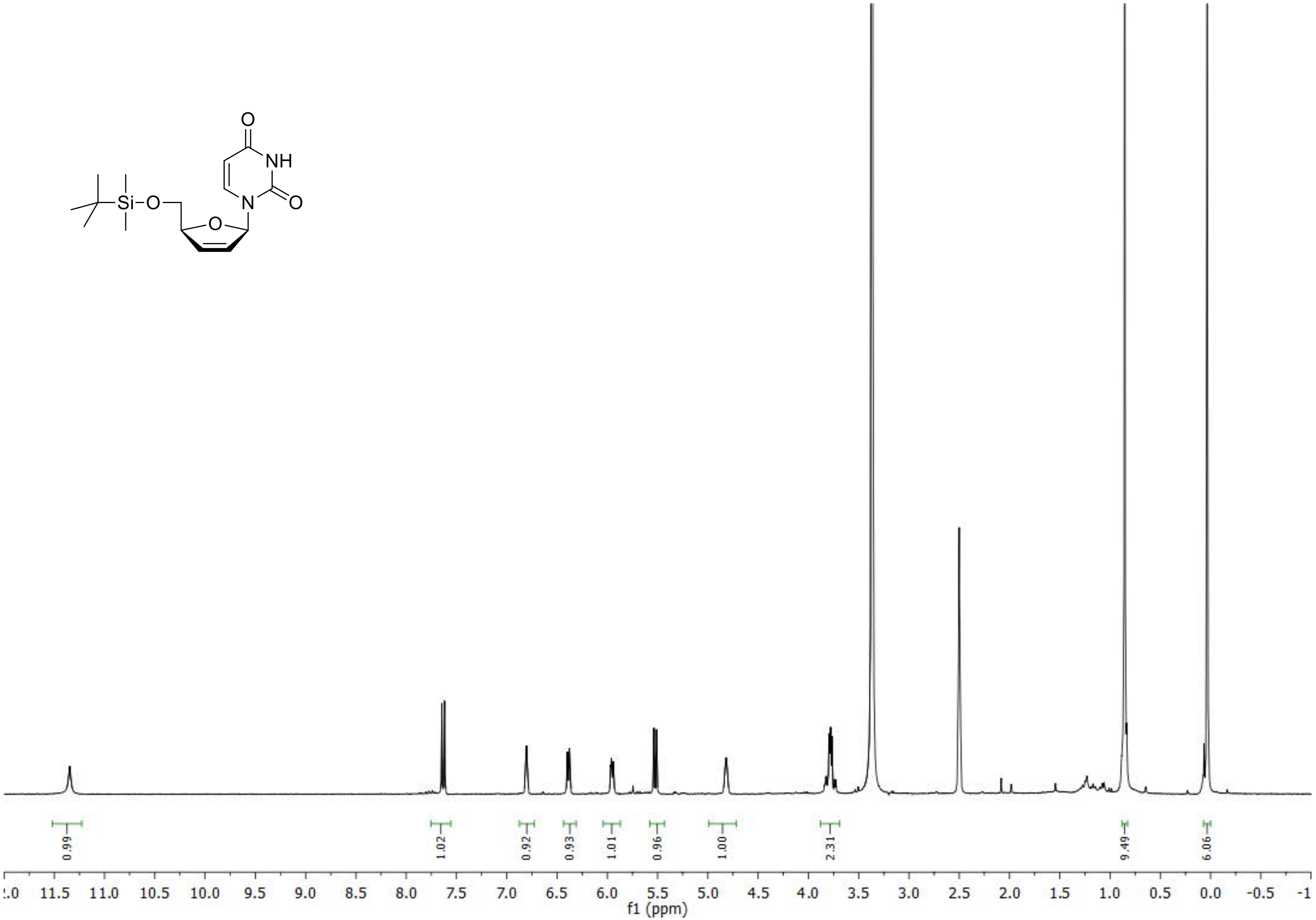

5’-*O*-(*tert*-Butyldimethylsilyl)-2’,3’-didehydro-2’,3’-dideoxy-β-D-uridine (7a)

^{13}C-NMR (75.5 MHz, DMSO-d_6)

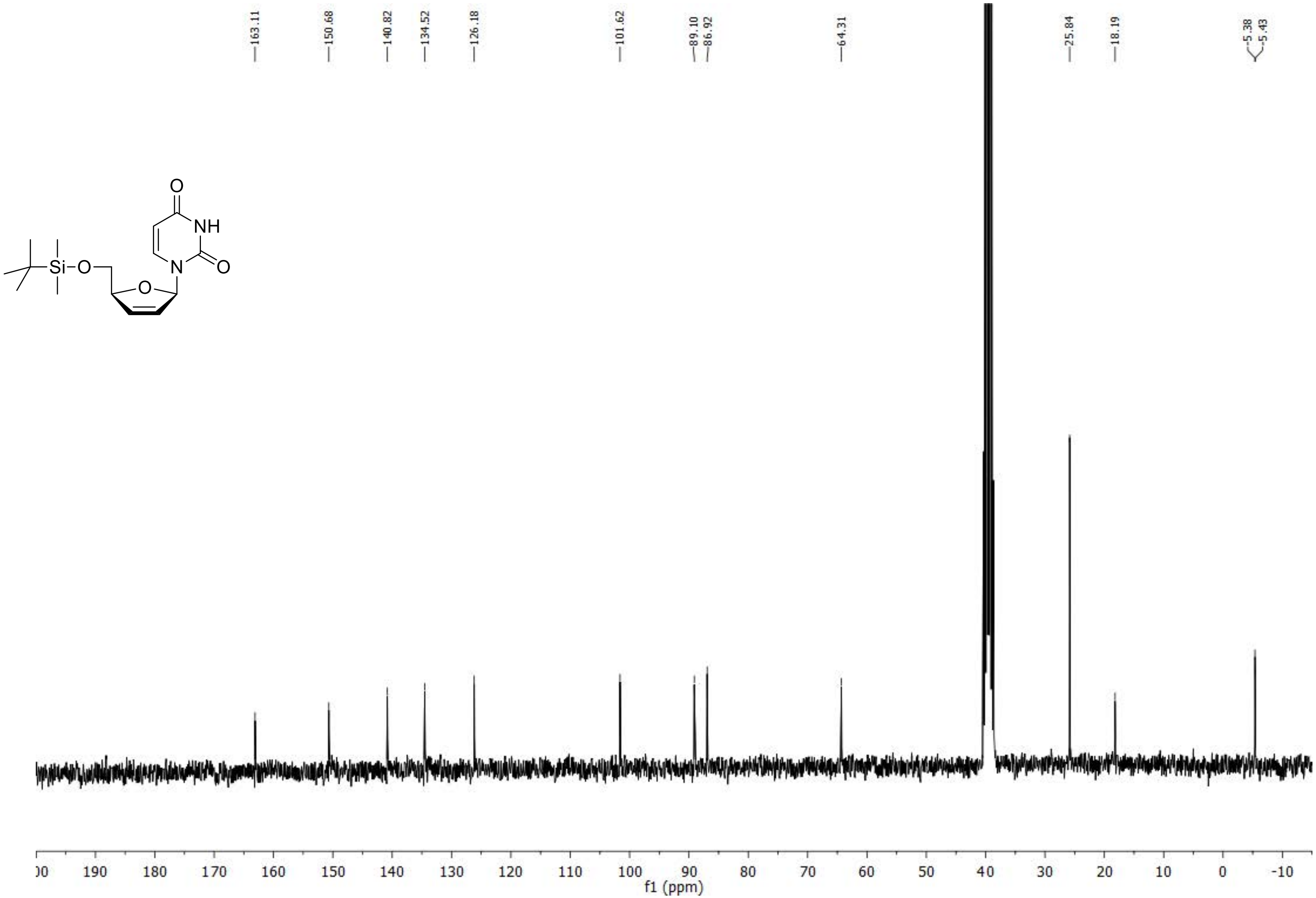

5’-*O*-(*tert*-Butyldimethylsilyl)-2’,3’-didehydro-3’-deoxy-β-D-5-thymidine (7b)

^{1}H-NMR (300.13 MHz, DMSO-d_6)

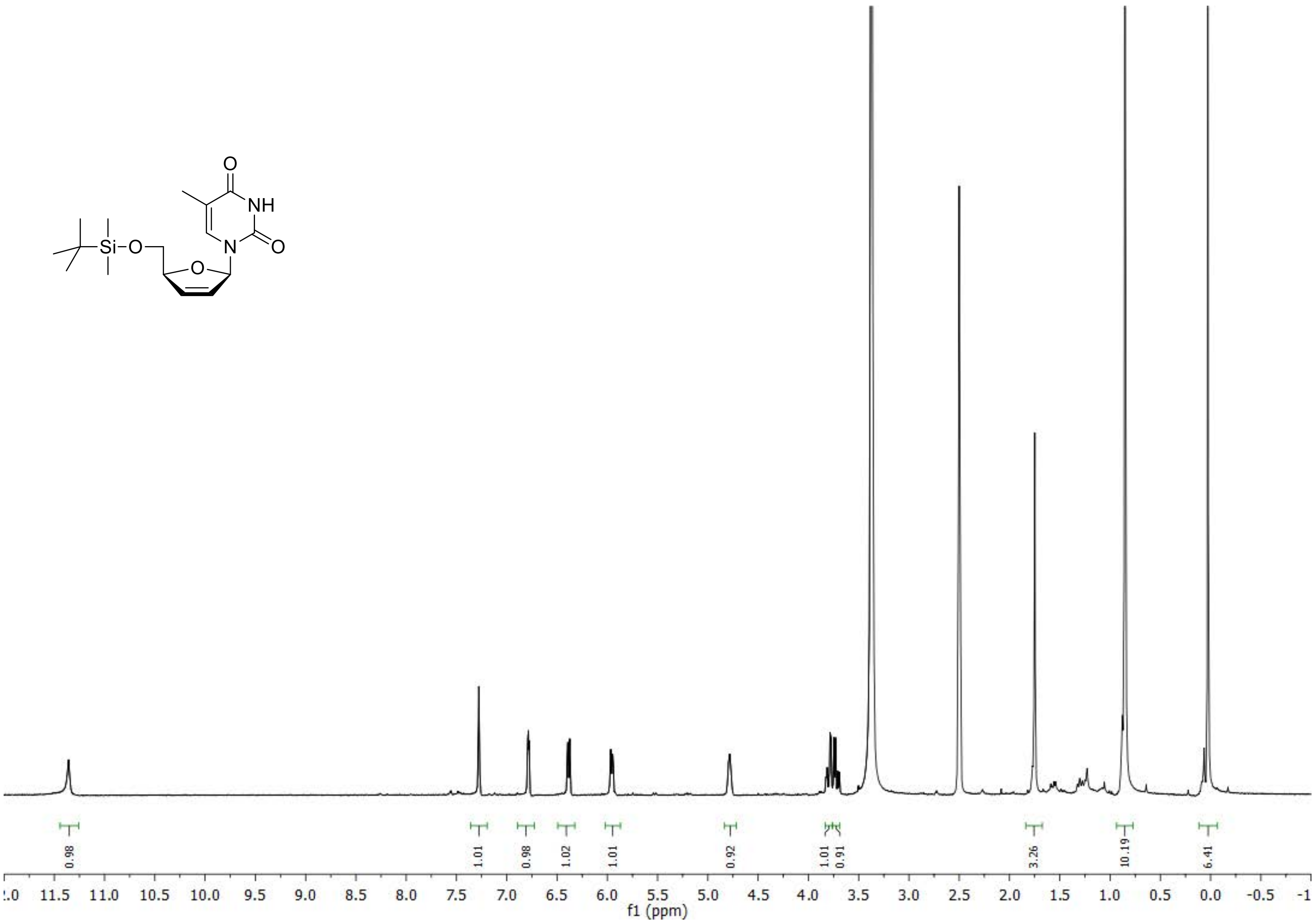

5’-*O*-(*tert*-Butyldimethylsilyl)-2’,3’-didehydro-3’-deoxy-β-D-5-thymidine (7b)

^{13}C-NMR (75.5 MHz, DMSO-d_6)

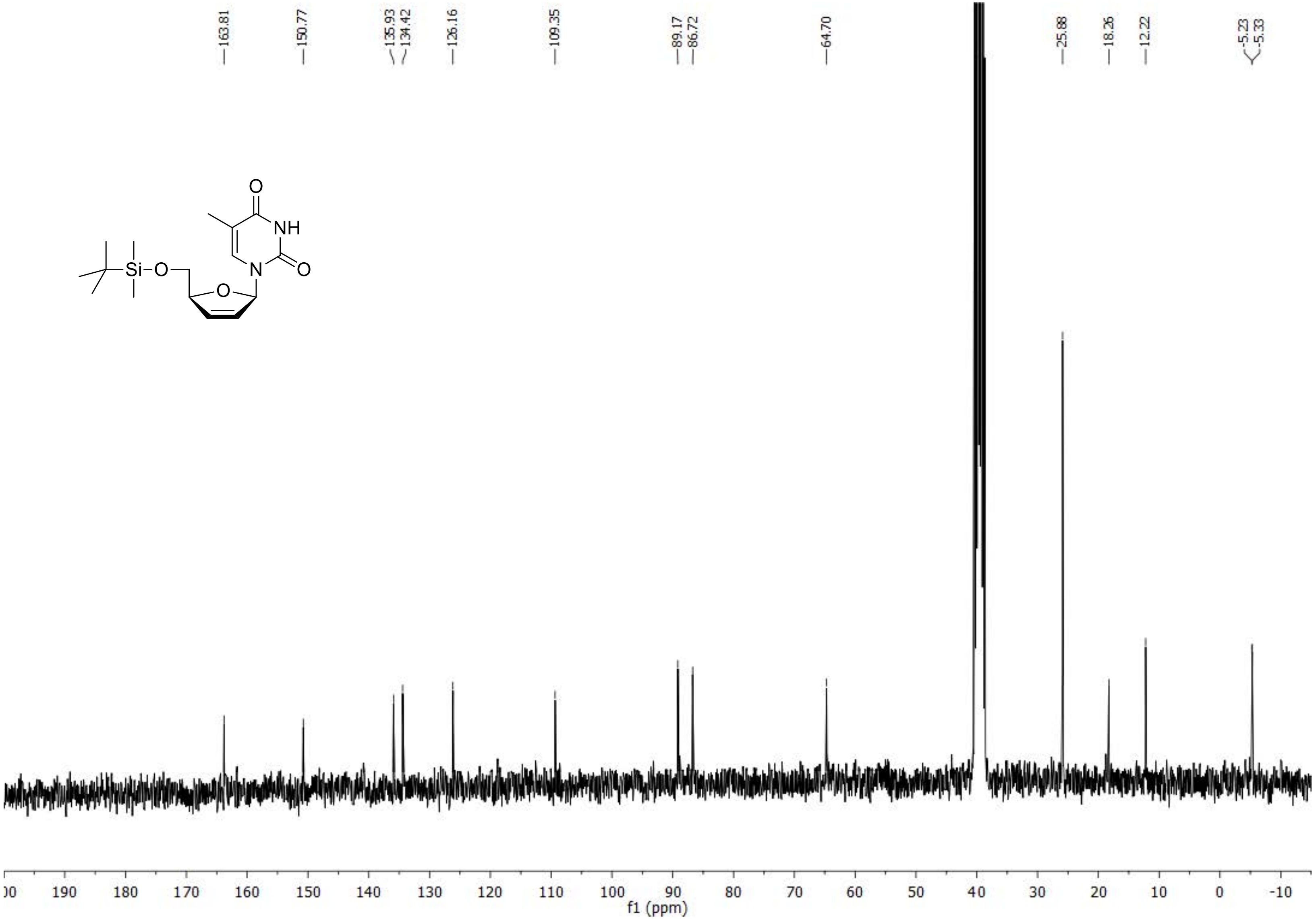

5’-*O*-(*tert*-Butyldimethylsilyl)-2’,3’-didehydro-3’-deoxy-β-D-5-thymidine (7b)

HSQC NMR (DMSO-d_6)

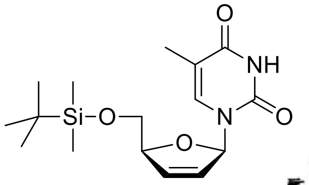

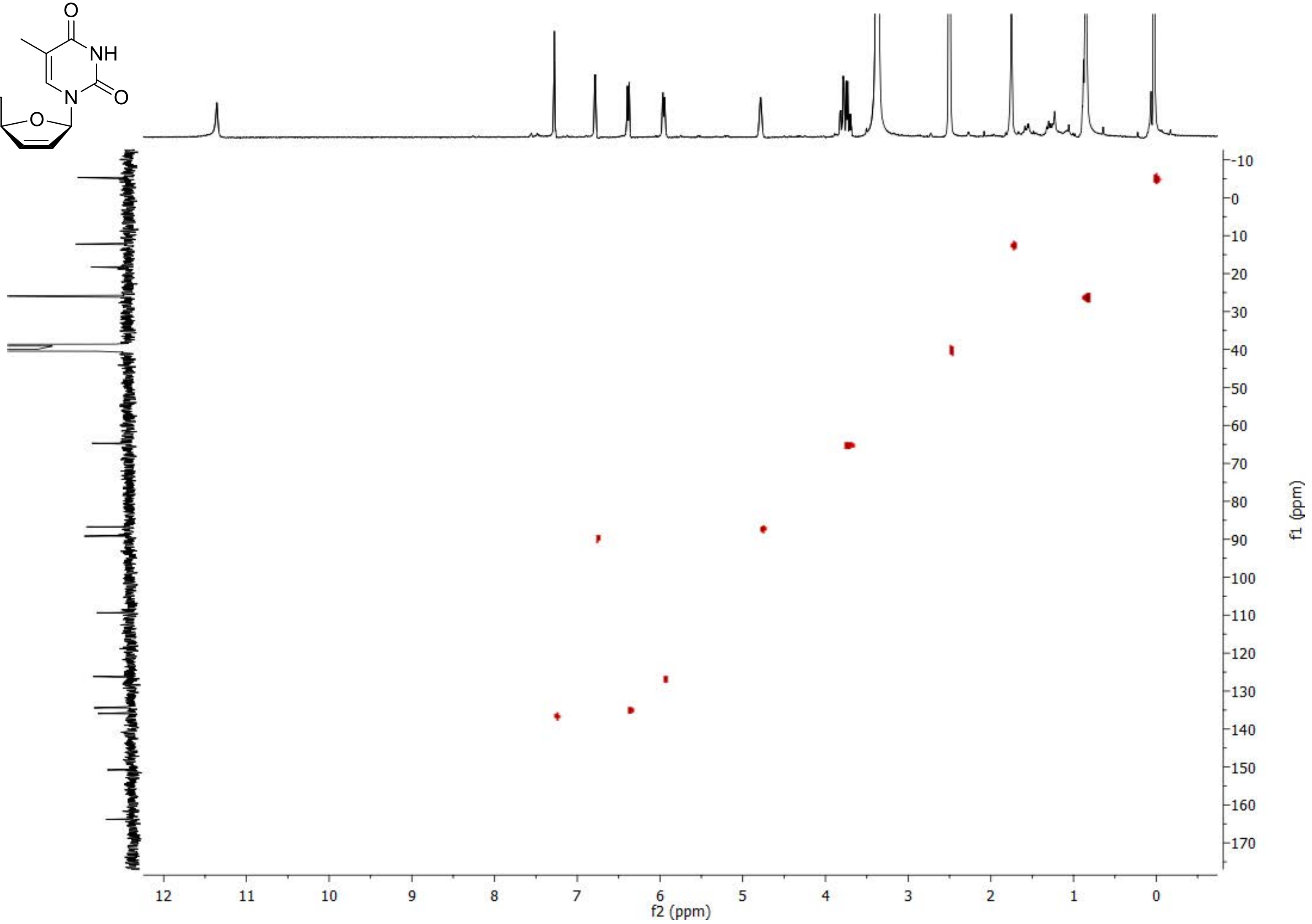

5’-*O*-(*tert*-Butyldimethylsilyl)-2’,3’-didehydro-2’,3’-dideoxy-β-D-cytidine (7c)

^{1}H-NMR (300.13 MHz, $CDCl_3$)

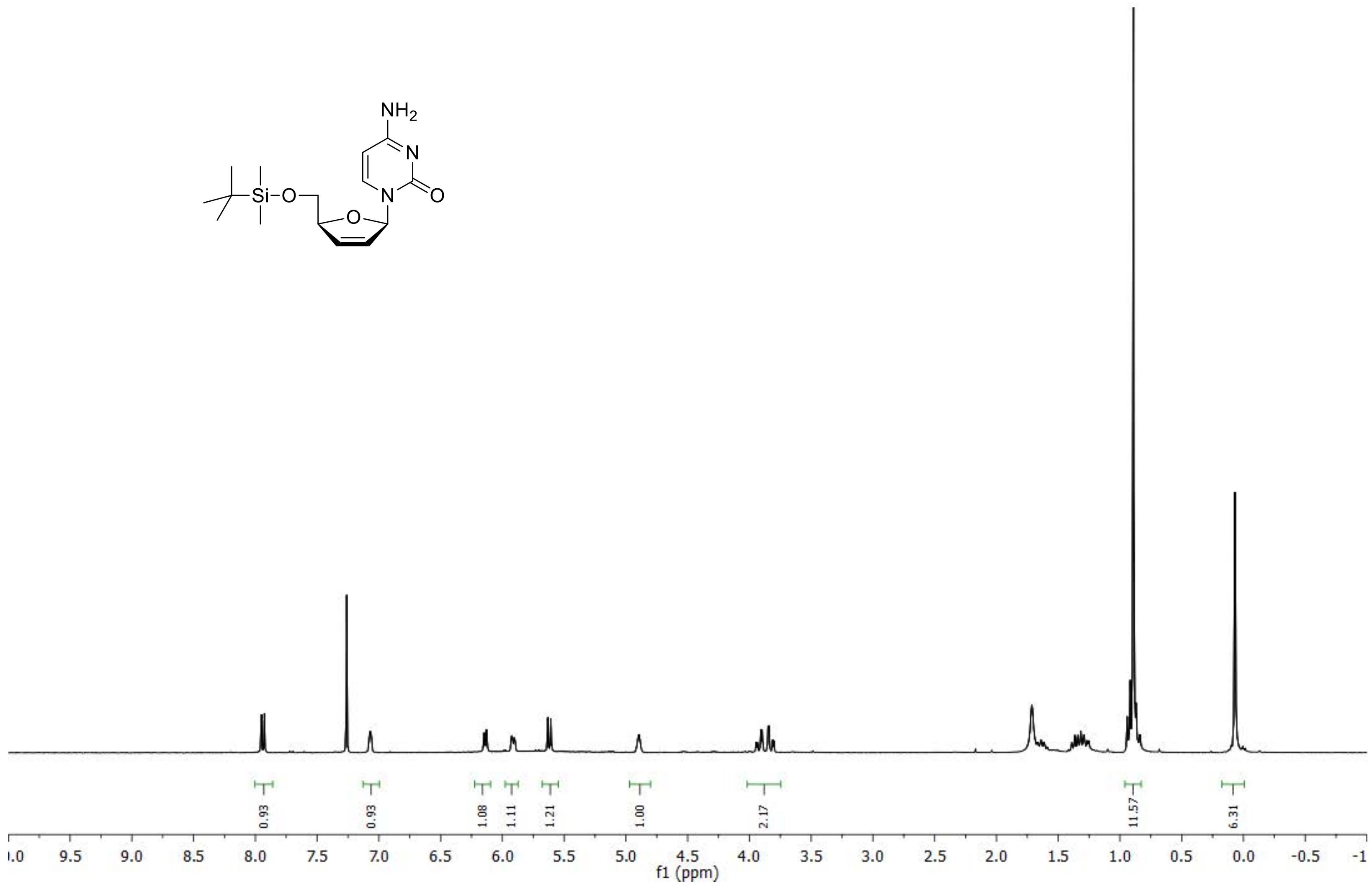

5’-*O*-(*tert*-Butyldimethylsilyl)-2’,3’-didehydro-2’,3’-dideoxy-β-D-cytidine (7c)

^{13}C-NMR (75.5 MHz, $CDCl_3$)

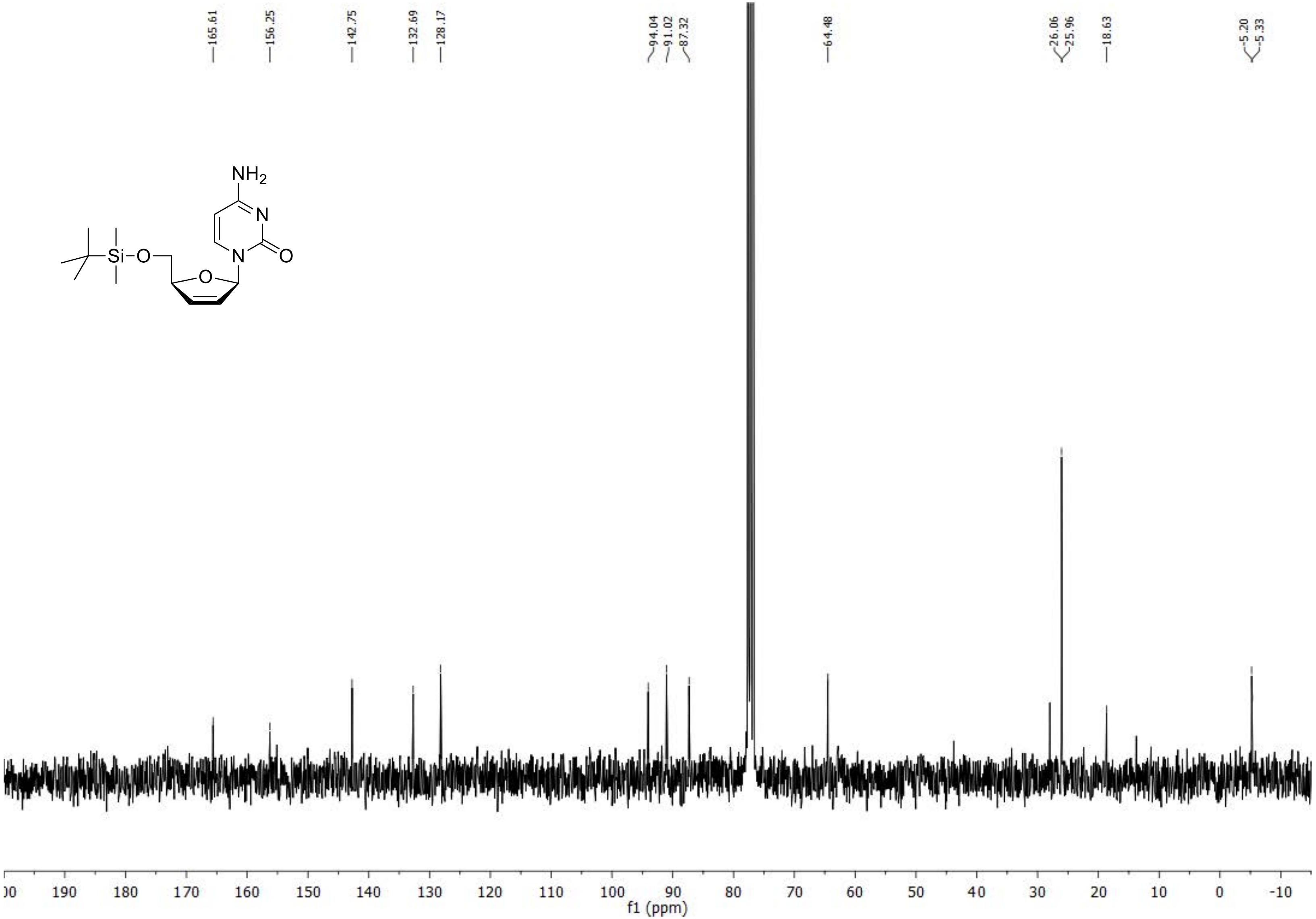

5’-*O*-(*tert*-Butyldimethylsilyl)-2’,3’-didehydro-2’,3’-dideoxy-β-D-cytidine (7c)

DEPT NMR (75.5 MHz, $CDCl_3$)

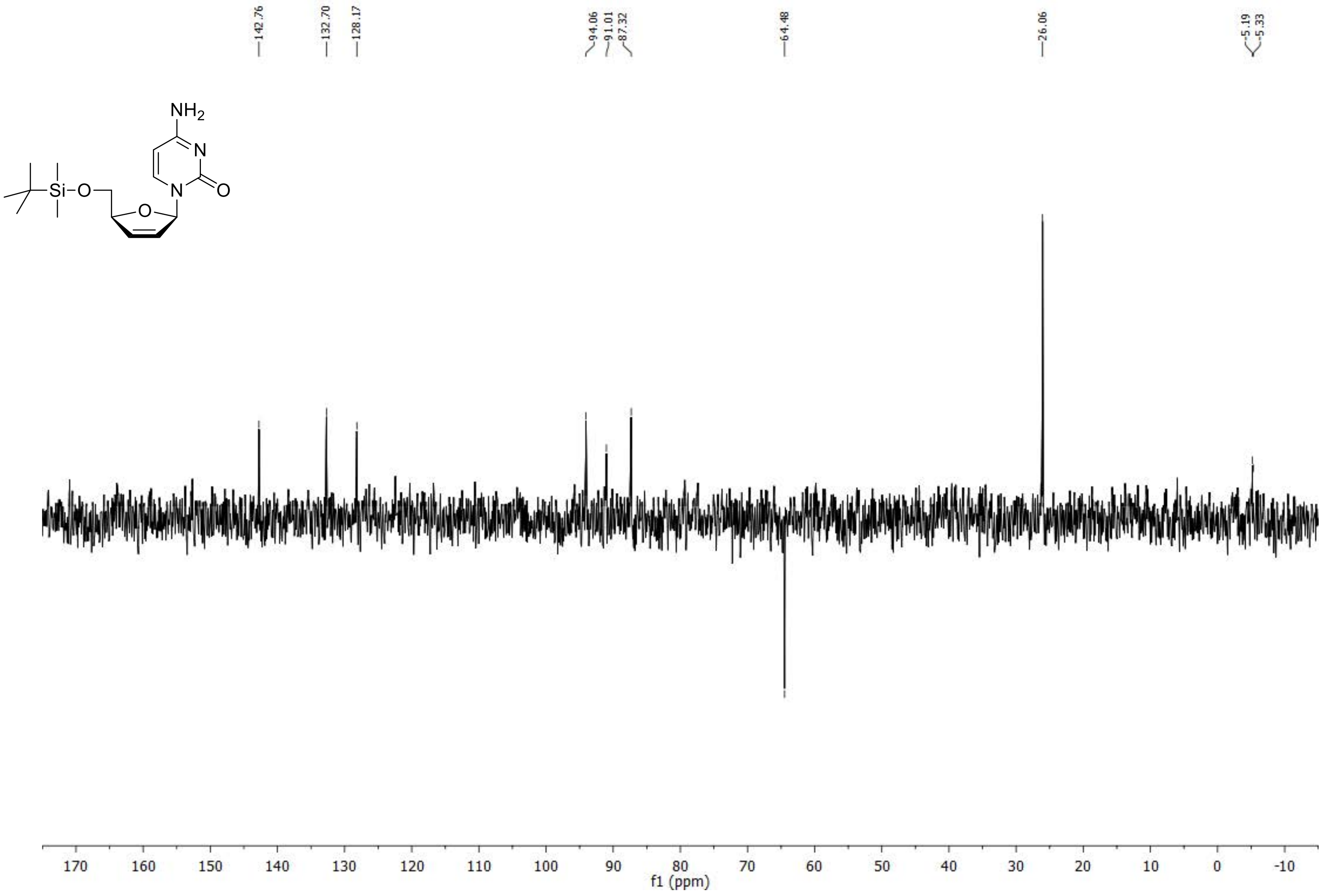

5’-*O*-(*tert*-Butyldimethylsilyl)-2’,3’-didehydro-2’,3’-dideoxy-β-D-cytidine (7c)

COSY NMR ($CDCl_3$)

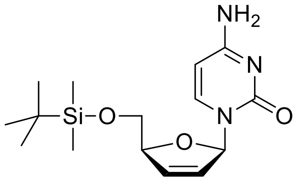

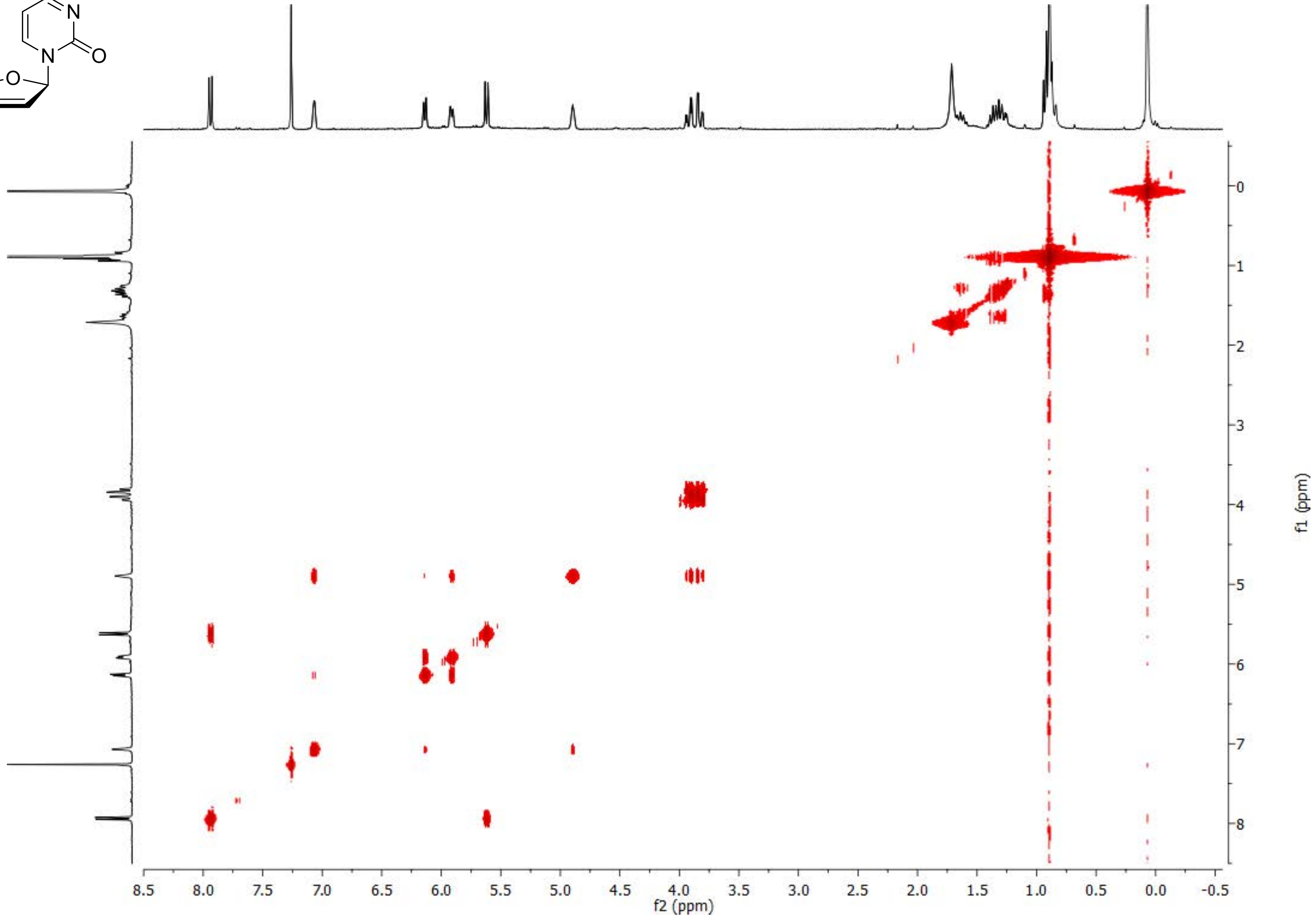

5’-*O*-(*tert*-Butyldimethylsilyl)-2’,3’-didehydro-2’,3’-dideoxy-β-D-cytidine (7c)

HSQC NMR ($CDCl_3$)

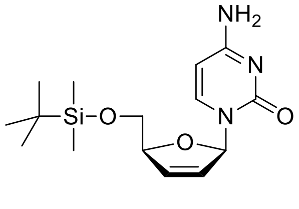

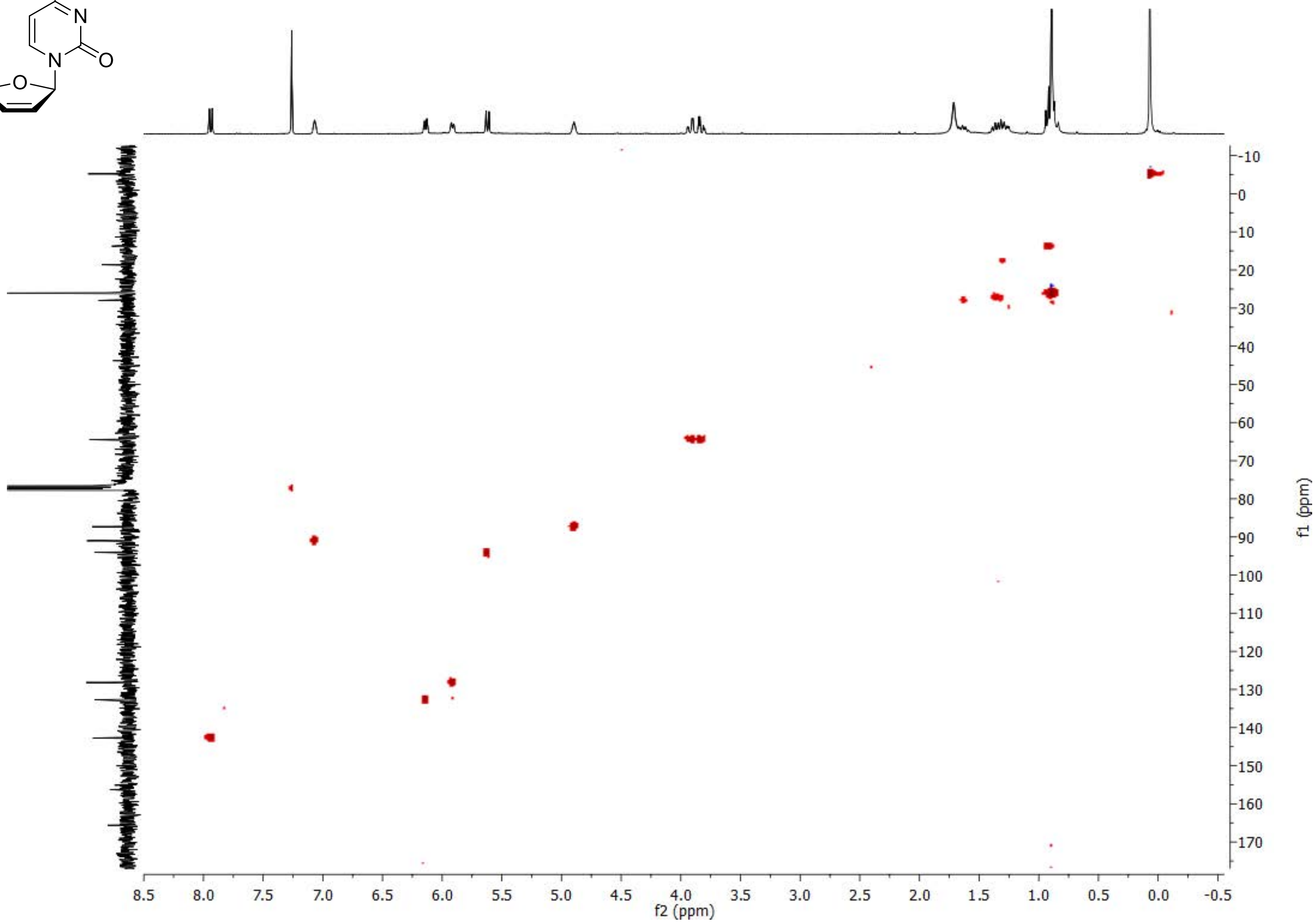

5’-*O*-(*tert*-Butyldimethylsilyl)-2’,3’-didehydro-2’,3’-dideoxy-β-D-cytidine (7c)

HMBC NMR ($CDCl_3$)

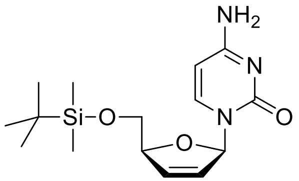

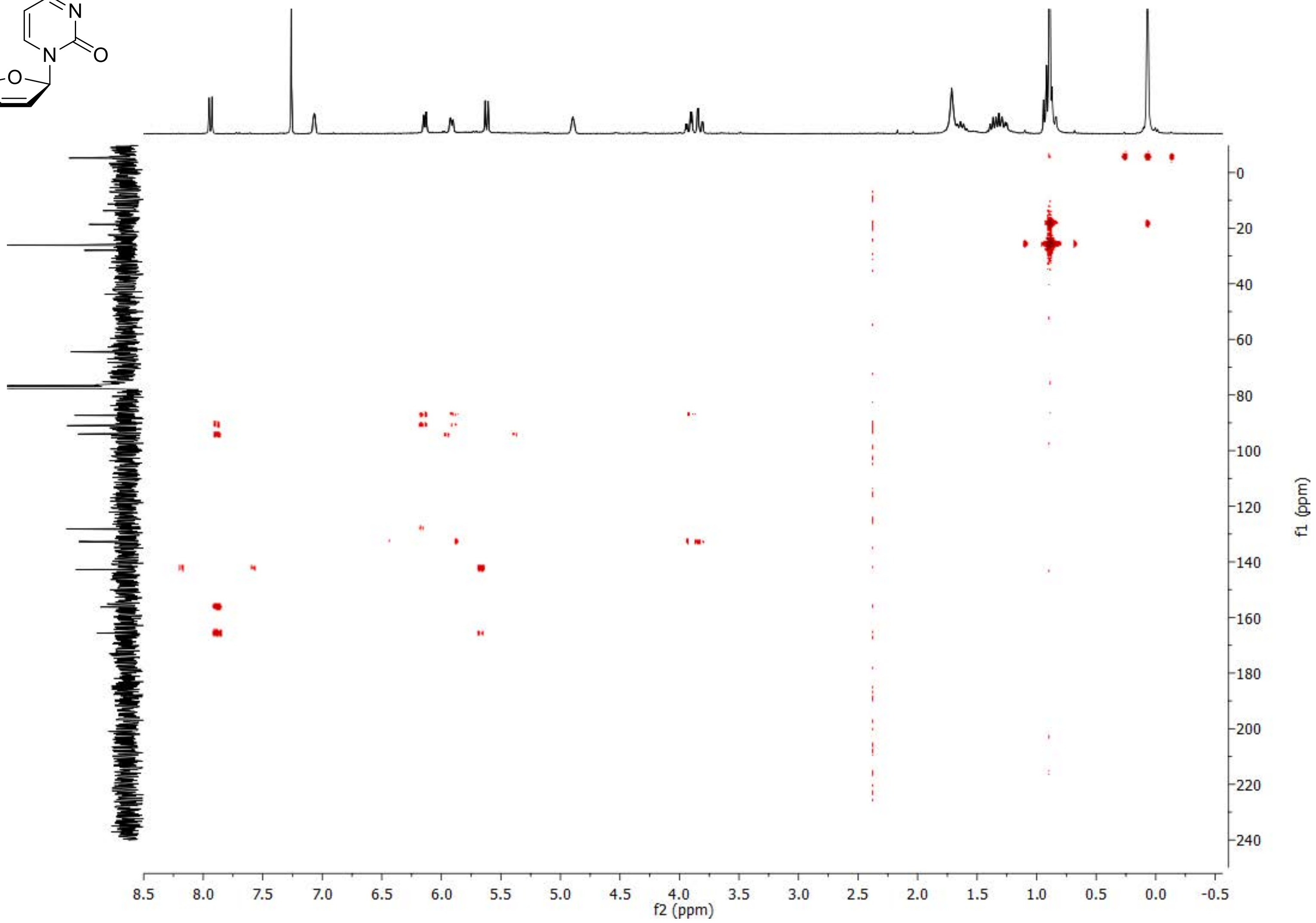

N^4-Benzoyl-5'-*O*-(*tert*-butyldimethylsilyl)-2',3'-didehydro-2',3'-dideoxy-β-D-cytidine (7d)

^{1}H-NMR (300.13 MHz, $CDCl_3$)

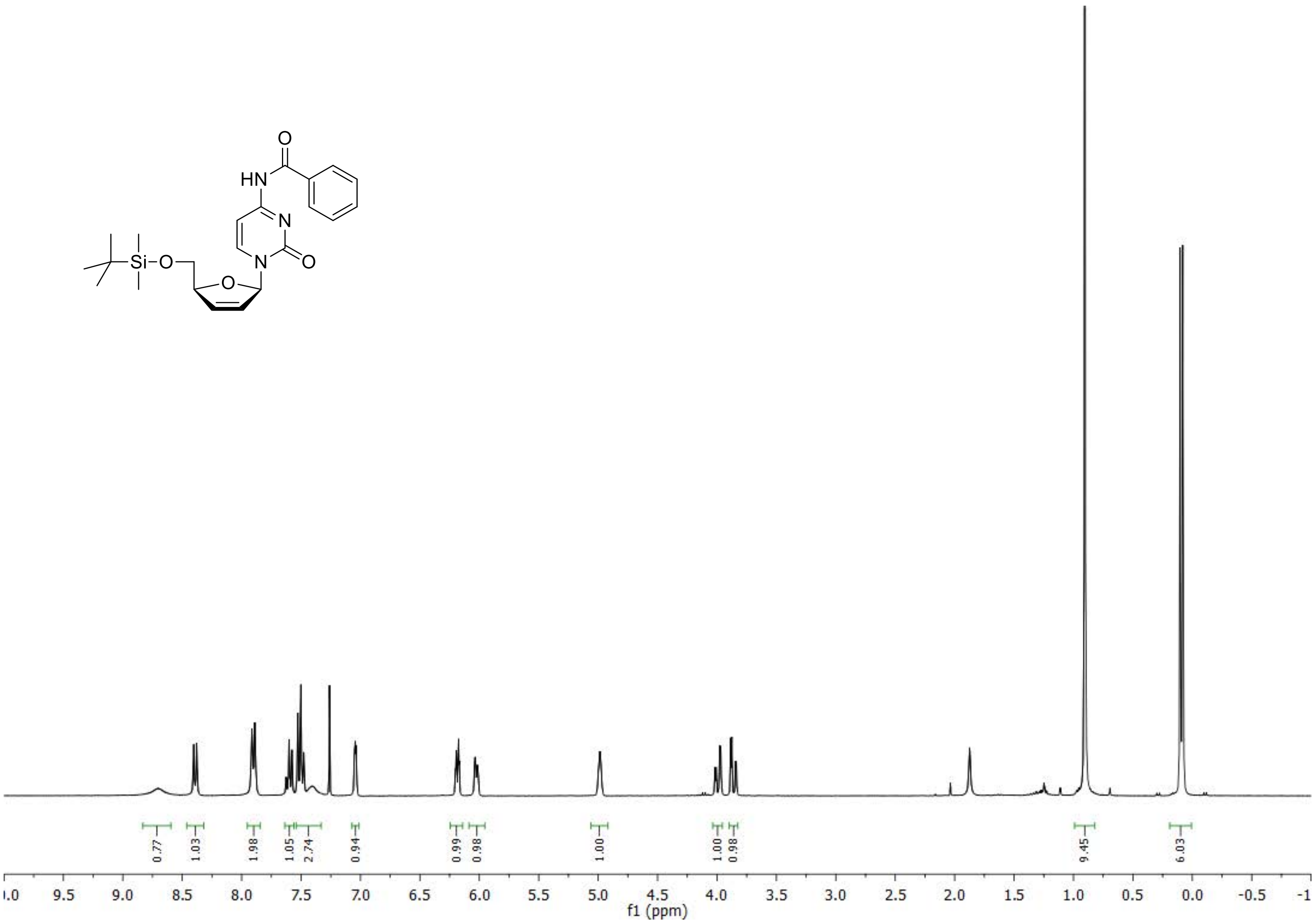

N^4-Benzoyl-5'-*O*-(*tert*-butyldimethylsilyl)-2',3'-didehydro-2',3'-dideoxy-β-D-cytidine (7d)

^{13}C-NMR (75.5 MHz, $CDCl_3$)

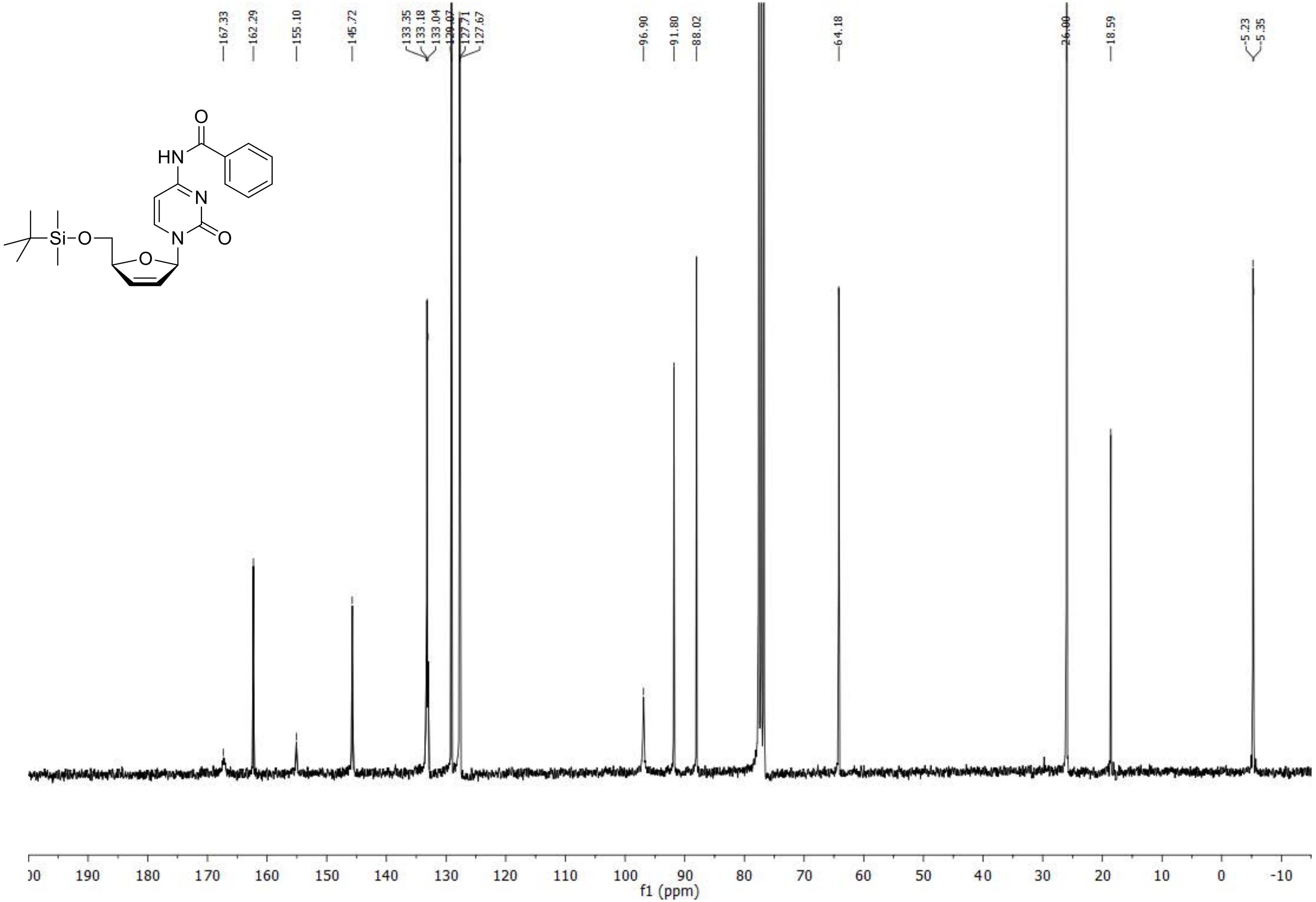

N^4-Benzoyl-5'-*O*-(*tert*-butyldimethylsilyl)-2',3'-didehydro-2',3'-dideoxy-β-D-cytidine (7d)

DEPT NMR (75.5 MHz, $CDCl_3$)

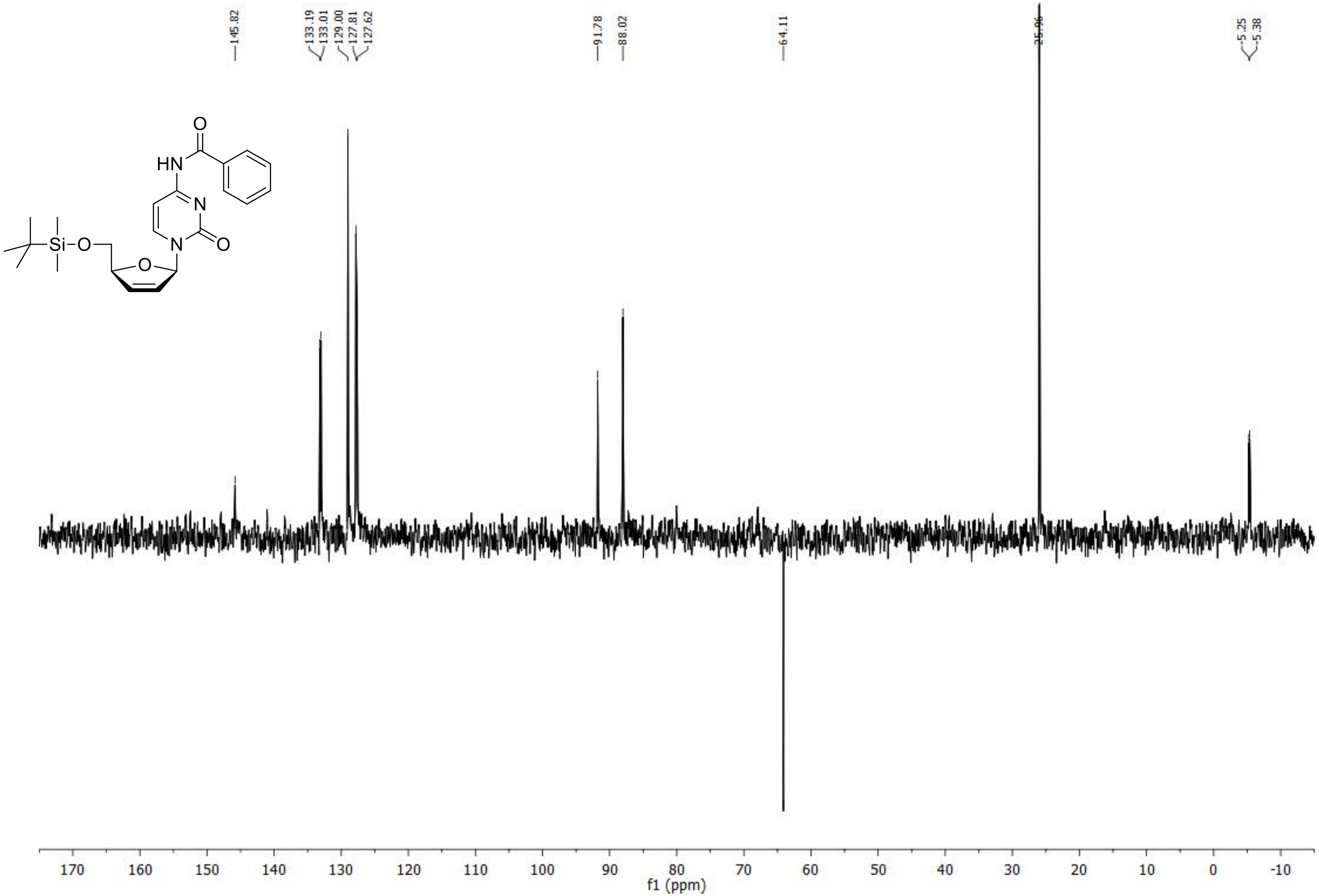

N^4-Benzoyl-5'-*O*-(*tert*-butyldimethylsilyl)-2',3'-didehydro-2',3'-dideoxy-β-D-cytidine (7d)

COSY NMR ($CDCl_3$)

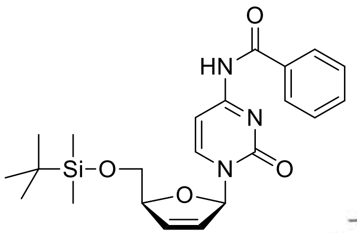

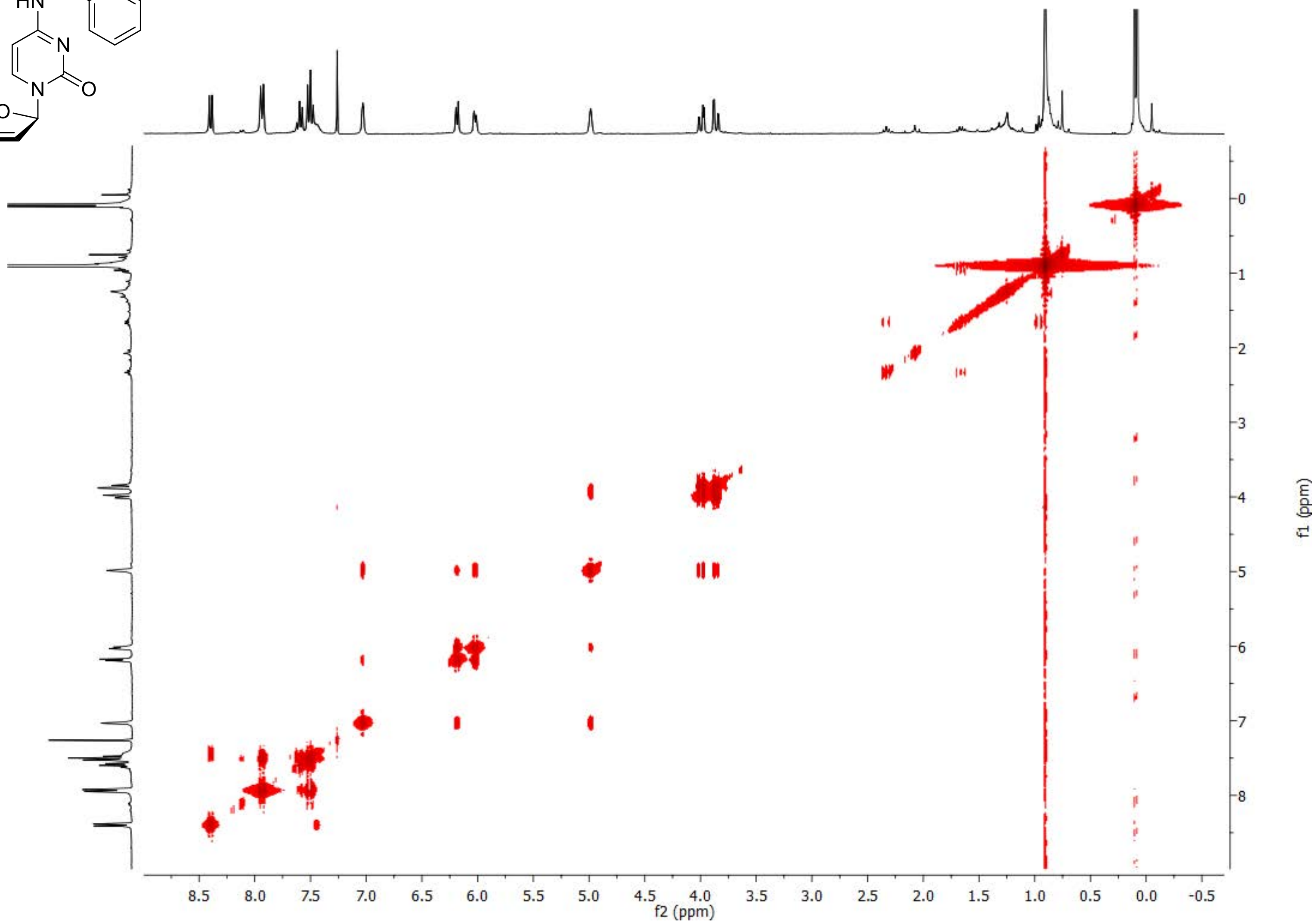

N^4-Benzoyl-5’-*O*-(*tert*-butyldimethylsilyl)-2’,3’-didehydro-2’,3’-dideoxy-β-D-cytidine (7d)

HSQC NMR ($CDCl_3$)

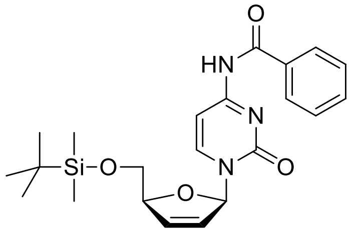

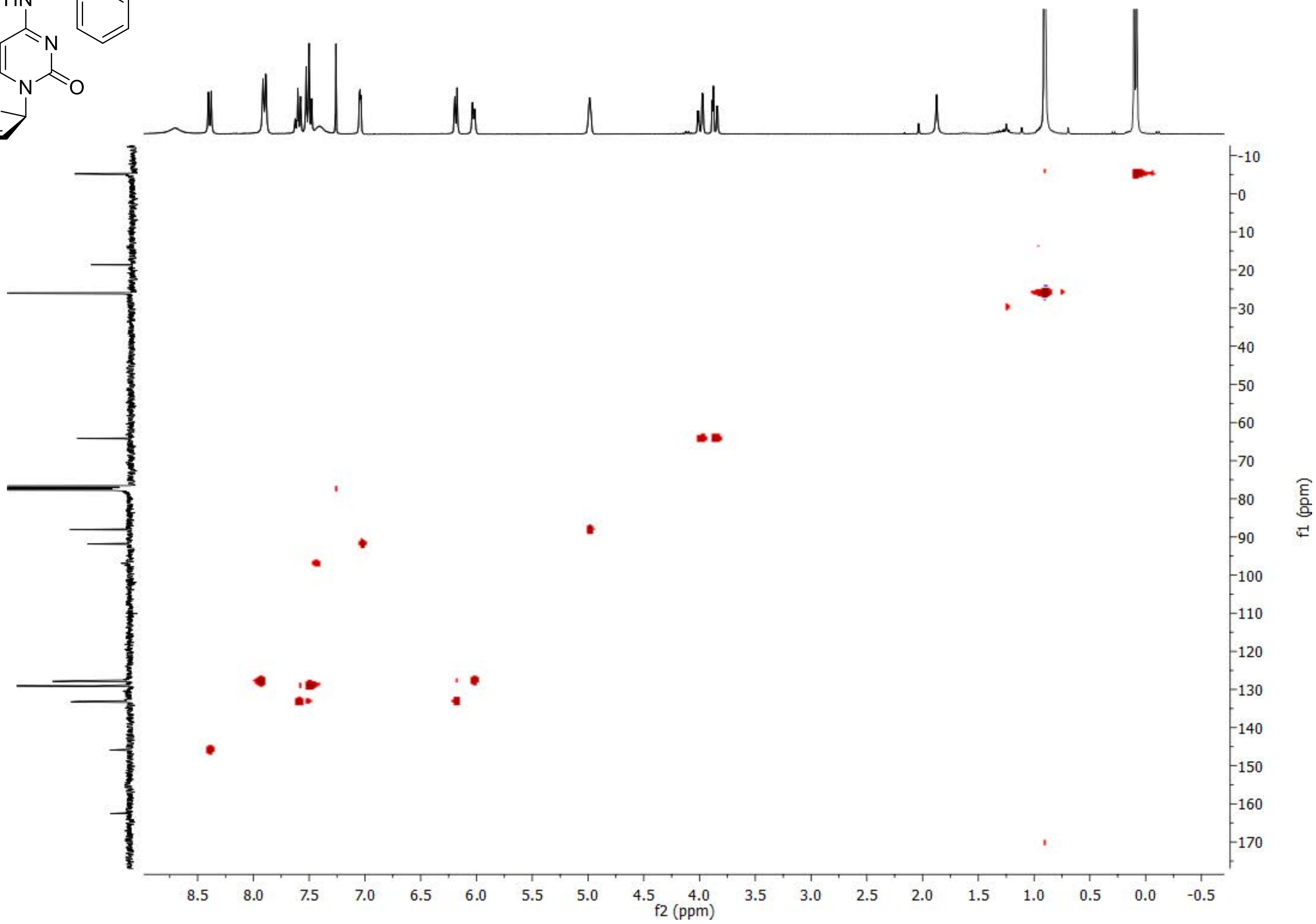

N^4-Benzoyl-5'-*O*-(*tert*-butyldimethylsilyl)-2',3'-didehydro-2',3'-dideoxy-β-D-cytidine (7d)

HMBC NMR ($CDCl_3$)

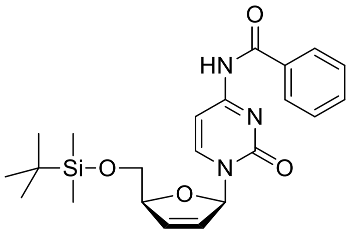

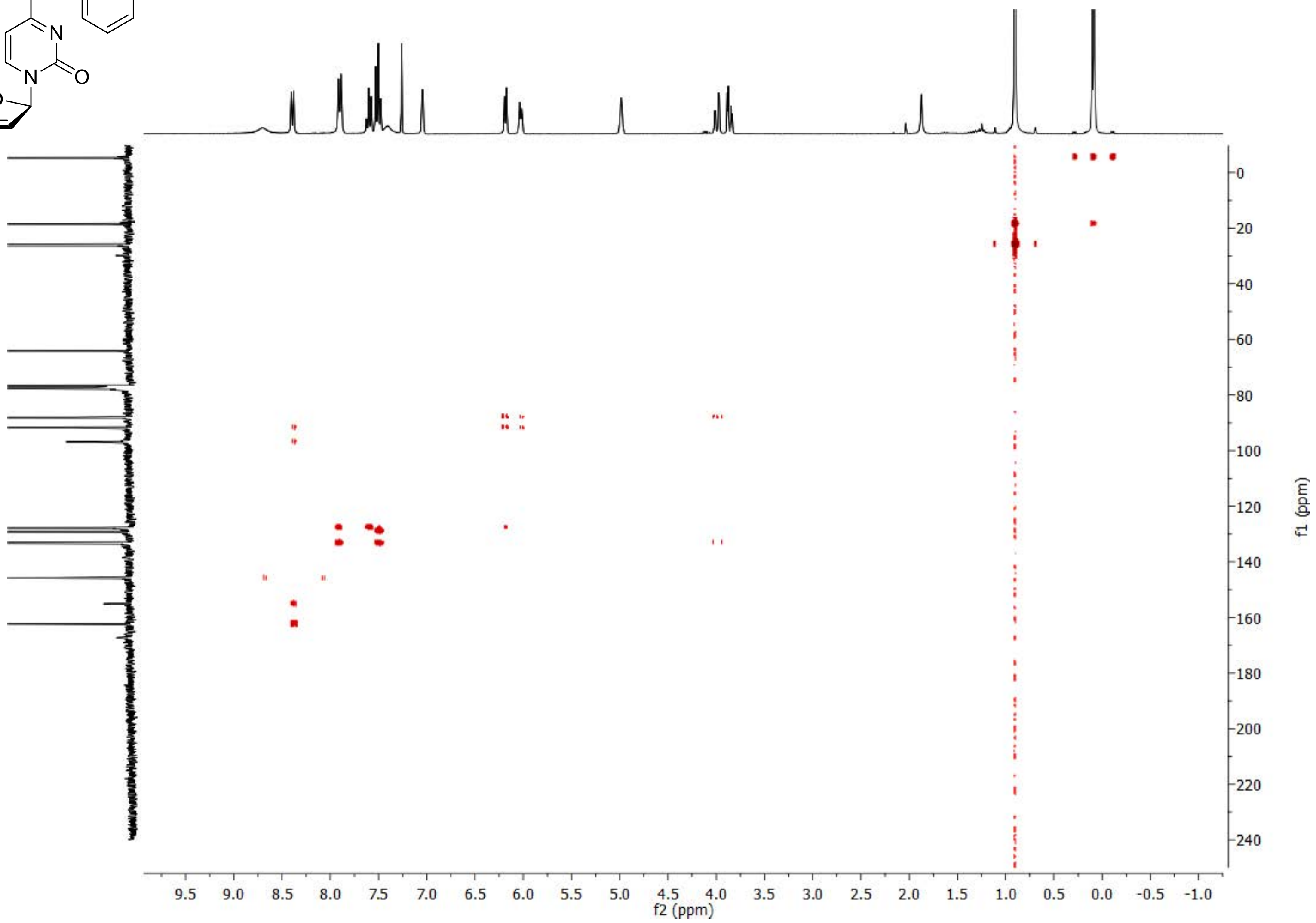

5’-*O*-(*tert*-Butyldimethylsilyl)-2’,3’-didehydro-2’,3’-dideoxy-β-D-adenosine (7e)

^{1}H-NMR (300.13 MHz, $CDCl_3$)

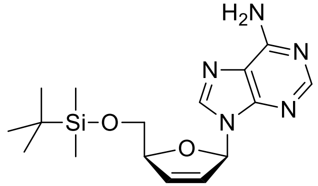

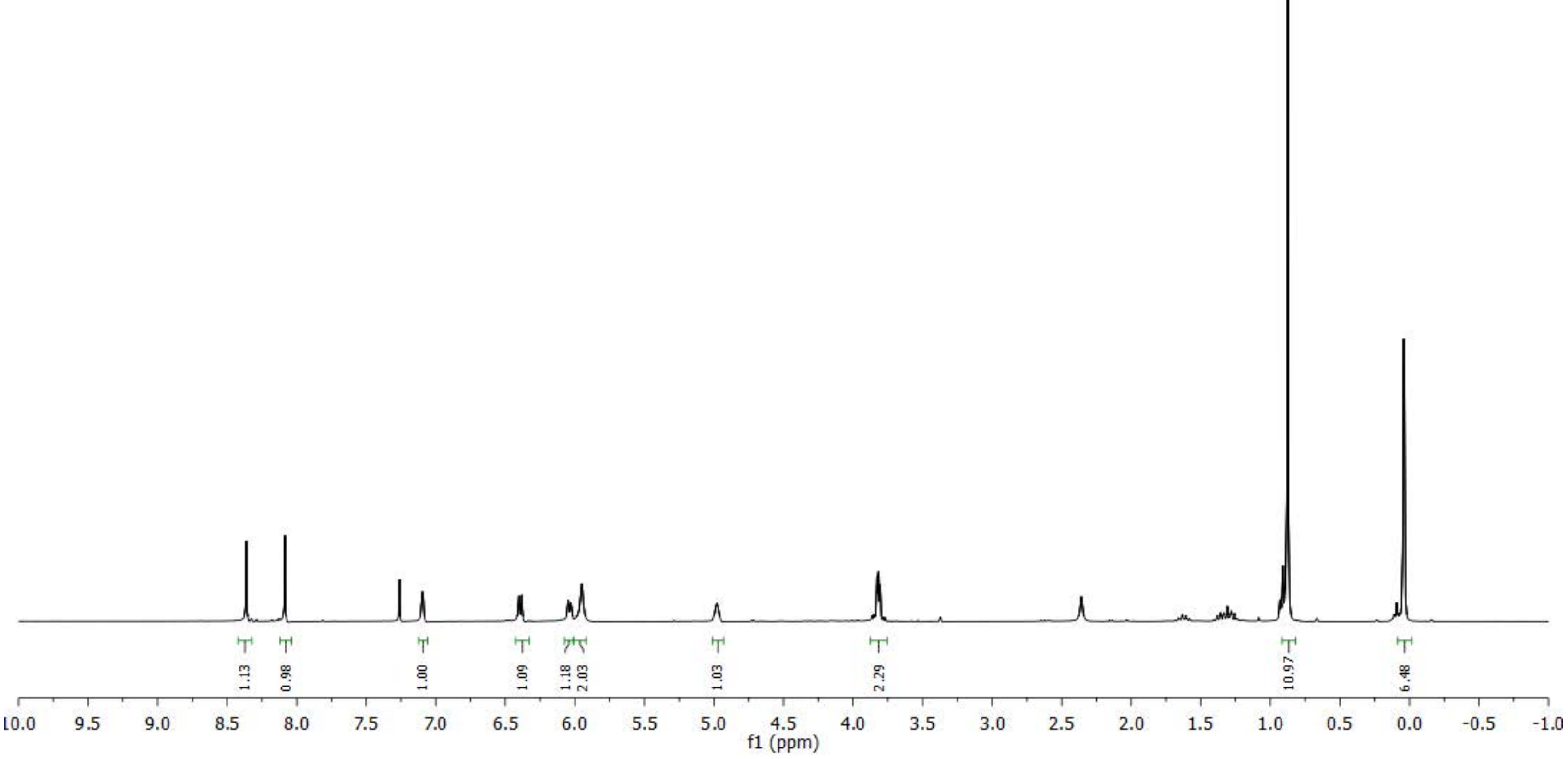

5’-*O*-(*tert*-Butyldimethylsilyl)-2’,3’-didehydro-2’,3’-dideoxy-β-D-adenosine (7e)

^{13}C-NMR (75.5 MHz, $CDCl_3$)

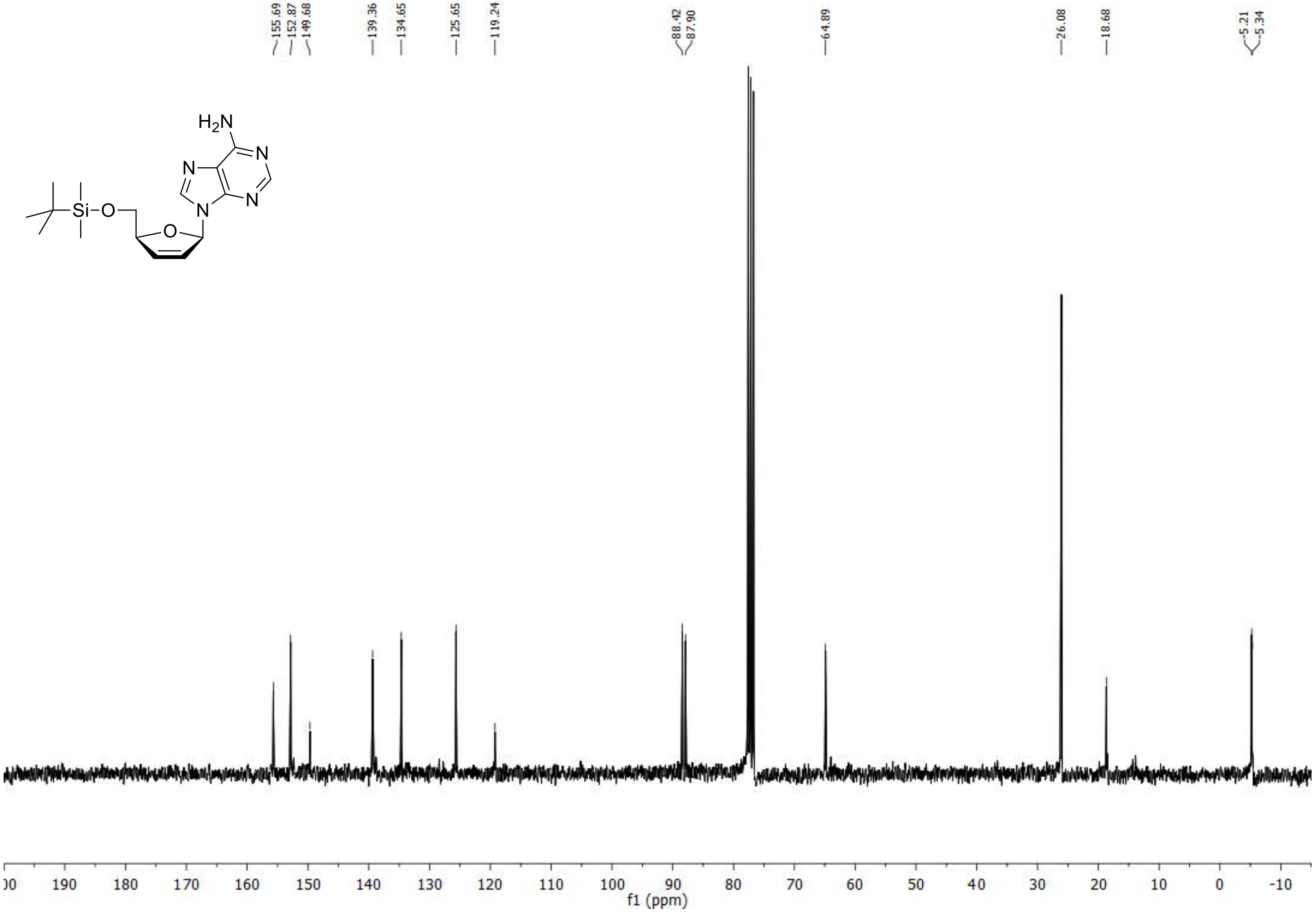

5’-*O*-(*tert*-Butyldimethylsilyl)-2’,3’-didehydro-2’,3’-dideoxy-β-D-adenosine (7e)

DEPT NMR (75.5 MHz, $CDCl_3$)

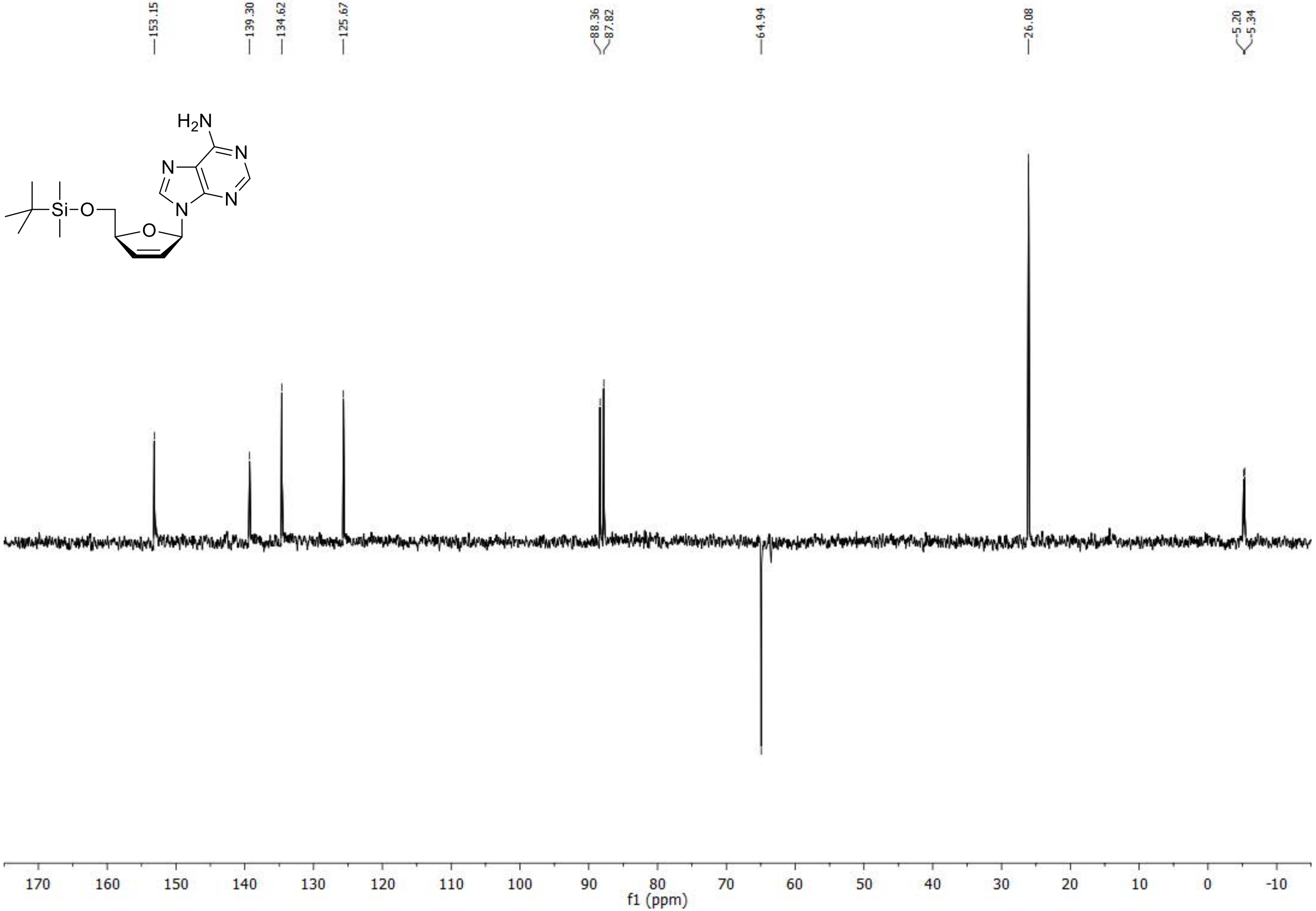

5’-*O*-(*tert*-Butyldimethylsilyl)-2’,3’-didehydro-2’,3’-dideoxy-β-D-adenosine (7e)

COSY NMR ($CDCl_3$)

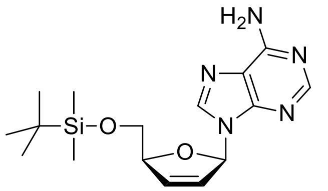

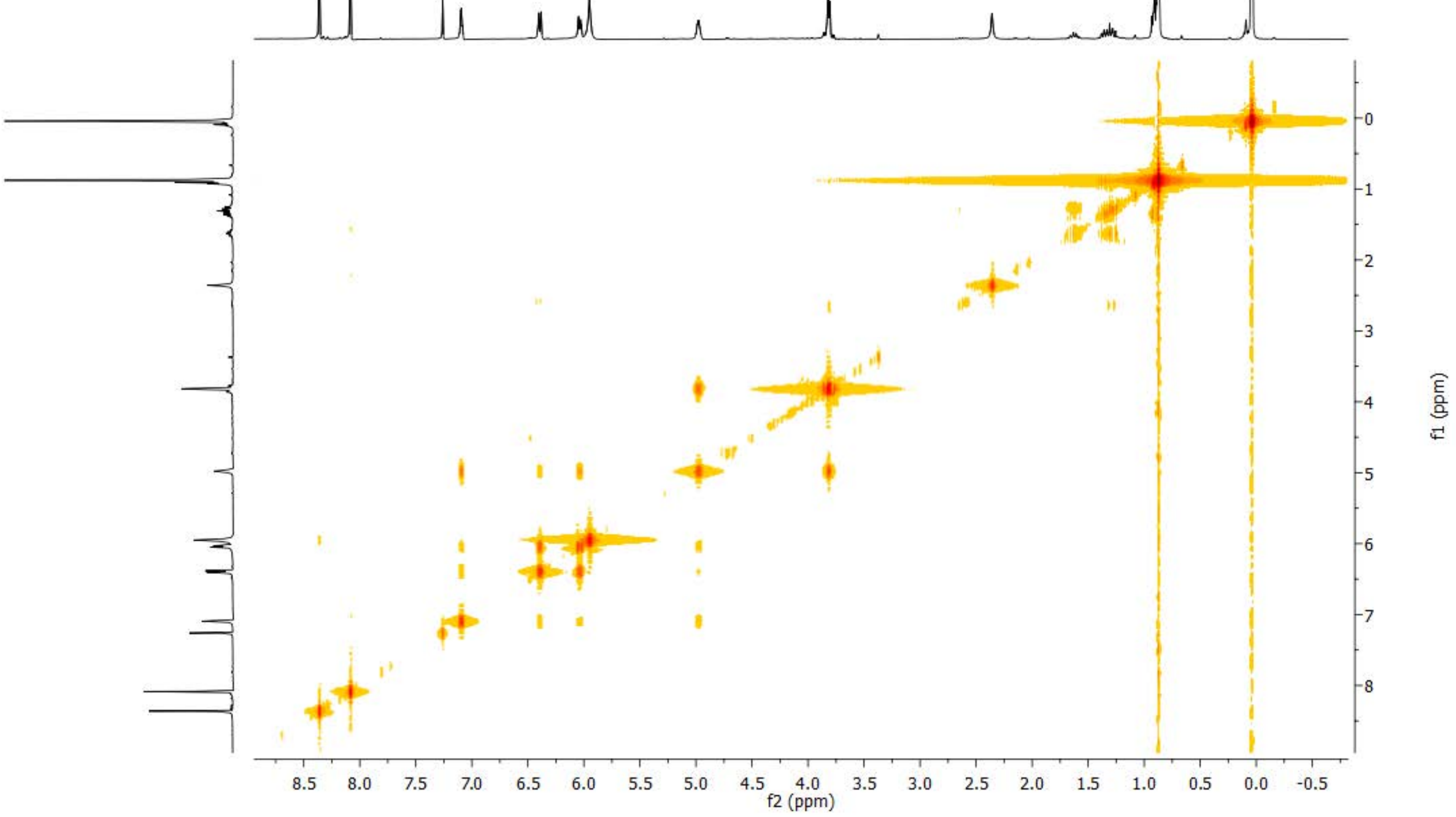

5'-*O*-(*tert*-Butyldimethylsilyl)-2',3'-didehydro-2',3'-dideoxy-β-D-adenosine (7e)

HSQC NMR ($CDCl_3$)

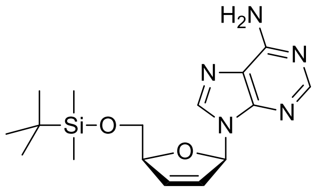

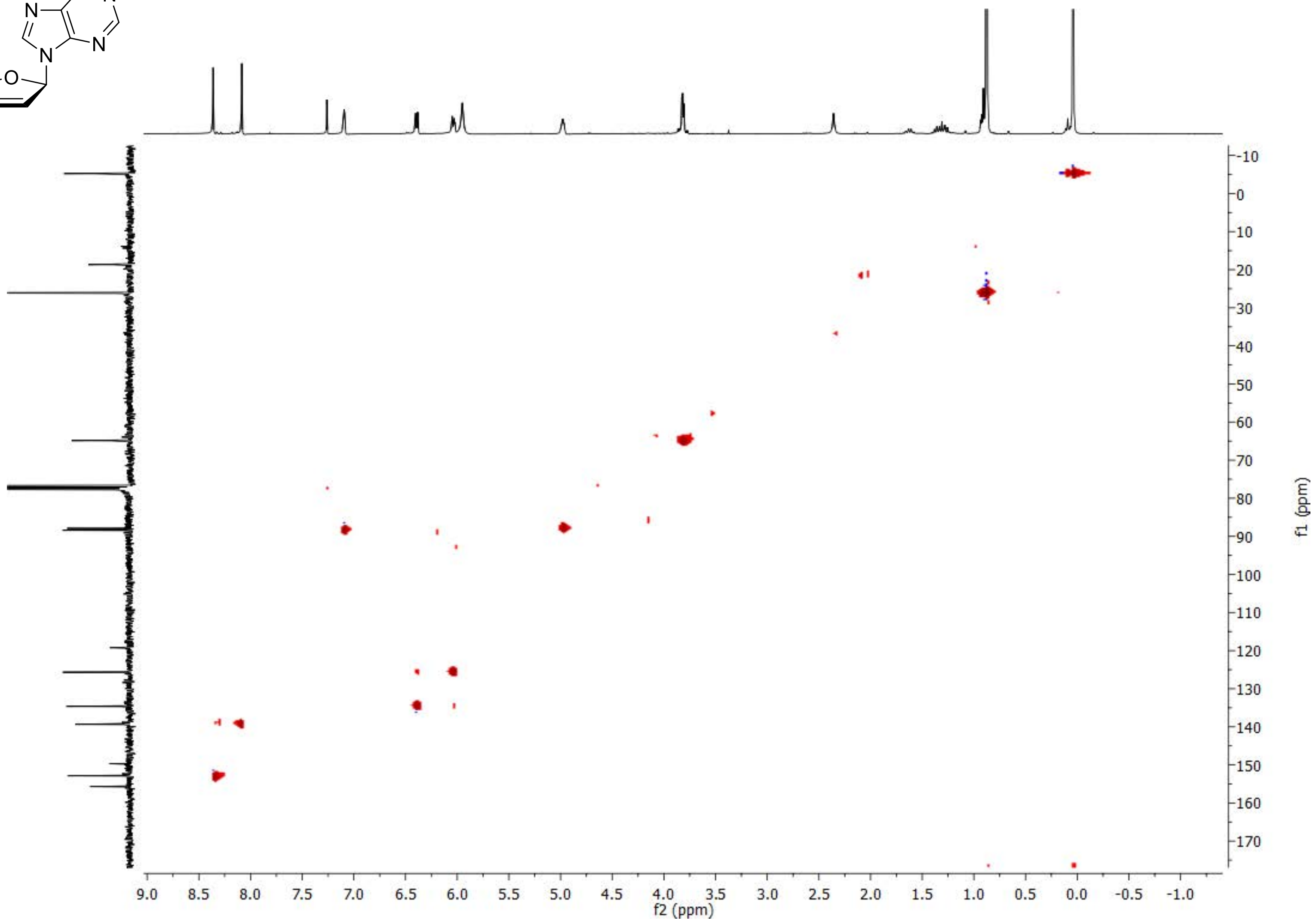

5’-*O*-(*tert*-Butyldimethylsilyl)-2’,3’-didehydro-2’,3’-dideoxy-β-D-inosine (7f)

^{1}H-NMR (300.13 MHz, $CDCl_3$)

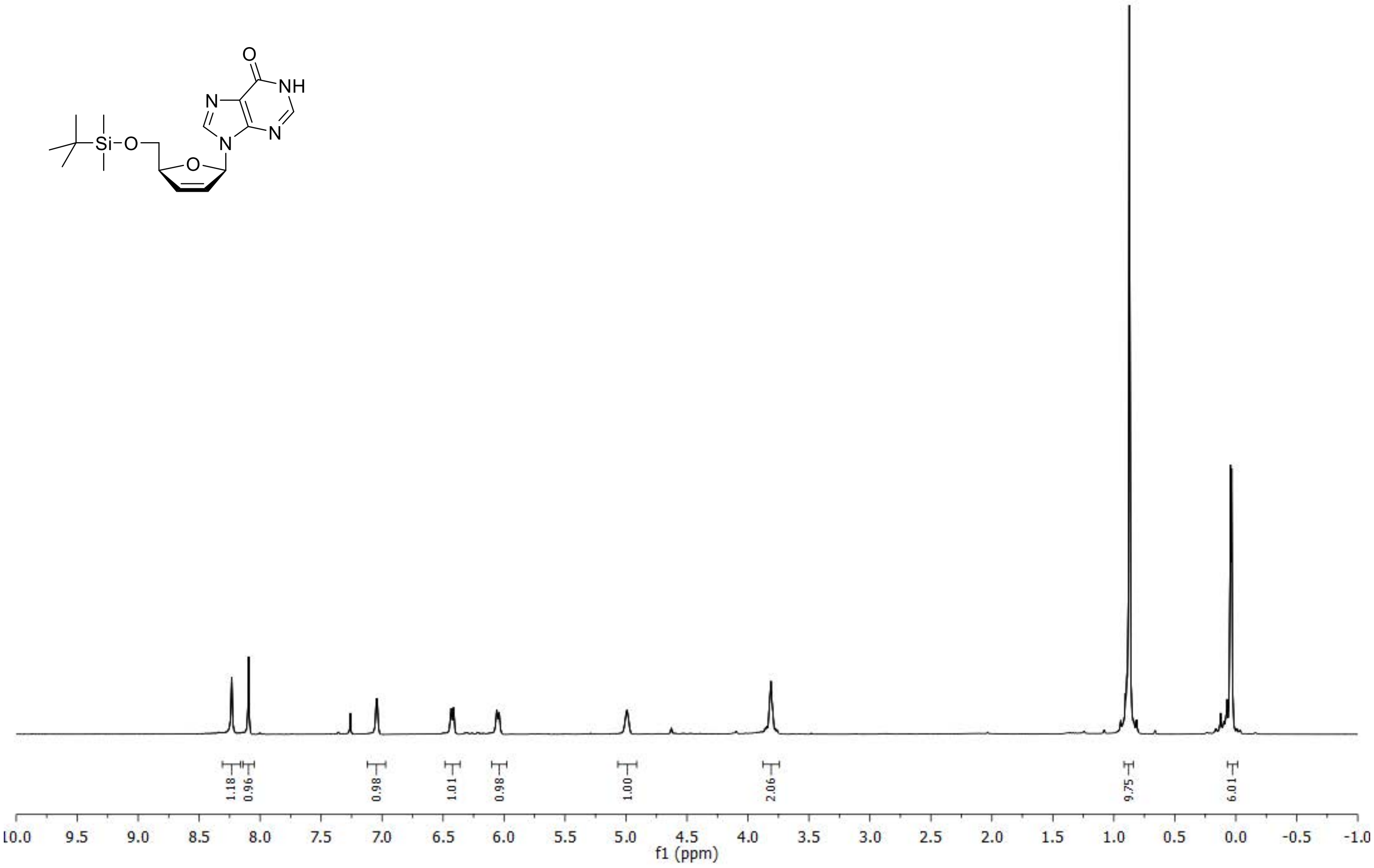

5’-*O*-(*tert*-Butyldimethylsilyl)-2’,3’-didehydro-2’,3’-dideoxy-β-D-inosine (7f)

^{13}C-NMR (75.5 MHz, $CDCl_3$)

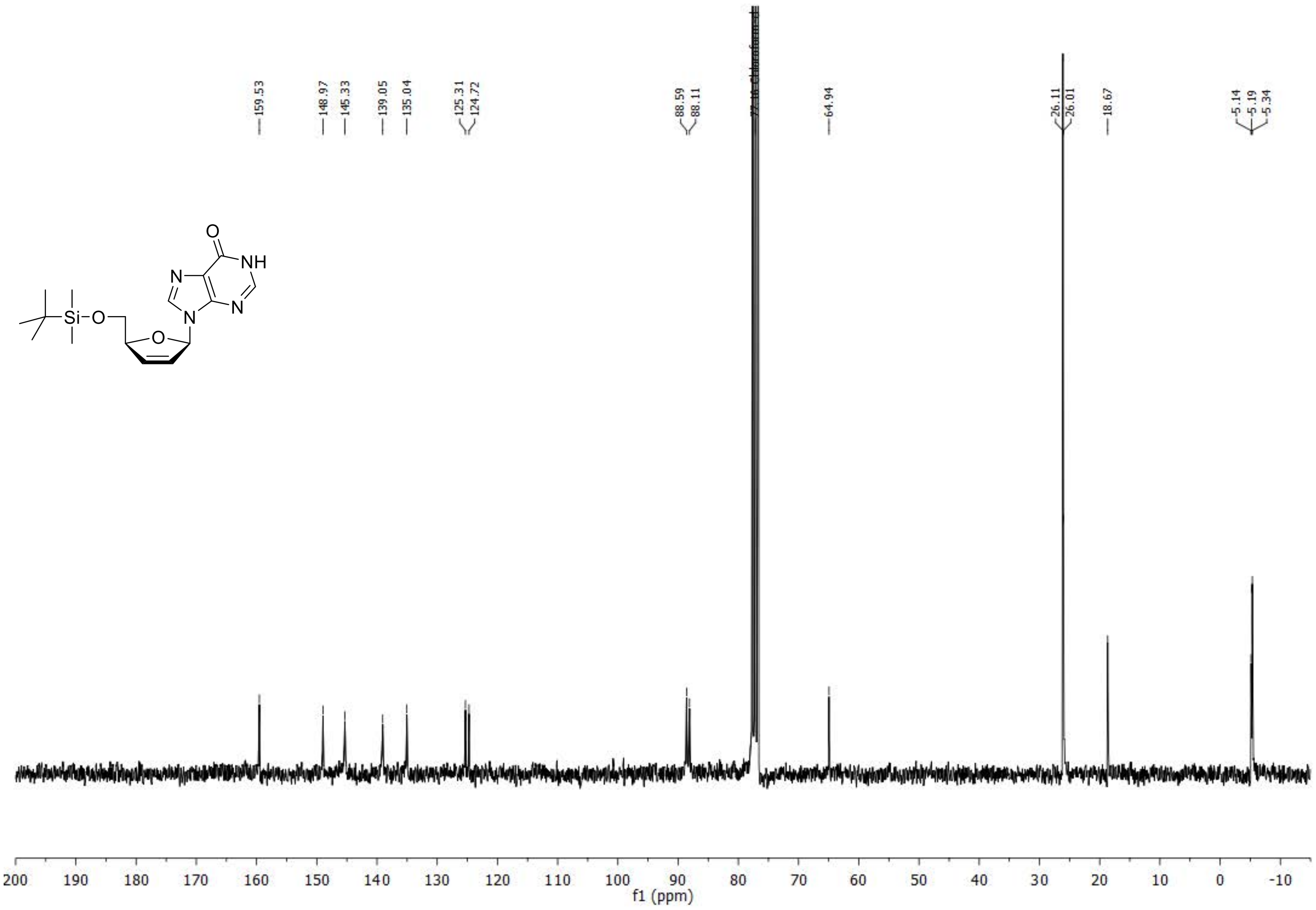

5’-*O*-(*tert*-Butyldimethylsilyl)-2’,3’-didehydro-2’,3’-dideoxy-β-D-inosine (7f)

COSY NMR ($CDCl_3$)

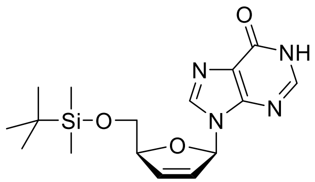

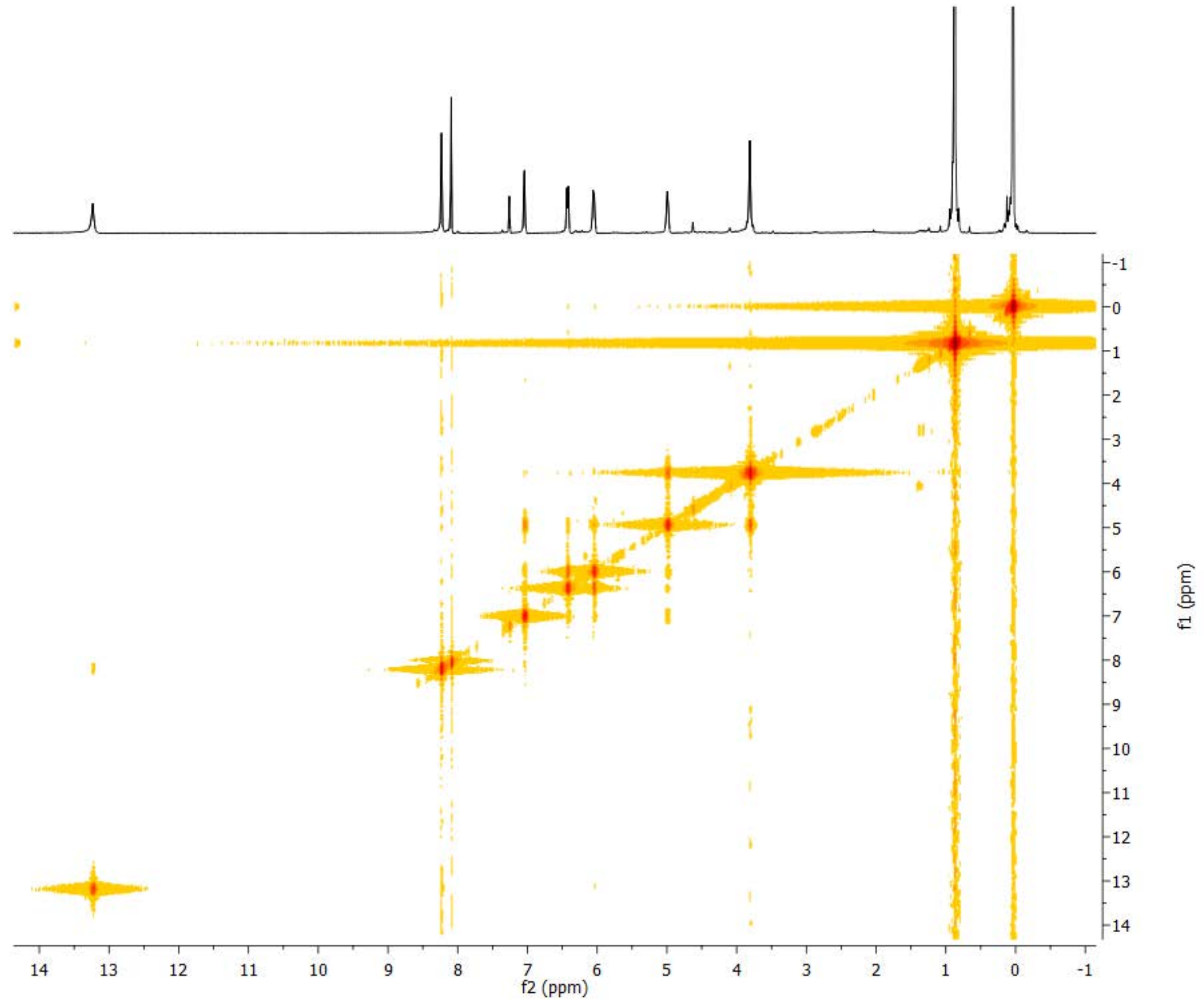

5’-*O*-(*tert*-Butyldimethylsilyl)-2’,3’-didehydro-2’,3’-dideoxy-β-D-inosine (7f)

HSQC NMR (CDCl$_3$)

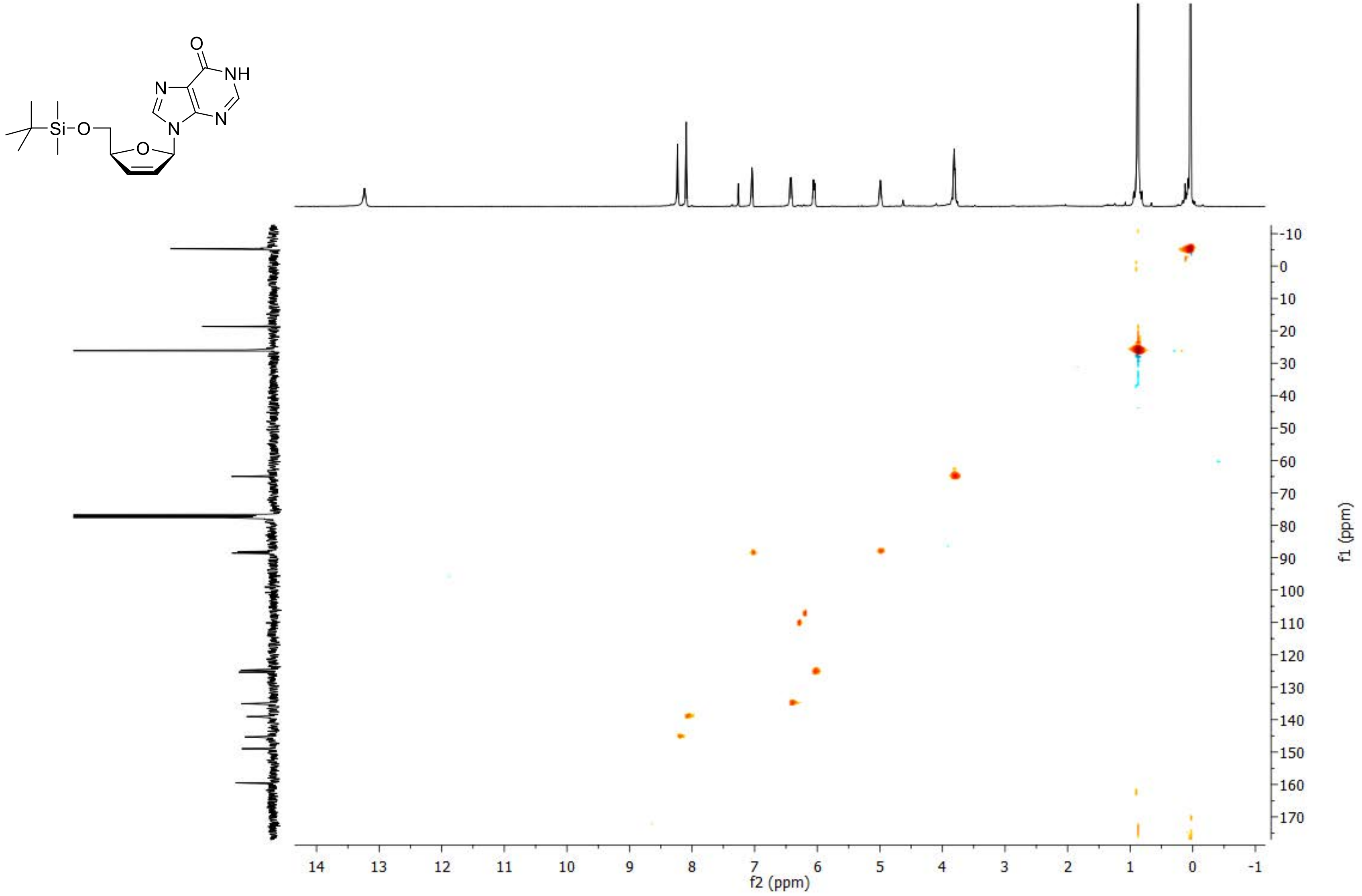

2’,3’-Didehydro-2’,3’-dideoxy-β-D-uridine (8a)

^{1}H-NMR (300.13 MHz, MeOH-d_4)

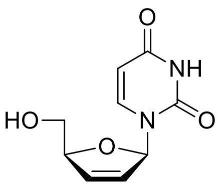

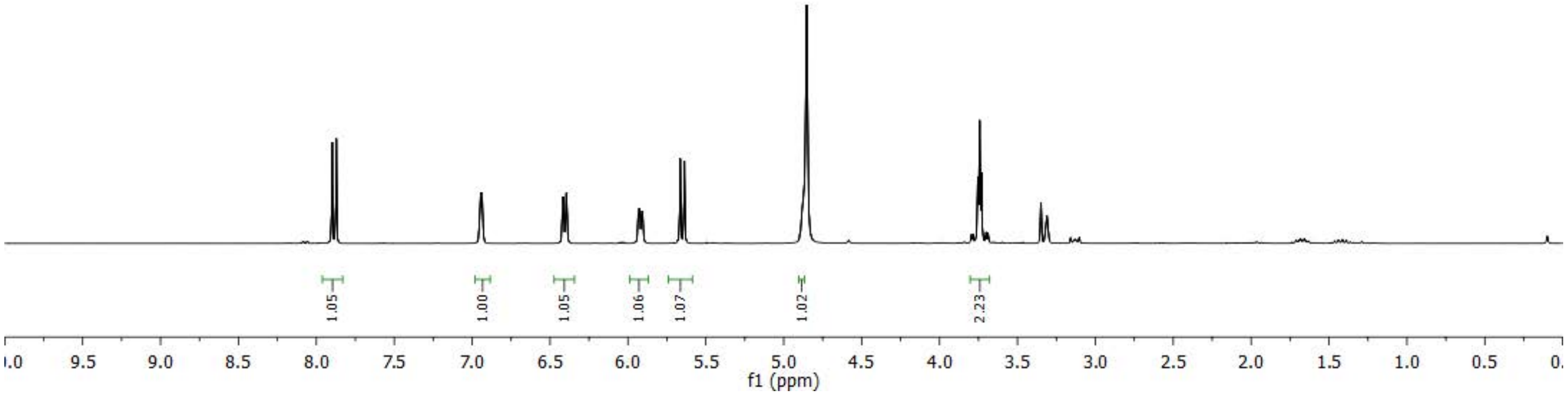

2’,3’-Didehydro-2’,3’-dideoxy-β-D-uridine (8a)

^{13}C-NMR (75.5 MHz, MeOH-d_4)

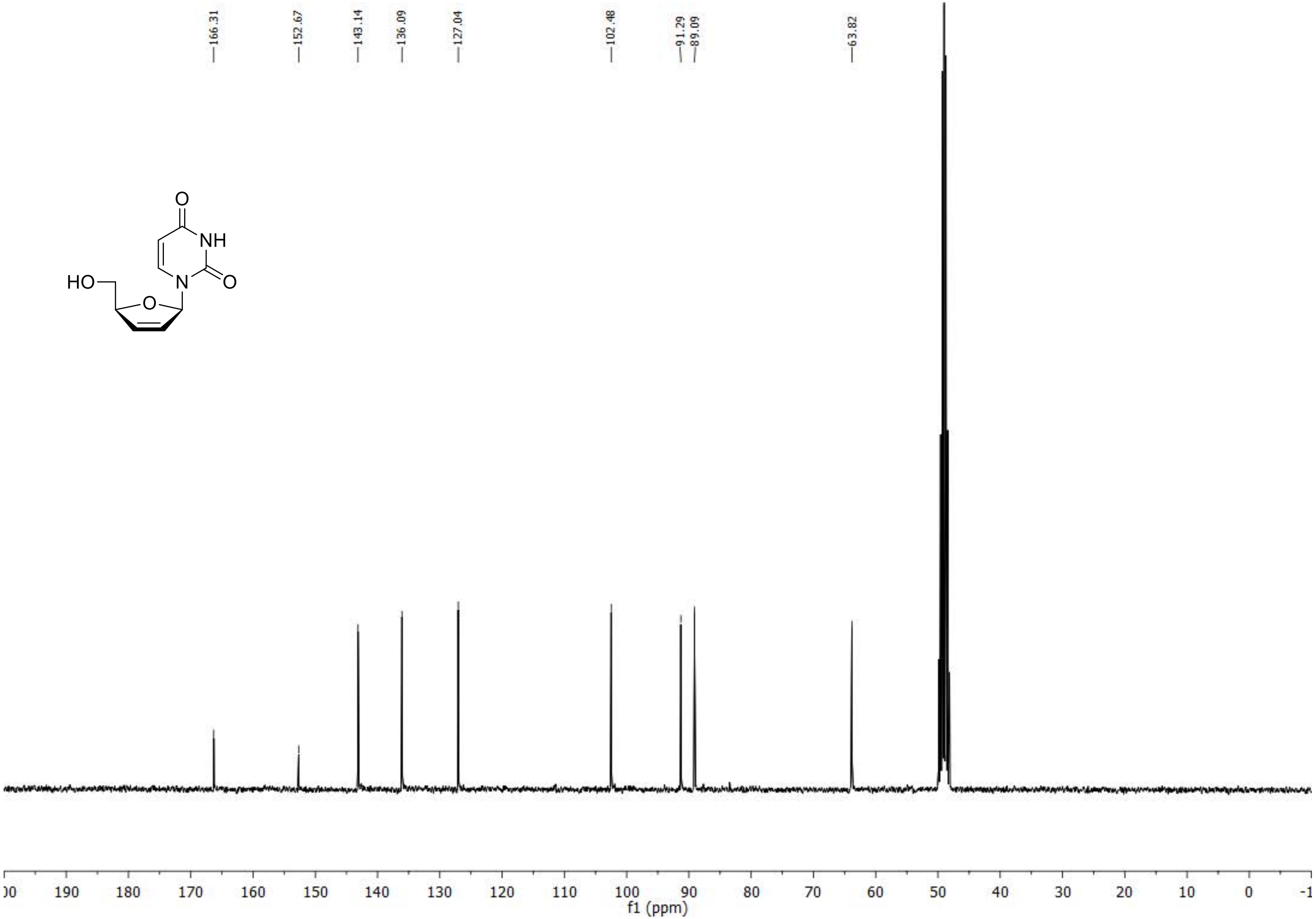

DEPT NMR (75.5 MHz, MeOH-d_4)

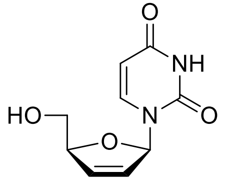

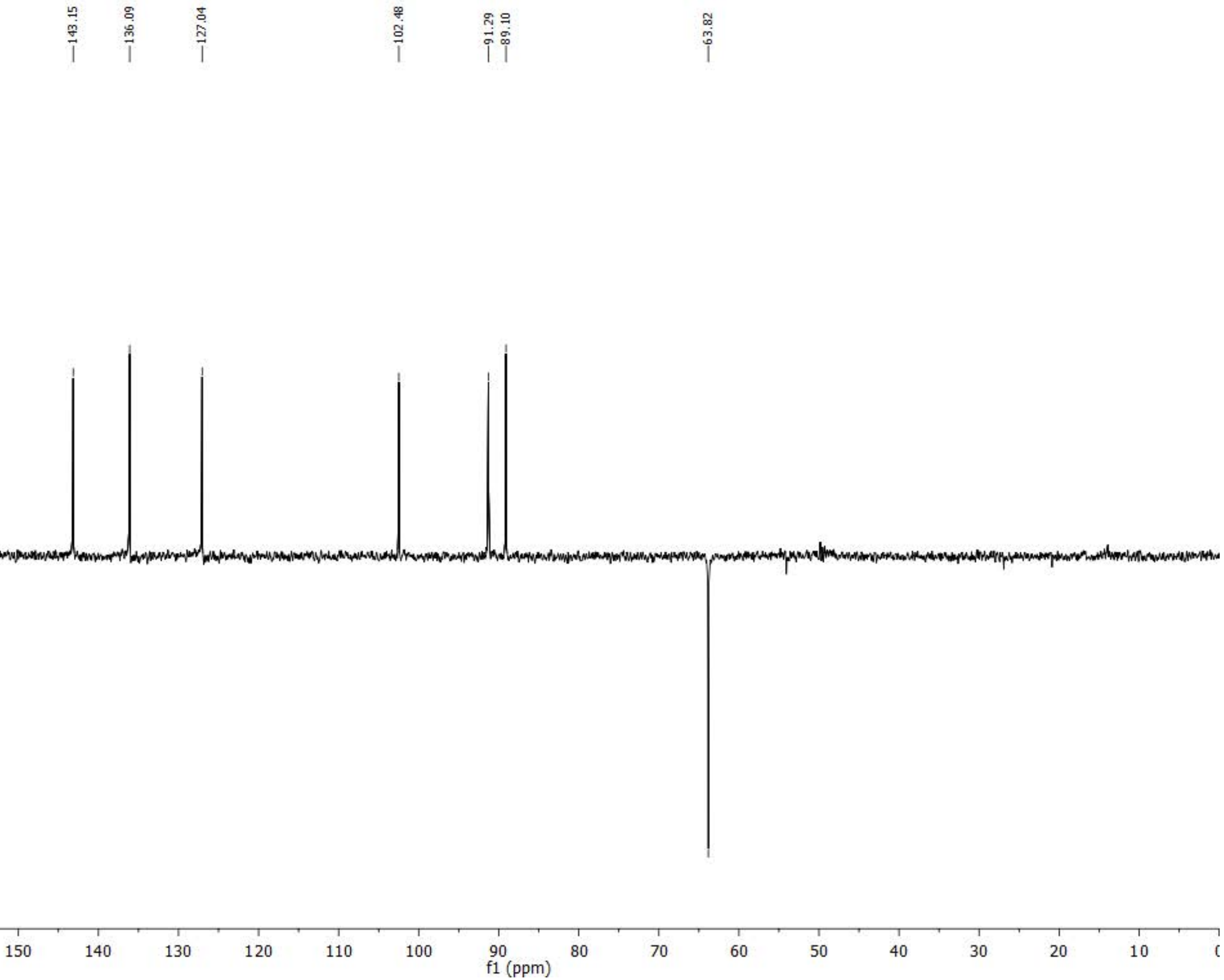

2’,3’-Didehydro-2’,3’-dideoxy-β-D-uridine (8a)

COSY NMR (MeOH-d_4)

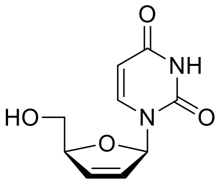

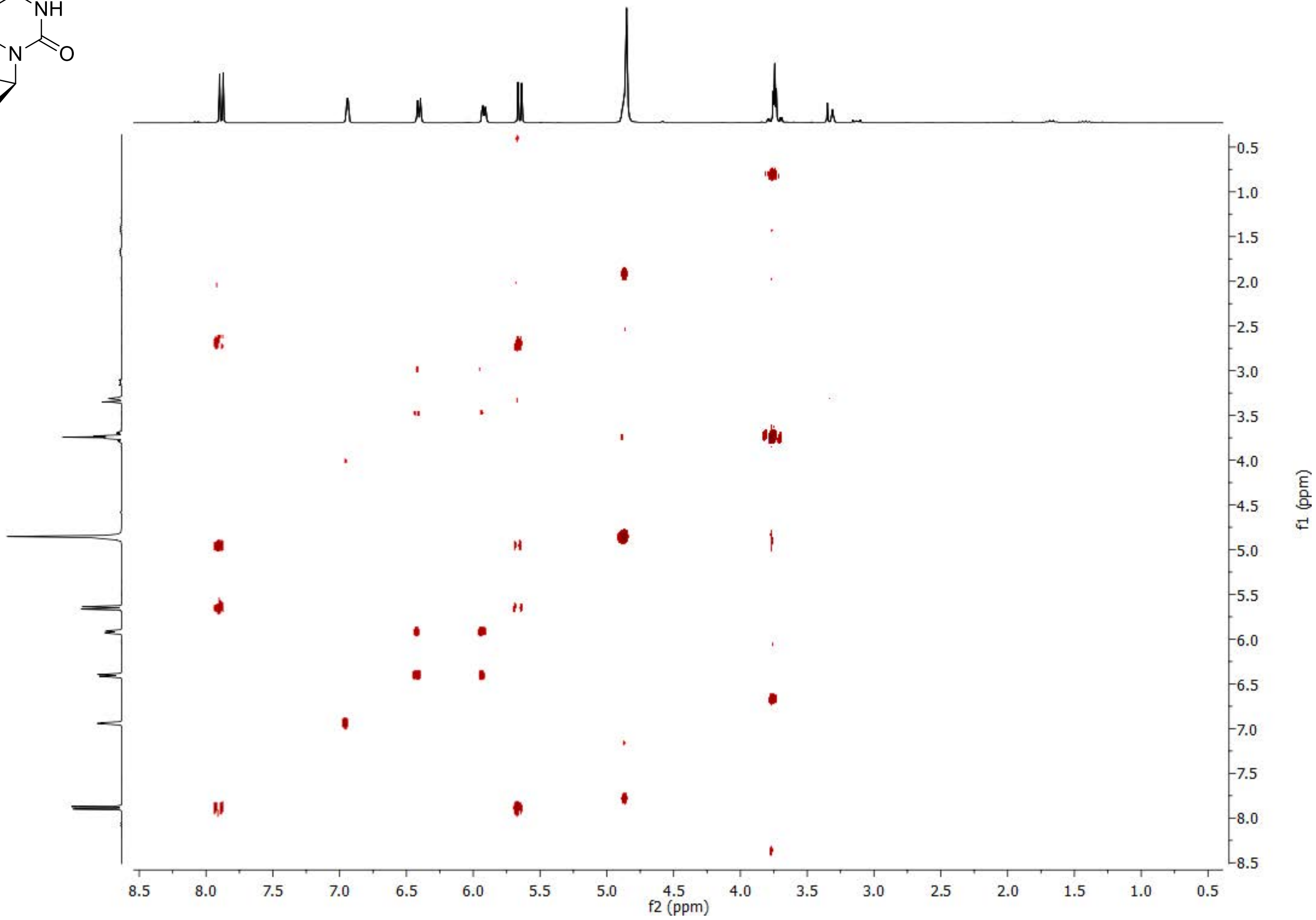

2',3'-Didehydro-2',3'-dideoxy-β-D-uridine (8a)

HSQC NMR (MeOH-d_4)

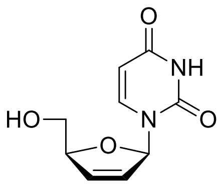

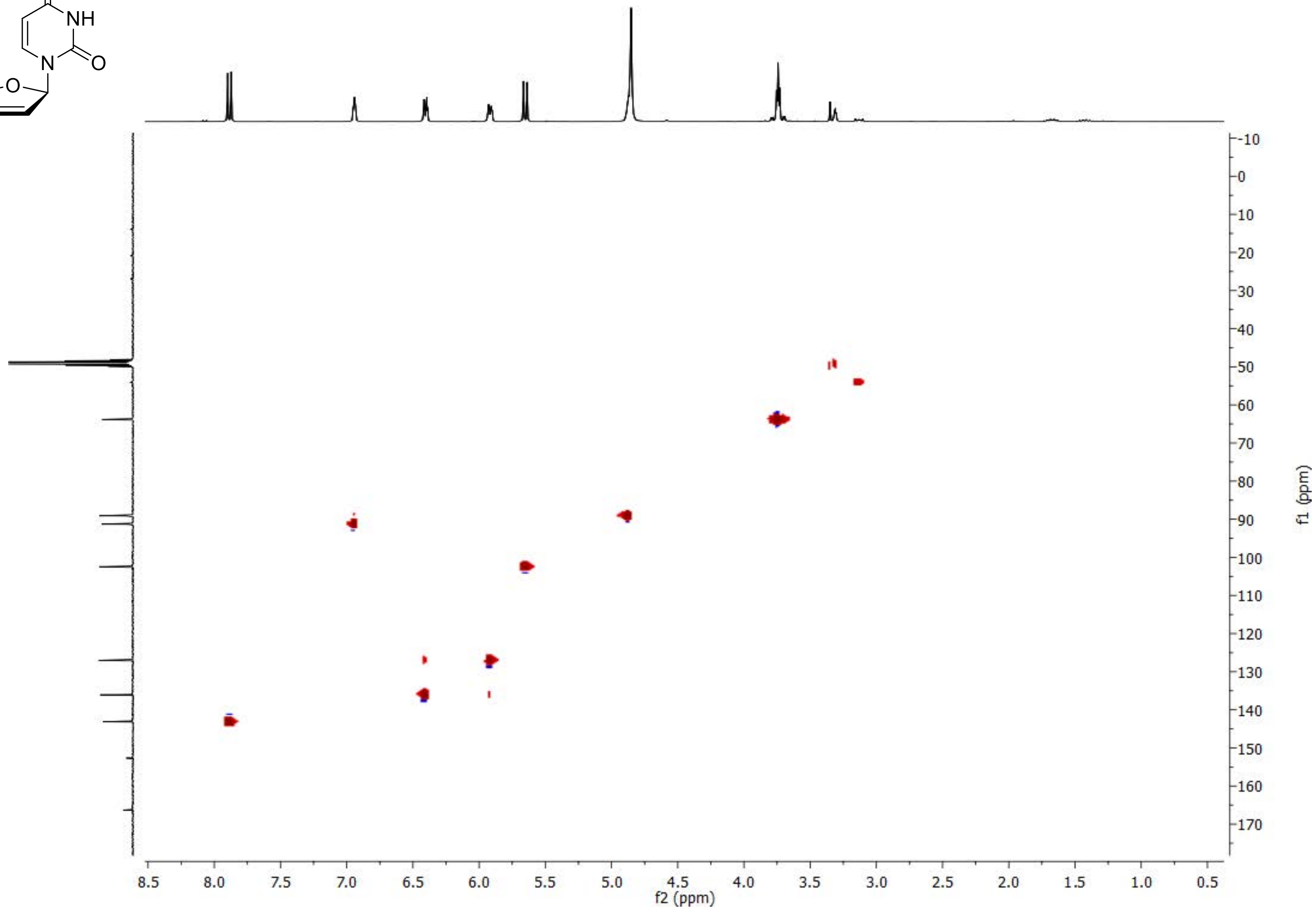

2’,3’-Didehydro-3’-deoxy-β-D-5-thymidine (8b)

^{1}H-NMR (300.13 MHz, MeOH-d_4)

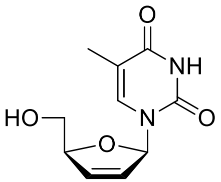

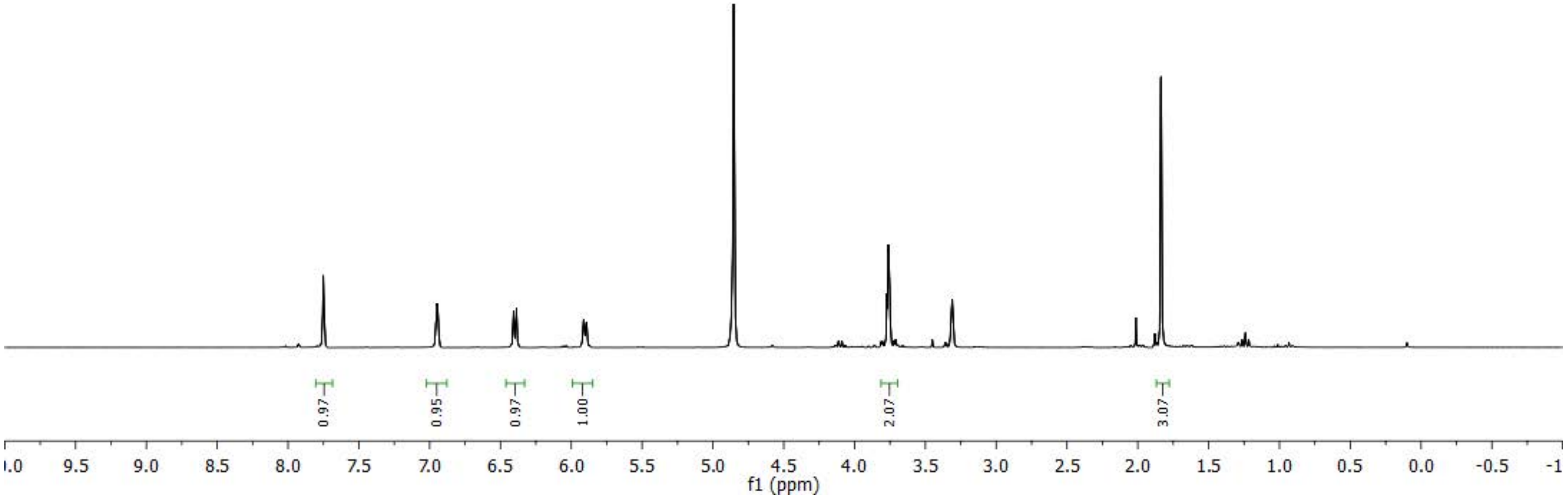

2’,3’-Didehydro-3’-deoxy-β-D-5-thymidine (8b)

^{13}C-NMR (75.5 MHz, MeOH-d_4)

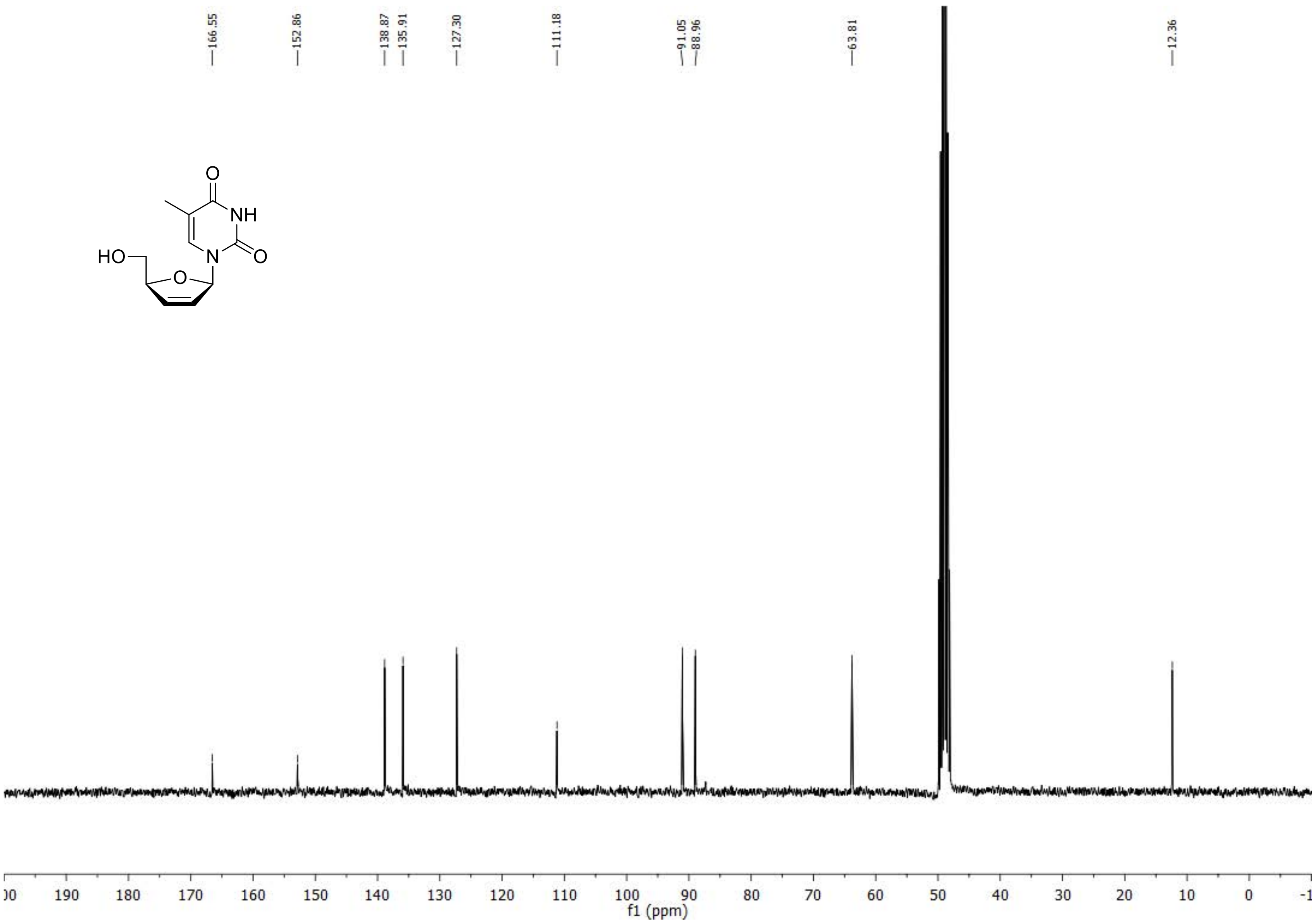

N[4]-Benzoyl-2’,3’-didehydro-2’,3’-dideoxy-β-D-cytidine (8d)

^{1}H-NMR (300.13 MHz, MeOH-d_4)

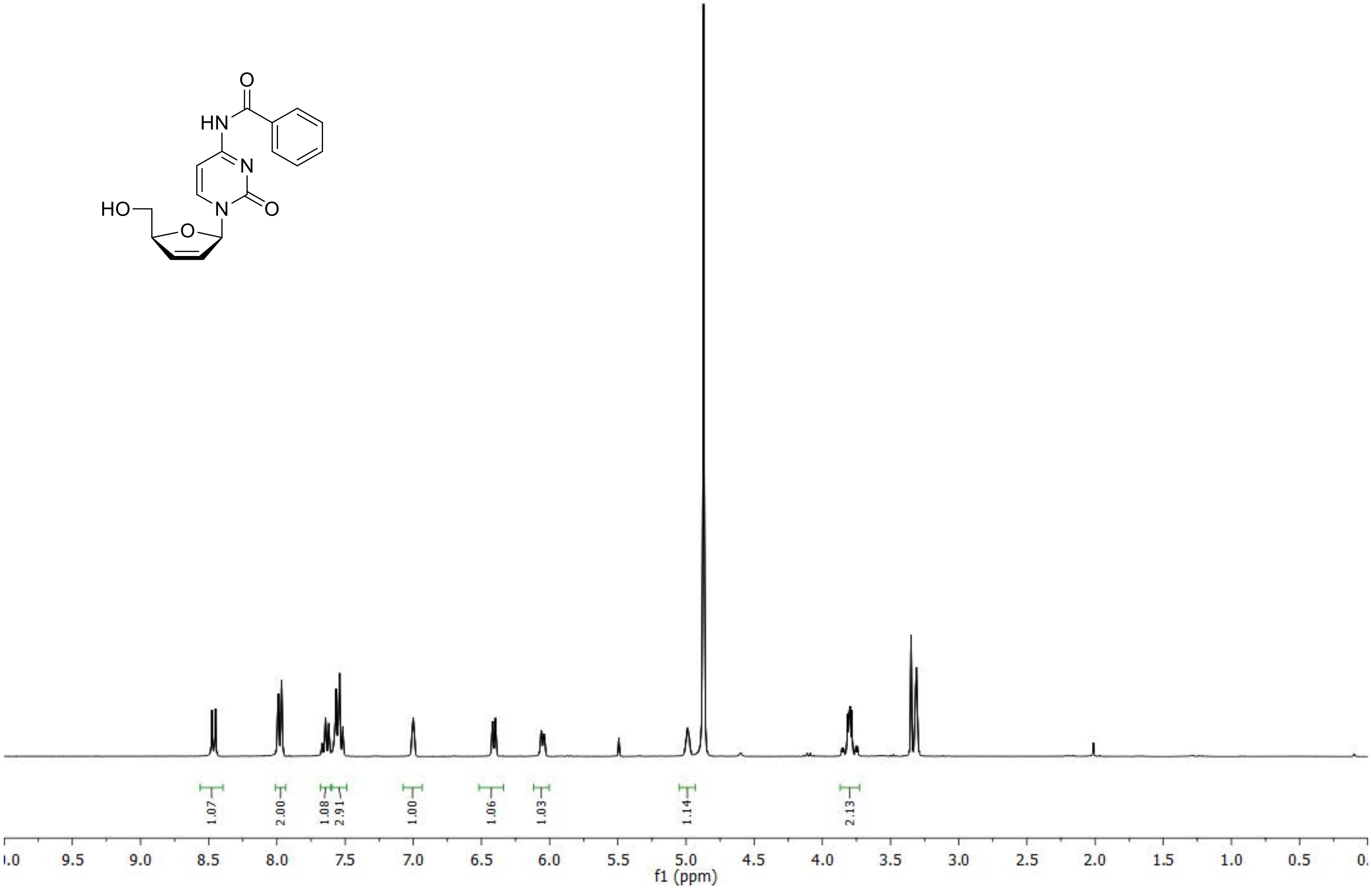

N^4-Benzoyl-2’,3’-didehydro-2’,3’-dideoxy-β-D-cytidine (8d)

^{13}C-NMR (75.5 MHz, MeOH-d_4)

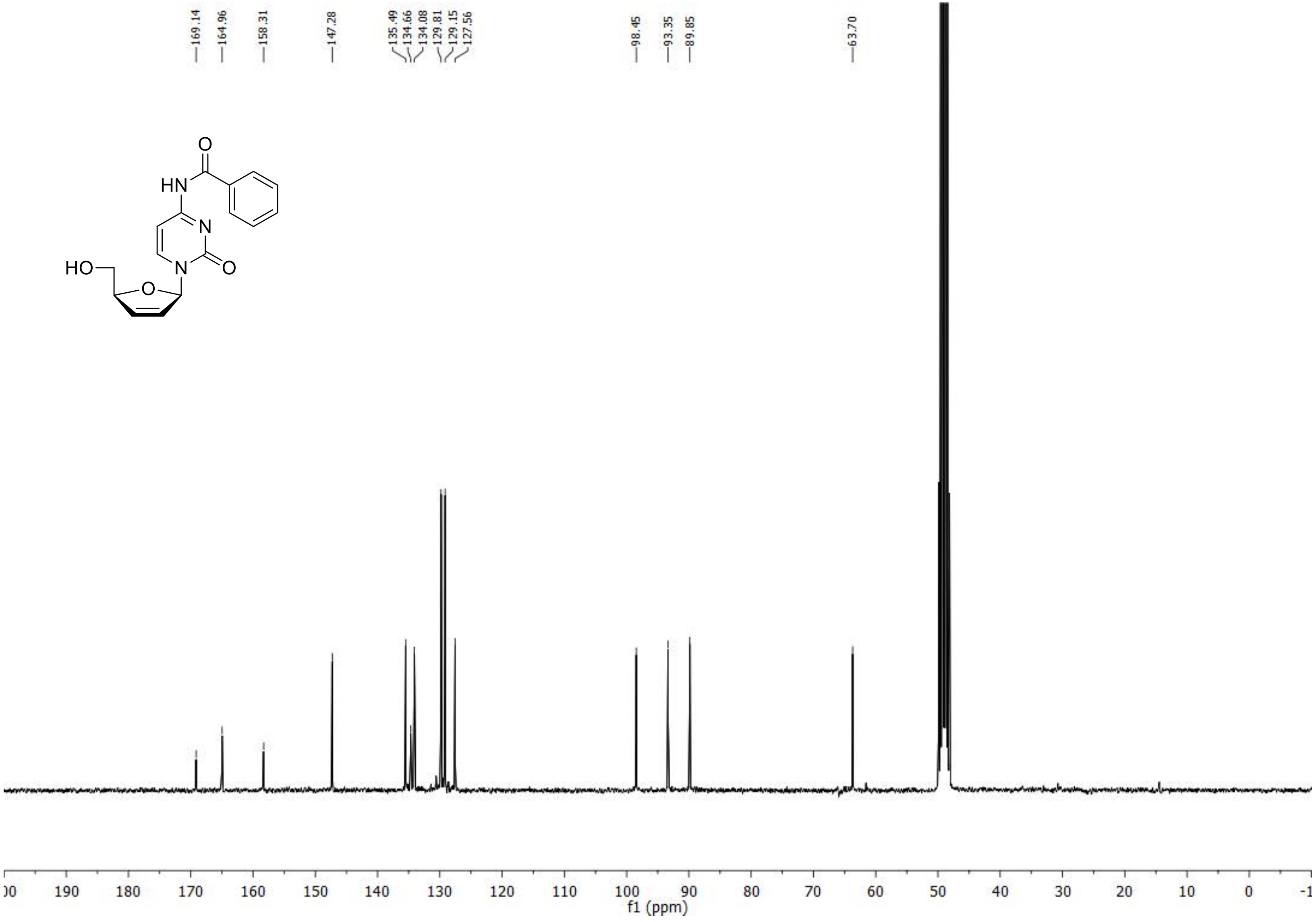

N^4-Benzoyl-2’,3’-didehydro-2’,3’-dideoxy-β-D-cytidine (8d)

DEPT NMR (75.5 MHz, MeOH-d_4)

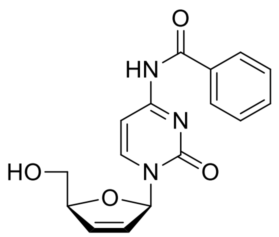

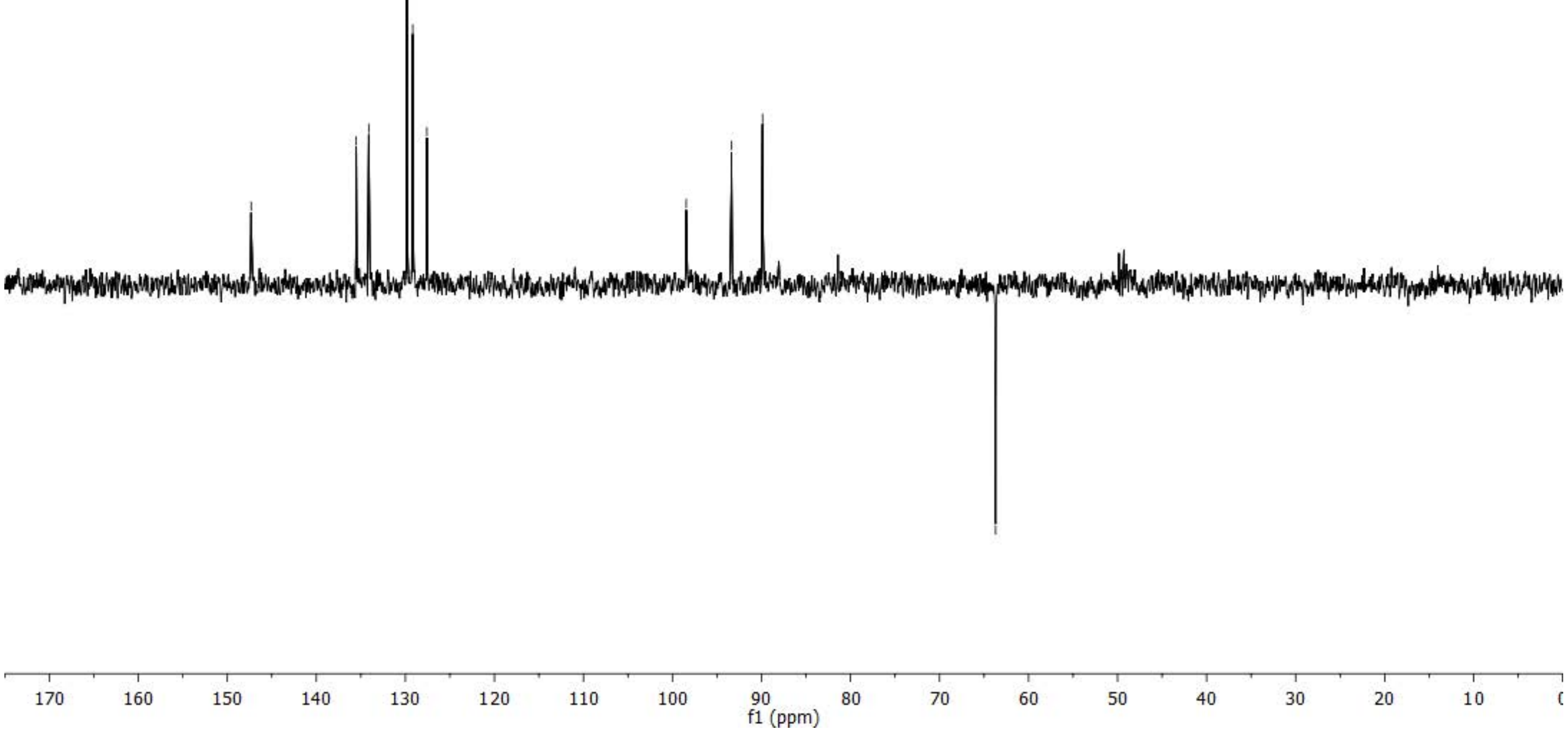

N^4-Benzoyl-2',3'-didehydro-2',3'-dideoxy-β-D-cytidine (8d)

COSY NMR (MeOH-d_4)

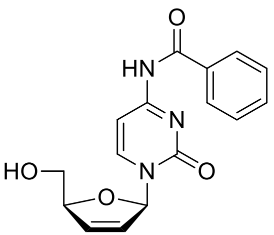

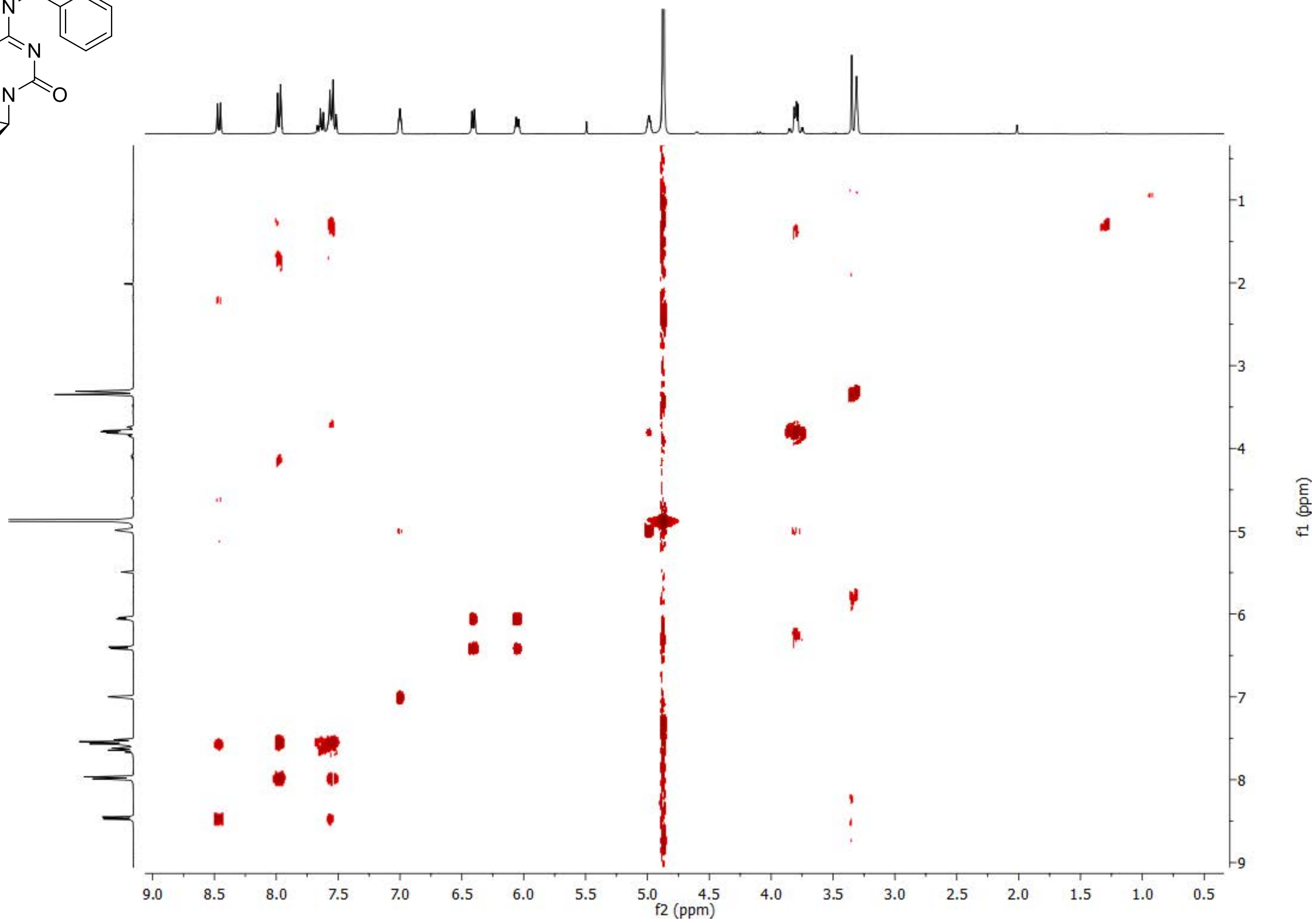

N^4-Benzoyl-2',3'-didehydro-2',3'-dideoxy-β-D-cytidine (8d)

HSQC NMR (MeOH-d_4)

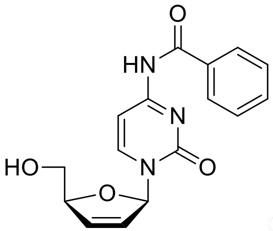

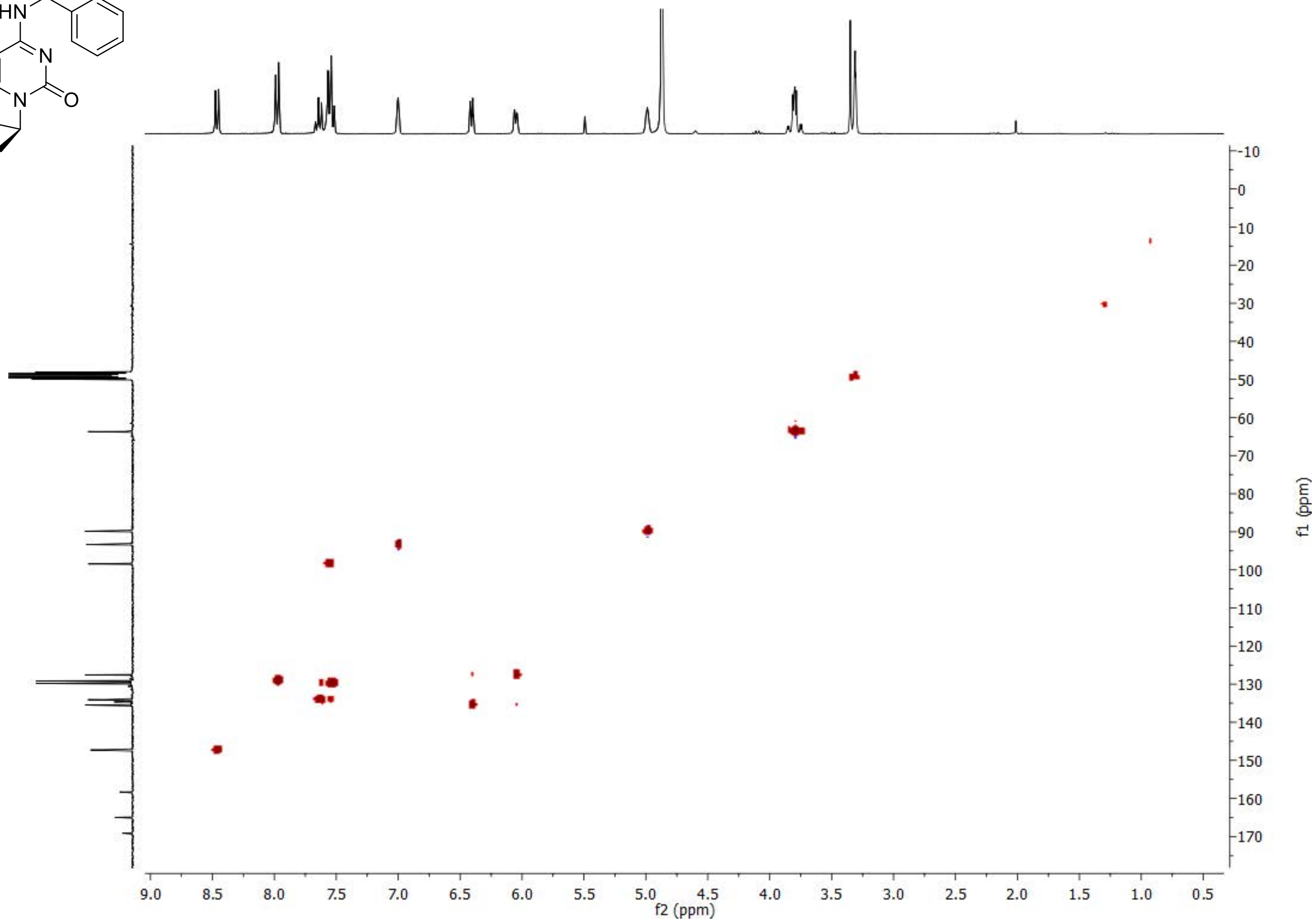

N^4-Benzoyl-2',3'-didehydro-2',3'-dideoxy-β-D-cytidine (8d)

HMBC NMR (MeOH-d_4)

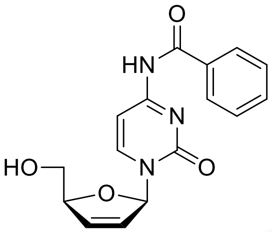

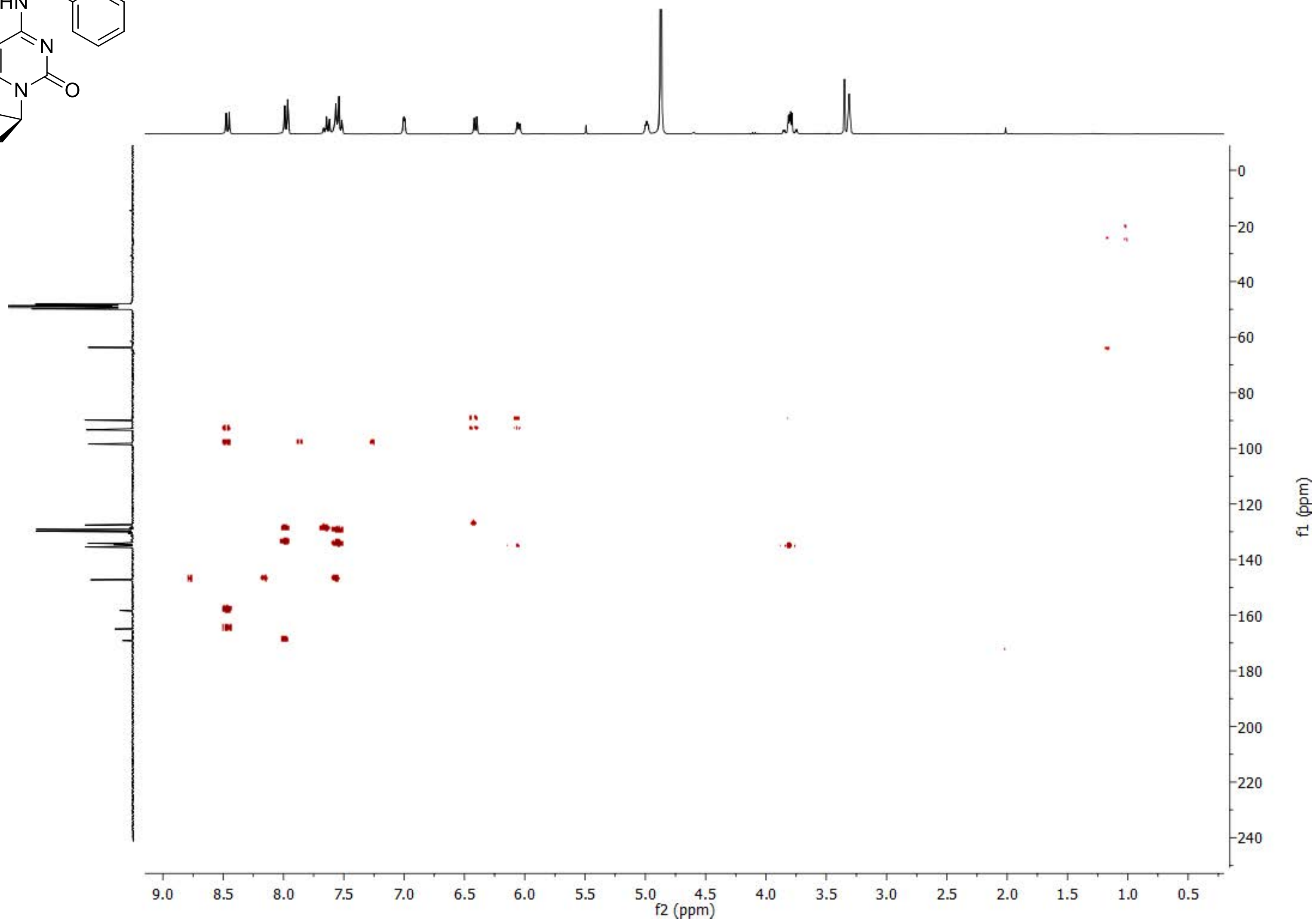

2’,3’-Didehydro-2’,3’-dideoxy-β-D-adenosine (8e)

^{1}H-NMR (300.13 MHz, MeOH-d_4)

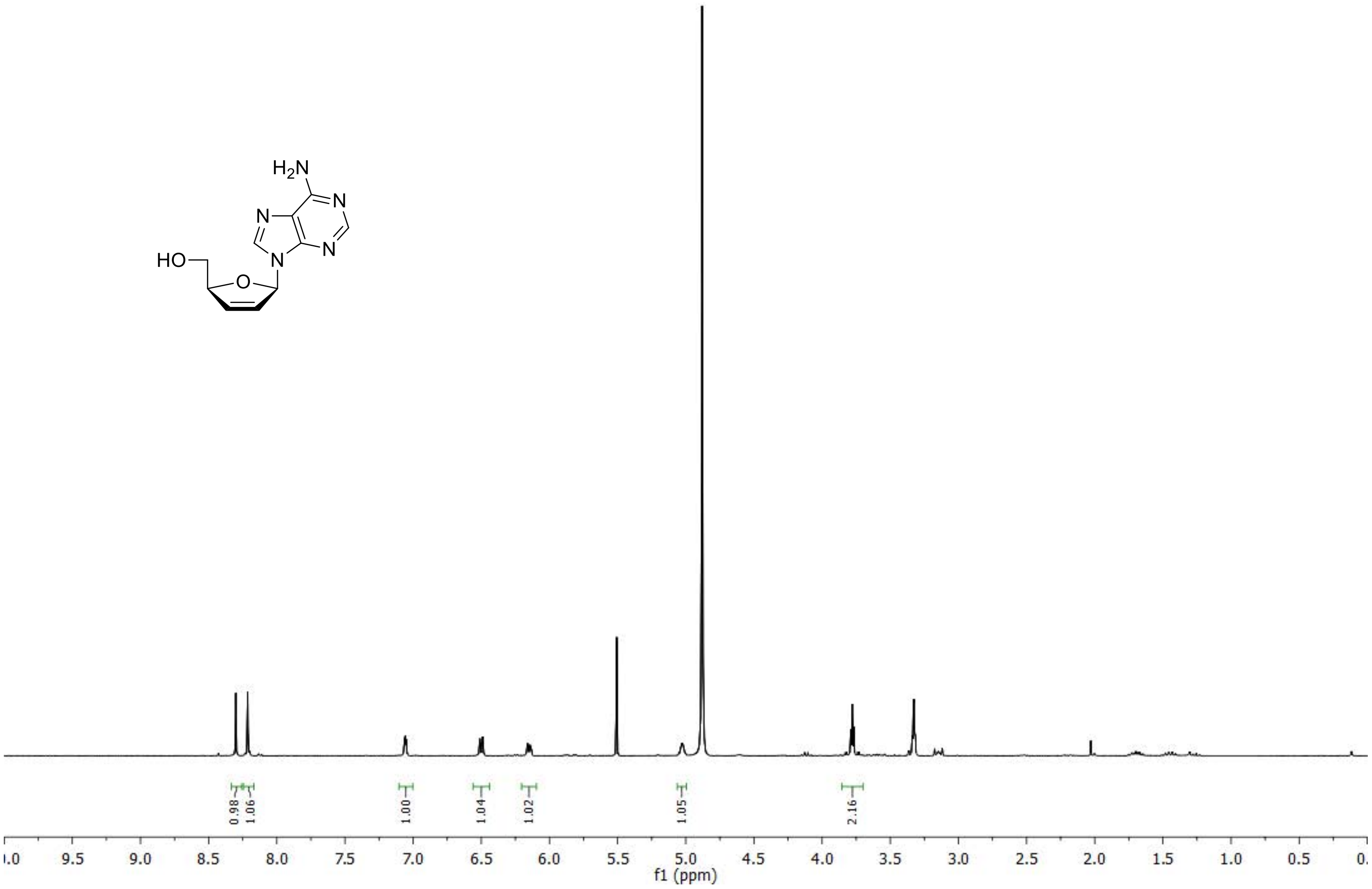

2’,3’-Didehydro-2’,3’-dideoxy-β-D-adenosine (8e)

^{13}C-NMR (75.5 MHz, MeOH-d_4)

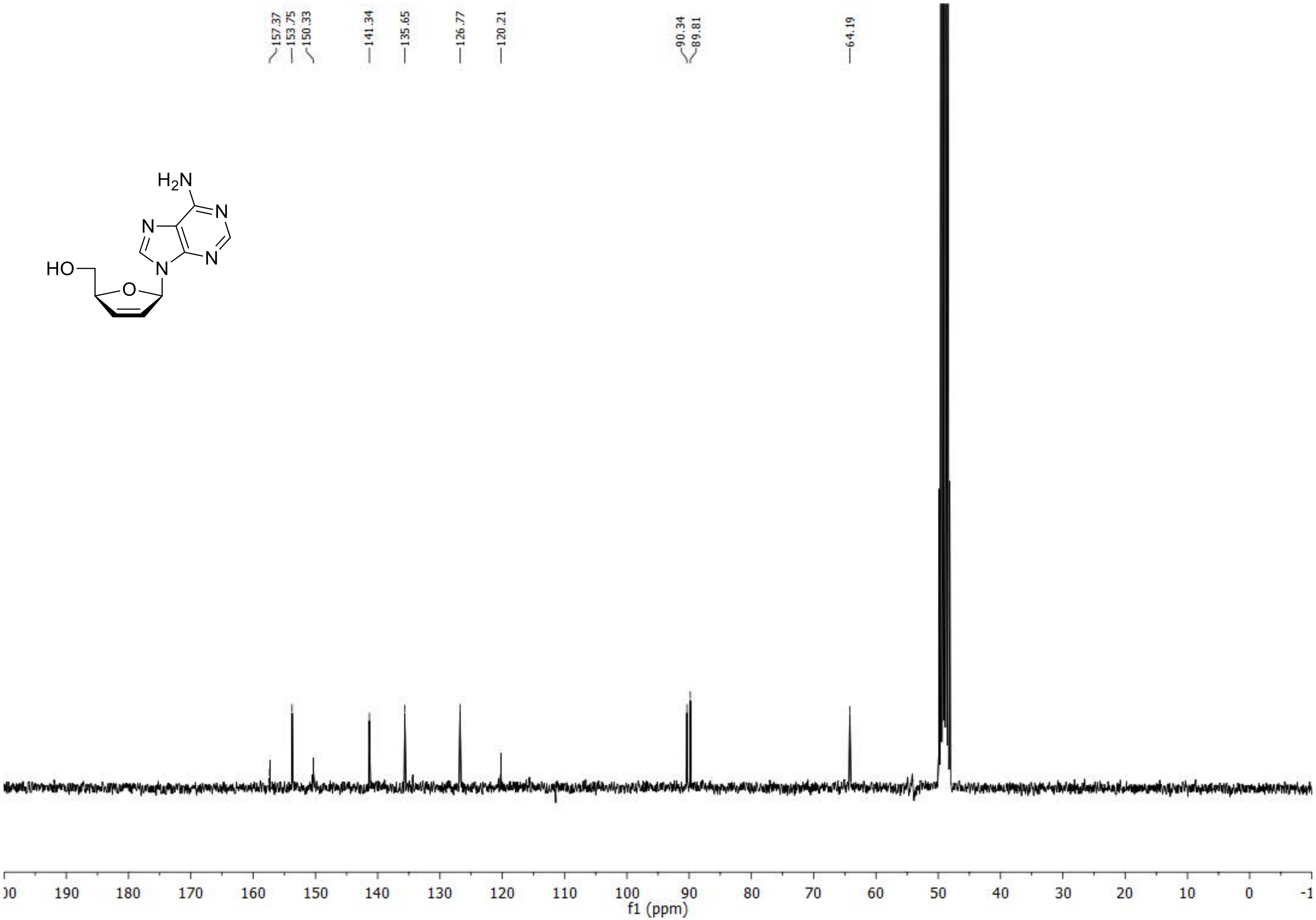

2’,3’-Didehydro-2’,3’-dideoxy-β-D-inosine (8f)

^{1}H-NMR (300.13 MHz, DMSO-d_6)

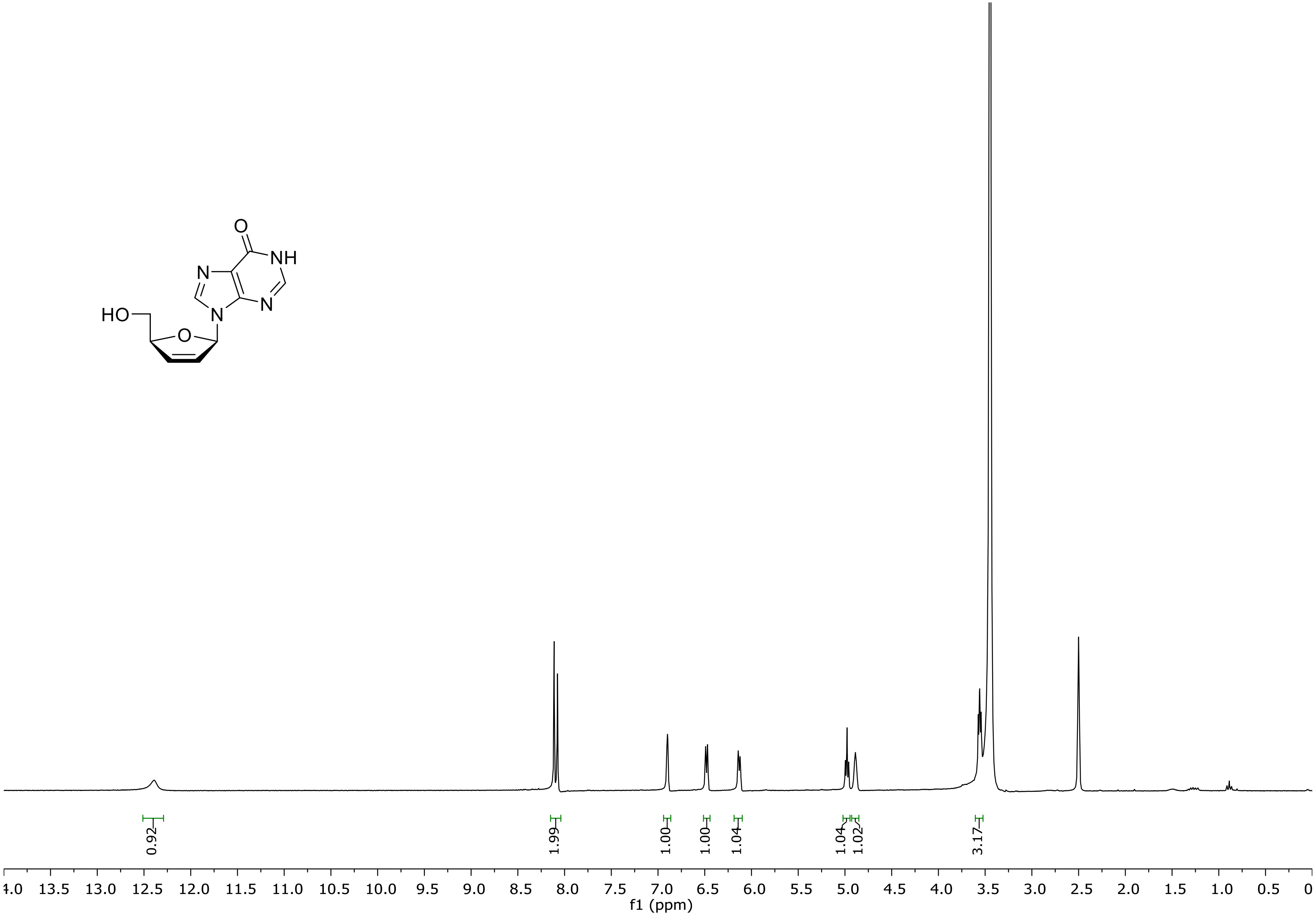

2’,3’-Didehydro-2’,3’-dideoxy-β-D-inosine (8f)

^{13}C-NMR (75.5 MHz, DMSO-d_6)

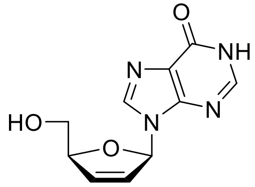

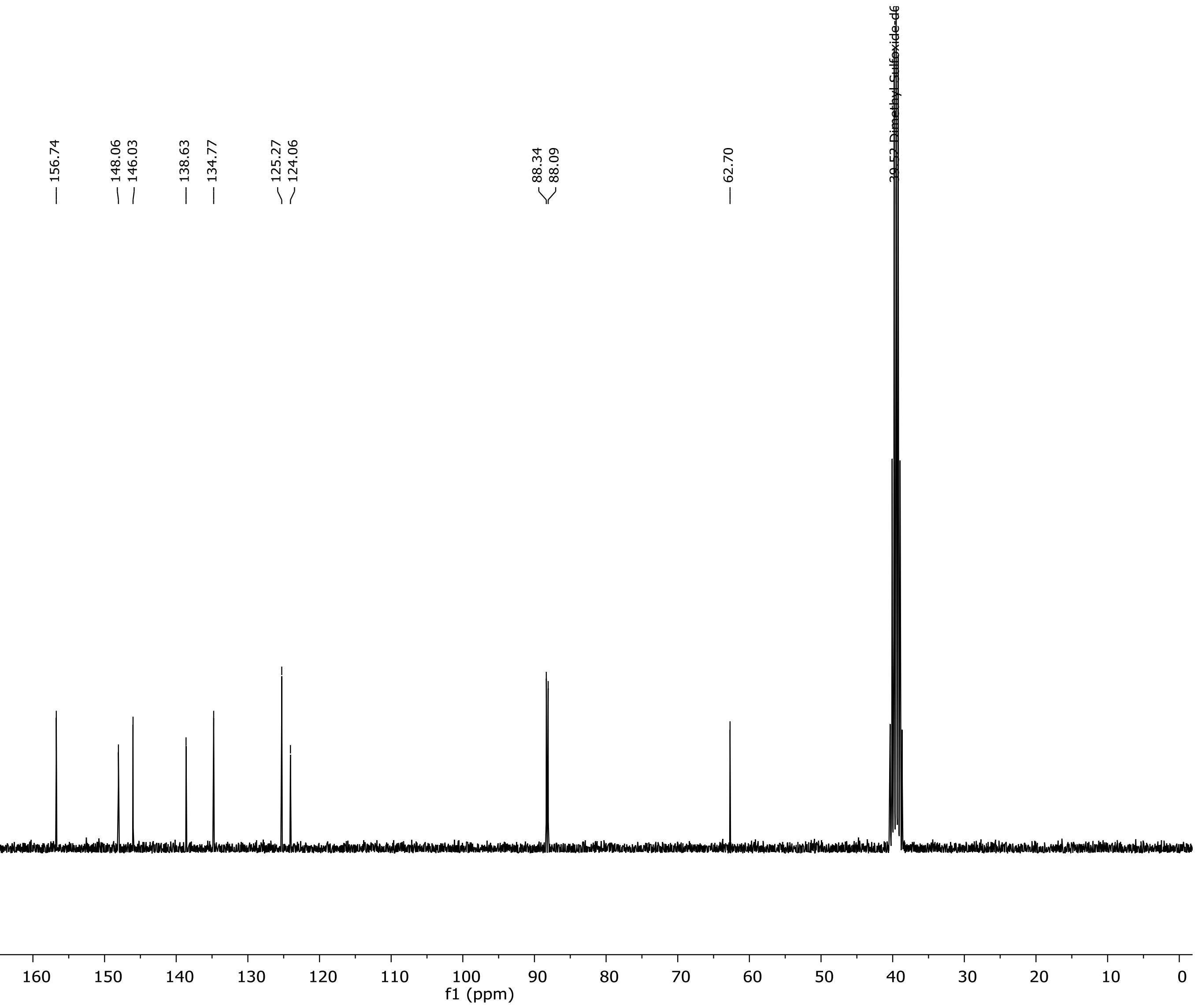

2’,3’-Didehydro-2’,3’-dideoxy-β-D-inosine (8f)

COSY NMR (DMSO-d_6)

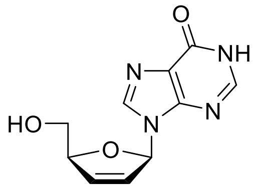

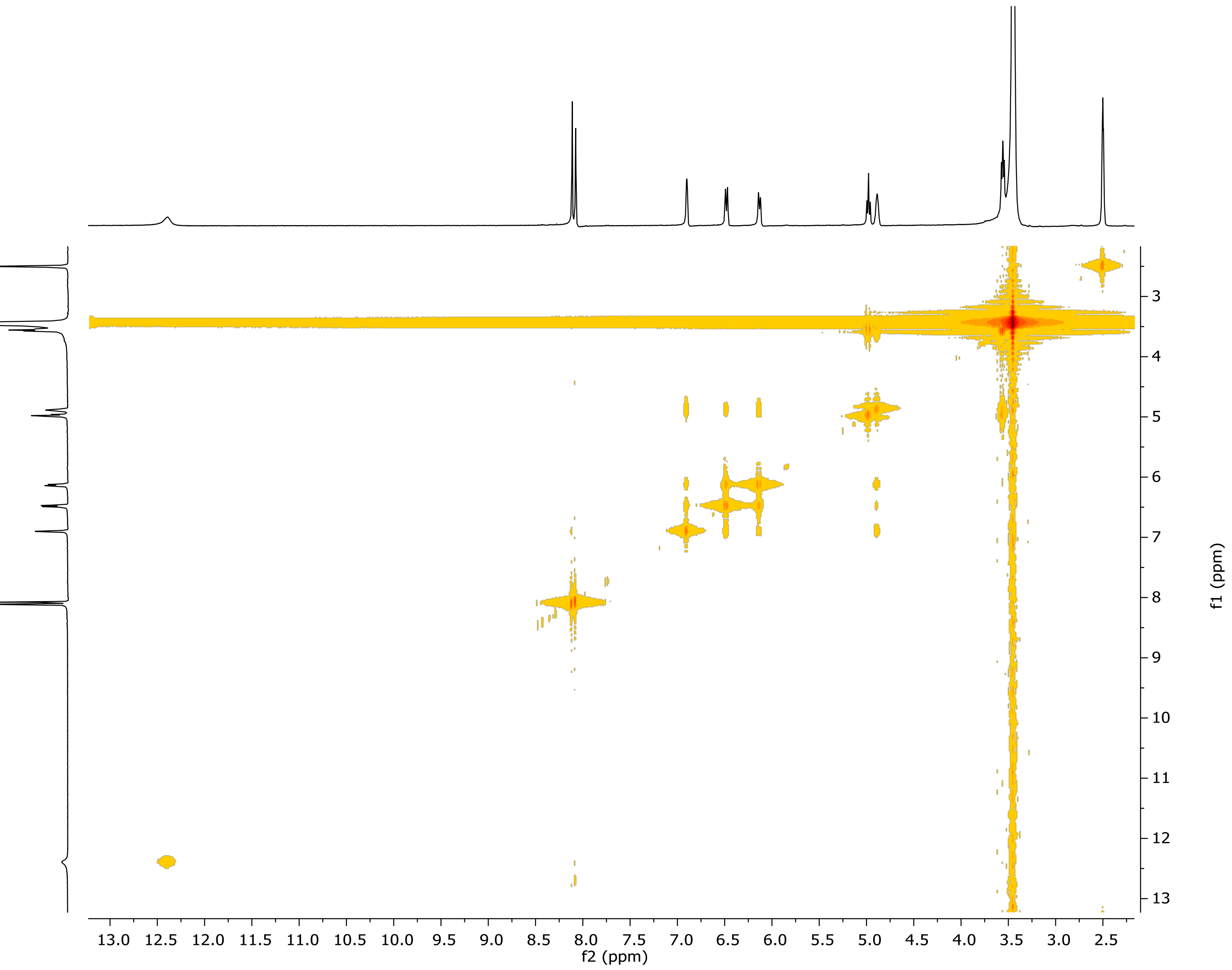

2’,3’-Didehydro-2’,3’-dideoxy-β-D-inosine (8f)

HSQC NMR (DMSO-d_6)

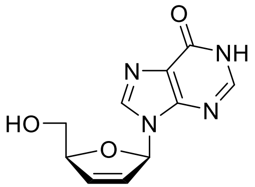

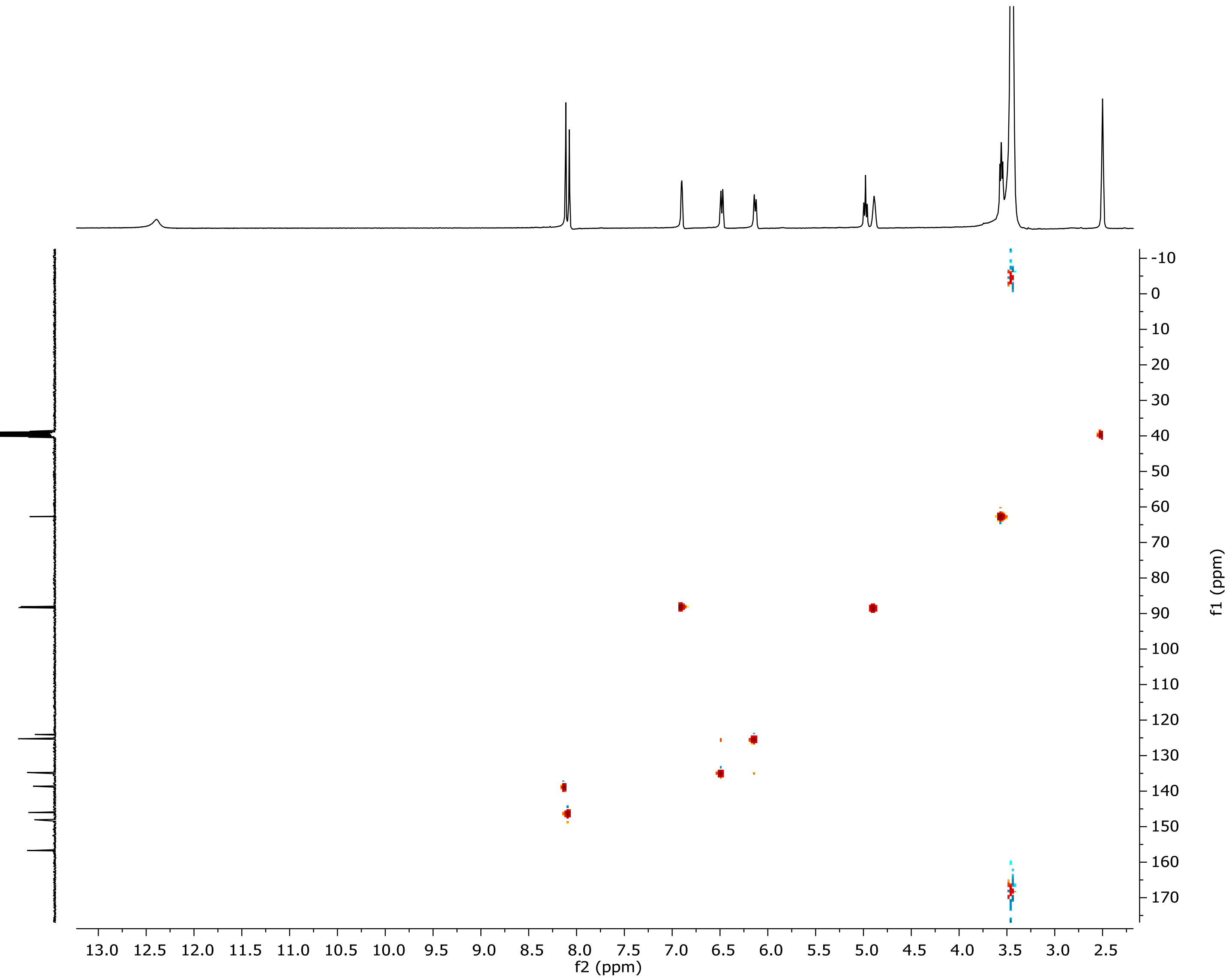

2’,3’-Didehydro-2’,3’-dideoxy-β-D-inosine (8f)

HMBC NMR (DMSO-d_6)

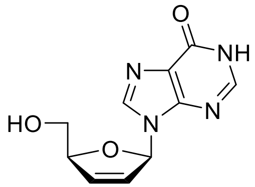

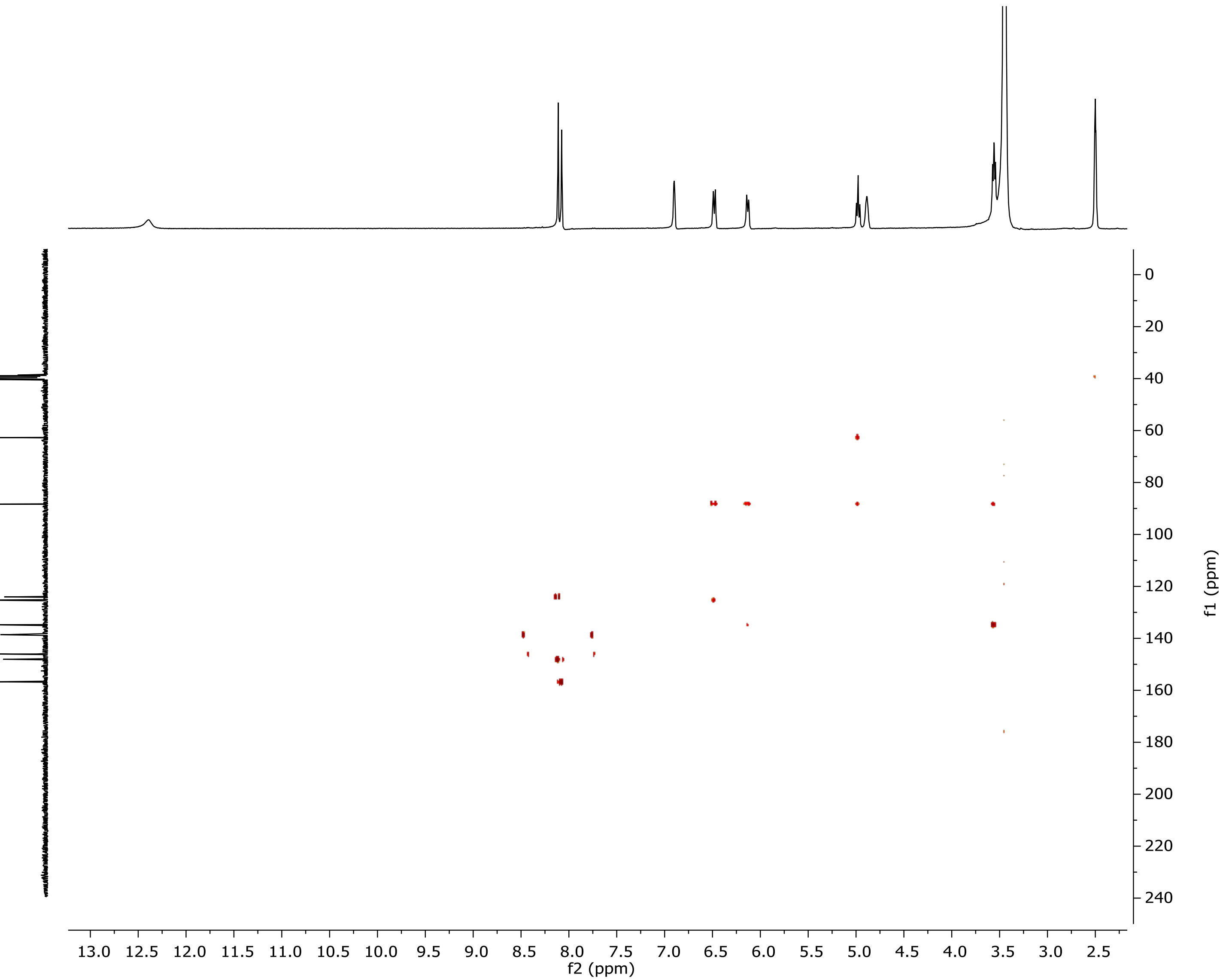

2’,3’-Dideoxy-β-D-uridine (9a)

^{1}H-NMR (300.13 MHz, MeOH-d_4)

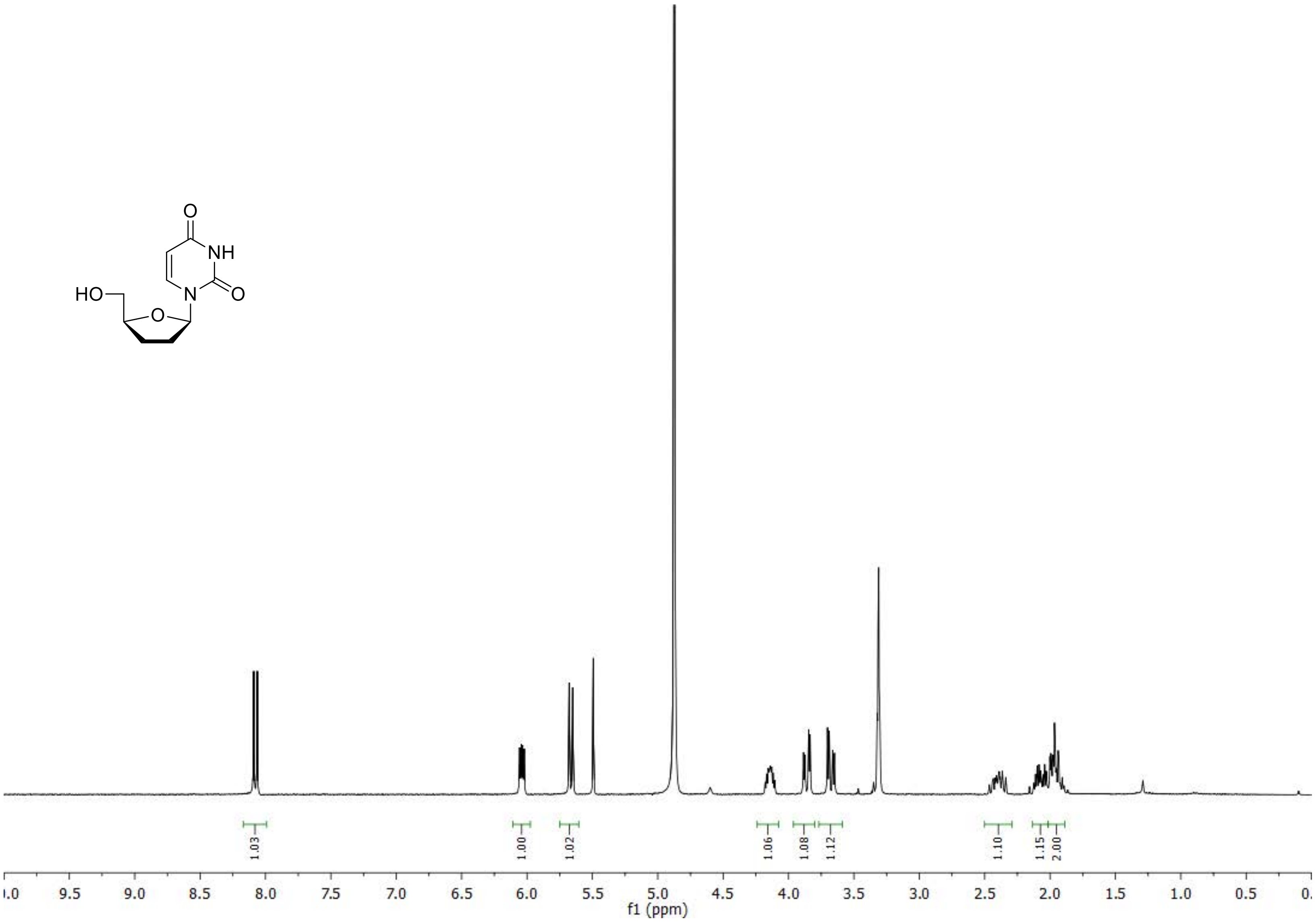

2’,3’-Dideoxy-β-D-uridine (9a)

^{13}C-NMR (75.5 MHz, MeOH-d_4)

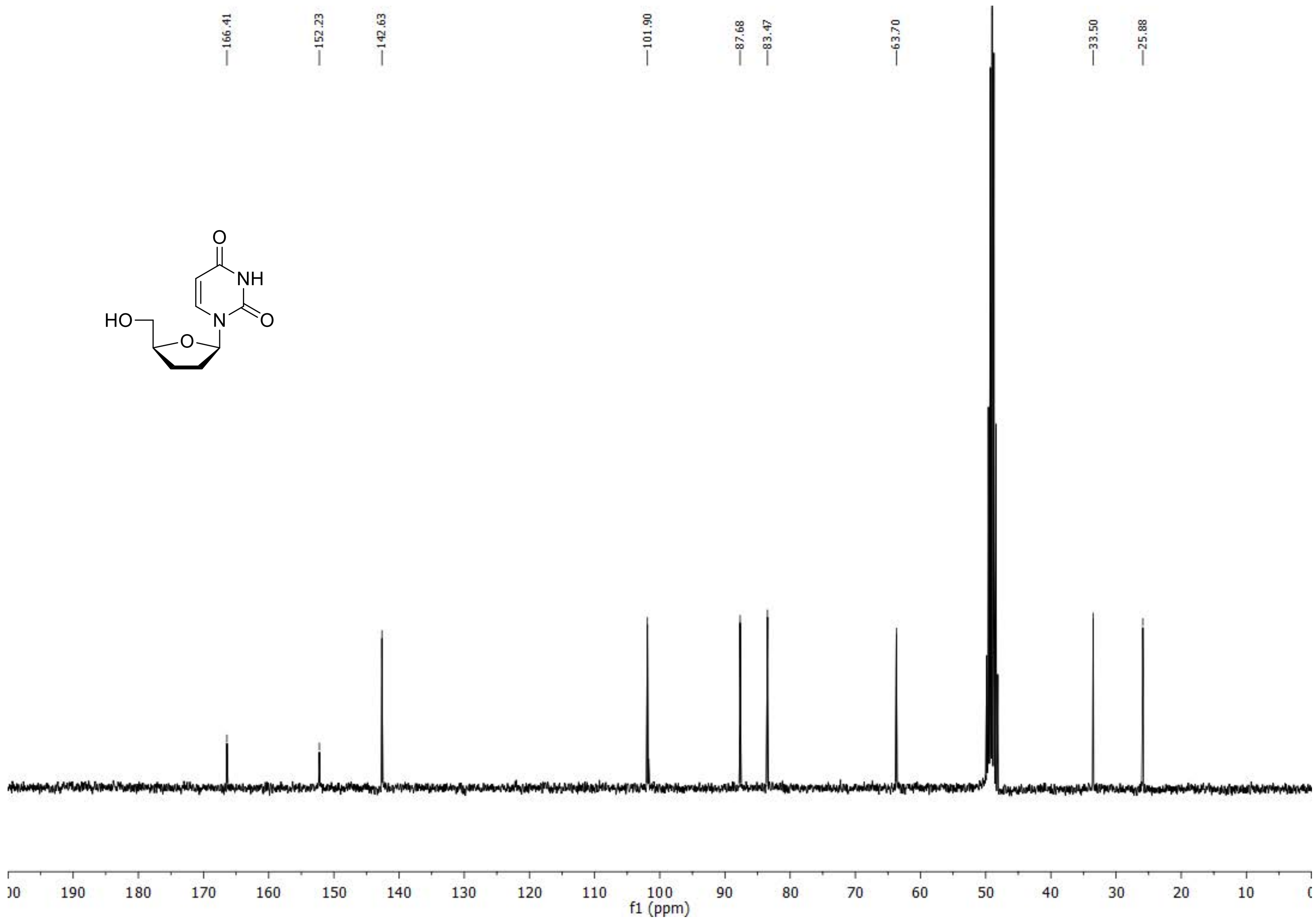

2’,3’-Dideoxy-β-D-uridine (9a)

DEPT NMR (75.5 MHz, MeOH-d_4)

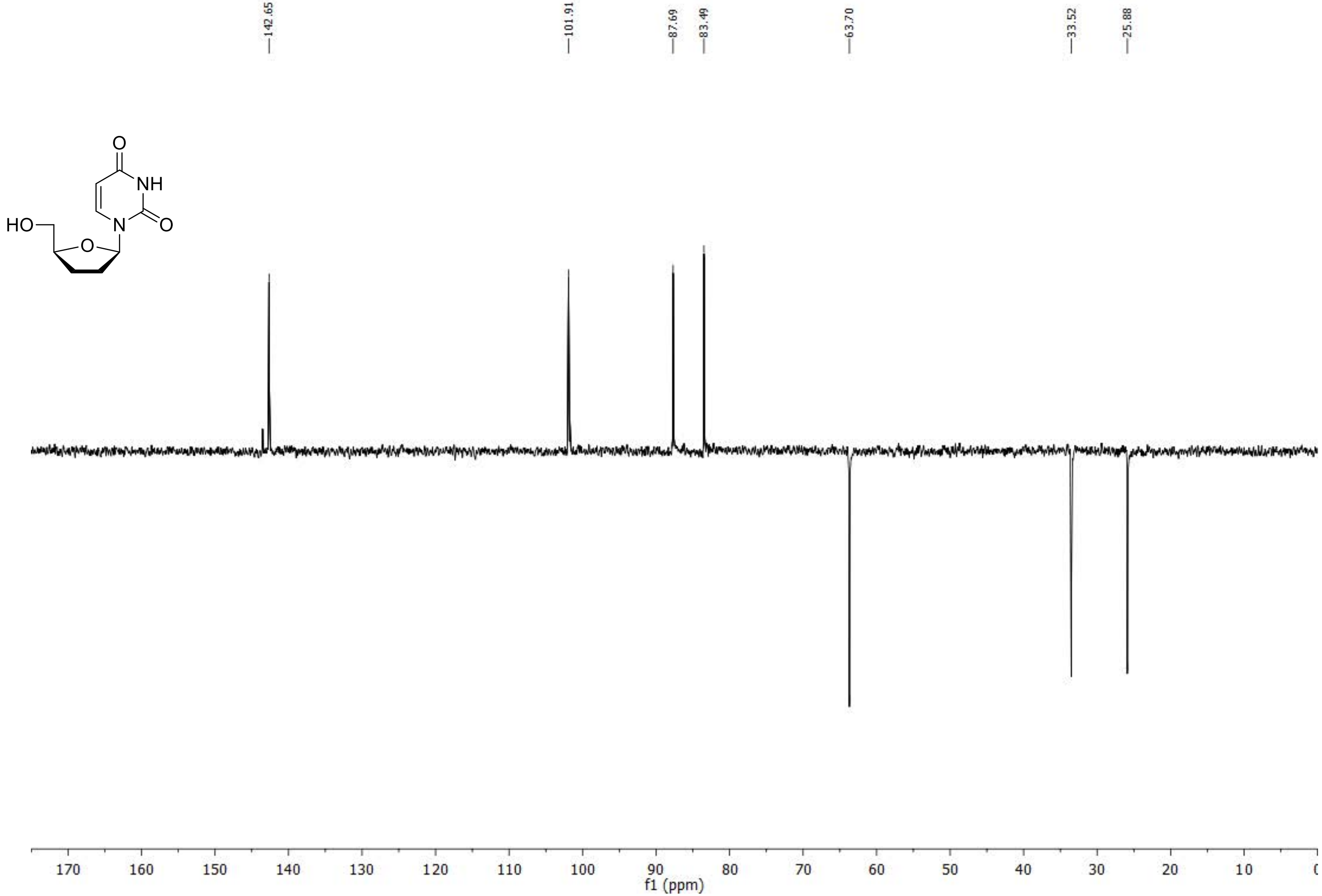

2’,3’-Dideoxy-β-D-uridine (9a)

COSY NMR (MeOH-d_4)

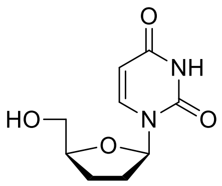

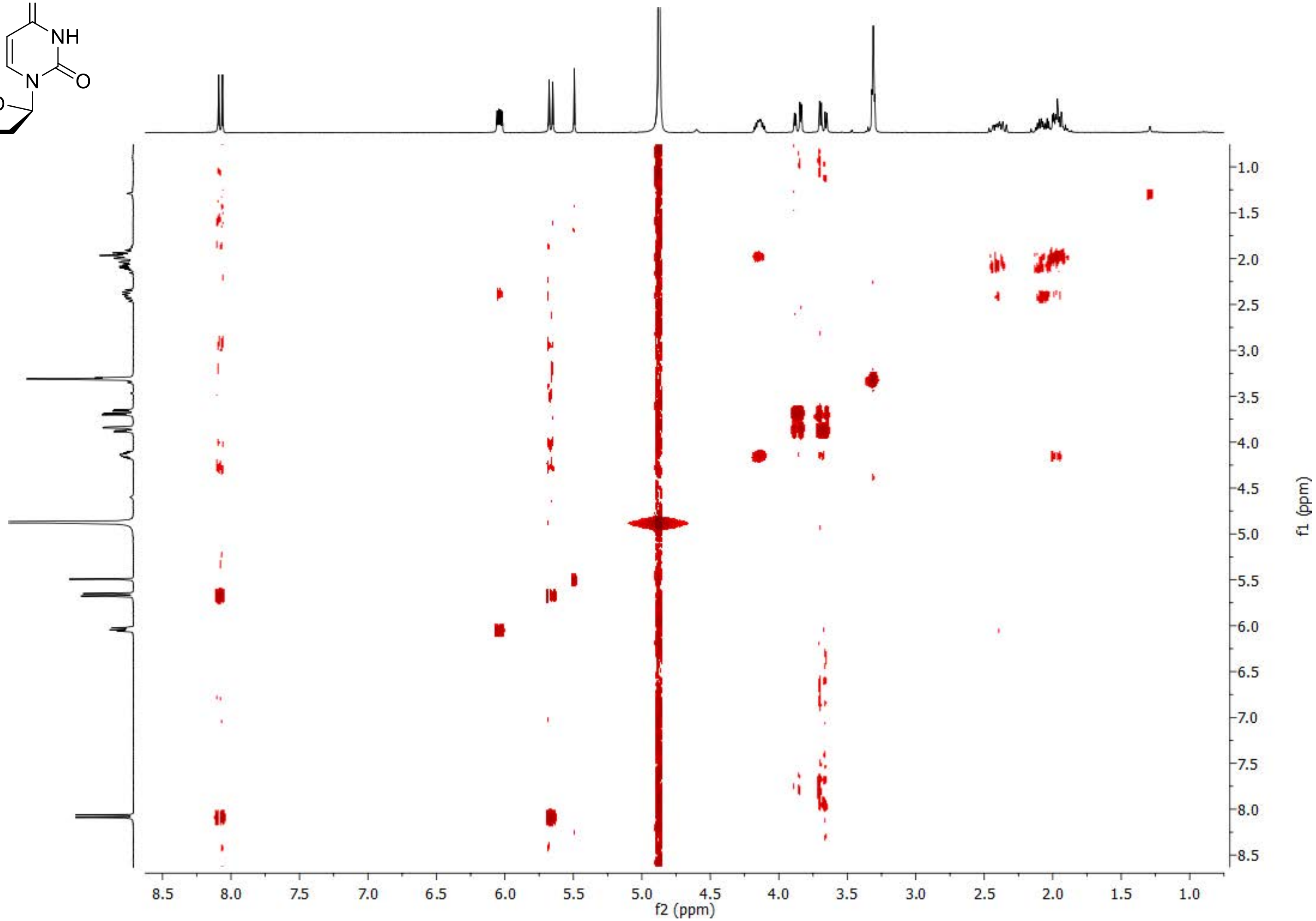

2’,3’-Dideoxy-β-D-uridine (9a)

HSQC NMR (MeOH-d_4)

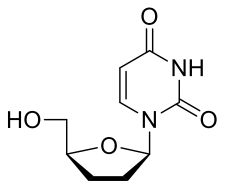

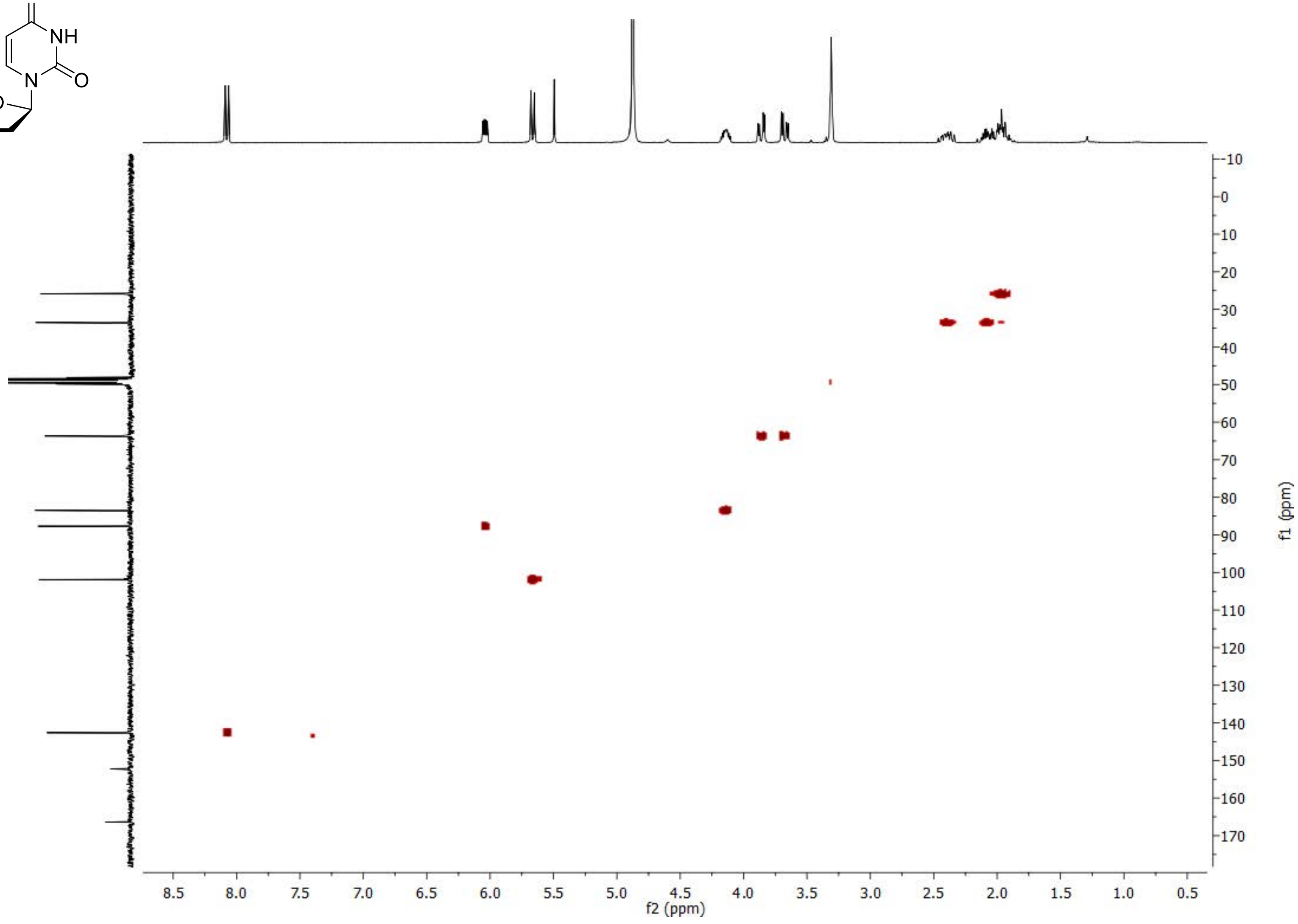

3’-Deoxy-β-D-5-thymidine (9b)

^{1}H-NMR (300.13 MHz, MeOH-d_4)

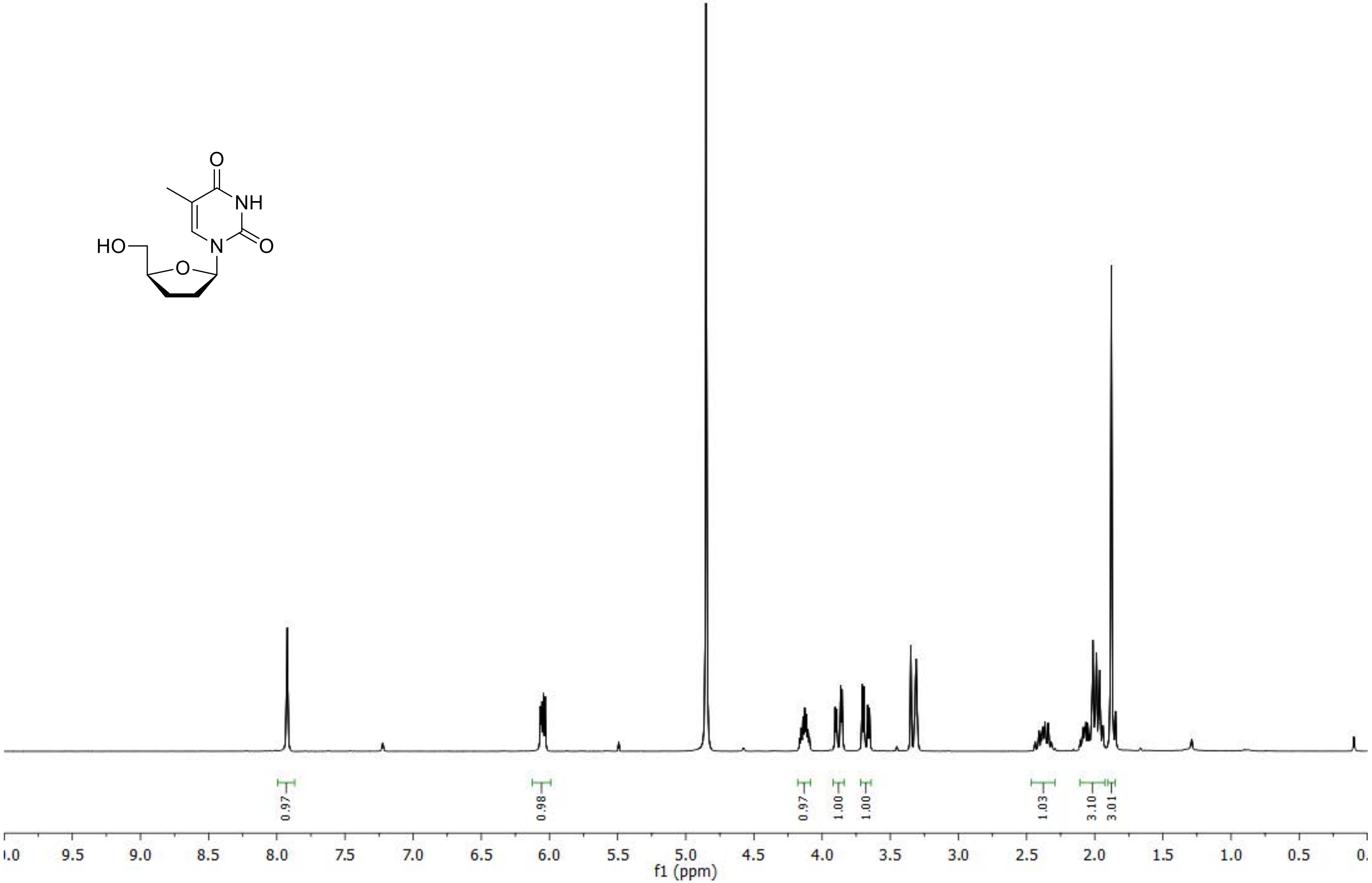

3’-Deoxy-β-D-5-thymidine (9b)

^{13}C-NMR (75.5 MHz, MeOH-d_4)

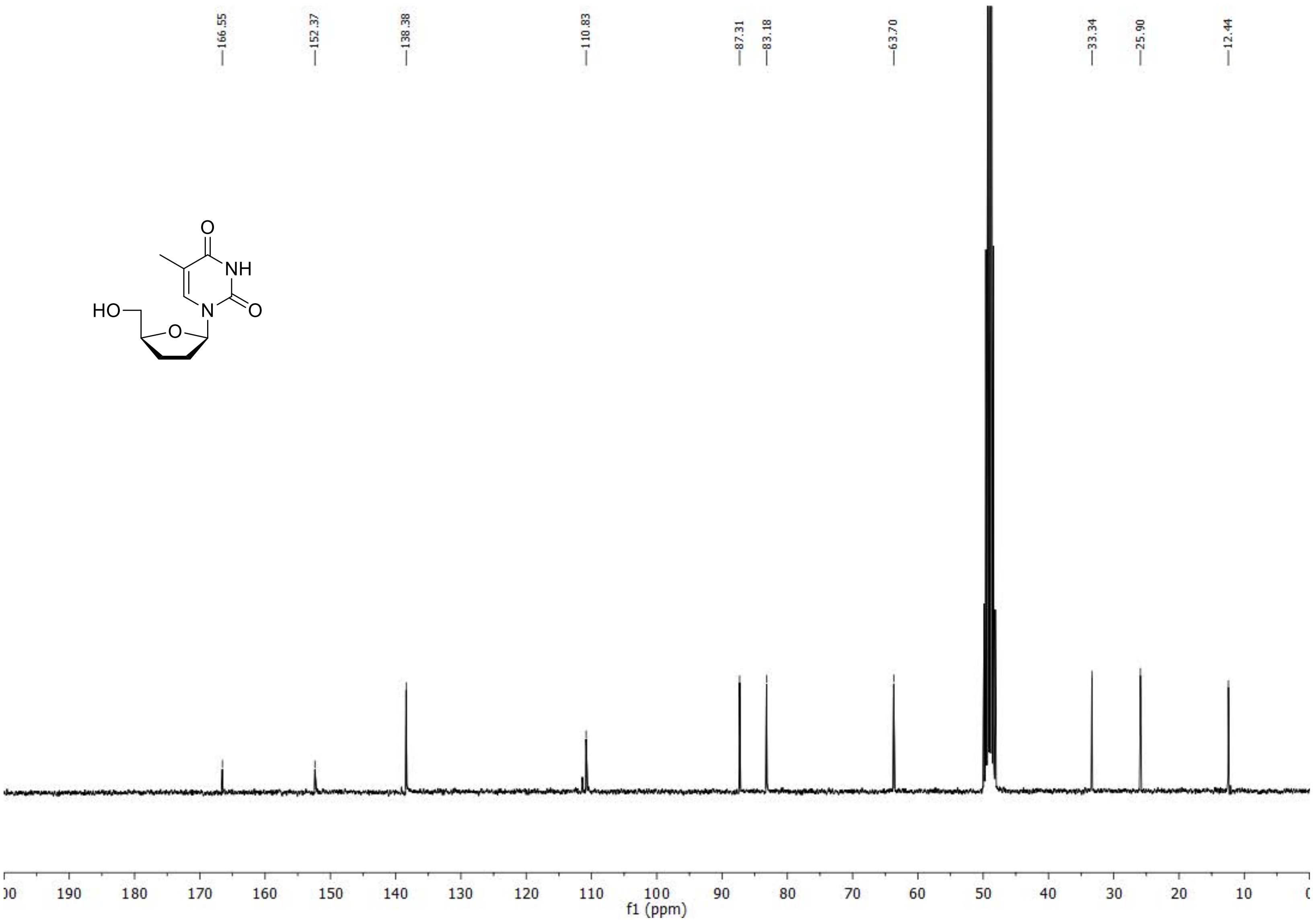

3’-Deoxy-β-D-5-thymidine (9b)

DEPT NMR (75.5 MHz, MeOH-d_4)

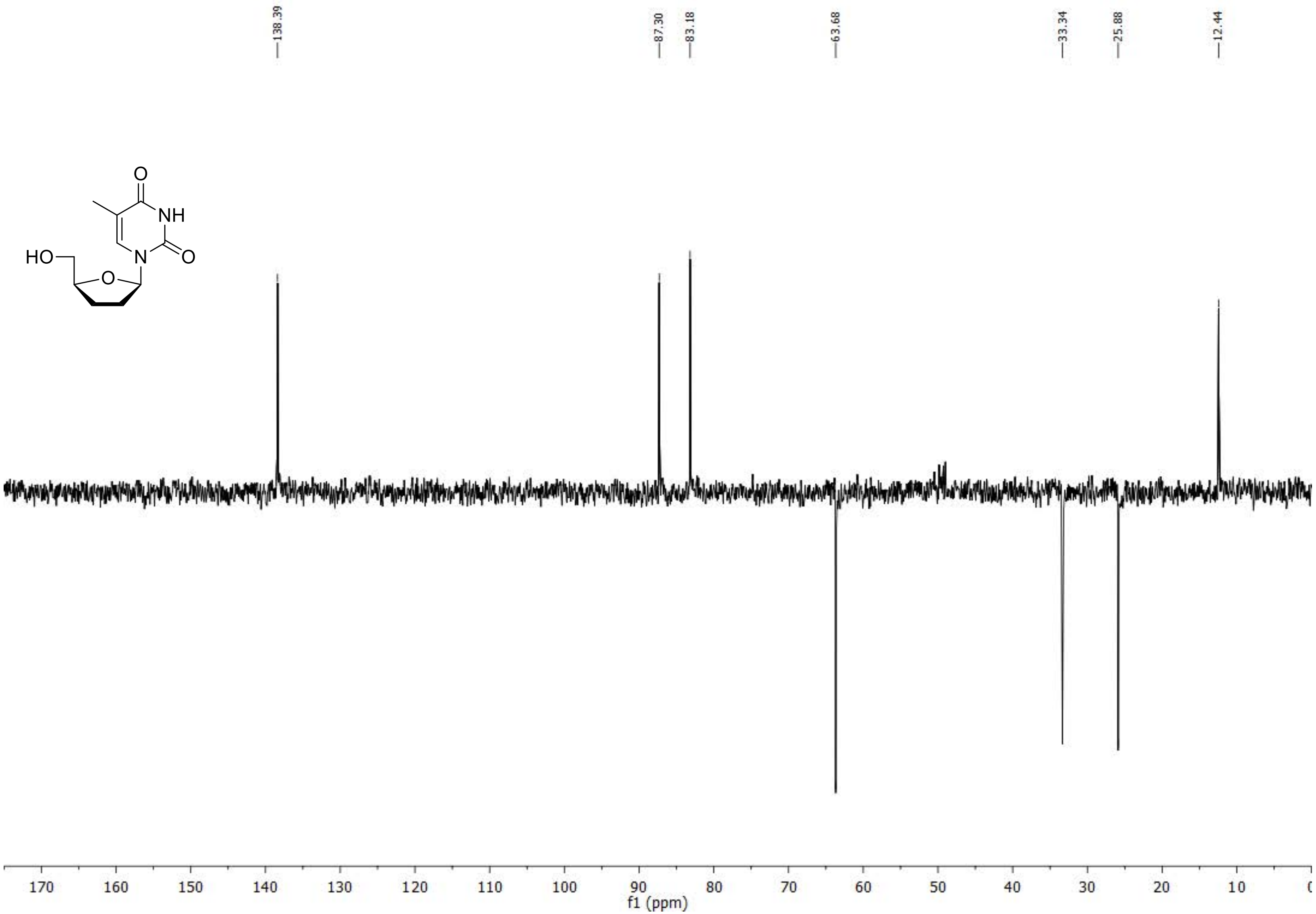

COSY NMR (MeOH-d_4)

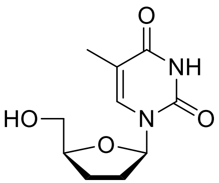

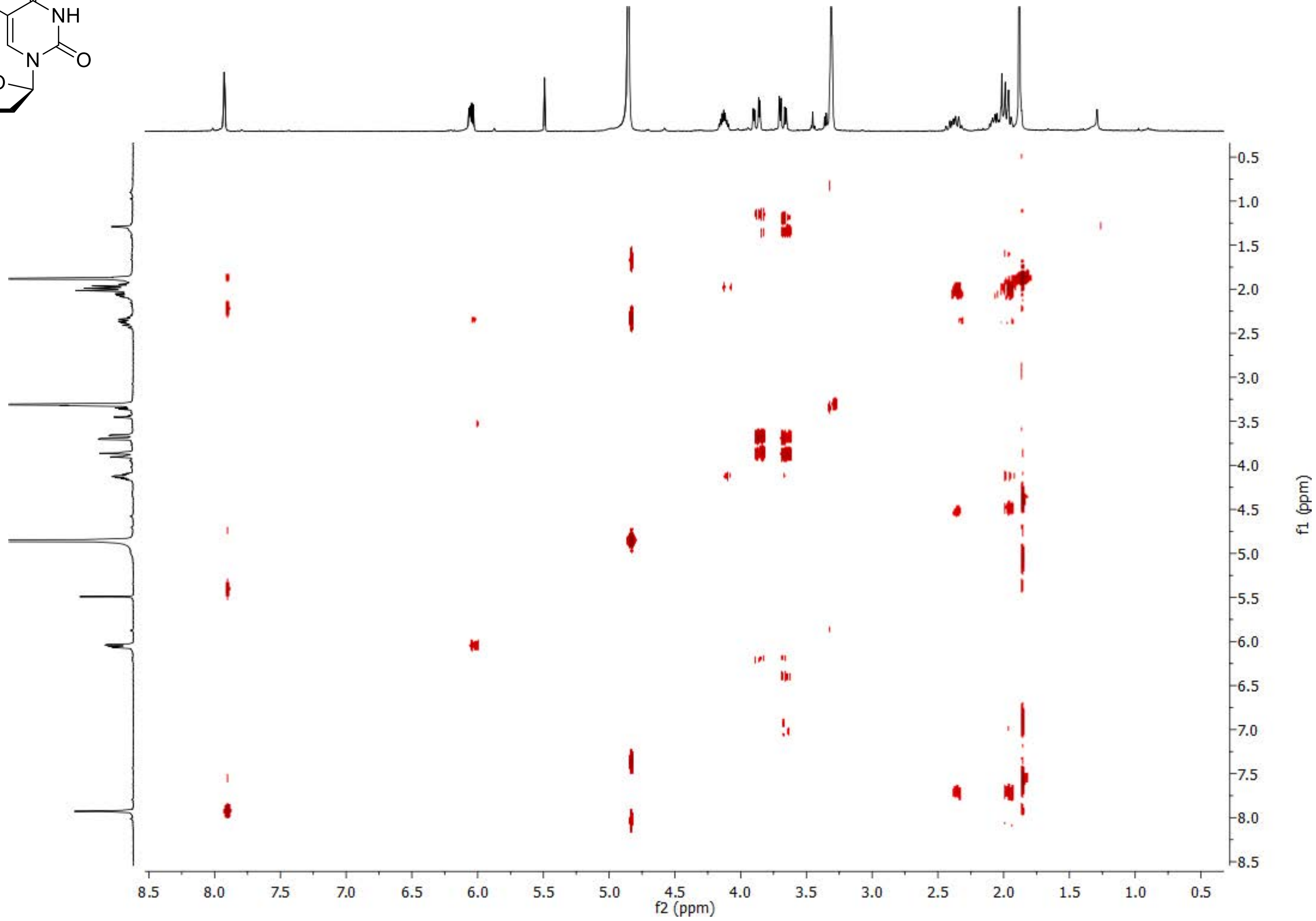

3’-Deoxy-β-D-5-thymidine (9b)

HSQC NMR (MeOH-d_4)

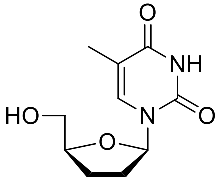

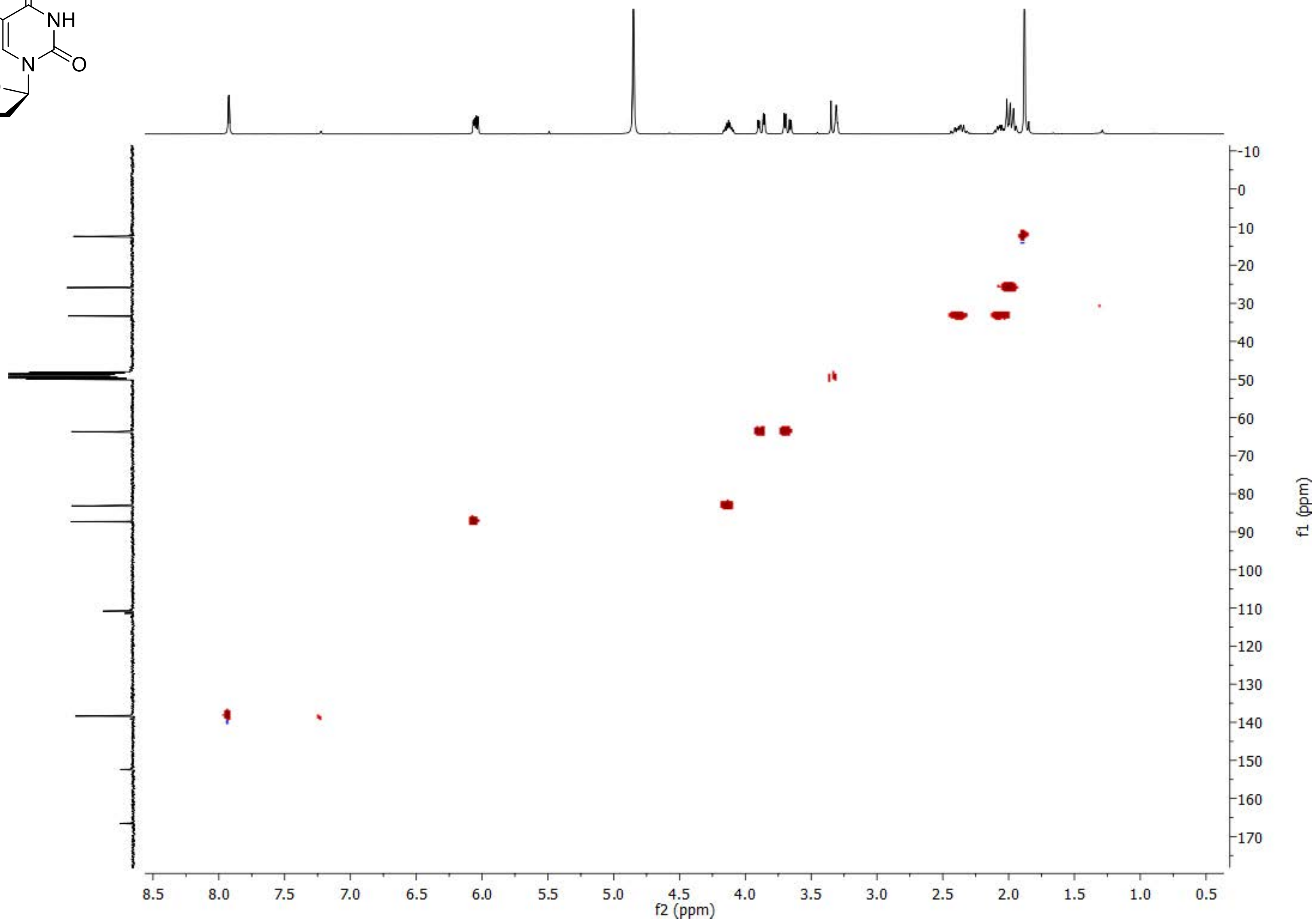

2’,3’-Dideoxy-β-D-cytidine (9c)

^{1}H-NMR (300.13 MHz, D_2O)

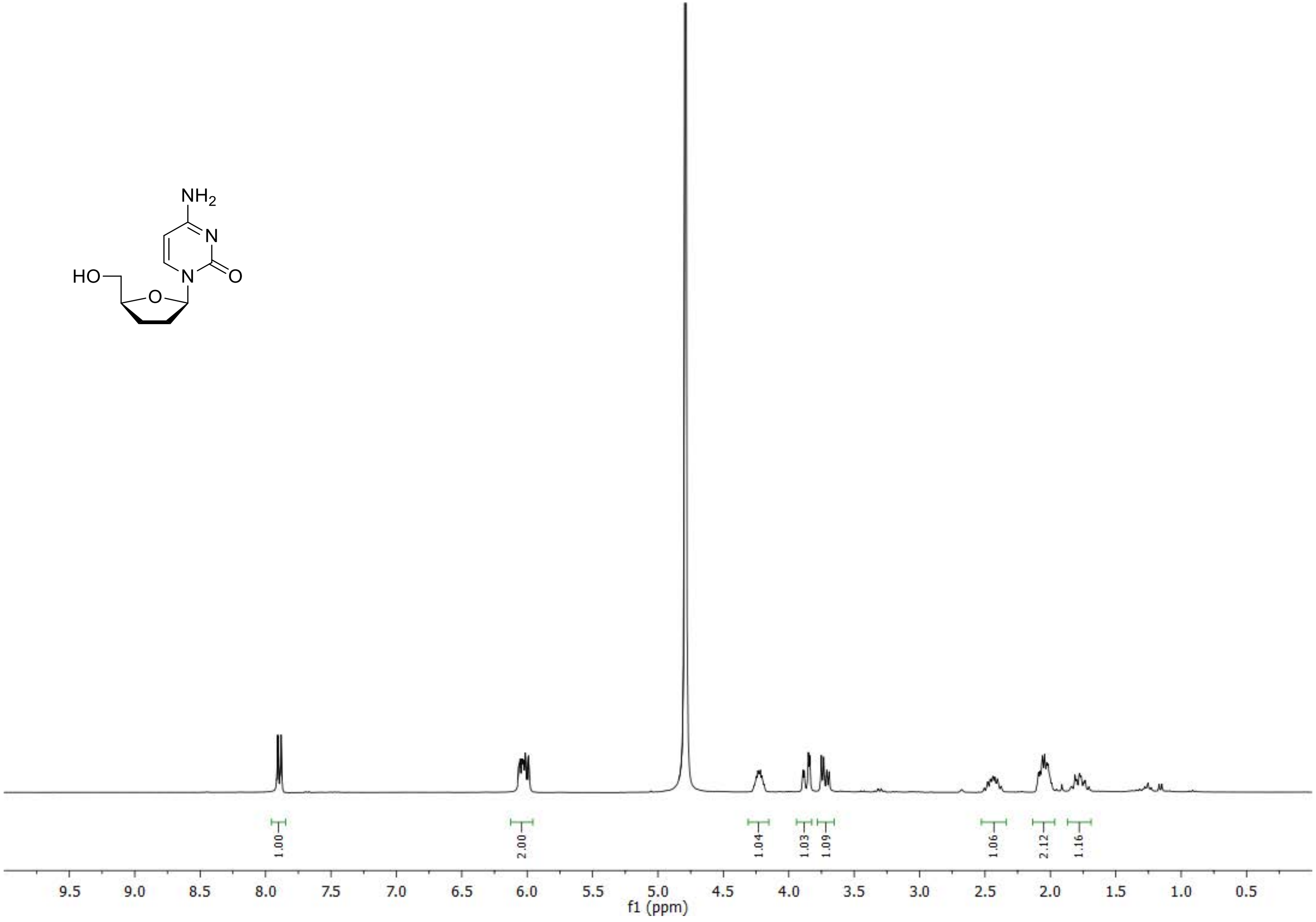

2’,3’-Dideoxy-β-D-cytidine (9c)

^{13}C-NMR (75.5 MHz, D_2O)

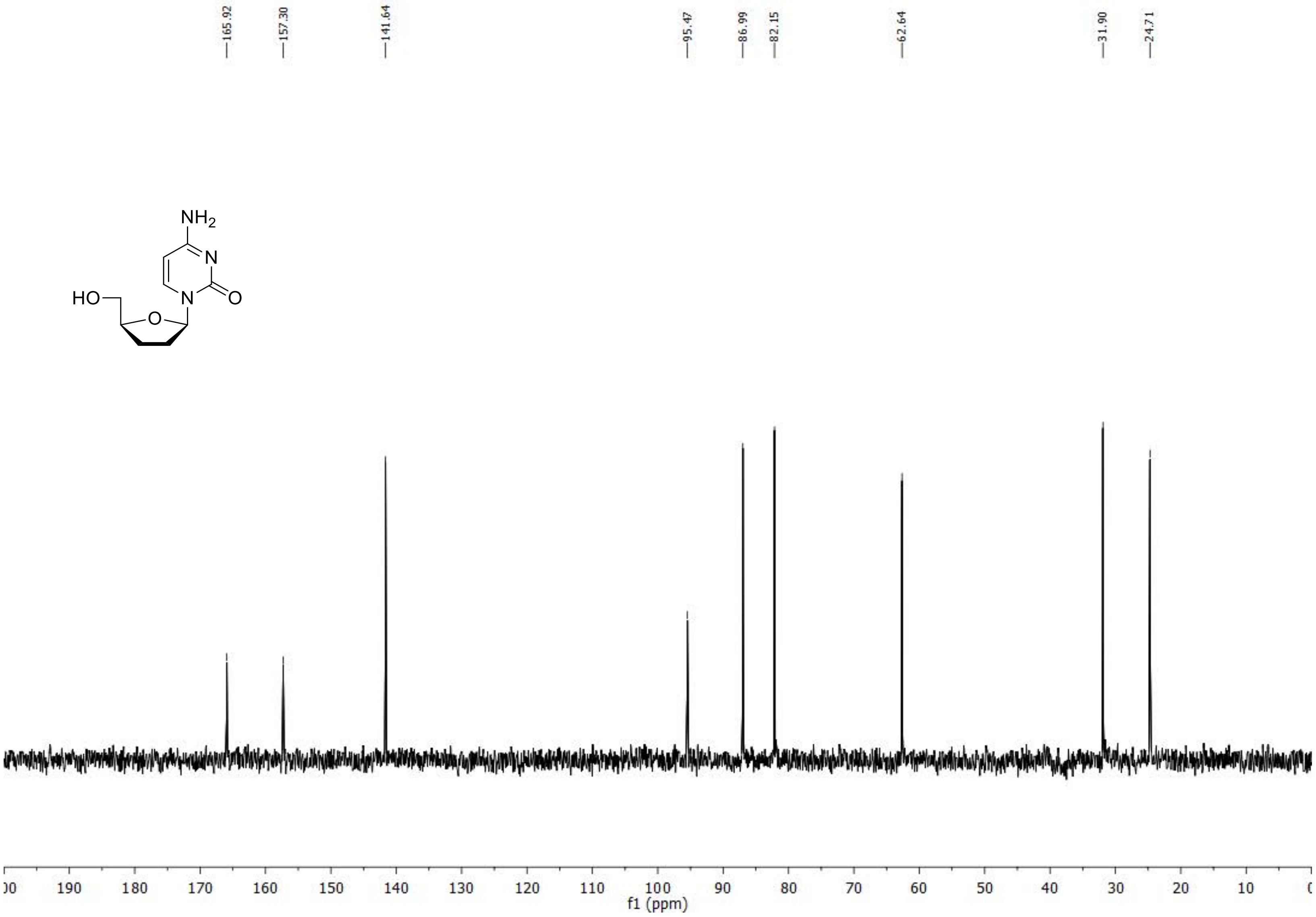

DEPT (75.5 MHz, D_2O)

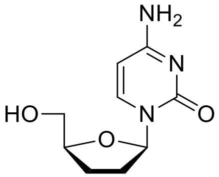

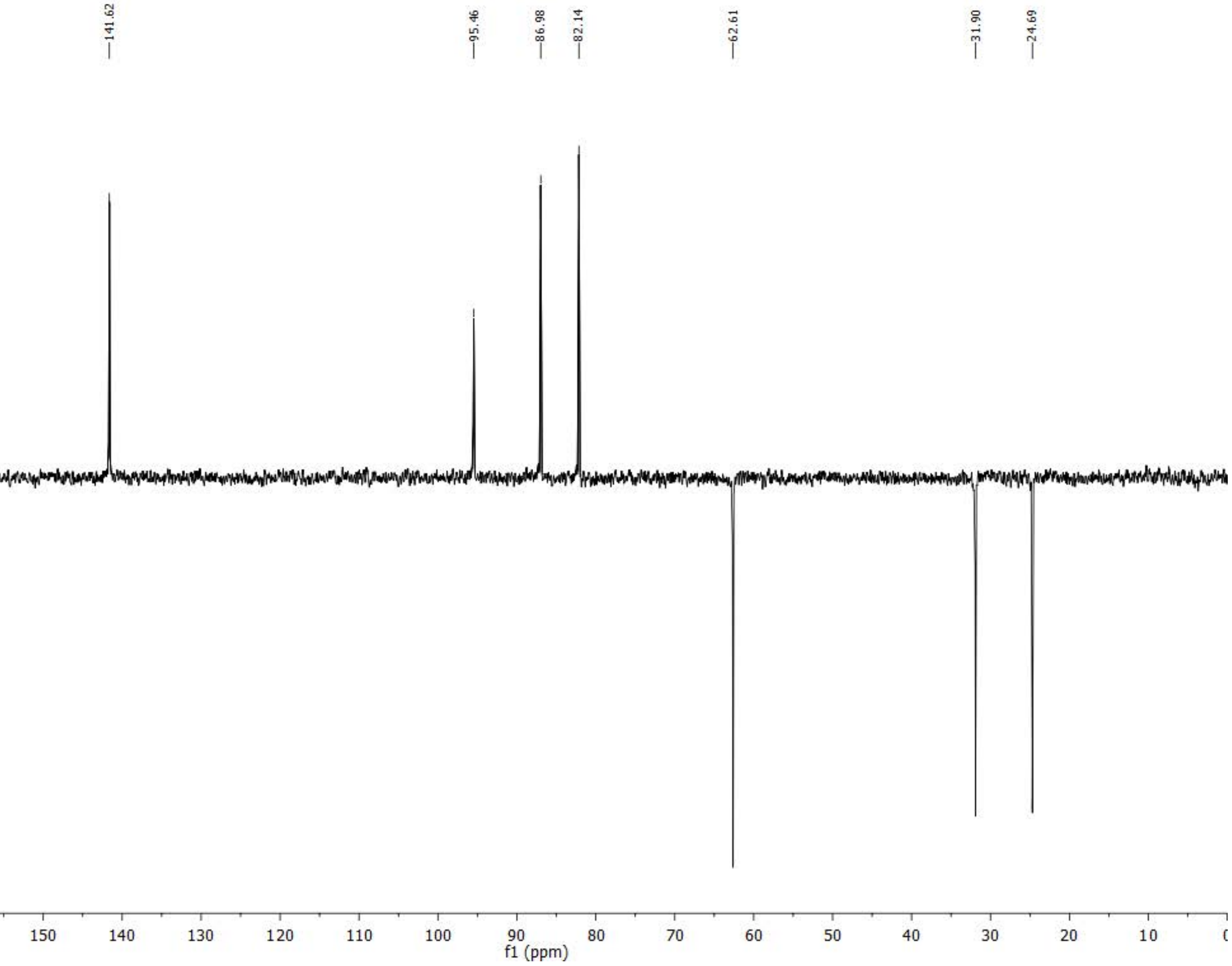

2',3'-Dideoxy-β-D-cytidine (9c)

COSY NMR (D_2O)

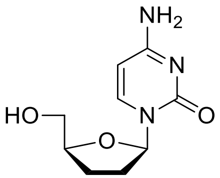

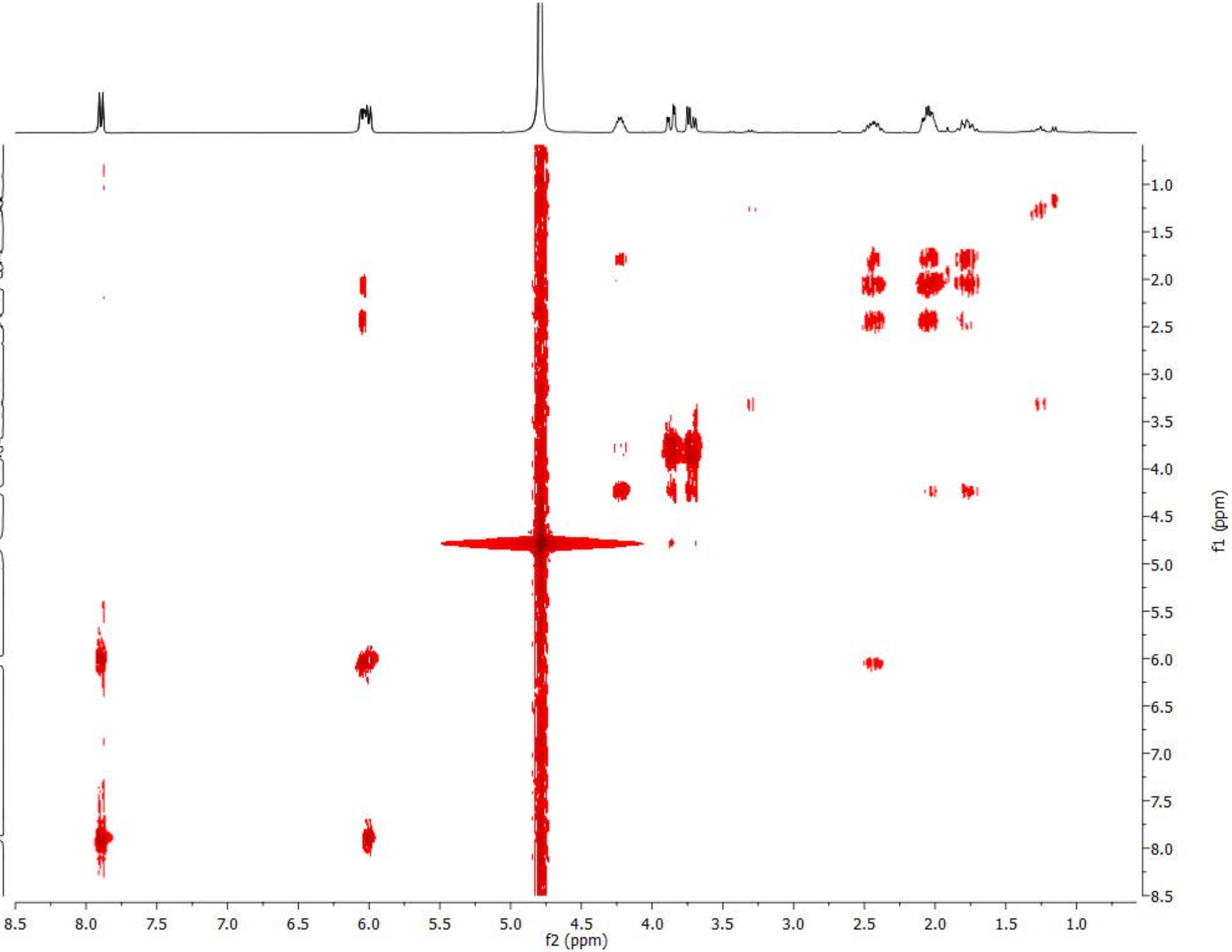

2’,3’-Dideoxy-β-D-cytidine (9c)

HSQC NMR (D_2O)

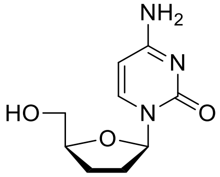

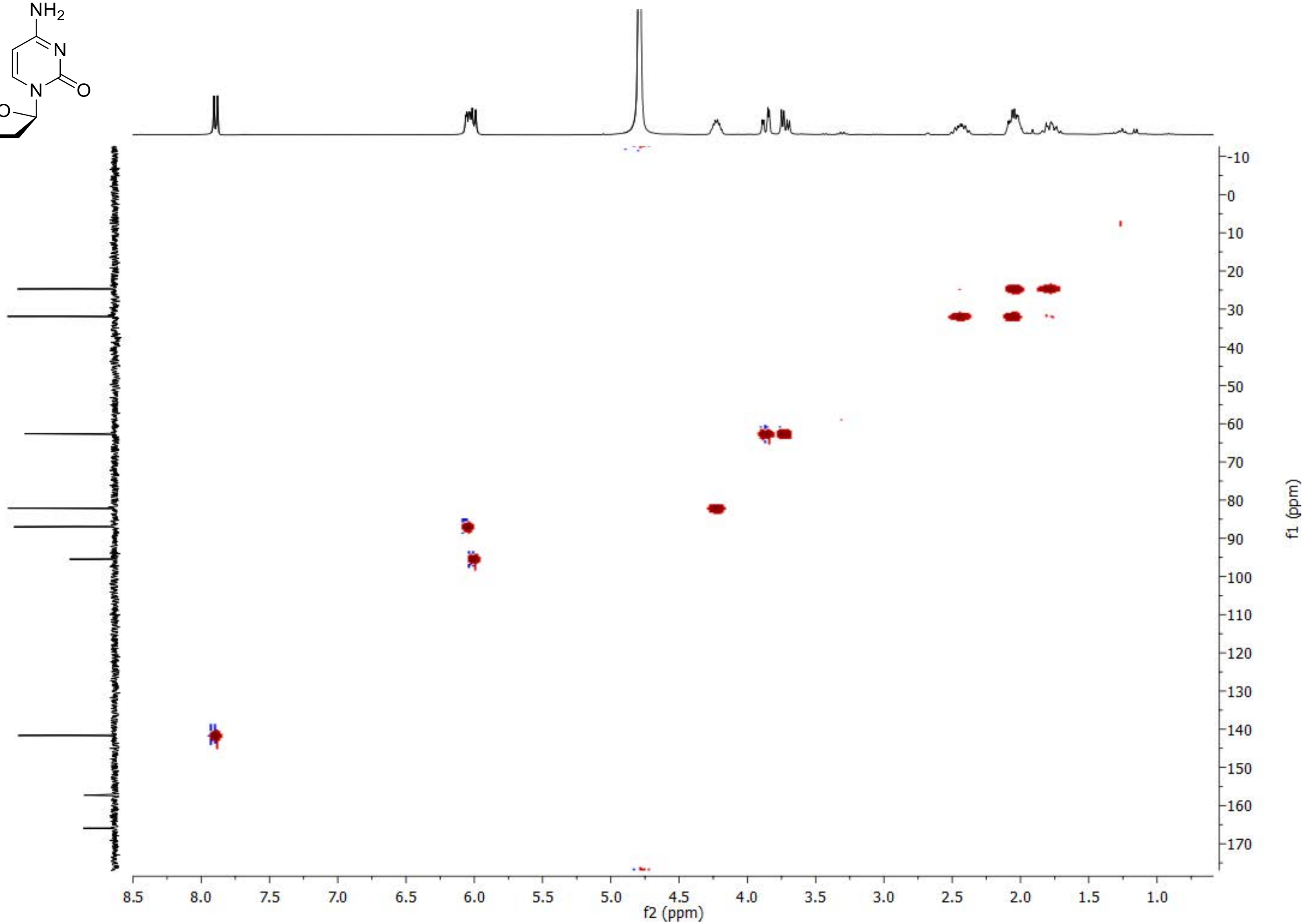

2’,3’-Dideoxy-β-D-cytidine (9c)

HMBC NMR (D_2O)

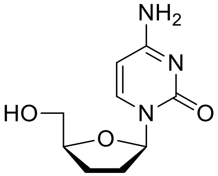

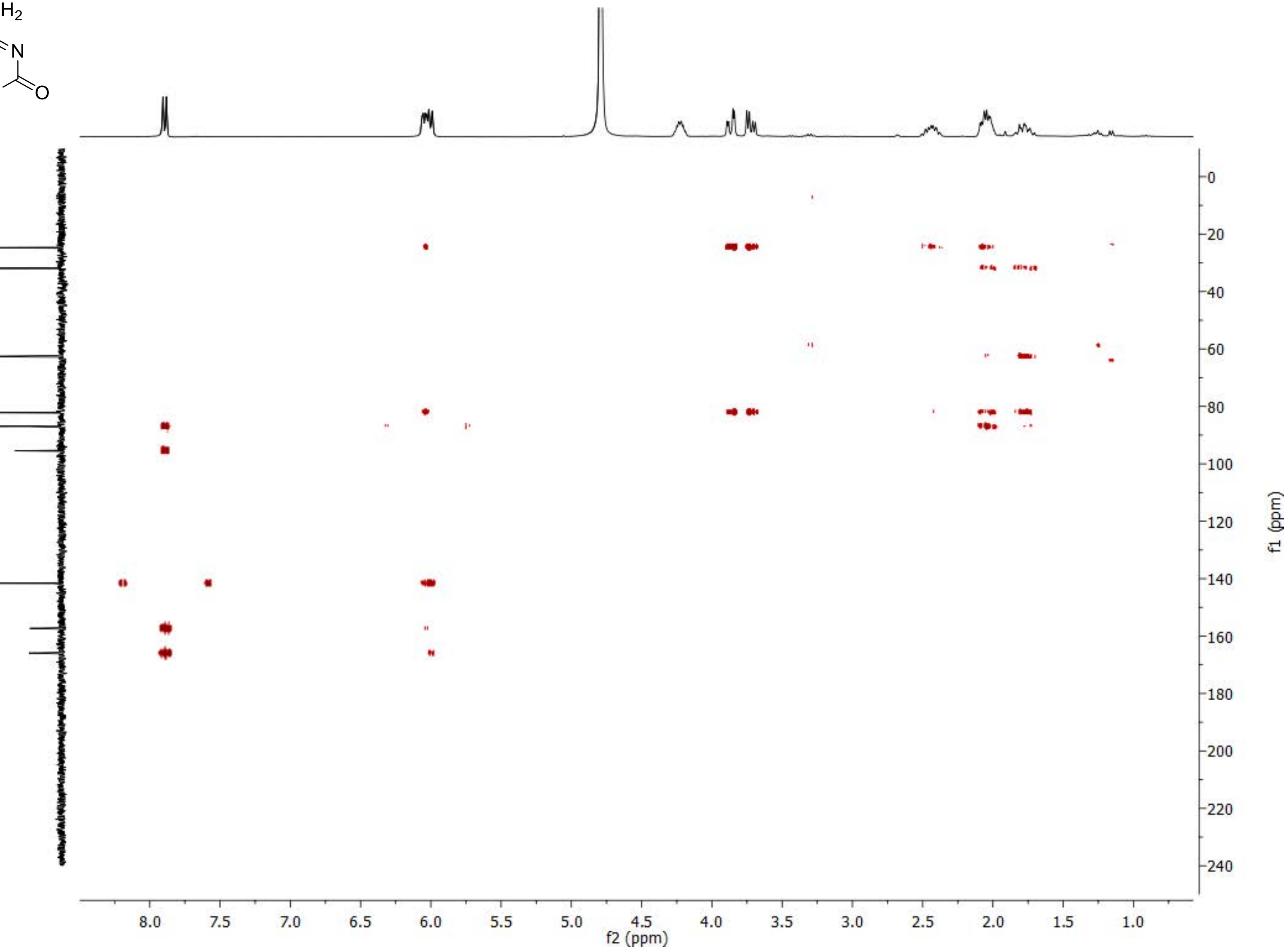

2',3'-Dideoxy-β-D-adenosine (9e)

^{1}H-NMR (300.13 MHz, MeOH-*d*4)

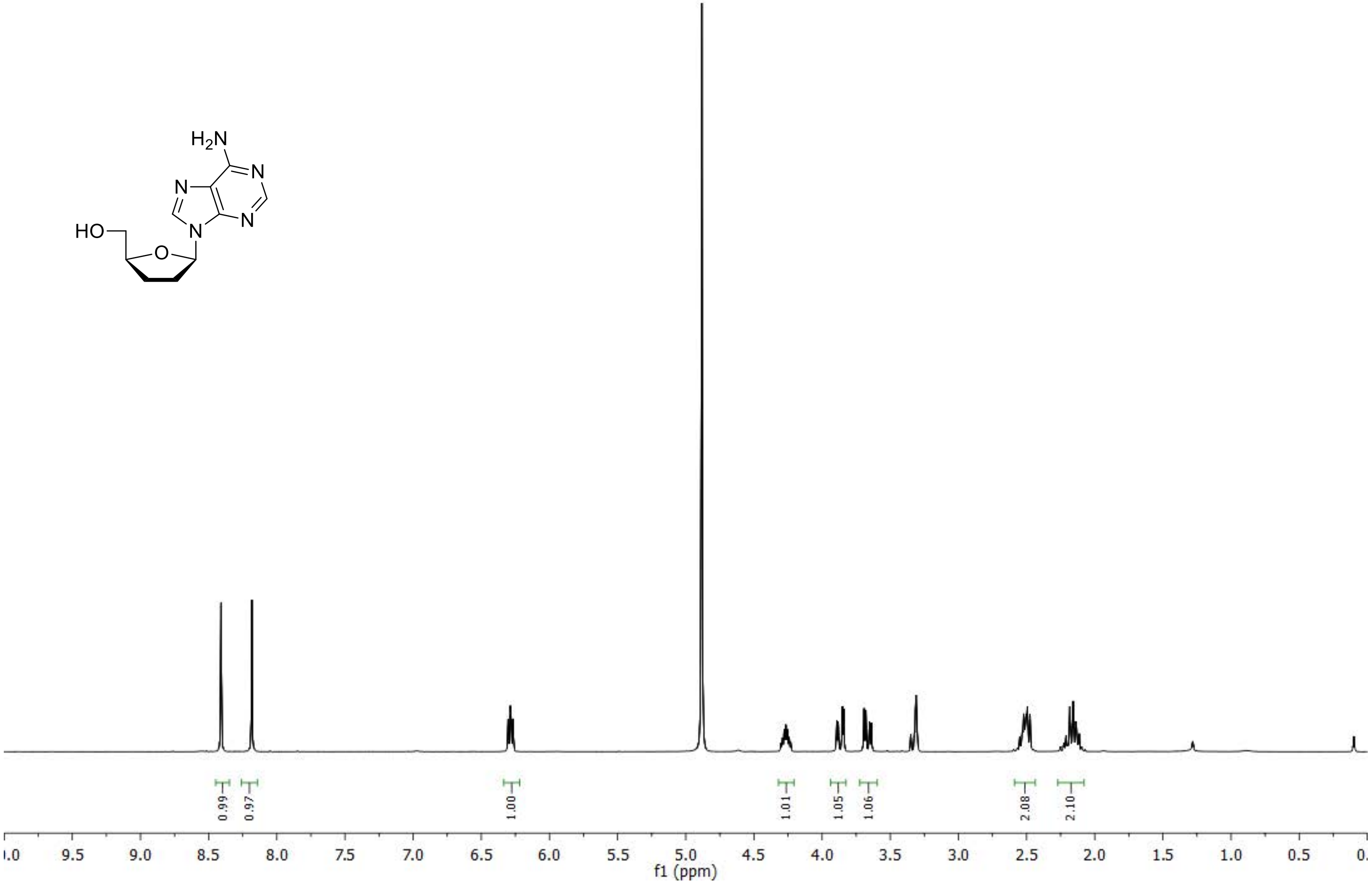

2’,3’-Dideoxy-β-D-adenosine (9e)

^{13}C-NMR (75.5 MHz, MeOH-d_4)

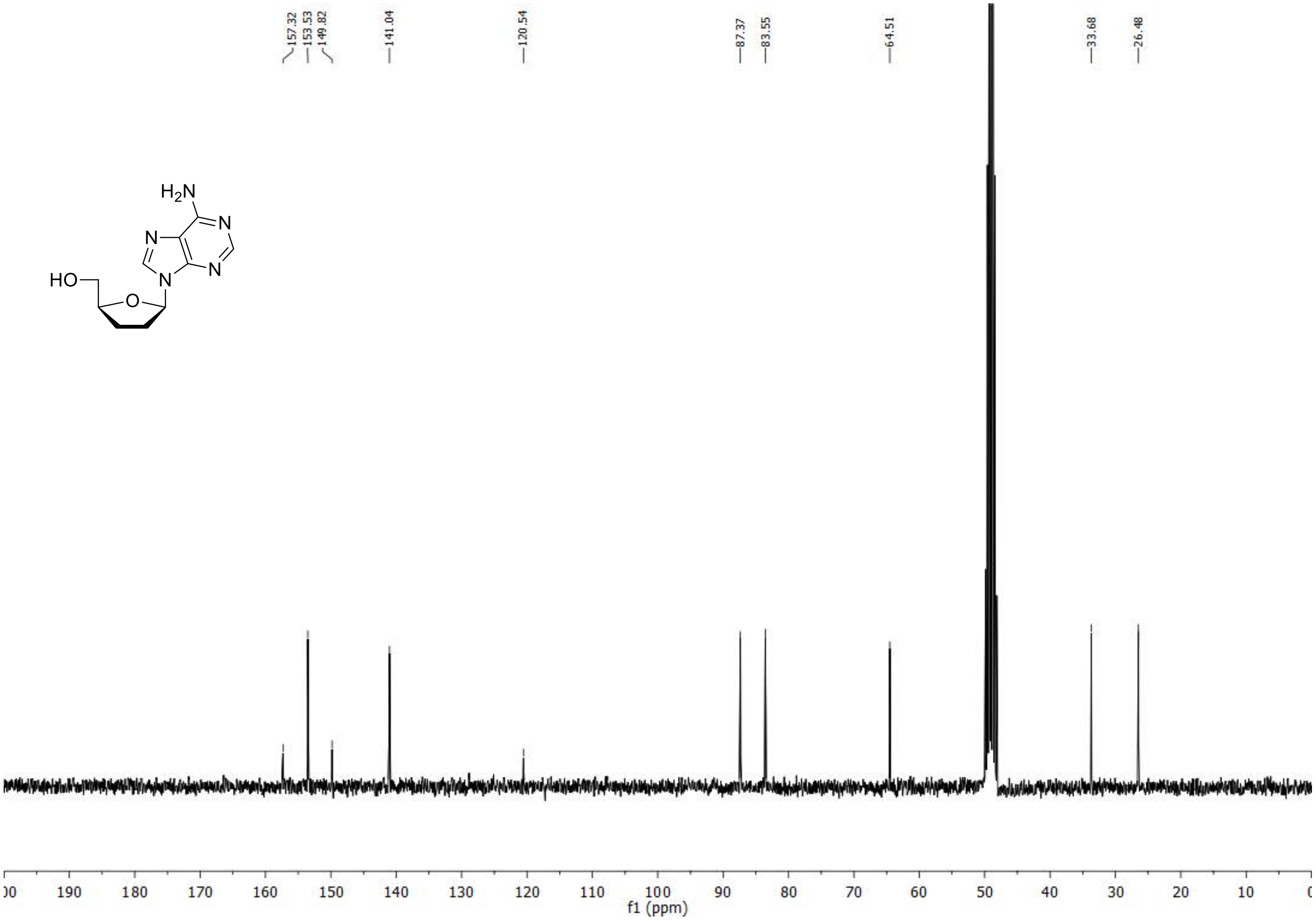

2’,3’-Dideoxy-β-D-adenosine (9e)

DEPT NMR (75.5 MHz, MeOH-d_4)

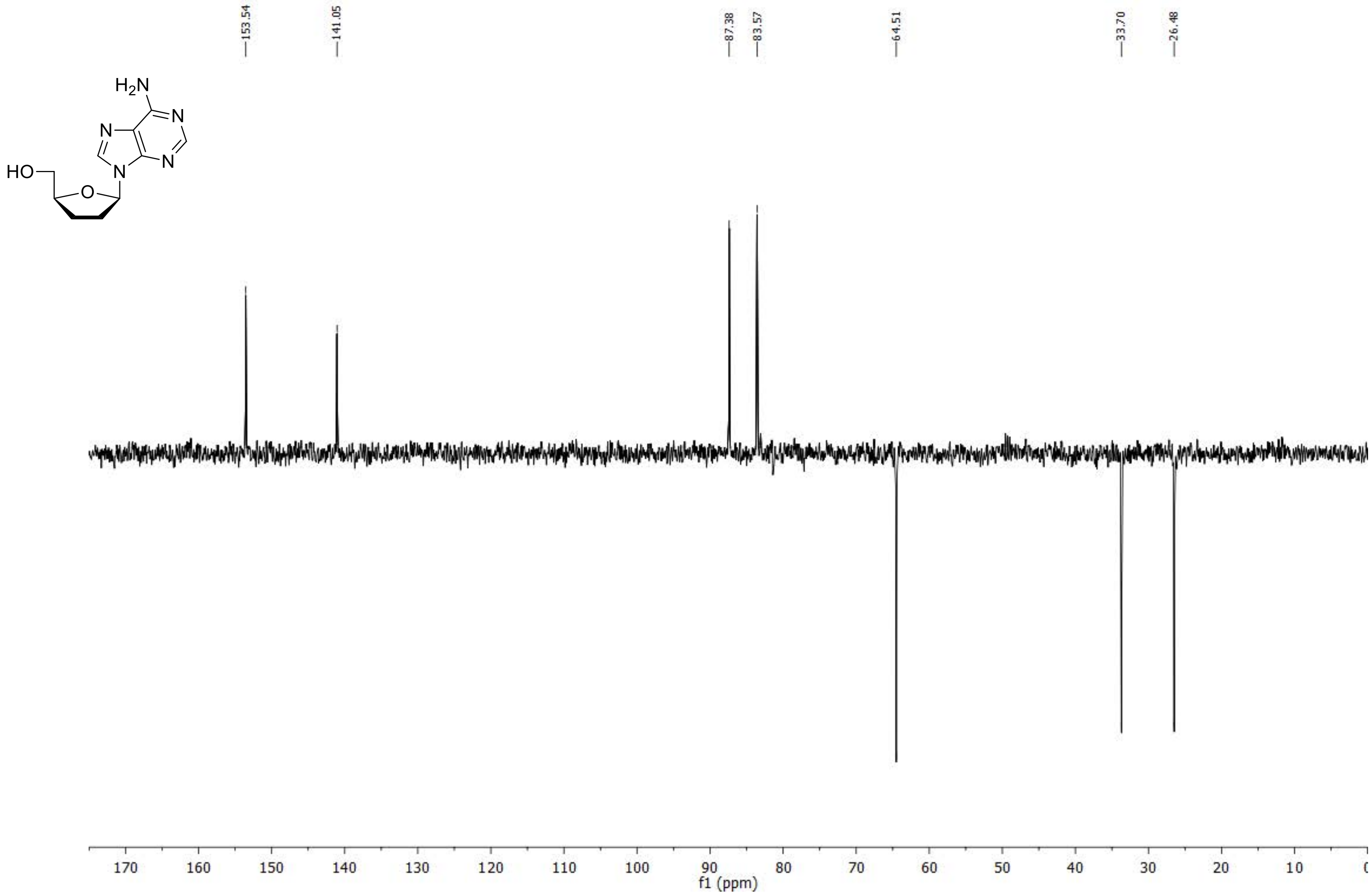

2’,3’-Dideoxy-β-D-adenosine (9e)

COSY NMR (MeOH-d_4)

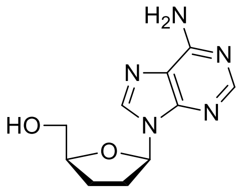

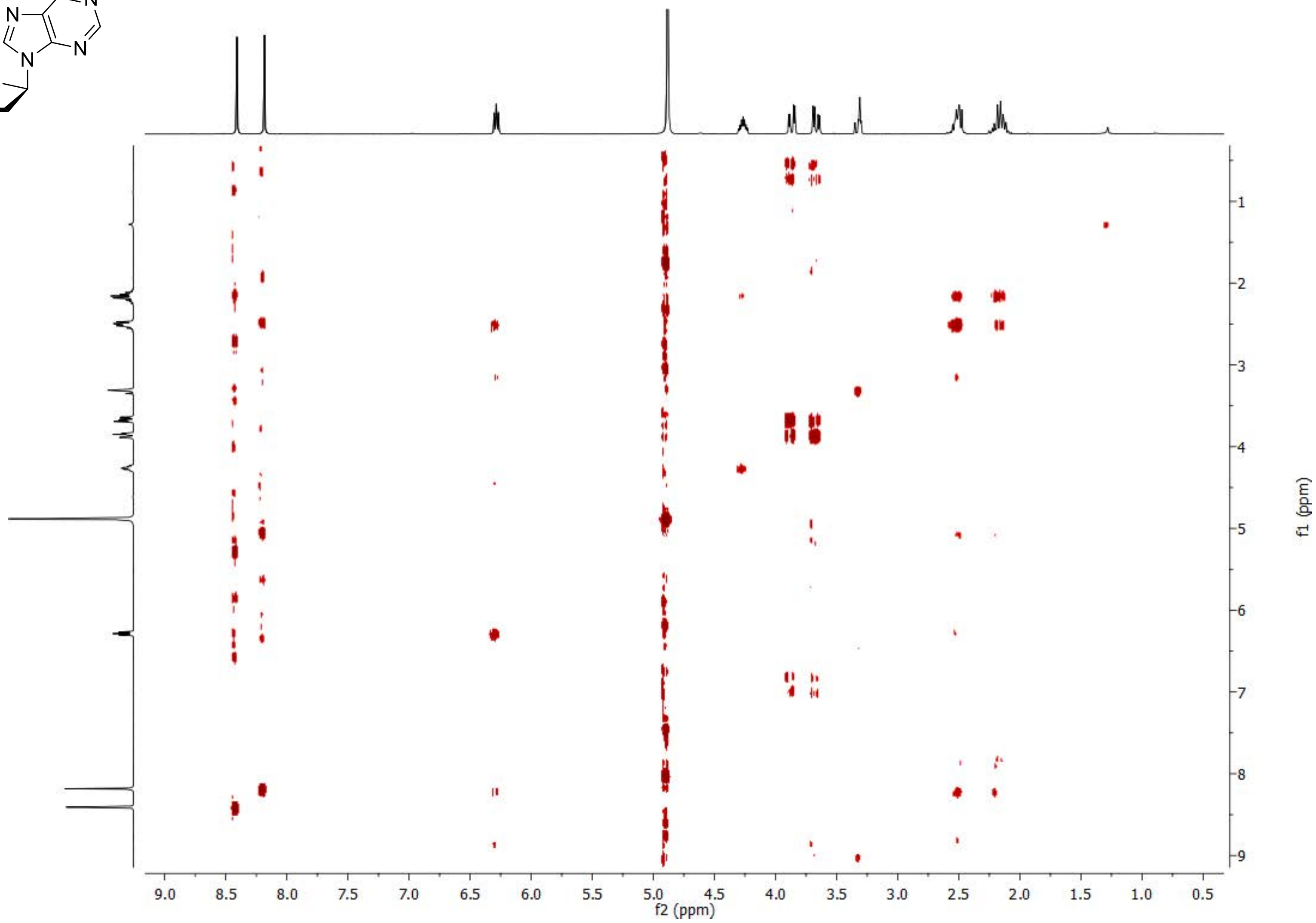

2’,3’-Dideoxy-β-D-adenosine (9e)

HSQC NMR (MeOH-d_4)

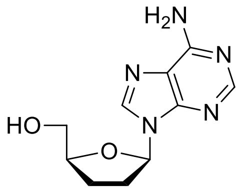

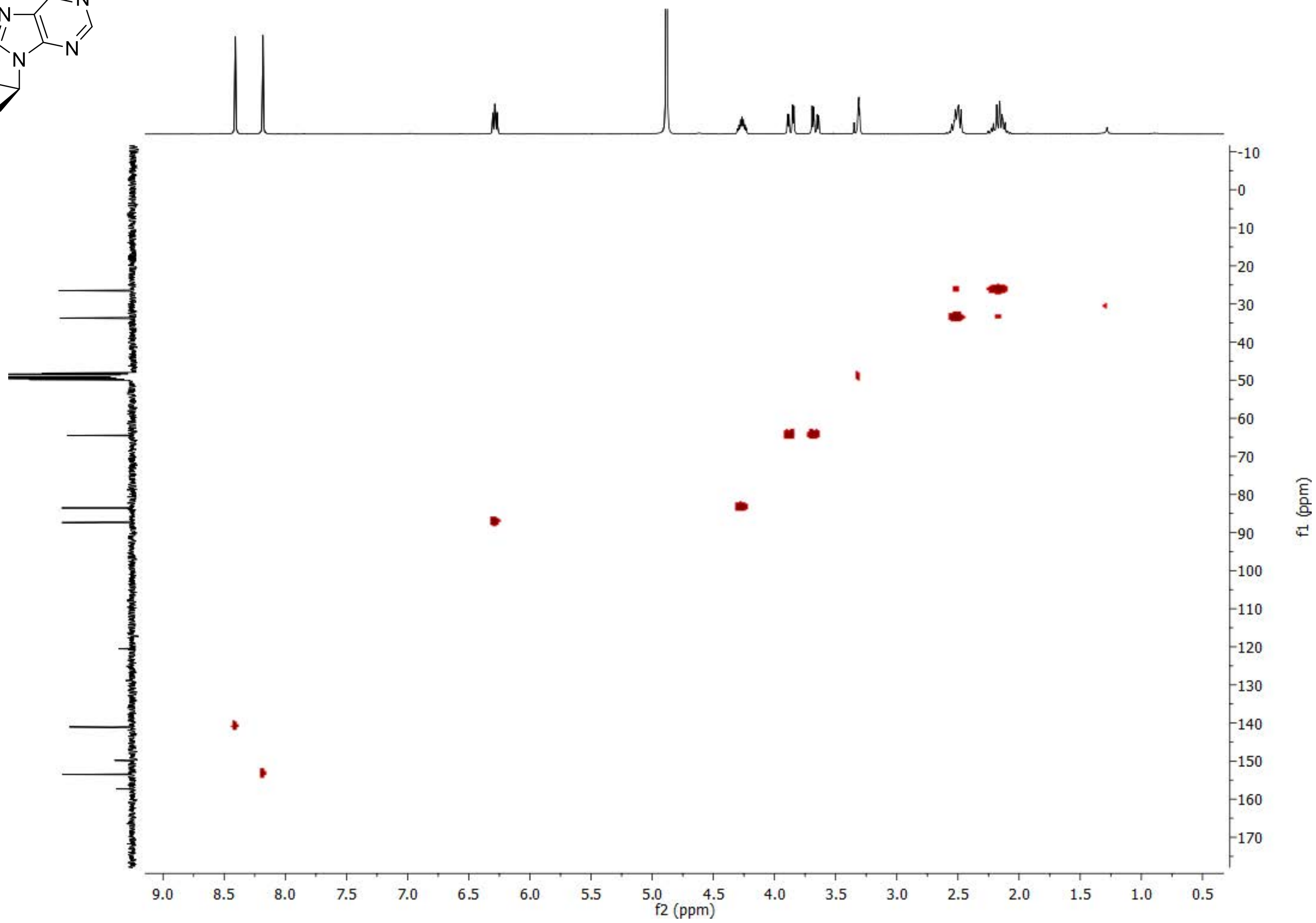

2’,3’-Dideoxy-β-D-adenosine (9e)

HMBC NMR (MeOH-d_4)

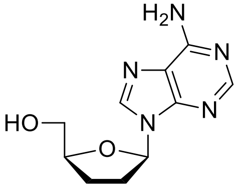

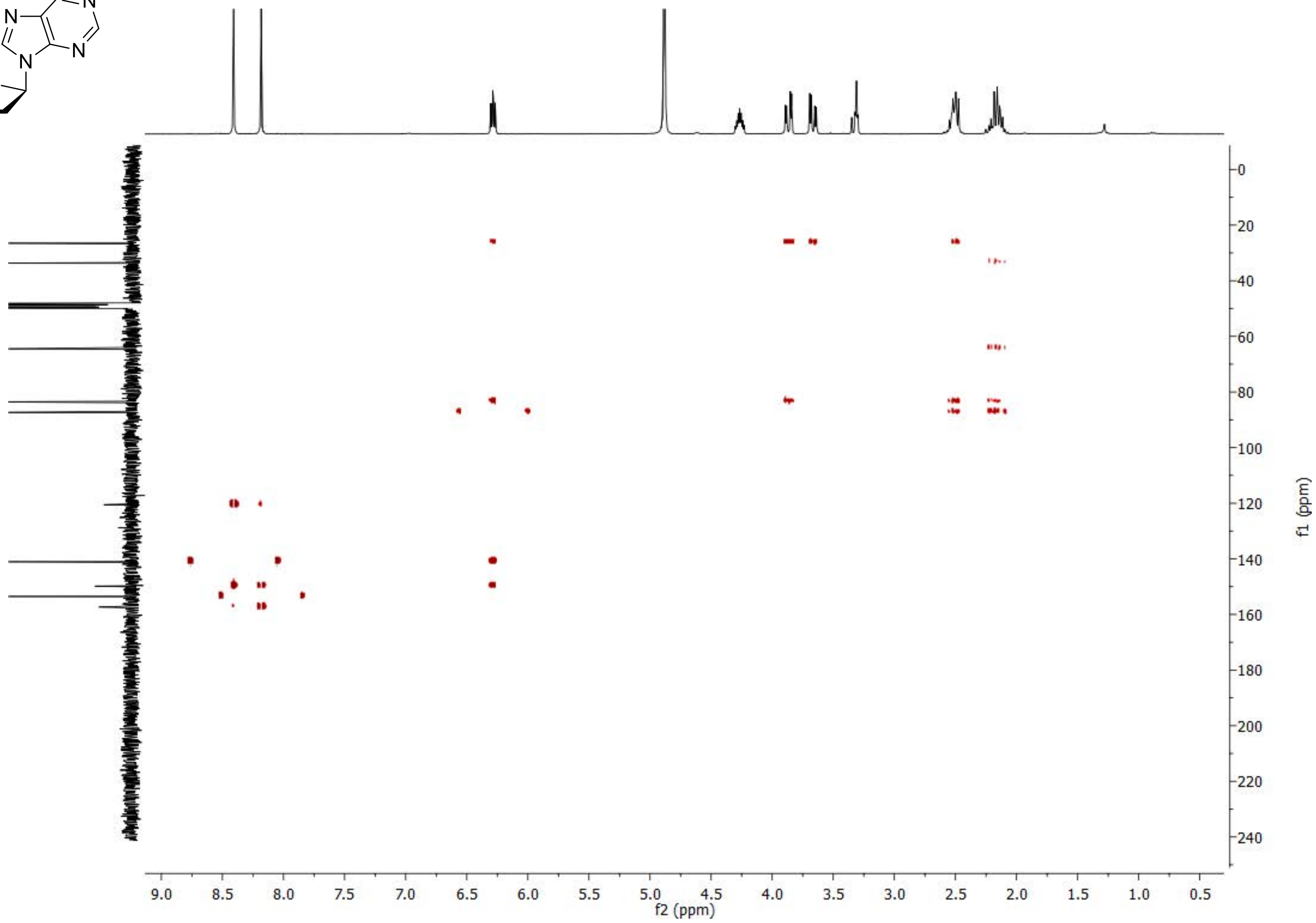

2’,3’-Dideoxy-β-D-inosine (9f)

^{1}H-NMR (300.13 MHz, MeOH-d_4)

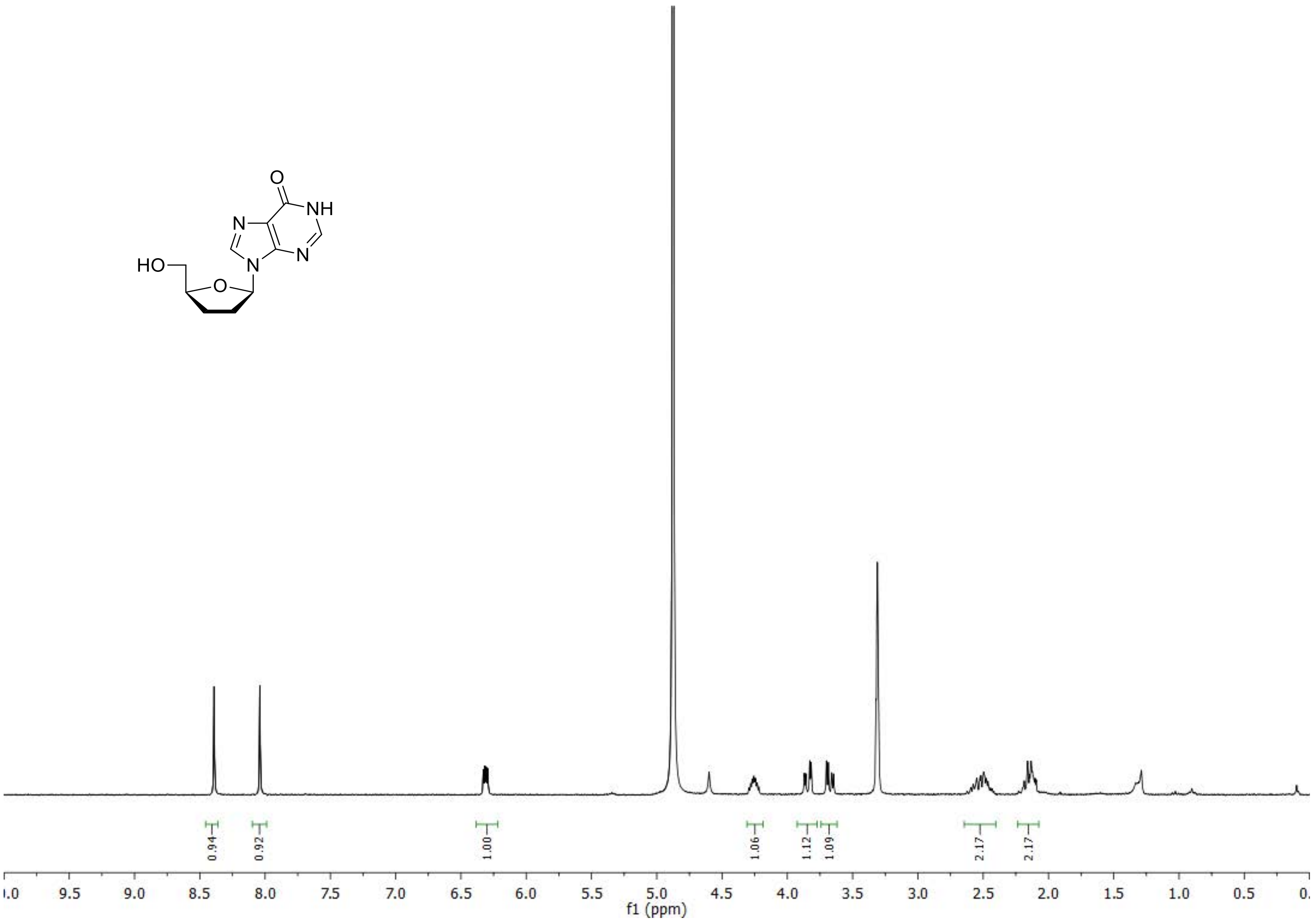

2',3'-Dideoxy-β-D-inosine (9f)

^{13}C-NMR (75.5 MHz, MeOH-d_4)

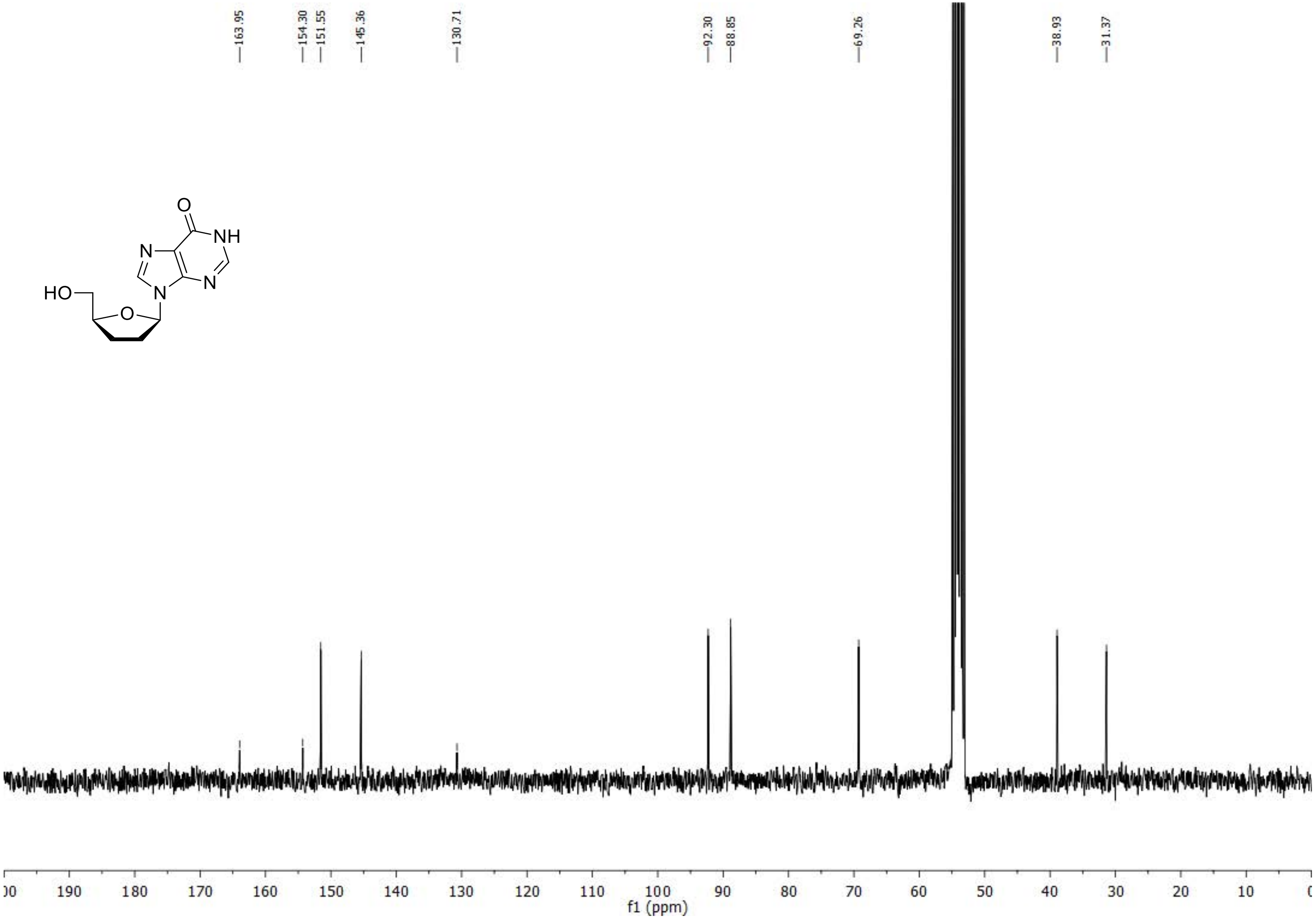

2',3'-Dideoxy-β-D-inosine (9f)

COSY NMR (MeOH-d_4)

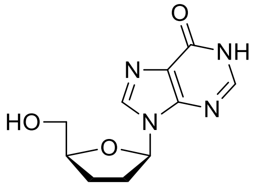

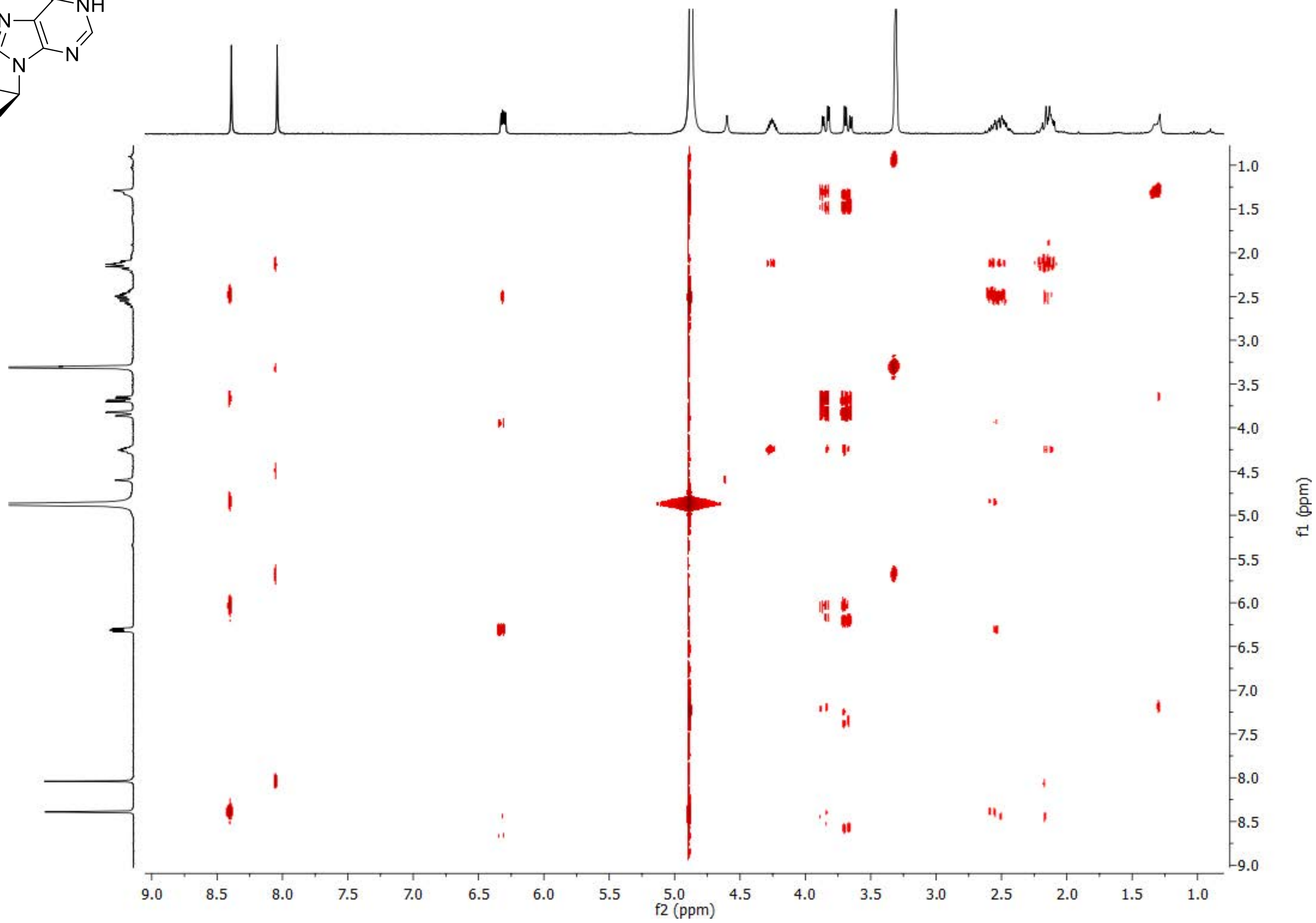

HSQC NMR (MeOH-d_4)

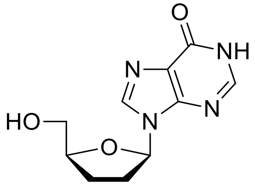

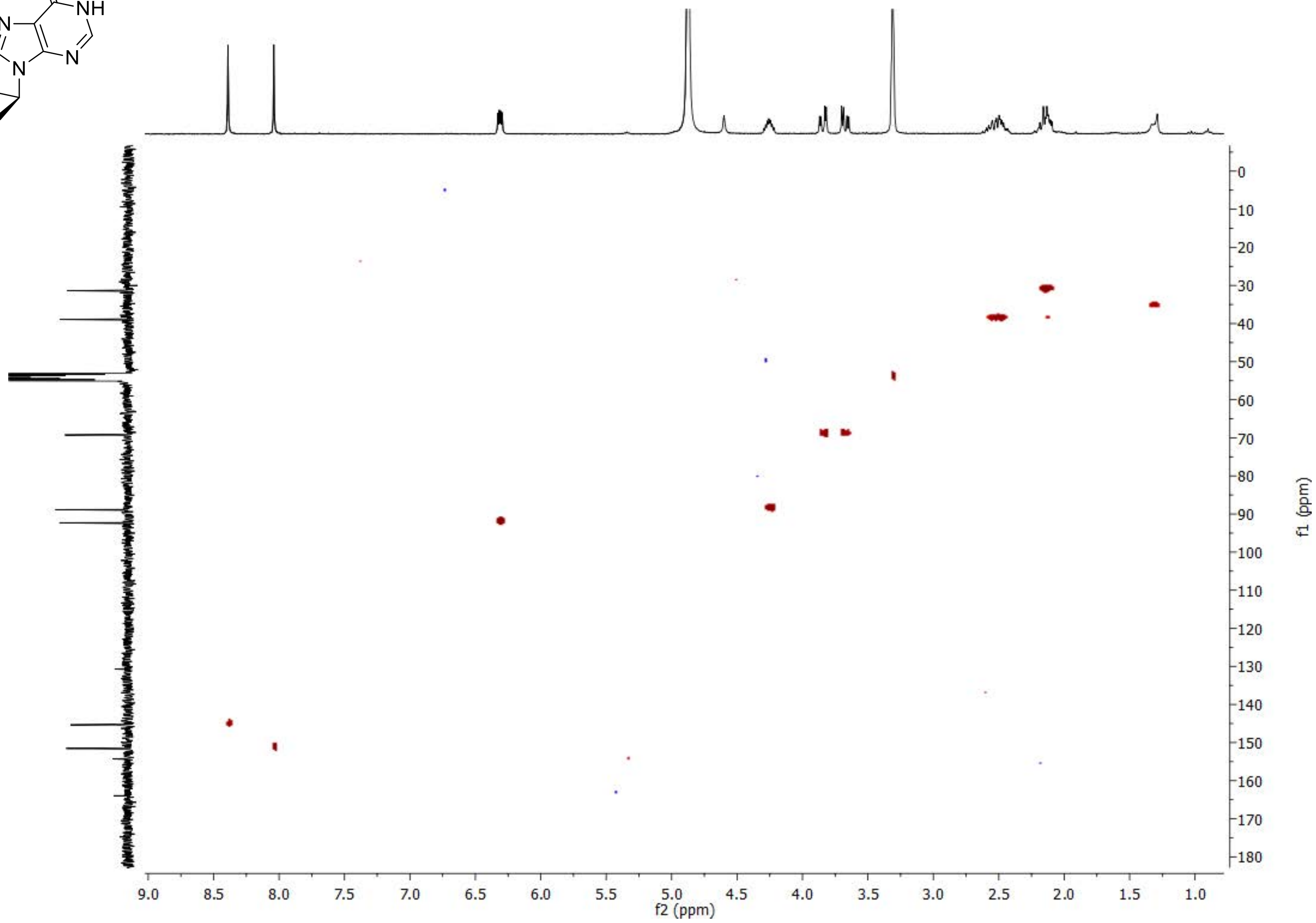

HMBC NMR (MeOH-d_4)

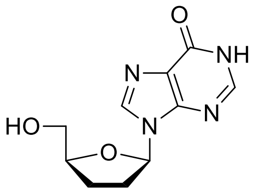

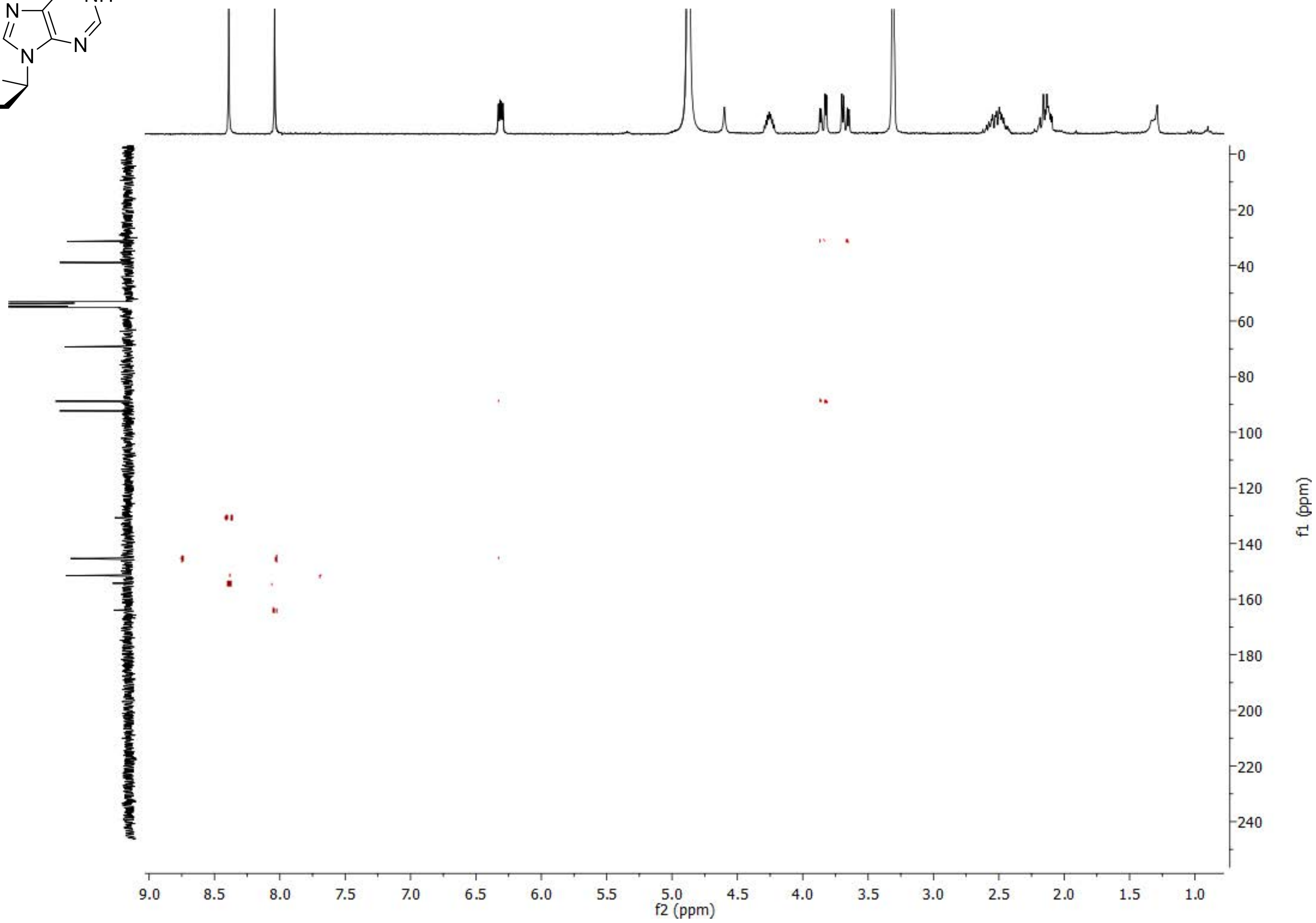

Supplement: Supplementary file 1 [file molecules-27-03993-s001.zip › molecules-1778358-supplementary.pdf]
